# Supplementary material for: Regio‐ and enantioselective nickel-alkyl catalyzed hydroalkylation of alkynes
Source: Nat Commun. 2024 Aug 2;15:6556. doi: 10.1038/s41467-024-50947-0 (PMC11297161; doi:10.1038/s41467-024-50947-0)
Supplement: Supplementary file 1 — Supplementary Information [file 41467_2024_50947_MOESM1_ESM.pdf]

Supplementary Information

For

Regio- and enantioselective nickel-alkyl catalyzed hydroalkylation of alkynes

Qian Gao[1, 2], Wei-Cheng Xu[2], Xuan Nie[1], Kang-Jie Bian[2], Hong-Rui Yuan[2],
Wen Zhang[2], Bing-Bing Wu[2] *, Xi-Sheng Wang[1, 2] *

[1]Department of Pharmacy, The First Affiliated Hospital of USTC, Division of Life Sciences and Medicine,
University of Science and Technology of China, Hefei 230026, China.
[2]Department of Chemistry, University of Science and Technology of China, Hefei 230026, China.

Table of Contents

Supplementary Notes 3

General Information 3

Supplementary Methods 4

Optimization of conditions 4

Supplementary Table 1. Optimization of Ligands 4

Supplementary Table 2. Optimization of Ni-Catalysts 4

Supplementary Table 3. Optimization of Bases 5

Supplementary Table 4. Optimization of Silanes 5

Supplementary Table 5. Optimization of Solvents 6

Supplementary Table 6. Optimization of Temperature 6

Supplementary Table 7. Optimization of Mixed Solvents 7

General Procedure for Regio- and enantioselective nickel-alkyl catalyzed hydroalkylation of alkynes 8

Synthetic Applications 28

General Procedure for Synthesis of Redox-Active NHP Esters 32

General Procedure for Synthesis of Alkynes 34

Supplementary Discussion 39

Mechanism Studies 39

Regioselectivity-Determining Step 39

Deuterium-Labeling Experiments 41

Radical Experiments. 45

Control Experiments. 47

Supplementary Table 8. Table of control experiments 47

X-ray Crystallographic Data of **28** 49

Supplementary Figures 50

NMR Spectra of New Compounds 50

HPLC Data 121

Supplementary References 172

Supplementary Notes

General Information

NMR spectra were recorded on Bruker-400 MHz NMR spectrometer (400 MHz for ^{1}H; 101 MHz for ^{13}C and 376 MHz for ^{19}F {^{13}C decoupled}), Bruker-500 MHz NMR spectrometer (500 MHz for ^{1}H; 126 MHz for ^{13}C and 470 MHz for ^{19}F {^{13}C decoupled}), Bruker-600 MHz NMR spectrometer (600 MHz for ^{1}H; 151 MHz for ^{13}C and 565 MHz for ^{19}F {^{13}C decoupled}). ^{1}H NMR chemical shifts were determined relative to internal $(CH_3)_4Si$ at δ 0.0 ppm or at the signal of a residual protonated solvent: $CDCl_3$ δ 7.26 ppm. ^{13}C NMR chemical shifts were determined relative to $CDCl_3$ δ 77.16 ppm. ^{19}F NMR chemical shifts were determined relative to $CFCl_3$ at δ 0.0 ppm. Data for ^{1}H, ^{13}C, ^{19}F NMR are recorded as follows: chemical shift (δ, ppm), multiplicity (s = singlet, d = doublet, t = triplet, m = multiplet, q = quartet et al.), integration, and coupling constant (Hz). High resolution mass spectra were recorded on P-SIMS-Gly of BrukerDaltonics Inc. using ESI-TOF (electrospray ionization-time of flight). $NiCl_2$·DME was obtained from Strem Chemicals. Trimethoxysilane was purchased from TCI. Calcium acetate was purchased from Sigma-Aldrich. Anhydrous NMP and THF were purchased from *J&K* Chemicals. Redox-active NHP esters and alkynes were synthesized via following method described in this supporting information.

Supplementary Methods

Optimization of conditions

Supplementary Table 1. Optimization of Ligands

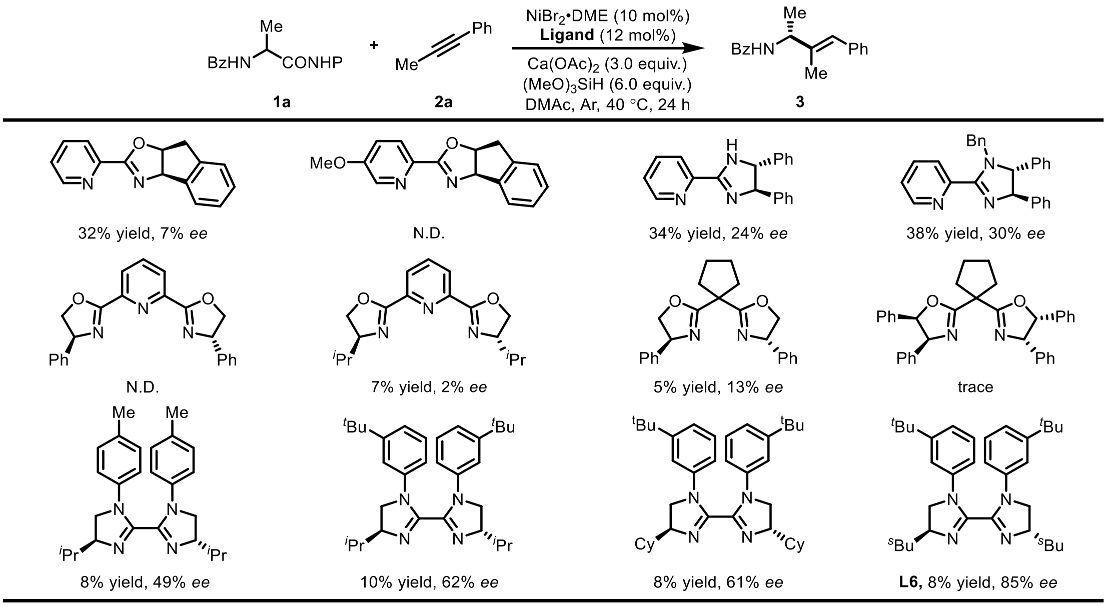

Unless otherwise noted, the reaction conditions were as follows: **1a** (0.10 mmol, 1.0 equiv.), **2a** (0.20 mmol, 2.0 equiv.), $NiBr_2$•DME (10 mol%), **Ligand** (12 mol%), $Ca(OAc)_2$ (0.30 mmol, 3.0 equiv.), $(MeO)_3SiH$ (0.60 mmol, 6.0 equiv.), DMAc (0.5 mL), Ar, 40 ˚C, 24 h; Isolated yield; The *ee* values were determined by HPLC on a chiral stationary phase.

Supplementary Table 2. Optimization of Ni-Catalysts

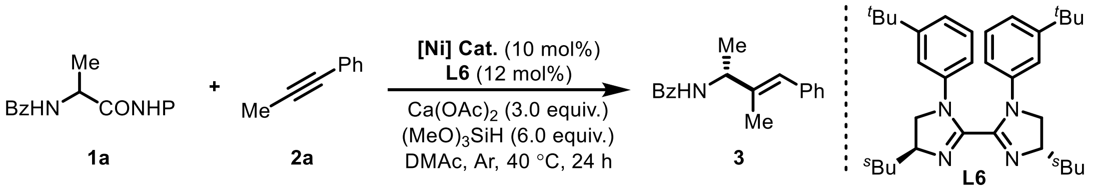

Entry	[Ni] cat.	Yield / %	*ee* / %
1	$NiBr_2$•DME	8	85
2	$NiCl_2$•DME	27	88
3	$NiCl_2$	20	87
4	$NiBr_2$	n.d.	-
5	NiI_2	n.d.	-
6	$NiCl_2$•$6H_2O$	19	48

Unless otherwise noted, the reaction conditions were as follows: **1a** (0.10 mmol, 1.0 equiv.), **2a** (0.20 mmol, 2.0 equiv.), **[Ni] cat.** (10 mol%), **L6** (12 mol%), $Ca(OAc)_2$ (0.30 mmol, 3.0 equiv.), $(MeO)_3SiH$ (0.60 mmol, 6.0 equiv.), DMAc (0.5 mL), Ar, 40 ˚C, 24 h; Isolated yield; The *ee* values were determined by HPLC on a chiral stationary phase.

Supplementary Table 3. Optimization of Bases

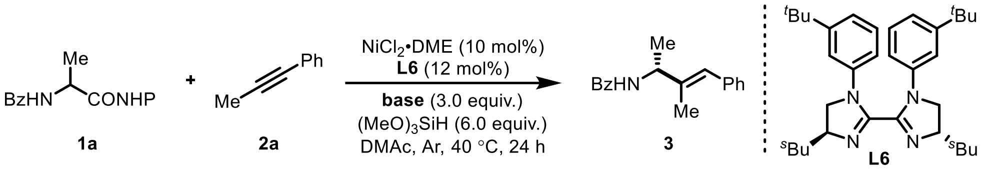

Entry	base	Yield / %	*ee* / %	Entry	base	Yield / %	*ee* / %
1	$Ca(OAc)_2$	27	88	7	$NaHCO_3$	8	91
2	NaOAc	n.d.	-	8	$KHCO_3$	n.d.	-
3	CsOAc	n.d.	-	9	Na_2HPO_4	14	85
4	Na_2CO_3	trace	90	10	K_3PO_4•H_2O	7	93
5	K_2CO_3	n.d.	-	11	CsF	n.d.	-
6	Cs_2CO_3	n.d.	-	12	CaF_2	11	86

Unless otherwise noted, the reaction conditions were as follows: **1a** (0.10 mmol, 1.0 equiv.), **2a** (0.20 mmol, 2.0 equiv.), $NiCl_2$•DME (10 mol%), **L6** (12 mol%), **base** (0.30 mmol, 3.0 equiv.), $(MeO)_3SiH$ (0.60 mmol, 6.0 equiv.), DMAc (0.5 mL), Ar, 40 °C, 24 h; Isolated yield; The *ee* values were determined by HPLC on a chiral stationary phase.

Supplementary Table 4. Optimization of Silanes

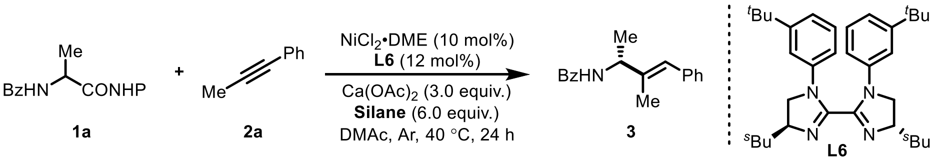

Entry	Silane	Yield / %	*ee* / %
1	$(MeO)_3SiH$	27	88
2	$(EtO)_3SiH$	n.d.	-
3	$(MeO)_2MeSiH$	n.d.	-
4	$(EtO)_2MeSiH$	n.d.	-
5	Et_3SiH	n.d.	-
6	PMHS	trace	84

Unless otherwise noted, the reaction conditions were as follows: **1a** (0.10 mmol, 1.0 equiv.), **2a** (0.20 mmol, 2.0 equiv.), $NiCl_2$•DME (10 mol%), **L6** (12 mol%), $Ca(OAc)_2$ (0.30 mmol, 3.0 equiv.), **Silane** (0.60 mmol, 6.0 equiv.), DMAc (0.5 mL), Ar, 40 °C, 24 h; Isolated yield; The *ee* values were determined by HPLC on a chiral stationary phase.

Supplementary Table 5. Optimization of Solvents

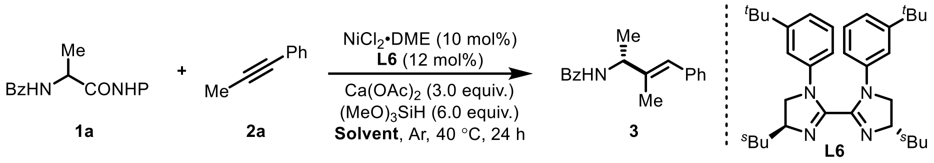

Entry	Solvent	Yield / %	*ee* / %
1	DMAc	27	88
2	DMF	5	77
3	NMP	37	89
4	THF	26	80
5	Dioxane	19	74
6	Toluene	8	58

Unless otherwise noted, the reaction conditions were as follows: **1a** (0.10 mmol, 1.0 equiv.), **2a** (0.20 mmol, 2.0 equiv.), $NiCl_2$•DME (10 mol%), **L6** (12 mol%), $Ca(OAc)_2$ (0.30 mmol, 3.0 equiv.), $(MeO)_3SiH$ (0.60 mmol, 6.0 equiv.), **Solvent** (0.5 mL), Ar, 40 °C, 24 h; Isolated yield; The *ee* values were determined by HPLC on a chiral stationary phase.

Supplementary Table 6. Optimization of Temperature

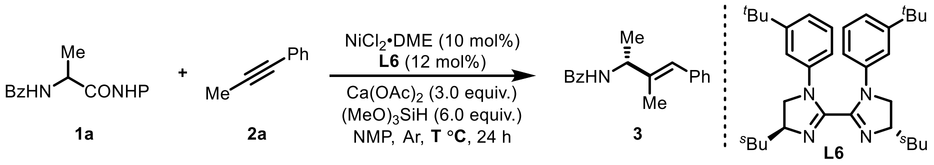

Entry	T / °C	Yield / %	*ee* / %
1	40	37	89
2	30	39	88
3	20	45	95
4	10	48	95
5	0	52	96
6	-10	30	96

Unless otherwise noted, the reaction conditions were as follows: **1a** (0.10 mmol, 1.0 equiv.), **2a** (0.20 mmol, 2.0 equiv.), $NiCl_2$•DME (10 mol%), **L6** (12 mol%), $Ca(OAc)_2$ (0.30 mmol, 3.0 equiv.), $(MeO)_3SiH$ (0.60 mmol, 6.0 equiv.), NMP (0.5 mL), Ar, **T** °C, 24 h; Isolated yield; The *ee* values were determined by HPLC on a chiral stationary phase.

Supplementary Table 7. Optimization of Mixed Solvents

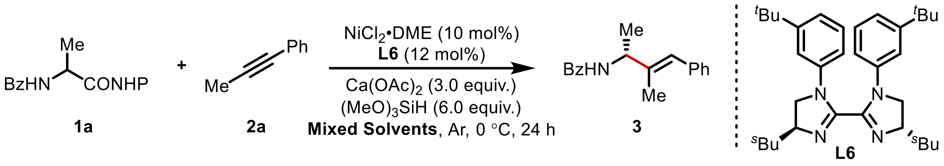

Entry	Mixed Solvents	Yield / %	*ee* / %
1	NMP/MeOH (7 / 1)	42	96
2	NMP/CH_3CN (7 / 1)	55	97
3	NMP/Toluene (7 / 1)	45	95
4	NMP/Dioxane (7 / 1)	52	91
5	NMP/DCM (7 / 1)	36	94
6	NMP/DME (7 / 1)	44	94
7	NMP/THF (7 / 1)	61	95
8	NMP/THF (11.5 / 1)	61	96
9	NMP/THF (4 / 1)	70	96
10	NMP/THF (2 / 1)	71	96
11	NMP/THF (1 / 1)	55	96
12	NMP/THF (1 / 2)	43	96

Unless otherwise noted, the reaction conditions were as follows: **1a** (0.10 mmol, 1.0 equiv.), **2a** (0.20 mmol, 2.0 equiv.), $NiCl_2$•DME (10 mol%), **L6** (12 mol%), $Ca(OAc)_2$ (0.30 mmol, 3.0 equiv.), $(MeO)_3SiH$ (0.60 mmol, 6.0 equiv.), **Mixed Solvents** (0.5 mL), Ar, 0 °C, 24 h; Isolated yield; The *ee* values were determined by HPLC on a chiral stationary phase.

General Procedure for Regio- and enantioselective nickel-alkyl catalyzed hydroalkylation of alkynes

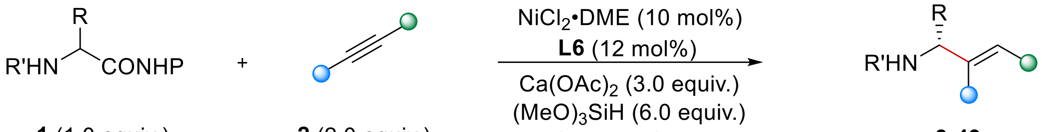

In glove box, $NiCl_2$•DME (0.01 mmol, 10 mol%), L6 (0.012 mmol, 12 mol%), $Ca(OAc)_2$ (0.3 mmol, 3.0 equiv.) and alkyl NHP ester **1** (0.10 mmol, 1.0 equiv.) were combined in a 5 mL oven-dried sealing tube. The vessel was evacuated and backfilled with Ar (repeated for 3 times). Alkyne **2** (0.20 mmol, 2.0 equiv.), $(MeO)_3SiH$ (0.60 mmol, 6.0 equiv.) and NMP/THF (v/v = 2/1, 0.5 mL) were then added via syringe. The tube was sealed with a Teflon lined cap and stirred at 0 ˚C for 24-48 h. The reaction mixture was then diluted with EtOAc (~20 mL) and filtered through a pad of celite. The filtrate was added brine (20 mL) and extracted with EtOAc (2×15 mL), the combined organic layer was dried over Na_2SO_4, filtrated and concentrated under vacuum. The residue was then purified by flash column chromatography to give desired product as a solid or oil.

(R,E)-N-(3-methyl-4-phenylbut-3-en-2-yl)benzamide

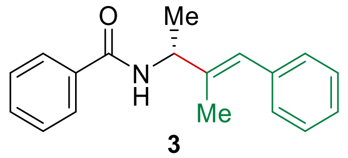

3

The product **3** was purified with silica gel chromatography (PE/EA = 10:1) as a white solid (71%). ^{1}H NMR (500 MHz, Chloroform-*d*) δ 7.86 – 7.76 (m, 2H), 7.55 – 7.48 (m, 1H), 7.44 (t, *J* = 7.5 Hz, 2H), 7.33 (t, *J* = 7.6 Hz, 2H), 7.28 – 7.24 (m, 2H), 7.24 – 7.18 (m, 1H), 6.54 (s, 1H), 6.23 (s, 1H), 4.89 – 4.80 (m, 1H), 1.93 (d, *J* = 1.3 Hz, 3H), 1.46 (d, *J* = 6.8 Hz, 3H). ^{13}C NMR (126 MHz, Chloroform-*d*) δ 166.84, 138.89, 137.66, 134.85, 131.58, 129.12, 128.72, 128.20, 127.03, 126.57, 125.20, 52.07, 19.97, 15.56. HRMS (ESI): m/z calcd. for $C_{18}H_{19}NONa^+$ [M + Na$^+$]: 288.1359, found: 288.1361. $[\alpha]_D^{20}$ = 7.1 (c = 1.0, $CHCl_3$), HPLC chiralcel AD-H column (10% isopropanol in hexanes, 1.0 mL/min, λ = 254 nm), t_R = 8.8 min (major), 12.0 min (minor), 96% *ee*.

(R,E)-N-(3-methyl-4-(naphthalen-2-yl)but-3-en-2-yl)benzamide

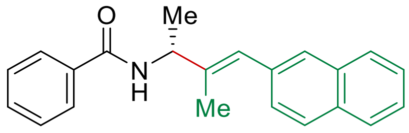

4

The product **4** was purified with silica gel chromatography (PE/EA = 10:1) as a white solid (61%). ^{1}H NMR (400 MHz, Chloroform-*d*) δ 7.88 – 7.76 (m, 5H), 7.76 – 7.69 (m, 1H), 7.55 – 7.49 (m, 1H), 7.49 – 7.42 (m, 4H), 7.40 (dd, *J* = 8.5, 1.8 Hz, 1H), 6.70 (s, 1H), 6.23 (d, *J* = 8.2 Hz, 1H), 4.96 –

4.84 (m, 1H), 2.01 (d, *J* = 1.4 Hz, 3H), 1.51 (d, *J* = 6.9 Hz, 3H). ^{13}C NMR (126 MHz, Chloroform-*d*) δ 166.87, 139.41, 135.22, 134.86, 133.39, 132.20, 131.64, 128.76, 127.99, 127.81, 127.71, 127.67, 127.60, 127.05, 126.18, 125.82, 125.25, 52.16, 20.05, 15.71. HRMS (ESI): m/z calcd. for $C_{22}H_{21}NONa^+$ [M + Na$^+$]: 338.1515, found: 338.1522. $[\alpha]_D^{20}$ = 2.4 (c = 1.0, $CHCl_3$), HPLC chiralcel AD-H column (10% isopropanol in hexanes, 1.0 mL/min, λ = 245 nm), t_R = 12.0 min (major), 17.3 min (minor), 91% *ee*.

(R,E)-N-(3-methyl-4-(o-tolyl)but-3-en-2-yl)benzamide

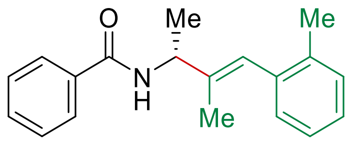

5

The product **5** was purified with silica gel chromatography (PE/EA = 10:1) as a white solid (69%). ^{1}H NMR (500 MHz, Chloroform-*d*) δ 7.83 – 7.76 (m, 2H), 7.54 – 7.47 (m, 1H), 7.48 – 7.41 (m, 2H), 7.20 – 7.10 (m, 4H), 6.53 (s, 1H), 6.23 (d, *J* = 8.0 Hz, 1H), 4.90 – 4.80 (m, 1H), 2.23 (s, 3H), 1.77 (d, *J* = 1.3 Hz, 3H), 1.47 (d, *J* = 6.9 Hz, 3H). ^{13}C NMR (126 MHz, Chloroform-*d*) δ 166.90, 138.89, 136.84, 136.58, 134.98, 131.55, 129.85, 129.28, 128.73, 126.99, 126.85, 125.41, 124.24, 51.74, 20.10, 19.97, 15.10. HRMS (ESI): m/z calcd. for $C_{19}H_{21}NOH^+$ [M + H$^+$]: 280.1696, found: 280.1697. $[\alpha]_D^{20}$ = 8.2 (c = 1.0, $CHCl_3$), HPLC chiralcel AD-H column (10% isopropanol in hexanes, 1.0 mL/min, λ = 245 nm), t_R = 7.1 min (major), 9.4 min (minor), 96% *ee*.

(R,E)-N-(3-methyl-4-(m-tolyl)but-3-en-2-yl)benzamide

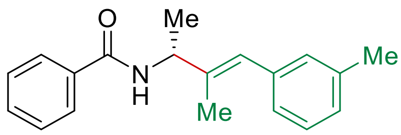

6

The product **6** was purified with silica gel chromatography (PE/EA = 10:1) as a white solid (66%). ^{1}H NMR (500 MHz, Chloroform-*d*) δ 7.83 – 7.78 (m, 2H), 7.54 – 7.47 (m, 1H), 7.47 – 7.40 (m, 2H), 7.24 – 7.17 (m, 1H), 7.11 – 6.99 (m, 3H), 6.52 (s, 1H), 6.22 (s, 1H), 4.88 – 4.79 (m, 1H), 2.34 (s, 3H), 1.93 (d, *J* = 1.4 Hz, 3H), 1.45 (d, *J* = 6.9 Hz, 3H). ^{13}C NMR (126 MHz, Chloroform-*d*) δ 166.83, 138.69, 137.73, 137.61, 134.89, 131.57, 129.89, 128.71, 128.09, 127.34, 127.03, 126.13, 125.29, 52.08, 21.56, 19.97, 15.60. HRMS (ESI): m/z calcd. for $C_{19}H_{21}NONa^+$ [M + Na$^+$]: 302.1515, found: 302.1516. $[\alpha]_D^{20}$ = 5.6 (c = 1.0, $CHCl_3$), HPLC chiralcel AD-H column (10% isopropanol in hexanes, 1.0 mL/min, λ = 245 nm), t_R = 7.5 min (major), 10.4 min (minor), 92% *ee*.

(R,E)-N-(3-methyl-4-(p-tolyl)but-3-en-2-yl)benzamide

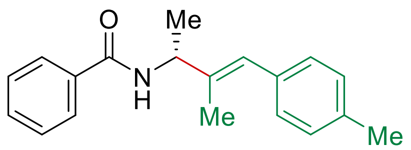

7

The product **7** was purified with silica gel chromatography (PE/EA = 10:1) as a white solid (62%). ^{1}H NMR (500 MHz, Chloroform-*d*) δ 7.83 – 7.77 (m, 2H), 7.55 – 7.47 (m, 1H), 7.47 – 7.40 (m, 2H), 7.19 – 7.11 (m, 4H), 6.51 (s, 1H), 6.19 (d, *J* = 8.3 Hz, 1H), 4.88 – 4.79 (m, 1H), 2.34 (s, 3H), 1.93 (d, *J* = 1.4 Hz, 3H), 1.45 (d, *J* = 6.9 Hz, 3H). ^{13}C NMR (126 MHz, Chloroform-*d*) δ 166.85, 138.09, 136.25, 134.90, 134.73, 131.57, 129.03, 128.92, 128.72, 127.02, 125.12, 52.11, 21.29, 19.98, 15.62. HRMS (ESI): m/z calcd. for $C_{19}H_{21}NONa^{+}$ [M + Na^{+}]: 302.1515, found: 302.1519. $[\alpha]_D^{20}$ = 5.9 (c = 1.0, $CHCl_3$), HPLC chiralcel AD-H column (10% isopropanol in hexanes, 1.0 mL/min, λ = 245 nm), t_R = 8.5 min (major), 10.5 min (minor), 91% *ee*.

(R,E)-N-(4-(3,5-dimethylphenyl)-3-methylbut-3-en-2-yl)benzamide

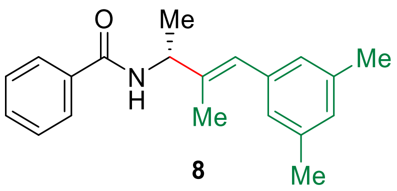

8

The product **8** was purified with silica gel chromatography (PE/EA = 10:1) as a white solid (60%). ^{1}H NMR (400 MHz, Chloroform-*d*) δ 7.83 – 7.77 (m, 2H), 7.55 – 7.47 (m, 1H), 7.47 – 7.39 (m, 2H), 6.89 (s, 2H), 6.87 (s, 1H), 6.49 (s, 1H), 6.19 (s, 1H), 4.88 – 4.77 (m, 1H), 2.31 (s, 6H), 1.93 (d, *J* = 1.3 Hz, 3H), 1.45 (d, *J* = 6.8 Hz, 3H). ^{13}C NMR (101 MHz, Chloroform-*d*) δ 166.82, 138.49, 137.64, 137.57, 134.93, 131.56, 128.72, 128.26, 127.03, 126.93, 125.36, 52.07, 21.45, 19.97, 15.66. HRMS (ESI): m/z calcd. for $C_{20}H_{23}NONa^{+}$ [M + Na^{+}]: 316.1672, found: 316.1675. $[\alpha]_D^{20}$ = 6.0 (c = 1.0, $CHCl_3$), HPLC chiralcel AD-H column (10% isopropanol in hexanes, 1.0 mL/min, λ = 245 nm), t_R = 6.7 min (major), 9.7 min (minor), 95% *ee*.

(R,E)-N-(4-(4-(tert-butyl)phenyl)-3-methylbut-3-en-2-yl)benzamide

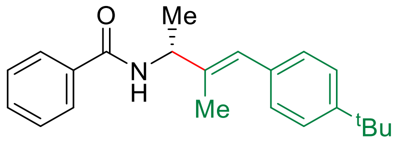

9

The product **9** was purified with silica gel chromatography (PE/EA = 10:1) as a white solid (30%). ^{1}H NMR (400 MHz, Chloroform-*d*) δ 7.86 – 7.73 (m, 2H), 7.54 – 7.48 (m, 1H), 7.48 – 7.40 (m, 2H), 7.38 – 7.31 (m, 2H), 7.24 – 7.19 (m, 2H), 6.51 (s, 1H), 6.16 (d, *J* = 8.3 Hz, 1H), 4.89 – 4.78 (m, 1H), 1.95 (d, *J* = 1.3 Hz, 3H), 1.45 (d, *J* = 6.8 Hz, 3H), 1.32 (s, 9H). ^{13}C NMR (101 MHz, Chloroform-*d*) δ 166.86, 149.53, 138.21, 134.93, 134.73, 131.58, 128.84, 128.74, 127.01, 125.15,

125.06, 52.14, 34.63, 31.45, 20.00, 15.70. HRMS (ESI): m/z calcd. for $C_{22}H_{27}NONa^+$ [M + Na^+]: 344.1985, found: 344.1991. $[\alpha]_D^{20}$ = 8.6 (c = 1.0, $CHCl_3$), HPLC chiralcel OD-H column (10% isopropanol in hexanes, 1.0 mL/min, λ = 245 nm), t_R = 8.2 min (major), 13.3 min (minor), 97% *ee*.

(R,E)-N-(4-(4-fluorophenyl)-3-methylbut-3-en-2-yl)benzamide

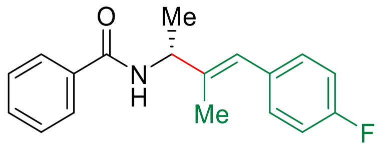

10

The product **10** was purified with silica gel chromatography (PE/EA = 10:1) as a white solid (72%). ^1H NMR (400 MHz, Chloroform-*d*) δ 7.84 – 7.75 (m, 2H), 7.55 – 7.48 (m, 1H), 7.47 – 7.40 (m, 2H), 7.25 – 7.17 (m, 2H), 7.08 – 6.94 (m, 2H), 6.49 (s, 1H), 6.20 (d, *J* = 8.2 Hz, 1H), 4.88 – 4.76 (m, 1H), 1.89 (d, *J* = 1.4 Hz, 3H), 1.45 (d, *J* = 6.9 Hz, 3H). ^{19}F NMR (376 MHz, Chloroform-*d*) δ -115.80. ^{13}C NMR (101 MHz, Chloroform-*d*) δ 166.86, 161.50 (d, *J* = 245.8 Hz), 138.84 (d, *J* = 1.4 Hz), 134.78, 133.65 (d, *J* = 3.4 Hz), 131.65, 130.66 (d, *J* = 7.8 Hz), 128.75, 127.02, 124.17, 115.09 (d, *J* = 21.3 Hz), 52.09, 19.98, 15.35. HRMS (ESI): m/z calcd. for $C_{18}H_{18}FNONa^+$ [M + Na^+]: 306.1265, found: 306.1268. $[\alpha]_D^{20}$ = 1.0 (c = 1.0, $CHCl_3$), HPLC chiralcel AD-H column (10% isopropanol in hexanes, 1.0 mL/min, λ = 245 nm), t_R = 9.8 min (major), 13.4 min (minor), 94% *ee*.

(R,E)-N-(4-(4-chlorophenyl)-3-methylbut-3-en-2-yl)benzamide

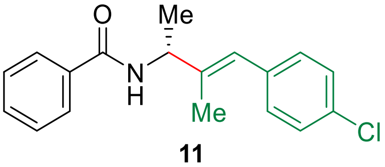

11

The product **11** was purified with silica gel chromatography (PE/EA = 10:1) as a white solid (70%). ^1H NMR (400 MHz, Chloroform-*d*) δ 7.84 – 7.75 (m, 2H), 7.55 – 7.48 (m, 1H), 7.48 – 7.40 (m, 2H), 7.31 – 7.26 (m, 2H), 7.23 – 7.14 (m, 2H), 6.48 (s, 1H), 6.19 (d, *J* = 8.2 Hz, 1H), 4.88 – 4.76 (m, 1H), 1.90 (d, *J* = 1.5 Hz, 3H), 1.44 (d, *J* = 6.9 Hz, 3H). ^{13}C NMR (101 MHz, Chloroform-*d*) δ 166.86, 139.69, 136.11, 134.73, 132.25, 131.68, 130.42, 128.76, 128.36, 127.02, 124.07, 52.11, 19.98, 15.44. HRMS (ESI): m/z calcd. for $C_{18}H_{18}ClNONa^+$ [M + Na^+]: 322.0969, found: 322.0974. $[\alpha]_D^{20}$ = 0.8 (c = 1.0, $CHCl_3$), HPLC chiralcel AD-H column (10% isopropanol in hexanes, 1.0 mL/min, λ = 245 nm), t_R = 10.5 min (major), 14.2 min (minor), 91% *ee*.

(R,E)-N-(4-(4-methoxyphenyl)-3-methylbut-3-en-2-yl)benzamide

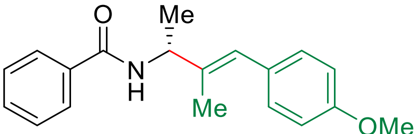

12

The product **12** was purified with silica gel chromatography (PE/EA = 10:1) as a white solid (44%). ^{1}H NMR (400 MHz, Chloroform-*d*) δ 7.83 – 7.76 (m, 2H), 7.54 – 7.48 (m, 1H), 7.47 – 7.41 (m, 2H), 7.24 – 7.16 (m, 2H), 6.92 – 6.82 (m, 2H), 6.48 (s, 1H), 6.16 (d, *J* = 8.3 Hz, 1H), 4.88 – 4.76 (m, 1H), 3.81 (s, 3H), 1.92 (d, *J* = 1.3 Hz, 3H), 1.45 (d, *J* = 6.8 Hz, 3H). ^{13}C NMR (101 MHz, Chloroform-*d*) δ 166.86, 158.23, 137.21, 134.90, 131.59, 130.31, 130.19, 128.74, 127.02, 124.76, 113.63, 55.37, 52.19, 20.00, 15.58. HRMS (ESI): m/z calcd. for $C_{19}H_{21}NO_2Na^+$ [M + Na$^+$]: 318.1465, found: 318.1467. $[\alpha]_D^{20}$ = 3.8 (c = 1.0, $CHCl_3$), HPLC chiralcel AD-H column (10% isopropanol in hexanes, 1.0 mL/min, λ = 254 nm), t_R = 12.8 min (major), 15.4 min (minor), 96% *ee*.

(R,E)-N-(4-(3-methoxyphenyl)-3-methylbut-3-en-2-yl)benzamide

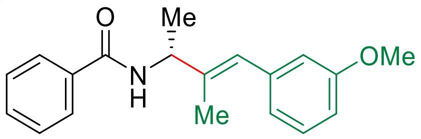

13

The product **13** was purified with silica gel chromatography (PE/EA = 10:1) as a white solid (51%). ^{1}H NMR (500 MHz, Chloroform-*d*) δ 7.83 – 7.76 (m, 2H), 7.53 – 7.49 (m, 1H), 7.48 – 7.42 (m, 2H), 7.24 (t, *J* = 7.9 Hz, 1H), 6.89 – 6.83 (m, 1H), 6.82 – 6.74 (m, 2H), 6.52 (s, 1H), 6.16 (d, *J* = 8.3 Hz, 1H), 4.88 – 4.79 (m, 1H), 3.80 (s, 3H), 1.93 (d, *J* = 1.3 Hz, 3H), 1.46 (d, *J* = 6.9 Hz, 3H). ^{13}C NMR (101 MHz, Chloroform-*d*) δ 166.83, 159.46, 139.20, 139.10, 134.86, 131.64, 129.19, 128.76, 127.02, 125.14, 121.67, 114.62, 112.23, 55.35, 52.06, 20.01, 15.66. HRMS (ESI): m/z calcd. for $C_{19}H_{21}NO_2Na^+$ [M + Na$^+$]: 318.1465, found: 318.1468. $[\alpha]_D^{20}$ = 2.1 (c = 1.0, $CHCl_3$), HPLC chiralcel AD-H column (10% isopropanol in hexanes, 1.0 mL/min, λ = 254 nm), t_R = 11.2 min (major), 19.1 min (minor), 96% *ee*.

(R,E)-N-(3-methyl-4-(4-(trifluoromethoxy)phenyl)but-3-en-2-yl)benzamide

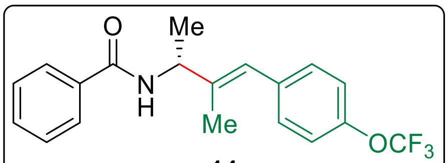

14

The product **14** was purified with silica gel chromatography (PE/EA = 10:1) as a white solid (55%). ^{1}H NMR (400 MHz, Chloroform-*d*) δ 7.84 – 7.76 (m, 2H), 7.55 – 7.48 (m, 1H), 7.48 – 7.40 (m, 2H), 7.30 – 7.23 (m, 2H), 7.19 – 7.09 (m, 2H), 6.51 (s, 1H), 6.19 (s, 1H), 4.89 – 4.77 (m, 1H), 1.91 (d, *J* = 1.3 Hz, 3H), 1.45 (d, *J* = 6.9 Hz, 3H). ^{19}F NMR (376 MHz, Chloroform-*d*) δ -57.83. ^{13}C NMR (101 MHz, Chloroform-*d*) δ 166.85, 147.69 (q, *J* = 2.2 Hz), 139.96, 136.43, 134.73, 131.69, 130.42, 128.77, 127.02, 123.89, 120.73, 120.60 (q, *J* = 257.6 Hz), 52.09, 19.98, 15.37. HRMS (ESI): m/z calcd. for $C_{19}H_{18}F_3NO_2Na^+$ [M + Na$^+$]: 372.1182, found: 372.1187. $[\alpha]_D^{20}$ = 2.1 (c = 1.0, $CHCl_3$), HPLC chiralcel AD-H column (10% isopropanol in hexanes, 1.0 mL/min, λ = 245 nm), t_R = 8.9 min

(major), 11.2 min (minor), 96% *ee*.

methyl (R,E)-3-(3-benzamido-2-methylbut-1-en-1-yl)benzoate

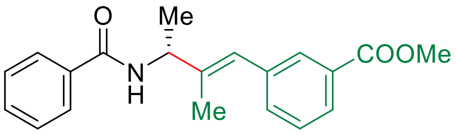

15

The product **15** was purified with silica gel chromatography (PE/EA = 10:1) as a white solid (47%). ^{1}H NMR (400 MHz, Chloroform-*d*) δ 7.93 (t, *J* = 1.7 Hz, 1H), 7.88 (dt, *J* = 7.5, 1.6 Hz, 1H), 7.84 – 7.77 (m, 2H), 7.56 – 7.49 (m, 1H), 7.48 – 7.42 (m, 3H), 7.42 – 7.36 (m, 1H), 6.56 (s, 1H), 6.18 (d, *J* = 8.2 Hz, 1H), 4.90 – 4.78 (m, 1H), 3.91 (s, 3H), 1.93 (d, *J* = 1.3 Hz, 3H), 1.46 (d, *J* = 6.9 Hz, 3H). ^{13}C NMR (101 MHz, Chloroform-*d*) δ 167.30, 166.88, 140.25, 137.99, 134.74, 133.59, 131.70, 130.22, 130.11, 128.78, 128.33, 127.72, 127.04, 124.25, 52.31, 52.07, 20.01, 15.48. HRMS (ESI): m/z calcd. for $C_{20}H_{21}NO_3Na^+$ [M + Na$^+$]: 346.1414, found: 346.1424. $[\alpha]_D{}^{20}$ = 1.1 (c = 1.0, $CHCl_3$), HPLC chiralcel AD-H column (10% isopropanol in hexanes, 1.0 mL/min, λ = 245 nm), t_R = 14.6 min (major), 26.0 min (minor), 96% *ee*.

(R,E)-N-(3-methyl-4-(thiophen-2-yl)but-3-en-2-yl)benzamide

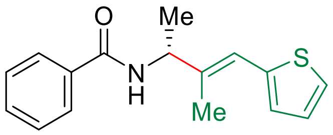

16

The product **16** was purified with silica gel chromatography (PE/EA = 10:1) as a yellow solid (40%). ^{1}H NMR (500 MHz, Chloroform-*d*) δ 7.85 – 7.71 (m, 2H), 7.54 – 7.47 (m, 1H), 7.47 – 7.39 (m, 2H), 7.26 – 7.24 (m, 1H), 7.04 – 6.98 (m, 2H), 6.68 (s, 1H), 6.15 (d, *J* = 8.2 Hz, 1H), 4.88 – 4.79 (m, 1H), 2.05 (d, *J* = 1.4 Hz, 3H), 1.44 (d, *J* = 6.8 Hz, 3H). ^{13}C NMR (126 MHz, Chloroform-*d*) δ 166.85, 140.66, 137.04, 134.73, 131.66, 128.75, 127.43, 127.03, 126.94, 125.14, 118.78, 52.42, 19.90, 16.29. HRMS (ESI): m/z calcd. for $C_{16}H_{17}NOSNa^+$ [M + Na$^+$]: 294.0923, found: 294.0918. $[\alpha]_D{}^{20}$ = 1.8 (c = 1.0, $CHCl_3$), HPLC chiralcel AD-H column (10% isopropanol in hexanes, 1.0 mL/min, λ = 272 nm), t_R = 11.7 min (major), 16.5 min (minor), 88% *ee*.

(R,E)-N-(3-benzylidenepentan-2-yl)benzamide

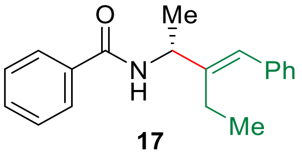

17

The product **17** was purified with silica gel chromatography (PE/EA = 10:1) as a white solid (50%). ^{1}H NMR (400 MHz, Chloroform-*d*) δ 7.83 – 7.76 (m, 2H), 7.55 – 7.47 (m, 1H), 7.47 – 7.38 (m, 2H),

7.40 – 7.27 (m, 2H), 7.27 – 7.14 (m, 3H), 6.52 (s, 1H), 6.15 (d, *J* = 8.4 Hz, 1H), 5.01 – 4.87 (m, 1H), 2.35 (qd, *J* = 7.5, 1.6 Hz, 2H), 1.48 (d, *J* = 6.8 Hz, 3H), 1.17 (t, *J* = 7.6 Hz, 3H). ^{13}C NMR (101 MHz, Chloroform-*d*) δ 166.79, 145.70, 137.65, 134.82, 131.61, 128.75, 128.75, 128.35, 127.01, 126.69, 124.68, 49.04, 22.95, 20.36, 13.68. HRMS (ESI): m/z calcd. for $C_{19}H_{21}NONa^+$ [M + Na$^+$]: 302.1515, found: 302.1522. $[\alpha]_D^{20}$ = 15.1 (c = 1.0, $CHCl_3$), HPLC chiralcel AD-H column (10% isopropanol in hexanes, 1.0 mL/min, λ = 245 nm), t_R = 7.2 min (major), 10.0 min (minor), 97% *ee*.

(R,E)-N-(3-benzylidenehexan-2-yl)benzamide

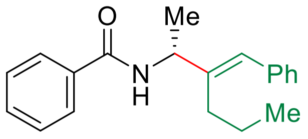

18

The product **18** was purified with silica gel chromatography (PE/EA = 10:1) as a white solid (56%). ^{1}H NMR (500 MHz, Chloroform-*d*) δ 7.84 – 7.73 (m, 2H), 7.54 – 7.47 (m, 1H), 7.48 – 7.41 (m, 2H), 7.33 (td, *J* = 7.3, 1.6 Hz, 2H), 7.26 – 7.19 (m, 3H), 6.54 (s, 1H), 6.17 (d, *J* = 8.3 Hz, 1H), 4.95 – 4.85 (m, 1H), 2.35 – 2.24 (m, 2H), 1.65 – 1.53 (m, 2H), 1.47 (d, *J* = 6.9 Hz, 3H), 0.92 (t, *J* = 7.3 Hz, 3H). ^{13}C NMR (126 MHz, Chloroform-*d*) δ 166.77, 144.42, 137.73, 134.83, 131.60, 128.78, 128.74, 128.33, 127.00, 126.64, 125.05, 49.34, 32.12, 22.16, 20.37, 14.44. HRMS (ESI): m/z calcd. for $C_{20}H_{23}NOH^+$ [M + H$^+$]: 294.1852, found: 294.1855. $[\alpha]_D^{20}$ = 8.7 (c = 1.0, $CHCl_3$), HPLC chiralcel AD-H column (10% isopropanol in hexanes, 1.0 mL/min, λ = 245 nm), t_R = 6.4 min (major), 8.8 min (minor), 92% *ee*.

(R,E)-N-(3-benzylidene-5-methylhexan-2-yl)benzamide

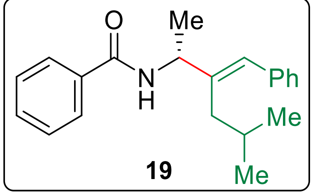

19

The product **19** was purified with silica gel chromatography (PE/EA = 10:1) as a white solid (52%). ^{1}H NMR (500 MHz, Chloroform-*d*) δ 7.83 – 7.77 (m, 2H), 7.56 – 7.48 (m, 1H), 7.48 – 7.35 (m, 2H), 7.38 – 7.27 (m, 2H), 7.25 – 7.18 (m, 3H), 6.61 (s, 1H), 6.13 (d, *J* = 8.5 Hz, 1H), 4.95 – 4.86 (m, 1H), 2.24 (d, *J* = 7.1 Hz, 2H), 2.03 – 1.88 (m, 1H), 1.47 (d, *J* = 6.9 Hz, 3H), 0.86 (d, *J* = 6.6 Hz, 3H), 0.84 (d, *J* = 6.6 Hz, 3H). ^{13}C NMR (126 MHz, Chloroform-*d*) δ 166.73, 143.46, 137.97, 134.86, 131.62, 129.03, 128.77, 128.27, 127.00, 126.54, 125.67, 48.92, 38.63, 26.83, 22.78, 22.65, 20.41. HRMS (ESI): m/z calcd. for $C_{21}H_{25}NOH^+$ [M + H$^+$]: 308.2009, found: 308.2012. $[\alpha]_D^{20}$ = 6.4 (c = 1.0, $CHCl_3$), HPLC chiralcel AD-H column (10% isopropanol in hexanes, 1.0 mL/min, λ = 254 nm), t_R = 5.5 min (minor), 7.0 min (major), 87% *ee*.

(R,E)-N-(3-benzylidenedecan-2-yl)benzamide

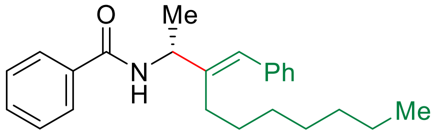

20

The product **20** was purified with silica gel chromatography (PE/EA = 10:1) as a white solid (34%). ^{1}H NMR (500 MHz, Chloroform-*d*) δ 7.83 – 7.77 (m, 2H), 7.54 – 7.48 (m, 1H), 7.48 – 7.41 (m, 2H), 7.41 – 7.29 (m, 2H), 7.25 – 7.19 (m, 3H), 6.53 (s, 1H), 6.19 – 6.11 (m, 1H), 4.94 – 4.85 (m, 1H), 2.36 – 2.25 (m, 2H), 1.61 – 1.51 (m, 2H), 1.47 (d, *J* = 6.9 Hz, 3H), 1.35 – 1.13 (m, 8H), 0.85 (t, *J* = 6.9 Hz, 3H). ^{13}C NMR (126 MHz, Chloroform-*d*) δ 166.75, 144.63, 137.74, 134.87, 131.58, 128.78, 128.73, 128.32, 127.00, 126.63, 124.89, 49.40, 31.90, 30.06, 29.94, 29.11, 28.85, 22.77, 20.41, 14.23. HRMS (ESI): m/z calcd. for $C_{24}H_{31}NOH^{+}$ [M + H^{+}]: 350.2478, found: 350.2478. $[\alpha]_D{}^{20}$ = 21.8 (c = 1.0, $CHCl_3$), HPLC chiralcel AD-H column (10% isopropanol in hexanes, 1.0 mL/min, λ = 245 nm), t_R = 5.3 min (major), 6.7 min (minor), 92% *ee*.

(R,E)-5-benzamido-4-benzylidenehexyl benzoate

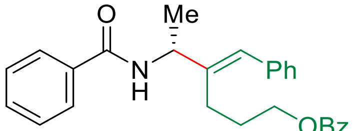

21

The product **21** was purified with silica gel chromatography (PE/EA = 5:1) as a white solid (42%). ^{1}H NMR (500 MHz, Chloroform-*d*) δ 7.98 – 7.88 (m, 2H), 7.85 – 7.75 (m, 2H), 7.57 – 7.47 (m, 2H), 7.47 – 7.40 (m, 2H), 7.42 – 7.35 (m, 2H), 7.29 – 7.21 (m, 4H), 7.21 – 7.14 (m, 1H), 6.61 (s, 1H), 6.18 (d, *J* = 8.3 Hz, 1H), 5.00 – 4.91 (m, 1H), 4.37 – 4.25 (m, 2H), 2.58 – 2.47 (m, 2H), 2.14 – 1.96 (m, 2H), 1.50 (d, *J* = 6.8 Hz, 3H). ^{13}C NMR (126 MHz, Chloroform-*d*) δ 166.83, 166.67, 143.14, 137.43, 134.69, 132.93, 131.69, 130.37, 129.70, 128.78, 128.70, 128.43, 128.40, 127.02, 126.81, 125.90, 64.74, 48.98, 27.88, 26.51, 20.35. HRMS (ESI): m/z calcd. for $C_{27}H_{27}NO_3Na^{+}$ [M + Na^{+}]: 436.1883, found: 436.1883. $[\alpha]_D{}^{20}$ = 5.6 (c = 1.0, $CHCl_3$), HPLC chiralcel AD-H column (10% isopropanol in hexanes, 1.0 mL/min, λ = 254 nm), t_R = 13.8 min (major), 26.8 min (minor), 94% *ee*.

(R,E)-N-(3-methylpent-3-en-2-yl)benzamide

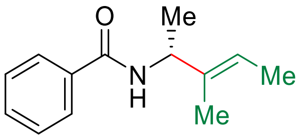

22

The product **22** was purified with silica gel chromatography (PE/EA = 10:1) as a colorless oil (40%). ^{1}H NMR (400 MHz, Chloroform-*d*) δ 7.81 – 7.71 (m, 2H), 7.52 – 7.43 (m, 1H), 7.45 – 7.36 (m, 2H), 6.11 (d, *J* = 8.0 Hz, 1H), 5.57 – 5.46 (m, 1H), 4.69 – 4.58 (m, 1H), 1.66 (s, 3H), 1.64 – 1.58 (m, 3H),

1.31 (d, J = 6.8 Hz, 3H). ^{13}C NMR (126 MHz, Chloroform-d) δ 166.74, 136.27, 134.96, 131.42, 128.62, 126.97, 119.53, 51.59, 19.76, 13.47, 13.37. HRMS (ESI): m/z calcd. for $C_{13}H_{17}NOH^+$ [M + H$^+$]: 204.1383, found: 204.1385. $[\alpha]_D{}^{20}$ = 9.3 (c = 1.0, $CHCl_3$), HPLC chiralcel AD-H column (10% isopropanol in hexanes, 1.0 mL/min, λ = 225 nm), t_R = 7.0 min (major), 8.4 min (minor), 93% *ee*.

(R,E)-N-(3-ethylhex-3-en-2-yl)benzamide

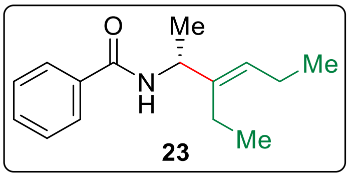

The product **23** was purified with silica gel chromatography (PE/EA = 10:1) as a colorless oil (39%). ^{1}H NMR (500 MHz, Chloroform-d) δ 7.81 – 7.69 (m, 2H), 7.51 – 7.45 (m, 1H), 7.45 – 7.38 (m, 2H), 6.04 (d, J = 8.5 Hz, 1H), 5.38 (t, J = 7.1 Hz, 1H), 4.76 – 4.67 (m, 1H), 2.17 – 2.00 (m, 4H), 1.33 (d, J = 6.8 Hz, 3H), 1.03 (t, J = 7.6 Hz, 3H), 0.98 (t, J = 7.5 Hz, 3H). ^{13}C NMR (126 MHz, Chloroform-d) δ 166.67, 141.47, 134.99, 131.42, 128.65, 127.01, 126.95, 49.20, 22.25, 20.89, 20.08, 14.54, 14.11. HRMS (ESI): m/z calcd. for $C_{15}H_{21}NONa^+$ [M + Na$^+$]: 254.1515, found: 254.1516. $[\alpha]_D{}^{20}$ = 4.5 (c = 1.0, $CHCl_3$), HPLC chiralcel AD-H column (5% isopropanol in hexanes, 1.0 mL/min, λ = 225 nm), t_R = 8.2 min (major), 10.0 min (minor), 95% *ee*.

(R,E)-N-(3-propylhept-3-en-2-yl)benzamide

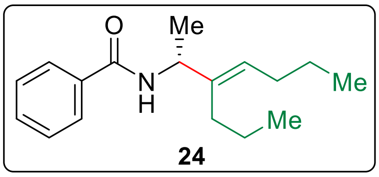

The product **24** was purified with silica gel chromatography (PE/EA = 10:1) as a colorless oil (34%). ^{1}H NMR (500 MHz, Chloroform-d) δ 7.79 – 7.70 (m, 2H), 7.52 – 7.46 (m, 1H), 7.46 – 7.38 (m, 2H), 5.99 (d, J = 8.4 Hz, 1H), 5.43 (t, J = 7.2 Hz, 1H), 4.73 – 4.64 (m, 1H), 2.11 – 1.97 (m, 4H), 1.52 – 1.41 (m, 2H), 1.42 – 1.35 (m, 2H), 1.33 (d, J = 6.8 Hz, 3H), 0.91 (2*t, J = 7.4 Hz, 6H). ^{13}C NMR (126 MHz, Chloroform-d) δ 166.68, 140.68, 135.05, 131.44, 128.68, 126.94, 125.81, 49.36, 31.57, 29.86, 23.11, 22.48, 20.20, 14.46, 14.05. HRMS (ESI): m/z calcd. for $C_{17}H_{25}NOH^+$ [M + H$^+$]: 260.2009, found: 260.2019. $[\alpha]_D{}^{20}$ = 30.8 (c = 1.0, $CHCl_3$), HPLC chiralcel AD-H column (10% isopropanol in hexanes, 1.0 mL/min, λ = 225 nm), t_R = 5.0 min (major), 5.8 min (minor), 95% *ee*.

(R,E)-N-(3-heptylundec-3-en-2-yl)benzamide

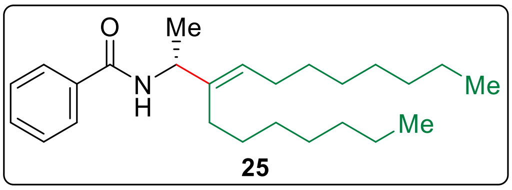

The product **25** was purified with silica gel chromatography (PE/EA = 10:1) as a colorless oil (31%). ^{1}H NMR (500 MHz, Chloroform-*d*) δ 7.80 – 7.70 (m, 2H), 7.52 – 7.46 (m, 1H), 7.46 – 7.39 (m, 2H), 5.98 (d, *J* = 8.4 Hz, 1H), 5.41 (t, *J* = 7.1 Hz, 1H), 4.73 – 4.64 (m, 1H), 2.14 – 1.97 (m, 4H), 1.47 – 1.21 (m, 23H), 0.91 – 0.83 (m, 6H). ^{13}C NMR (126 MHz, Chloroform-*d*) δ 166.67, 140.66, 135.06, 131.43, 128.67, 126.94, 125.87, 49.42, 31.97, 30.03, 29.98, 29.53, 29.52, 29.35, 29.30, 27.81, 22.80, 20.19, 14.26, 14.25. HRMS (ESI): m/z calcd. for $C_{25}H_{41}NOH^+$ [M + H$^+$]: 372.3261, found: 372.3269. $[\alpha]_D^{20}$ = 4.1 (c = 1.0, $CHCl_3$), HPLC chiralcel AD-H column (5% isopropanol in hexanes, 1.0 mL/min, λ = 245 nm), t_R = 4.9 min (major), 5.7 min (minor), 93% *ee*.

(R,E)-N-(3,5-dimethylhex-3-en-2-yl)benzamide

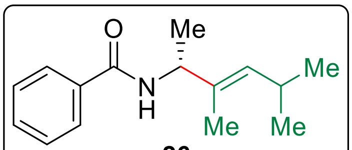

26

The product **26** was purified with silica gel chromatography (PE/EA = 10:1) as a colorless oil (35%, 4 : 1 r.r.). ^{1}H NMR (500 MHz, Chloroform-*d*) δ 7.79 – 7.71 (m, 2H), 7.52 – 7.46 (m, 1H), 7.45 – 7.39 (m, 2H), 5.99 (d, *J* = 8.2 Hz, 1H), 5.55 (q, *J* = 6.9 Hz, 0.2H), 5.27 – 5.22 (m, 0.8H), 4.77 – 4.69 (m, 0.2H), 4.67 – 4.58 (m, 0.8H), 2.92 – 2.82 (m, 0.2H), 2.59 – 2.47 (m, 0.8H), 1.72 – 1.64 (m, 3H), 1.39 – 1.28 (m, 3H), 1.10 – 1.04 (m, 1H), 1.00 – 0.84 (m, 5H). ^{13}C NMR (126 MHz, Chloroform-*d*) δ 166.75, 166.19, 147.69, 135.14, 135.08, 133.29, 133.15, 131.45, 131.40, 128.69, 128.67, 126.97, 126.92, 118.97, 51.35, 45.57, 28.74, 27.06, 23.08, 23.04, 21.65, 21.02, 21.00, 19.85, 13.97, 13.11. HRMS (ESI): m/z calcd. for $C_{15}H_{21}NOH^+$ [M + H$^+$]: 232.1696, found: 232.1696. $[\alpha]_D^{20}$ = 28.4 (c = 1.0, $CHCl_3$), HPLC chiralcel OD-H column (5% isopropanol in hexanes, 1.0 mL/min, λ = 225 nm), t_R = 7.1 min (major), 8.1 min (major), 13.4 min (minor), 15.2 min (minor), 96% *ee*.

(R,E)-N-(2,4,6-trimethylhept-4-en-3-yl)benzamide

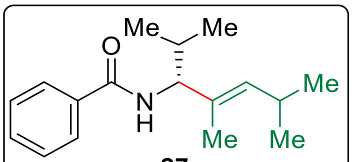

27

The product **27** was purified with silica gel chromatography (PE/EA = 10:1) as a colorless oil (25%, 10 : 1 r.r.). ^{1}H NMR (400 MHz, Chloroform-*d*) δ 7.81 – 7.70 (m, 2H), 7.53 – 7.47 (m, 1H), 7.47 – 7.40 (m, 2H), 6.07 (d, *J* = 9.4 Hz, 1H), 5.47 (q, *J* = 6.8 Hz, 0.09H), 5.26 – 5.19 (m, 0.9H), 4.38 – 4.30 (m, 0.09H), 4.31 – 4.22 (m, 0.9H), 2.87 – 2.76 (m, 0.09H), 2.61 – 2.44 (m, 0.9H), 1.98 – 1.81 (m, 1H), 1.64 (d, *J* = 1.4 Hz, 3H), 1.13 – 0.98 (m, 1H), 0.98 – 0.90 (m, 11H). ^{13}C NMR (101 MHz, Chloroform-*d*) δ 166.91, 135.40, 135.26, 131.41, 130.88, 128.73, 126.96, 62.16, 30.54, 27.10, 23.05, 20.24, 18.66, 13.34. HRMS (ESI): m/z calcd. for $C_{17}H_{25}NONa^+$ [M + Na$^+$]: 282.1828, found:

282.1838. $[\alpha]_D{}^{20}$ = -8.8 (c = 1.0, $CHCl_3$), HPLC chiralcel OD-H column (5% isopropanol in hexanes, 1.0 mL/min, λ = 245 nm), t_R = 5.9 min (major), 7.1 min (minor), 97% *ee*.

(R,E)-N-(2-methyl-1-phenylpent-1-en-3-yl)benzamide

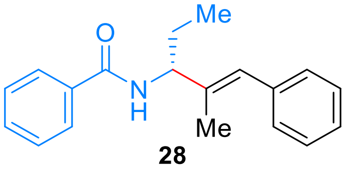

28

The product **28** was purified with silica gel chromatography (PE/EA = 10:1) as a white solid (65%). ^{1}H NMR (400 MHz, Chloroform-*d*) δ 7.86 – 7.75 (m, 2H), 7.54 – 7.48 (m, 1H), 7.48 – 7.41 (m, 2H), 7.35 – 7.29 (m, 2H), 7.28 – 7.25 (m, 2H), 7.24 – 7.17 (m, 1H), 6.55 (s, 1H), 6.20 (d, J = 8.5 Hz, 1H), 4.68 – 4.58 (m, 1H), 1.90 (d, J = 1.4 Hz, 3H), 1.87 – 1.71 (m, 2H), 1.01 (t, J = 7.4 Hz, 3H). ^{13}C NMR (101 MHz, Chloroform-*d*) δ 167.08, 137.68, 137.23, 134.97, 131.58, 129.15, 128.74, 128.19, 127.01, 126.63, 126.56, 58.39, 26.72, 15.02, 10.89. HRMS (ESI): m/z calcd. for $C_{19}H_{21}NONa^+$ [M + Na^+]: 302.1515, found: 302.1519. $[\alpha]_D{}^{20}$ = 0.4 (c = 1.0, $CHCl_3$), HPLC chiralcel AD-H column (10% isopropanol in hexanes, 1.0 mL/min, λ = 254 nm), t_R = 9.0 min (major), 11.9 min (minor), 95% *ee*.

(R,E)-N-(2,4-dimethyl-1-phenylpent-1-en-3-yl)benzamide

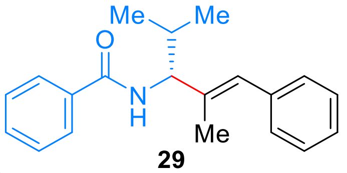

29

The product **29** was purified with silica gel chromatography (PE/EA = 10:1) as a white solid (53%). ^{1}H NMR (400 MHz, Chloroform-*d*) δ 7.84 – 7.76 (m, 2H), 7.56 – 7.47 (m, 1H), 7.49 – 7.41 (m, 2H), 7.36 – 7.27 (m, 2H), 7.29 – 7.22 (m, 2H), 7.25 – 7.16 (m, 1H), 6.53 (s, 1H), 6.23 (s, 1H), 4.55 – 4.42 (m, 1H), 2.11 – 1.98 (m, 1H), 1.89 (d, J = 1.4 Hz, 3H), 1.07 – 0.97 (m, 6H). ^{13}C NMR (101 MHz, Chloroform-*d*) δ 167.11, 137.70, 136.72, 135.10, 131.57, 129.17, 128.78, 128.17, 127.38, 126.99, 126.52, 62.85, 30.66, 20.34, 18.73, 15.02. HRMS (ESI): m/z calcd. for $C_{20}H_{23}NONa^+$ [M + Na^+]: 316.1672, found: 316.1676. $[\alpha]_D{}^{20}$ = -11.9 (c = 1.0, $CHCl_3$), HPLC chiralcel AD-H column (10% isopropanol in hexanes, 1.0 mL/min, λ = 254 nm), t_R = 9.1 min (major), 9.9 min (minor), 90% *ee*.

(R,E)-N-(2-methyl-1-phenylhex-1-en-3-yl)benzamide

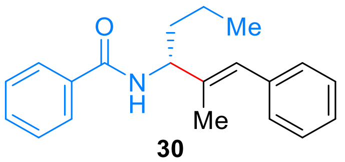

30

The product **30** was purified with silica gel chromatography (PE/EA = 10:1) as a white solid (59%). ^{1}H NMR (400 MHz, Chloroform-*d*) δ 7.84 – 7.76 (m, 2H), 7.54 – 7.47 (m, 1H), 7.47 – 7.41 (m, 2H), 7.35 – 7.29 (m, 2H), 7.28 – 7.24 (m, 2H), 7.24 – 7.17 (m, 1H), 6.55 (s, 1H), 6.23 (d, *J* = 8.4 Hz, 1H), 4.77 – 4.67 (m, 1H), 1.90 (d, *J* = 1.3 Hz, 3H), 1.83 – 1.61 (m, 2H), 1.52 – 1.36 (m, 2H), 0.98 (t, *J* = 7.3 Hz, 3H). ^{13}C NMR (101 MHz, Chloroform-*d*) δ 167.00, 137.69, 137.58, 134.95, 131.56, 129.14, 128.72, 128.17, 127.01, 126.52, 126.41, 56.75, 35.96, 19.67, 14.99, 14.09. HRMS (ESI): m/z calcd. for $C_{20}H_{23}NONa^{+}$ [M + Na^{+}]: 316.1672, found: 316.1678. $[\alpha]_D^{20}$ = -2.6 (c = 1.0, $CHCl_3$), HPLC chiralcel AD-H column (10% isopropanol in hexanes, 1.0 mL/min, λ = 254 nm), t_R = 9.2 min (minor), 10.5 min (major), 95% *ee*.

(R,E)-N-(2-methyl-1-phenylhept-1-en-3-yl)benzamide

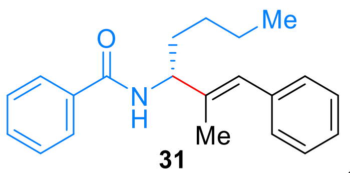

31

The product **31** was purified with silica gel chromatography (PE/EA = 10:1) as a white solid (60%). ^{1}H NMR (400 MHz, Chloroform-*d*) δ 7.87 – 7.76 (m, 2H), 7.54 – 7.47 (m, 1H), 7.47 – 7.40 (m, 2H), 7.36 – 7.29 (m, 2H), 7.29 – 7.24 (m, 2H), 7.24 – 7.18 (m, 1H), 6.55 (s, 1H), 6.37 – 6.09 (m, 1H), 4.75 – 4.65 (m, 1H), 1.91 (d, *J* = 1.4 Hz, 3H), 1.85 – 1.64 (m, 2H), 1.45 – 1.33 (m, 4H), 0.97 – 0.88 (m, 3H). ^{13}C NMR (101 MHz, Chloroform-*d*) δ 166.95, 137.70, 137.58, 134.97, 131.55, 129.14, 128.72, 128.17, 127.02, 126.52, 126.43, 56.97, 33.50, 28.55, 22.68, 15.00, 14.17. HRMS (ESI): m/z calcd. for $C_{21}H_{25}NOH^{+}$ [M + H^{+}]: 308.2009, found: 308.2012. $[\alpha]_D^{20}$ = -3.3 (c = 1.0, $CHCl_3$), HPLC chiralcel AD-H column (10% isopropanol in hexanes, 1.0 mL/min, λ = 245 nm), t_R = 9.5 min (major), 10.2 min (minor), 95% *ee*.

(R,E)-N-(2,5-dimethyl-1-phenylhex-1-en-3-yl)benzamide

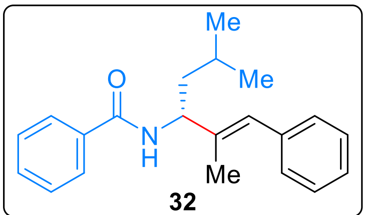

32

The product **32** was purified with silica gel chromatography (PE/EA = 10:1) as a white solid (48%). ^{1}H NMR (500 MHz, Chloroform-*d*) δ 7.82 – 7.75 (m, 2H), 7.54 – 7.47 (m, 1H), 7.47 – 7.41 (m, 2H), 7.34 – 7.29 (m, 2H), 7.28 – 7.24 (m, 2H), 7.23 – 7.17 (m, 1H), 6.56 (s, 1H), 6.13 (d, *J* = 8.5 Hz, 1H), 4.84 – 4.75 (m, 1H), 1.90 (d, *J* = 1.4 Hz, 3H), 1.76 – 1.67 (m, 1H), 1.64 – 1.54 (m, 2H), 1.00 (2*d, *J* = 2.2 Hz, 6H). ^{13}C NMR (126 MHz, Chloroform-*d*) δ 166.88, 137.84, 137.72, 134.96, 131.58, 129.16, 128.75, 128.19, 127.01, 126.53, 126.35, 55.25, 43.16, 25.35, 23.05, 22.64, 14.93. HRMS (ESI): m/z calcd. for $C_{21}H_{25}NONa^{+}$ [M + Na^{+}]: 330.1828, found: 330.1832. $[\alpha]_D^{20}$ = -4.0 (c = 1.0,

$CHCl_3$), HPLC chiralcel OD-H column (10% isopropanol in hexanes, 1.0 mL/min, λ = 245 nm), t_R = 9.2 min (major), 17.4 min (minor), 96% *ee*.

(R,E)-N-(3-methyl-1,4-diphenylbut-3-en-2-yl)benzamide

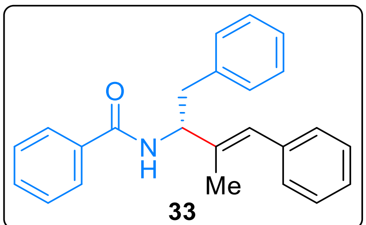

33

The product **33** was purified with silica gel chromatography (PE/EA = 10:1) as a white solid (64%). ^{1}H NMR (500 MHz, Chloroform-*d*) δ 7.72 – 7.67 (m, 2H), 7.52 – 7.45 (m, 1H), 7.44 – 7.38 (m, 2H), 7.35 – 7.28 (m, 4H), 7.27 – 7.23 (m, 3H), 7.23 – 7.16 (m, 3H), 6.37 (s, 1H), 6.31 (d, *J* = 8.1 Hz, 1H), 5.04 – 4.96 (m, 1H), 3.17 – 3.03 (m, 2H), 1.96 (d, *J* = 1.4 Hz, 3H). ^{13}C NMR (126 MHz, Chloroform-*d*) δ 166.88, 137.57, 137.46, 136.78, 134.81, 131.57, 129.44, 129.05, 128.71, 128.63, 128.15, 126.95, 126.87, 126.68, 126.54, 57.41, 39.85, 15.76. HRMS (ESI): m/z calcd. for $C_{24}H_{23}NONa^+$ [M + Na^+]: 364.1672, found: 364.1676. $[\alpha]_D^{20}$ = -1.5 (c = 1.0, $CHCl_3$), HPLC chiralcel AD-H column (10% isopropanol in hexanes, 1.0 mL/min, λ = 245 nm), t_R = 14.8 min (major), 25.4 min (minor), 94% *ee*.

(R,E)-N-(2-methyl-1,5-diphenylpent-1-en-3-yl)benzamide

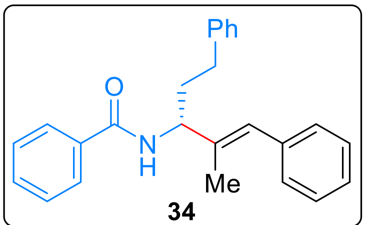

34

The product **34** was purified with silica gel chromatography (PE/EA = 10:1) as a white solid (37%). ^{1}H NMR (500 MHz, Chloroform-*d*) δ 7.75 – 7.67 (m, 2H), 7.54 – 7.48 (m, 1H), 7.46 – 7.40 (m, 2H), 7.36 – 7.26 (m, 6H), 7.25 – 7.18 (m, 4H), 6.55 (s, 1H), 6.20 (d, *J* = 8.5 Hz, 1H), 4.85 – 4.74 (m, 1H), 2.77 (t, *J* = 7.8 Hz, 2H), 2.19 – 2.05 (m, 2H), 1.93 (d, *J* = 1.4 Hz, 3H). ^{13}C NMR (126 MHz, Chloroform-*d*) δ 166.88, 141.65, 137.56, 137.11, 134.75, 131.62, 129.16, 128.73, 128.71, 128.58, 128.23, 127.00, 126.80, 126.65, 126.22, 56.83, 35.27, 32.74, 15.21. HRMS (ESI): m/z calcd. for $C_{25}H_{25}NONa^+$ [M + Na^+]: 378.1828, found: 378.1839. $[\alpha]_D^{20}$ = 0.9 (c = 1.0, $CHCl_3$), HPLC chiralcel AD-H column (10% isopropanol in hexanes, 1.0 mL/min, λ = 245 nm), t_R = 12.7 min (major), 18.1 min (minor), 94% *ee*.

(R,E)-N-(1-cyclopentyl-2-methyl-3-phenylallyl)benzamide

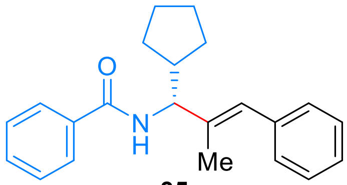

35

The product **35** was purified with silica gel chromatography (PE/EA = 10:1) as a white solid (68%). ^{1}H NMR (500 MHz, Chloroform-*d*) δ 7.85 – 7.72 (m, 2H), 7.53 – 7.47 (m, 1H), 7.50 – 7.40 (m, 2H), 7.34 – 7.29 (m, 2H), 7.29 – 7.26 (m, 2H), 7.23 – 7.17 (m, 1H), 6.58 (s, 1H), 6.34 – 6.21 (m, 1H), 4.55 – 4.48 (m, 1H), 2.31 – 2.19 (m, 1H), 1.89 (d, *J* = 1.4 Hz, 3H), 1.87 – 1.77 (m, 1H), 1.76 – 1.66 (m, 3H), 1.63 – 1.52 (m, 2H), 1.50 – 1.34 (m, 2H). ^{13}C NMR (126 MHz, Chloroform-*d*) δ 166.98, 137.74, 137.18, 135.11, 131.51, 129.18, 128.72, 128.15, 127.63, 127.01, 126.49, 62.15, 42.83, 30.27, 29.99, 25.57, 25.52, 14.58. HRMS (ESI): m/z calcd. for $C_{22}H_{25}NONa^{+}$ [M + Na^{+}]: 342.1828, found: 342.1822. $[\alpha]_D^{20}$ = -11.8 (c = 1.0, $CHCl_3$), HPLC chiralcel AD-H column (10% isopropanol in hexanes, 1.0 mL/min, λ = 245 nm), t_R = 11.0 min (minor), 13.6 min (major), 87% *ee*.

(R,E)-N-(1-cyclohexyl-2-methyl-3-phenylallyl)benzamide

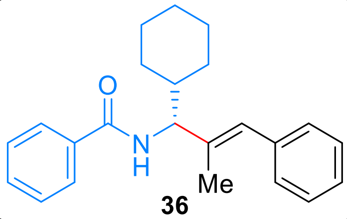

36

The product **36** was purified with silica gel chromatography (PE/EA = 10:1) as a white solid (45%). ^{1}H NMR (500 MHz, Chloroform-*d*) δ 7.85 – 7.75 (m, 2H), 7.54 – 7.48 (m, 1H), 7.48 – 7.42 (m, 2H), 7.34 – 7.29 (m, 2H), 7.28 – 7.25 (m, 2H), 7.23 – 7.17 (m, 1H), 6.53 (s, 1H), 6.22 (d, *J* = 9.1 Hz, 1H), 4.55 – 4.47 (m, 1H), 1.93 – 1.89 (m, 1H), 1.88 (d, *J* = 1.4 Hz, 3H), 1.84 – 1.72 (m, 3H), 1.70 – 1.65 (m, 1H), 1.32 – 1.15 (m, 4H), 1.14 – 1.03 (m, 2H). ^{13}C NMR (126 MHz, Chloroform-*d*) δ 167.05, 137.70, 136.25, 135.13, 131.54, 129.18, 128.76, 128.17, 127.78, 127.00, 126.52, 62.16, 40.29, 30.67, 29.46, 26.45, 26.29, 26.27, 14.84. HRMS (ESI): m/z calcd. for $C_{23}H_{27}NONa^{+}$ [M + Na^{+}]: 356.1985, found: 356.1990. $[\alpha]_D^{20}$ = -9.4 (c = 1.0, $CHCl_3$), HPLC chiralcel AD-H column (10% isopropanol in hexanes, 1.0 mL/min, λ = 245 nm), t_R = 10.0 min (minor), 13.8 min (major), 93% *ee*.

(R,E)-N-(2-methyl-5-(methylthio)-1-phenylpent-1-en-3-yl)benzamide

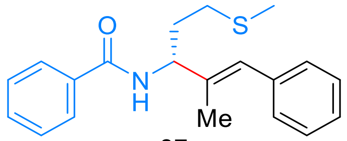

37

The product **37** was purified with silica gel chromatography (PE/EA = 10:1) as a white solid (47%).

^{1}H NMR (500 MHz, Chloroform-*d*) δ 7.85 – 7.79 (m, 2H), 7.54 – 7.49 (m, 1H), 7.48 – 7.42 (m, 2H), 7.35 – 7.30 (m, 2H), 7.28 – 7.26 (m, 1H), 7.25 – 7.18 (m, 2H), 6.62 (d, *J* = 8.4 Hz, 1H), 6.57 (s, 1H), 4.89 – 4.81 (m, 1H), 2.62 (t, *J* = 7.4 Hz, 2H), 2.15 (s, 3H), 2.13 – 2.01 (m, 2H), 1.92 (d, *J* = 1.5 Hz, 3H). ^{13}C NMR (126 MHz, Chloroform-*d*) δ 166.94, 137.41, 136.65, 134.62, 131.71, 129.14, 128.78, 128.24, 127.06, 126.85, 126.71, 56.40, 32.66, 30.95, 15.83, 15.35. HRMS (ESI): m/z calcd. for $C_{20}H_{23}NOSNa^{+}$ [M + Na^{+}]: 348.1393, found: 348.1404. $[\alpha]_D^{20}$ = 0.9 (c = 1.0, $CHCl_3$), HPLC chiralcel AD-H column (10% isopropanol in hexanes, 1.0 mL/min, λ = 245 nm), t_R = 13.7 min (major), 18.0 min (minor), 96% *ee*.

(R,E)-N-(3-methyl-4-phenylbut-3-en-2-yl)-2-naphthamide

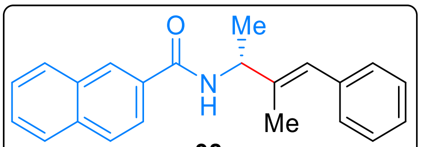

The product **38** was purified with silica gel chromatography (PE/EA = 10:1) as a white solid (54%). ^{1}H NMR (400 MHz, Chloroform-*d*) δ 8.32 (d, *J* = 1.5 Hz, 1H), 7.96 – 7.84 (m, 4H), 7.61 – 7.49 (m, 2H), 7.38 – 7.31 (m, 2H), 7.30 – 7.26 (m, 2H), 7.25 – 7.19 (m, 1H), 6.59 (s, 1H), 6.51 – 6.37 (m, 1H), 4.96 – 4.85 (m, 1H), 1.97 (d, *J* = 1.4 Hz, 3H), 1.50 (d, *J* = 6.9 Hz, 3H). ^{13}C NMR (126 MHz, Chloroform-*d*) δ 166.92, 138.93, 137.67, 134.82, 132.72, 132.04, 129.13, 129.00, 128.60, 128.21, 127.87, 127.74, 127.41, 126.88, 126.57, 125.24, 123.74, 52.21, 20.01, 15.61. HRMS (ESI): m/z calcd. for $C_{22}H_{21}NONa^{+}$ [M + Na^{+}]: 338.1515, found: 338.1524. $[\alpha]_D^{20}$ = -17.9 (c = 1.0, $CHCl_3$), HPLC chiralcel AD-H column (10% isopropanol in hexanes, 1.0 mL/min, λ = 230 nm), t_R = 14.7 min (major), 36.2 min (minor), 93% *ee*.

(R,E)-N-(3-methyl-4-phenylbut-3-en-2-yl)thiophene-2-carboxamide

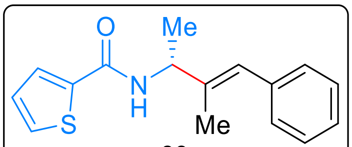

The product **39** was purified with silica gel chromatography (PE/EA = 10:1) as a white solid (67%). ^{1}H NMR (400 MHz, Chloroform-*d*) δ 7.54 (dd, *J* = 3.7, 1.2 Hz, 1H), 7.47 (dd, *J* = 5.0, 1.2 Hz, 1H), 7.36 – 7.28 (m, 2H), 7.27 – 7.15 (m, 3H), 7.07 (dd, *J* = 5.0, 3.7 Hz, 1H), 6.53 (s, 1H), 6.11 (s, 1H), 4.86 – 4.74 (m, 1H), 1.92 (d, *J* = 1.3 Hz, 3H), 1.45 (d, *J* = 6.8 Hz, 3H). ^{13}C NMR (101 MHz, Chloroform-*d*) δ 161.26, 139.22, 138.72, 137.61, 130.03, 129.11, 128.20, 128.08, 127.74, 126.59, 125.32, 52.12, 19.93, 15.52. HRMS (ESI): m/z calcd. for $C_{16}H_{17}NOSH^{+}$ [M + H^{+}]: 272.1109, found: 272.1100. $[\alpha]_D^{20}$ = 0.9 (c = 1.0, $CHCl_3$), HPLC chiralcel AD-H column (10% isopropanol in hexanes, 1.0 mL/min, λ = 245 nm), t_R = 8.8 min (major), 11.4 min (minor), 96% *ee*.

(R,E)-N-(3-methyl-4-phenylbut-3-en-2-yl)pivalamide

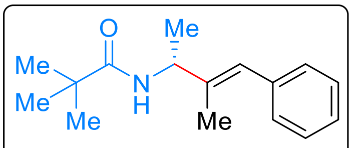

40

The product **40** was purified with silica gel chromatography (PE/EA = 10:1) as a white solid (30%). ^{1}H NMR (400 MHz, Chloroform-*d*) δ 7.36 – 7.28 (m, 2H), 7.26 – 7.17 (m, 3H), 6.42 (s, 1H), 5.63 (d, J = 8.2 Hz, 1H), 4.65 – 4.54 (m, 1H), 1.85 (d, J = 1.3 Hz, 3H), 1.33 (d, J = 6.9 Hz, 3H), 1.22 (s, 9H). ^{13}C NMR (101 MHz, Chloroform-*d*) δ 177.70, 139.28, 137.78, 129.10, 128.20, 126.50, 124.58, 51.26, 38.83, 27.75, 19.90, 15.53. HRMS (ESI): m/z calcd. for $C_{16}H_{23}NONa^{+}$ [M + Na^{+}]: 268.1672, found: 268.1677. $[\alpha]_D^{20}$ = 63.9 (c = 1.0, $CHCl_3$), HPLC chiralcel AD-H column (10% isopropanol in hexanes, 1.0 mL/min, λ = 254 nm), t_R = 4.5 min (major), 5.1 min (minor), 95% *ee*.

(R,E)-2-(3-methyl-4-phenylbut-3-en-2-yl)isoindoline-1,3-dione

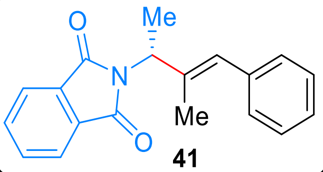

41

The product **41** was purified with silica gel chromatography (PE/EA = 20:1) as a white solid (57%). ^{1}H NMR (600 MHz, Chloroform-*d*) δ 7.84 (dd, J = 5.4, 3.1 Hz, 2H), 7.71 (dd, J = 5.5, 3.0 Hz, 2H), 7.34 – 7.28 (m, 2H), 7.27 – 7.23 (m, 2H), 7.22 – 7.17 (m, 1H), 6.60 (s, 1H), 5.00 (q, J = 7.2 Hz, 1H), 1.87 (s, 3H), 1.75 (d, J = 7.2 Hz, 3H). ^{13}C NMR (151 MHz, Chloroform-*d*) δ 168.50, 137.68, 136.14, 134.06, 132.08, 129.19, 128.16, 126.92, 126.60, 123.32, 52.75, 16.57, 16.10. HRMS (ESI): m/z calcd. for $C_{19}H_{17}NO_2Na^{+}$ [M + Na^{+}]: 314.1151, found: 314.1151. $[\alpha]_D^{20}$ = 8.0 (c = 1.0, $CHCl_3$), HPLC chiralcel IC column (5% isopropanol in hexanes, 0.5 mL/min, λ = 245 nm), t_R = 14.0 min (minor), 14.7 min (major), 49% *ee*.

(R)-5-benzamido-4-((E)-benzylidene)hexyl (S)-2-(2-fluoro-[1,1'-biphenyl]-4-yl)propanoate

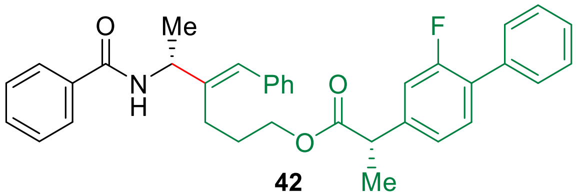

42

The product **42** was purified with silica gel chromatography (PE/EA = 5:1) as a white solid (54%). ^{1}H NMR (400 MHz, Chloroform-*d*) δ 7.82 – 7.76 (m, 2H), 7.54 – 7.48 (m, 3H), 7.47 – 7.39 (m, 4H), 7.39 – 7.29 (m, 4H), 7.27 – 7.18 (m, 3H), 7.12 – 7.01 (m, 2H), 6.57 (s, 1H), 6.16 (d, J = 7.8 Hz, 1H), 4.93 – 4.81 (m, 1H), 4.16 – 3.99 (m, 2H), 3.68 – 3.59 (m, 1H), 2.43 – 2.26 (m, 2H), 1.95 – 1.83 (m, 2H), 1.49 – 1.41 (m, 6H). ^{19}F NMR (376 MHz, Chloroform-*d*) δ -117.66 (d, J = 8.8 Hz). ^{13}C

NMR (101 MHz, Chloroform-*d*) δ 174.04, 166.78, 159.74 (d, J = 248.3 Hz), 143.02, 141.97 (d, J = 7.8 Hz), 137.43, 135.61, 134.67 (d, J = 2.9 Hz), 131.70, 130.88 (d, J = 4.0 Hz), 129.06 (d, J = 2.9 Hz), 128.78, 128.71, 128.55, 128.43, 127.75, 127.00, 126.87, 125.75, 123.68, 123.68 (d, J = 5.6 Hz), 115.35 (d, J = 23.6 Hz), 64.87, 49.03, 45.06, 27.65, 26.22, 20.28, 18.41. HRMS (ESI): m/z calcd. for $C_{35}H_{34}FNO_3H^+$ [M + H$^+$]: 536.2595, found: 536.2595. $[\alpha]_D^{20}$ = 5.4 (c = 1.0, $CHCl_3$), HPLC chiralcel AD-H column (10% isopropanol in hexanes, 1.0 mL/min, λ = 245 nm), t_R = 13.8 min (major), 23.6 min (minor), 95% *de*.

(R)-5-benzamido-4-((E)-benzylidene)hexyl (S)-2-(6-methoxynaphthalen-2-yl)propanoate

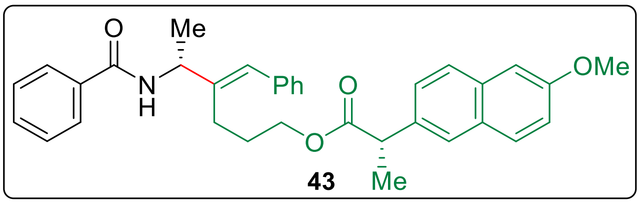

43

The product **43** was purified with silica gel chromatography (PE/EA = 5:1) as a white solid (60%). ^{1}H NMR (400 MHz, Chloroform-*d*) δ 7.81 – 7.75 (m, 2H), 7.70 – 7.61 (m, 3H), 7.53 – 7.48 (m, 1H), 7.47 – 7.40 (m, 2H), 7.37 – 7.28 (m, 3H), 7.25 – 7.17 (m, 3H), 7.14 – 7.07 (m, 2H), 6.54 (s, 1H), 6.13 (d, J = 8.3 Hz, 1H), 4.88 – 4.77 (m, 1H), 4.13 – 4.05 (m, 1H), 4.05 – 3.97 (m, 1H), 3.90 (s, 3H), 3.76 (q, J = 7.1 Hz, 1H), 2.39 – 2.25 (m, 2H), 1.93 – 1.78 (m, 2H), 1.51 (d, J = 7.1 Hz, 3H), 1.36 (d, J = 6.8 Hz, 3H). ^{13}C NMR (101 MHz, Chloroform-*d*) δ 174.76, 166.74, 157.65, 143.02, 137.38, 135.76, 134.64, 133.73, 131.65, 129.37, 128.97, 128.73, 128.66, 128.39, 127.20, 126.99, 126.80, 126.36, 126.03, 125.67, 119.04, 105.60, 64.65, 55.40, 49.16, 45.46, 27.68, 26.18, 20.17, 18.50. HRMS (ESI): m/z calcd. for $C_{34}H_{35}NO_4Na^+$ [M + Na$^+$]: 544.2458, found: 544.2463. $[\alpha]_D^{20}$ = 14.0 (c = 1.0, $CHCl_3$), HPLC chiralcel AD-H column (10% isopropanol in hexanes, 1.0 mL/min, λ = 245 nm), t_R = 21.7 min (major), 40.3 min (minor), 96% *de*.

(R)-5-benzamido-4-((E)-benzylidene)hexyl 2-(4-benzoylphenyl)propanoate

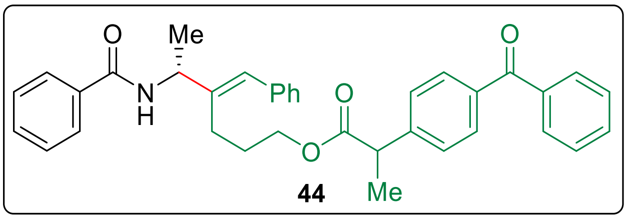

44

The product **44** was purified with silica gel chromatography (PE/EA = 5:1) as a white solid (45%, dr = 1 : 1). ^{1}H NMR (500 MHz, Chloroform-*d*) δ 7.82 – 7.74 (m, 4H), 7.72 – 7.67 (m, 1H), 7.66 – 7.61 (m, 1H), 7.60 – 7.55 (m, 1H), 7.51 – 7.35 (m, 7H), 7.32 – 7.26 (m, 2H), 7.23 – 7.16 (m, 3H), 6.57 (s, 1H), 6.38 – 6.25 (m, 1H), 4.89 – 4.83 (m, 1H), 4.12 – 4.04 (m, 1H), 4.04 – 3.97 (m, 1H), 3.71 – 3.62 (m, 1H), 2.39 – 2.25 (m, 2H), 1.94 – 1.79 (m, 2H), 1.56 – 1.41 (m, 6H). ^{13}C NMR (126 MHz, Chloroform-*d*) δ 196.69, 174.09, 166.79, 142.97, 140.98, 137.89, 137.53, 137.40, 134.65, 134.61, 132.64, 131.75, 131.64, 131.62, 130.17, 129.30, 129.21, 129.09, 128.71, 128.66, 128.60,

128.41, 128.38, 127.02, 126.80, 125.79, 64.82, 49.21, 49.12, 45.42, 45.40, 27.61, 27.60, 26.11, 26.06, 20.26, 20.22, 18.53, 18.48. HRMS (ESI): m/z calcd. for $C_{36}H_{35}NO_4Na^+$ [M + Na$^+$]: 568.2458, found: 568.2461. $[\alpha]_D^{20}$ = 9.3 (c = 1.0, $CHCl_3$), HPLC chiralcel AD-H column (5% isopropanol in hexanes, 0.5 mL/min, λ = 245 nm), t_R = 188.6 min (major), 197.5 min (major), 374.2 min (minor), 432.7 min (minor), 95% *ee*.

4-((R,E)-3-benzamido-2-methylbut-1-en-1-yl)benzyl (S)-2-(4-isobutylphenyl)propanoate

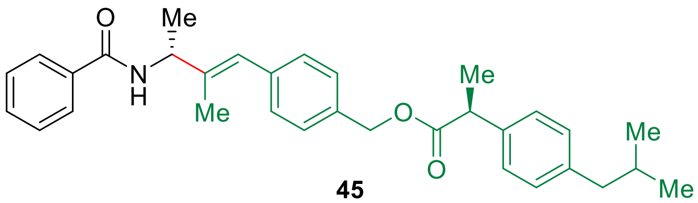

45

The product **45** was purified with silica gel chromatography (PE/EA = 5:1) as a white solid (73%). ^{1}H NMR (500 MHz, Chloroform-*d*) δ 7.86 – 7.74 (m, 2H), 7.55 – 7.48 (m, 1H), 7.47 – 7.41 (m, 2H), 7.24 – 7.14 (m, 6H), 7.11 – 7.06 (m, 2H), 6.51 (s, 1H), 6.27 – 6.12 (m, 1H), 5.16 – 5.02 (m, 2H), 4.88 – 4.79 (m, 1H), 3.75 (q, *J* = 7.2 Hz, 1H), 2.45 (d, *J* = 7.2 Hz, 2H), 1.91 (s, 3H), 1.90 – 1.78 (m, 1H), 1.51 (d, *J* = 7.2 Hz, 3H), 1.45 (d, *J* = 6.8 Hz, 3H), 0.89 (d, *J* = 6.6 Hz, 6H). ^{13}C NMR (101 MHz, Chloroform-*d*) δ 174.70, 166.86, 140.69, 139.29, 137.72, 137.48, 134.83, 134.31, 131.63, 129.44, 129.18, 128.75, 127.74, 127.35, 127.02, 124.78, 66.25, 52.10, 45.27, 45.14, 30.32, 22.51, 19.99, 18.55, 15.53. HRMS (ESI): m/z calcd. for $C_{32}H_{37}NO_3Na^+$ [M + Na$^+$]: 506.2666, found: 506.2675. $[\alpha]_D^{20}$ = 1.2 (c = 1.0, $CHCl_3$), HPLC chiralcel AD-H column (30% isopropanol in hexanes, 1.0 mL/min, λ = 245 nm), t_R = 4.8 min (major), 5.7 min (minor), 93% *de*.

(R,E)-4-(3-benzamido-2-methylbut-1-en-1-yl)phenyl 2-(11-oxo-6,11 dihydrodibenzo[b,e]oxepin-2-yl)acetate

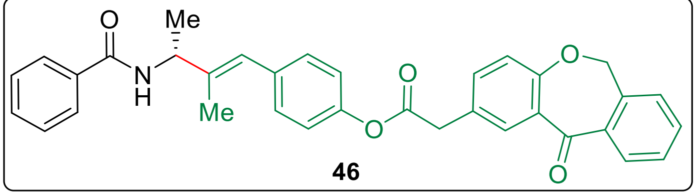

46

The product **46** was purified with silica gel chromatography (PE/EA = 5:1) as a white solid (65%). ^{1}H NMR (400 MHz, Chloroform-*d*) δ 8.22 (d, *J* = 2.4 Hz, 1H), 7.93 – 7.87 (m, 1H), 7.84 – 7.75 (m, 2H), 7.61 – 7.46 (m, 4H), 7.48 – 7.39 (m, 2H), 7.40 – 7.34 (m, 1H), 7.26 – 7.20 (m, 2H), 7.10 – 7.01 (m, 3H), 6.49 (s, 1H), 6.30 – 6.12 (m, 1H), 5.19 (s, 2H), 4.87 – 4.76 (m, 1H), 3.88 (s, 2H), 1.90 (s, 3H), 1.43 (d, *J* = 6.8 Hz, 3H). ^{13}C NMR (101 MHz, Chloroform-*d*) δ 191.01, 170.06, 166.86, 160.77, 149.16, 140.55, 139.26, 136.45, 135.62, 135.48, 134.79, 132.98, 132.74, 131.63, 130.10, 129.62, 129.44, 128.74, 127.98, 127.32, 127.02, 125.33, 124.31, 121.39, 121.19, 73.77, 52.06, 40.42, 19.97, 15.48. HRMS (ESI): m/z calcd. for $C_{35}H_{32}NO_5H^+$ [M + H$^+$]: 547.2353, found: 547.2334. $[\alpha]_D^{20}$ = -

0.5 (c = 1.0, $CHCl_3$), HPLC chiralcel AD-H column (30% isopropanol in hexanes, 1.0 mL/min, λ = 254 nm), t_R = 24.7 min (major), 33.2 min (minor), 91% *ee*.

(R,E)-4-(3-benzamido-2-methylbut-1-en-1-yl)benzyl 2-(4-(4-chlorobenzoyl)phenoxy)-2-methylpropanoate

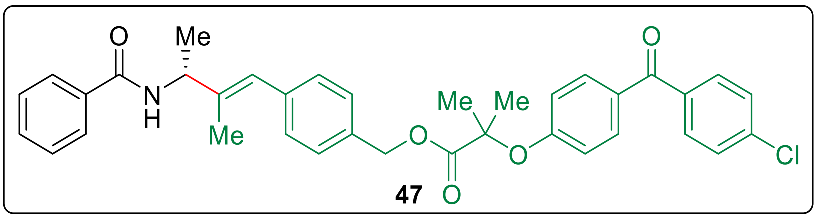

The product **47** was purified with silica gel chromatography (PE/EA = 5:1) as a white solid (62%). ^{1}H NMR (500 MHz, Chloroform-*d*) δ 7.86 – 7.76 (m, 2H), 7.67 – 7.63 (m, 2H), 7.63 – 7.58 (m, 2H), 7.52 – 7.47 (m, 1H), 7.47 – 7.39 (m, 4H), 7.24 – 7.16 (m, 4H), 6.78 – 6.70 (m, 2H), 6.49 (s, 1H), 6.28 (d, *J* = 8.1 Hz, 1H), 5.23 – 5.14 (m, 2H), 4.87 – 4.77 (m, 1H), 1.90 (d, *J* = 1.4 Hz, 3H), 1.67 (2*s, 6H), 1.43 (d, *J* = 6.8 Hz, 3H). ^{13}C NMR (126 MHz, Chloroform-*d*) δ 194.34, 173.57, 166.90, 159.67, 139.68, 138.51, 138.11, 136.50, 134.86, 133.25, 132.10, 131.59, 131.28, 130.30, 129.29, 128.71, 128.68, 128.59, 127.10, 124.62, 117.30, 79.55, 67.33, 52.10, 25.64, 25.47, 19.88, 15.72. HRMS (ESI): m/z calcd. for $C_{36}H_{34}ClNO_5Na^+$ [M + Na$^+$]: 618.2018, found: 618.2017. $[\alpha]_D^{20}$ = 2.7 (c = 1.0, $CHCl_3$), HPLC chiralcel AD-H column (30% isopropanol in hexanes, 1.0 mL/min, λ = 254 nm), t_R = 9.1 min (major), 10.8 min (minor), 93% *ee*.

4-((R,E)-3-benzamido-2-methylbut-1-en-1-yl)benzyl (4R)-4-((8R,9S,10S,13R,14S,17R)-10,13-dimethyl-3,7,12-trioxohexadecahydro-1H-cyclopenta[a]phenanthren-17-yl)pentanoate

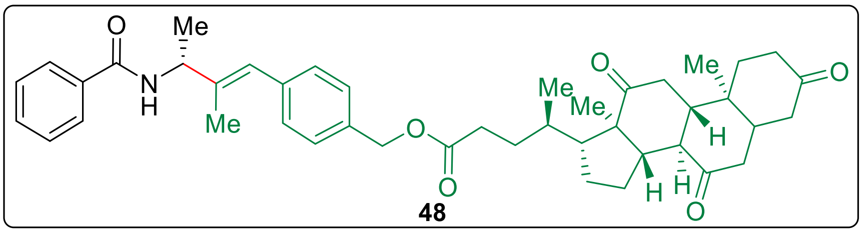

The product **48** was purified with silica gel chromatography (PE/EA = 5:1) as a white solid (57%). ^{1}H NMR (500 MHz, Chloroform-*d*) δ 7.81 – 7.76 (m, 2H), 7.53 – 7.47 (m, 1H), 7.47 – 7.40 (m, 2H), 7.32 – 7.27 (m, 2H), 7.26 – 7.21 (m, 2H), 6.51 (s, 1H), 6.29 – 6.16 (m, 1H), 5.14 – 5.03 (m, 2H), 4.87 – 4.78 (m, 1H), 3.00 (s, 1H), 2.93 (s, 1H), 2.92 – 2.78 (m, 3H), 2.48 – 2.38 (m, 1H), 2.37 – 2.16 (m, 8H), 2.15 – 2.07 (m, 3H), 2.03 – 1.97 (m, 2H), 1.92 (s, 3H), 1.87 – 1.79 (m, 2H), 1.65 – 1.54 (m, 1H), 1.45 (d, *J* = 6.9 Hz, 3H), 1.38 (s, 3H), 1.27 – 1.18 (m, 2H), 1.02 (d, *J* = 3.0 Hz, 3H), 0.82 (d, *J* = 6.7 Hz, 3H). ^{13}C NMR (126 MHz, Chloroform-*d*) δ 212.13, 209.29, 208.94, 174.01, 166.83, 139.40, 137.68, 134.79, 134.22, 131.62, 129.27, 128.72, 128.21, 127.02, 124.67, 66.03, 56.97, 52.09, 51.84, 49.06, 46.94, 45.71, 45.62, 45.08, 42.89, 38.73, 36.59, 36.10, 35.55, 35.35, 31.65, 30.53, 27.71, 25.22, 22.01, 19.98, 18.72, 15.52, 11.92. HRMS (ESI): m/z calcd. for

$C_{43}H_{53}NO_6H^+$ [M + H$^+$]: 680.3946, found: 680.3947. $[\alpha]_D{}^{20}$ = 11.4 (c = 1.0, $CHCl_3$), HPLC chiralcel AD-H column (30% isopropanol in hexanes, 1.0 mL/min, λ = 254 nm), t_R = 17.3 min (major), 22.4 min (minor), 90% *de*.

4-((R,E)-3-benzamido-2-methylbut-1-en-1-yl)benzyl ((S)-2,5,7,8-tetramethyl-2-((4S,8S)-4,8,12-trimethyltridecyl)chroman-6-yl) succinate

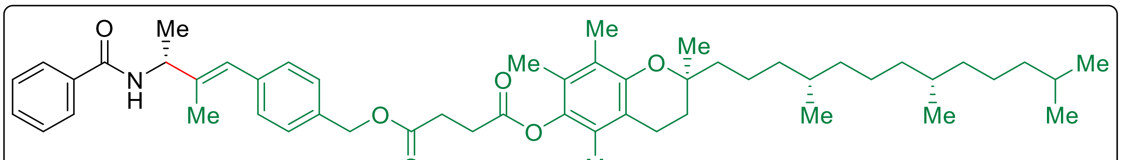

49

The product **49** was purified with silica gel chromatography (PE/EA = 5:1) as a white solid (75%). ^{1}H NMR (400 MHz, Chloroform-*d*) δ 7.84 – 7.76 (m, 2H), 7.54 – 7.48 (m, 1H), 7.48 – 7.41 (m, 2H), 7.35 – 7.29 (m, 2H), 7.26 – 7.22 (m, 2H), 6.52 (s, 1H), 6.20 (d, *J* = 8.1 Hz, 1H), 5.14 (s, 2H), 4.90 – 4.78 (m, 1H), 2.99 – 2.91 (m, 2H), 2.86 – 2.78 (m, 2H), 2.58 (t, *J* = 6.8 Hz, 2H), 2.08 (s, 3H), 2.00 (s, 3H), 1.96 (s, 3H), 1.92 (d, *J* = 1.3 Hz, 3H), 1.86 – 1.73 (m, 2H), 1.58 – 1.49 (m, 3H), 1.46 (d, *J* = 6.9 Hz, 3H), 1.42 – 1.20 (m, 15H), 1.17 – 1.03 (m, 6H), 0.89 – 0.83 (m, 12H). ^{13}C NMR (101 MHz, Chloroform-*d*) δ 172.17, 171.07, 166.84, 149.54, 140.53, 139.45, 137.79, 134.82, 133.90, 131.62, 129.31, 128.73, 128.21, 127.02, 126.80, 125.06, 124.72, 123.13, 117.49, 75.16, 66.57, 52.08, 39.48, 37.56, 37.54, 37.40, 32.91, 32.82, 29.34, 28.97, 28.10, 24.92, 24.56, 22.85, 22.76, 21.14, 20.70, 19.97, 19.88, 19.78, 15.54, 13.05, 12.20, 11.94. HRMS (ESI): m/z calcd. for $C_{52}H_{73}NO_6H^+$ [M + H$^+$]: 808.5511, found: 808.5524. $[\alpha]_D{}^{20}$ = 1.5 (c = 1.0, $CHCl_3$), HPLC chiralcel AD-H column (30% isopropanol in hexanes, 1.0 mL/min, λ = 254 nm), t_R = 4.6 min (major), 5.3 min (minor), 94% *de*.

Unsuccessful substrates

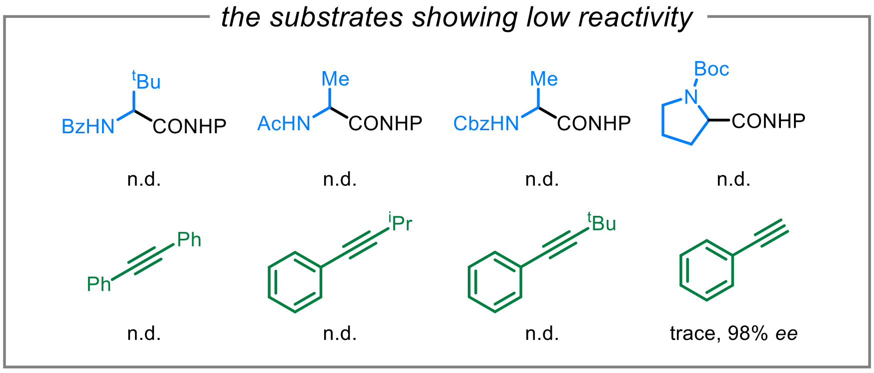

Supplementary Figure 0. The substrates showing low reactivity in the system.

Synthetic Applications

Gram scale reaction

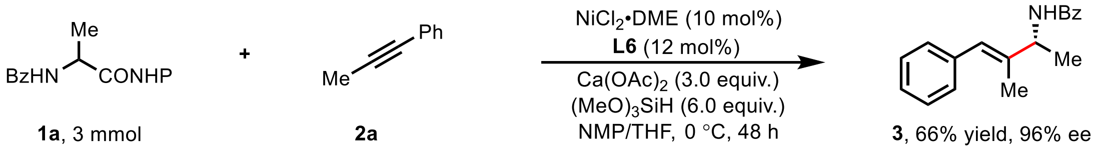

In glove box, $NiCl_2$•DME (0.3 mmol, 10 mol%), L6 (0.36 mmol, 12 mol%), $Ca(OAc)_2$ (9 mmol, 3.0 equiv.) and alkyl NHP ester **1a** (3 mmol, 1.0 equiv.) were combined in a 50 mL oven-dried sealing tube. The vessel was evacuated and backfilled with Ar (repeated for 3 times). Alkyne **2a** (6 mmol, 2.0 equiv.), $(MeO)_3SiH$ (18 mmol, 6.0 equiv.) and NMP/THF (v/v = 2/1, 15 mL) were then added via syringe. The tube was sealed with a Teflon lined cap and stirred at 0 ˚C for 48 h. The reaction mixture was then diluted with EtOAc (~20 mL) and filtered through a pad of celite. The filtrate was added brine (20 mL) and extracted with EtOAc (2×15 mL), the combined organic layer was dried over Na_2SO_4, filtrated and concentrated under vacuum. The residue was then purified by flash column chromatography to give desired product **3** in 66% yield with 96% *ee*.

Preparation of chiral amino ketone and amino alcohol analogues

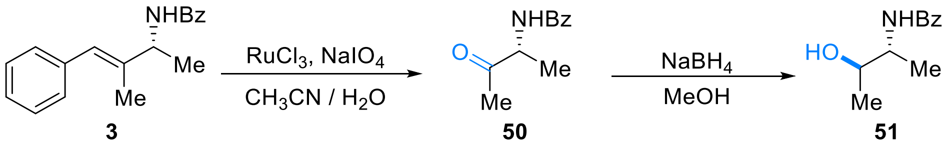

A 4 mL screw-cap reaction tube equipped with a stirring bar was charged with (R,E)-N-(3-methyl-4-phenylbut-3-en-2-yl)benzamide **3** (0.2 mmol, 1.0 equiv.), $RuCl_3$•xH_2O (0.004 mmol, 2 mmol%), $NaIO_4$ (0.6 mmol, 3 equiv.), CH_3CN (2 mL, 0.1 M) and H_2O (2 mL, 0.1 M). The mixture was stirred at r.t. for 2 hours. After completion, 2 mL H_2O was added, the reaction mixture was extracted with ethyl acetate (3×2 mL). After concentration under reduced pressure, the crude mixture was purified by column chromatography on silica gel to give the corresponding product **50**.

To a solution of (R)-N-(3-oxobutan-2-yl)benzamide **50** (0.1 mmol, 1.0 equiv.) in MeOH (2 mL) at -78 °C, $NaBH_4$ (0.3 mmol, 3.0 equiv.) was added and the reaction was stirred for 2 hours at -78 °C until TLC indicated that the reaction was complete. The mixture was quenched with saturated aqueous NH_4Cl solution (10 mL). The resulting solution was warmed to room temperature, extracted with EtOAc (10 mL × 3), and the combined organic layers were washed with brine (20 mL), dried over Na_2SO_4, and concentrated under vacuum. The residue was then purified by column chromatography on silica gel to give the corresponding product **51**.

(R)-N-(3-oxobutan-2-yl)benzamide

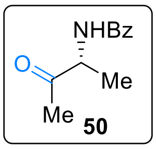

The product **50** was purified with silica gel chromatography (PE/EA = 3:1) as a colorless oil (75%). ^{1}H NMR (600 MHz, Chloroform-*d*) δ 7.82 – 7.78 (m, 2H), 7.52 – 7.47 (m, 1H), 7.45 – 7.39 (m, 2H), 7.11 (s, 1H), 4.81 – 4.74 (m, 1H), 2.27 (s, 3H), 1.48 (d, *J* = 7.2 Hz, 3H). ^{13}C NMR (151 MHz, Chloroform-*d*) δ 207.09, 166.84, 134.04, 131.82, 128.68, 127.13, 55.13, 26.71, 17.74. HRMS (ESI): m/z calcd. for $C_{11}H_{13}NO_2Na^+$ [M + Na$^+$]: 214.0838, found: 214.0847. $[\alpha]_D{}^{20}$ = -3.3 (c = 1.0, $CHCl_3$), HPLC chiralcel AD-H column (10% isopropanol in hexanes, 1.0 mL/min, λ = 245 nm), t_R = 9.4 min (major), 10.2 min (minor), 96% *ee*.

N-((2R)-3-hydroxybutan-2-yl)benzamide

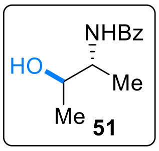

The product **51** was purified with silica gel chromatography (PE/EA = 1:1) as a white solid (85%). ^{1}H NMR (400 MHz, Chloroform-*d*) δ 7.80 – 7.72 (m, 2H), 7.52 – 7.44 (m, 1H), 7.44 – 7.35 (m, 2H), 6.66 – 6.49 (m, 1H), 4.23 – 4.13 (m, 0.72H), 4.13 – 4.04 (m, 0.27H), 3.96 (qd, *J* = 6.5, 2.8 Hz, 0.72H), 3.83 (qd, *J* = 6.3, 3.6 Hz, 0.28H), 2.72 (s, 1H), 1.26 (d, *J* = 6.8 Hz, 1H), 1.23 – 1.15 (m, 5H). ^{13}C NMR (151 MHz, Chloroform-*d*) δ 168.04, 167.94, 134.61, 134.46, 131.70, 131.64, 128.67, 127.11, 127.08, 70.85, 70.37, 51.04, 51.01, 20.82, 19.15, 18.23, 14.36. HRMS (ESI): m/z calcd. for $C_{11}H_{15}NO_2Na^+$ [M + Na$^+$]: 216.0995, found: 216.1001. $[\alpha]_D{}^{20}$ = 7.5 (c = 1.0, $CHCl_3$), HPLC chiralcel AD-H column (10% isopropanol in hexanes, 0.5 mL/min, λ = 225 nm), t_R = 14.5 min (minor), 15.0 min (major), 15.6 min (minor), 16.4 min (major), 97% *ee*, *dr* = 2.7:1.

Preparation of epoxidation product of chiral allylic amine

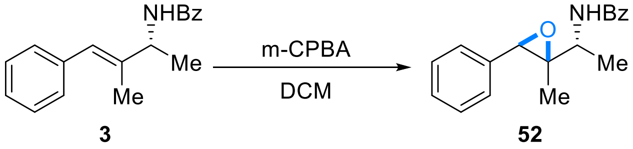

A 4 mL screw-cap reaction tube equipped with a stirring bar was charged with (R,E)-N-(3-methyl-4-phenylbut-3-en-2-yl)benzamide **3** (0.2 mmol, 1.0 equiv.) and 1 mL DCM, and m-CPBA (0.36 mmol, 1.8 equiv.) was then added. The mixture was stirred at room temperature for 2 hours. After completion, 1 mL saturated $NaHCO_3$ was added and then the reaction mixture was extracted with dichloromethane (3 x 2 mL). After concentration under reduced pressure, the crude mixture was purified by column chromatography on silica gel to give the corresponding product **52**.

N-((1R)-1-(2-methyl-3-phenyloxiran-2-yl)ethyl)benzamide

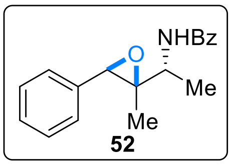

The product **52** was purified with silica gel chromatography (PE/EA = 3:1) as a white solid (71%).

^{1}H NMR (600 MHz, Chloroform-*d*) δ 7.97 – 7.89 (m, 1H), 7.88 – 7.79 (m, 1H), 7.50 – 7.44 (m, 2H), 7.43 – 7.30 (m, 6H), 5.03 (s, 0.46H), 4.89 (s, 0.51H), 4.55 (q, *J* = 7.1 Hz, 0.51H), 4.12 (q, *J* = 6.9 Hz, 0.46H), 2.83 (s, 0.33H), 2.55 (s, 0.36H), 1.50 (d, *J* = 6.9 Hz, 1.47H), 1.28 (s, 1.48H), 1.23 (s, 1.57H), 1.10 (d, *J* = 7.1 Hz, 1.56H). ^{13}C NMR (151 MHz, Chloroform-*d*) δ 161.61, 161.49, 141.19, 139.09, 131.54, 131.45, 128.50, 128.43, 128.32, 128.25, 128.20, 128.06, 127.92, 127.87, 127.82, 127.43, 90.23, 87.46, 77.72, 73.29, 70.67, 64.54, 19.63, 17.19, 16.87, 16.72. HRMS (ESI): m/z calcd. for $C_{18}H_{19}NO_2Na^+$ [M + Na^+]: 304.1308, found: 304.1322. $[\alpha]_D^{20}$ = 25.8 (c = 1.0, $CHCl_3$), HPLC chiralcel OD-H column (10% isopropanol in hexanes, 1.0 mL/min, λ = 264 nm), t_R = 7.0 min (major), 8.0 min (minor), 8.8 min (minor), 10.8 min (major), 98% *ee*, *dr* = 1.1:1.

Deprotection and protection of chiral allylic amine

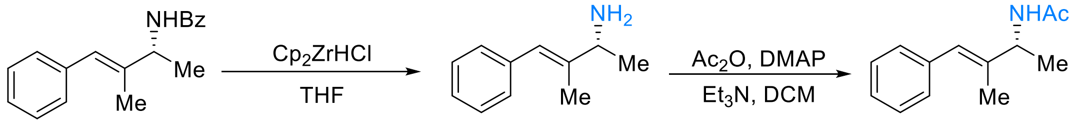

3 **53** **54**

In a glovebox, Schwartz's reagent (1.6 mmol, 8.0 equiv.) and **3** (0.2 mmol, 1.0 equiv.) was added to an oven-dried 25 mL vial, then anhydrous THF (2 mL, 0.1 M) were added. The vial was sealed with a septum cap, and the reaction mixture was transferred out of the glovebox and stirred at room temperature for 3 h. After the reaction complete, water (4 mL) was added and the resulting mixture was stirred at room temperature for another 15 min, then 10 mL NaOH (2 M) solution was added, and the reaction mixture was extracted with Et_2O (3 × 5 mL). The combined organic layers were dried over Na_2SO_4, concentrated under reduced pressure. The product was purified by column chromatography on silica gel (PE/ EA = 1:2, with 1% Et_3N) to afford the crude product **53**.

To a solution of crude **53** (0.2 mmol, 1.0 equiv.) in DCM, DMAP (0.02 mmol, 10 mol%), Et_3N (0.3 mmol, 1.5 equiv.) and Ac_2O (0.4 mmol, 2.0 equiv.) was added. The resulting mixture was stirred overnight and then was quenched with saturated aqueous NH_4Cl and extracted with EtOAc. The organic layer was dried over anhydrous Na_2SO_4 and concentrated. The residue was purified by column chromatography on silica gel to give product **54**.

(R,E)-N-(3-methyl-4-phenylbut-3-en-2-yl)acetamide

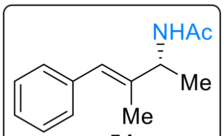

54

The product **54** was purified with silica gel chromatography (PE/EA = 1:1) as a yellow oil (80% over 2 steps). ^{1}H NMR (600 MHz, Chloroform-*d*) δ 7.34 – 7.29 (m, 2H), 7.25 – 7.18 (m, 3H), 6.45 (s, 1H), 5.62 (d, *J* = 7.9 Hz, 1H), 4.66 – 4.58 (m, 1H), 2.02 (s, 3H), 1.86 (s, 3H), 1.33 (d, *J* = 6.8 Hz, 3H). ^{13}C NMR (151 MHz, Chloroform-*d*) δ 169.40, 138.86, 137.68, 129.08, 128.20, 126.54, 125.02, 51.65, 23.66, 19.91, 15.43. HRMS (ESI): m/z calcd. for $C_{13}H_{17}NONa^+$ [M + Na^+]: 226.1202, found:

226.1209. $[\alpha]_D^{20}$ = 28.2 (c = 1.0, $CHCl_3$), HPLC chiralcel AD-H column (10% isopropanol in hexanes, 1.0 mL/min, λ = 245 nm), t_R = 6.2 min (major), 6.9 min (minor), 96% *ee*.

General Procedure for Synthesis of Redox-Active NHP Esters

Redox-Active NHP Esters **60** and **S1-S4** were prepared according to reported method[1,2].

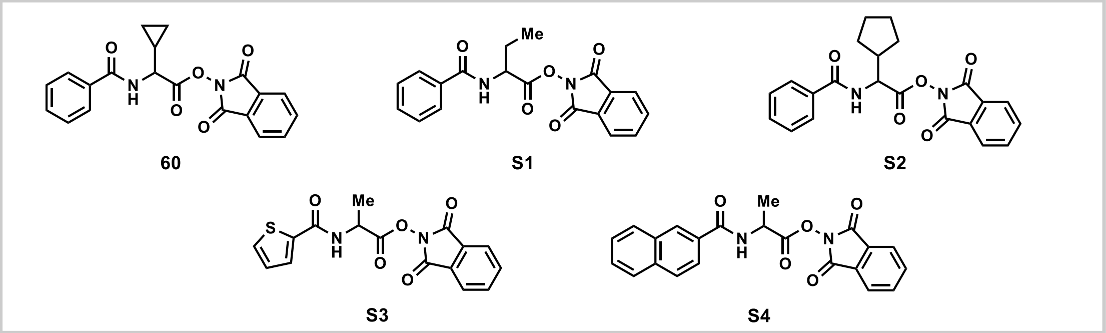

General Procedure

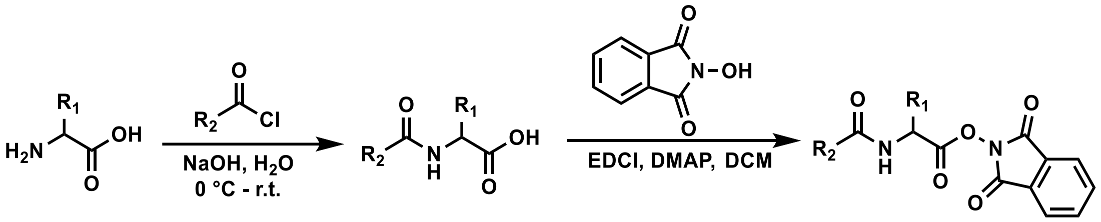

Step 1. The amino acid (1.0 equiv.) and NaOH (4.0 equiv.) were dissolved in H_2O (0.3 M). After cooling to 0 °C, aromatic acyl chloride (1.05 equiv.) was added dropwise at this temperature. After the addition was completed, the mixture was stirred for additional 2 h at 0 °C. Subsequently, the mixture was allowed to warm to r.t. and was stirred for one additional hour. All volatiles were then removed under reduced pressure before conc. HCl was added to cause precipitation. The mixture was filtered and the filter cake was washed with ice-cold diethylether to afford crude product as a white solid.

Step 2. To a flask containing N-acyl-protected-amino acid (1.0 equiv.), EDCI (1.0 equiv.) and DMAP (0.1 equiv.) in DCM (25 mL) was added N-hydroxyphthalimide (1.0 equiv.) portion-wise over 30 min with rapid stirring waiting for the intermediary yellow color to fade between each addition. After the final addition the reaction was stirred for a further 30 min. Then the mixture was washed by deionized water and saturated brine for three times. Recrystallization from the residue with n-hexane/dichloromethane gave pure product as a white solid.

1,3-dioxoisoindolin-2-yl 2-benzamido-2-cyclopropylacetate

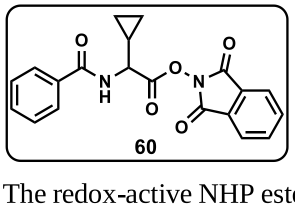

The redox-active NHP ester **60** was purified by recrystallization as a white solid (36% over 2 steps). ^{1}H NMR (400 MHz, Methylene Chloride-d_2) δ 7.94 – 7.85 (m, 2H), 7.88 – 7.77 (m, 4H), 7.61 – 7.52 (m, 1H), 7.52 – 7.44 (m, 2H), 6.72 (d, J = 6.8 Hz, 1H), 4.42 (dd, J = 9.1, 6.8 Hz, 1H), 1.47 –

1.34 (m, 1H), 0.94 – 0.81 (m, 1H), 0.83 – 0.71 (m, 2H), 0.73 – 0.60 (m, 1H). ^{13}C NMR (101 MHz, Methylene Chloride-*d*$_2$) δ 168.95, 167.28, 161.95, 135.31, 133.59, 132.43, 129.18, 129.02, 127.52, 124.30, 55.60, 13.82, 4.33, 4.06. HRMS (ESI): m/z calcd. for $C_{20}H_{16}N_2O_5Na^+$ [M + Na$^+$]: 387.0951, found: 387.0957.

1,3-dioxoisoindolin-2-yl 2-benzamidobutanoate

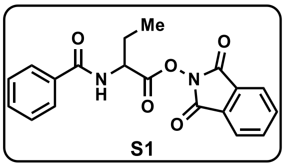

S1

The redox-active NHP ester **S1** was purified by recrystallization as a white solid (48% over 2 steps). ^{1}H NMR (400 MHz, Chloroform-*d*) δ 7.94 – 7.86 (m, 2H), 7.86 – 7.76 (m, 4H), 7.57 – 7.49 (m, 1H), 7.49 – 7.41 (m, 2H), 6.66 (d, *J* = 8.2 Hz, 1H), 5.32 – 5.22 (m, 1H), 2.30 – 2.16 (m, 1H), 2.16 – 2.02 (m, 1H), 1.17 (t, *J* = 7.4 Hz, 3H). ^{13}C NMR (101 MHz, Chloroform-*d*) δ 169.45, 167.15, 161.64, 135.07, 133.61, 132.18, 128.91, 128.84, 127.28, 124.25, 52.11, 26.40, 9.47. HRMS (ESI): m/z calcd. for $C_{19}H_{16}N_2O_5Na^+$ [M + Na$^+$]: 375.0951, found: 375.0940.

1,3-dioxoisoindolin-2-yl 2-benzamido-2-cyclopentylacetate

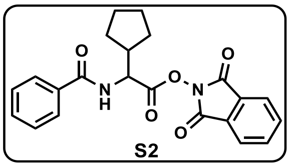

S2

The redox-active NHP ester **S2** was purified by recrystallization as a white solid (34% over 2 steps). ^{1}H NMR (400 MHz, Methylene Chloride-*d*$_2$) δ 7.93 – 7.86 (m, 2H), 7.85 – 7.77 (m, 4H), 7.63 – 7.51 (m, 1H), 7.52 – 7.43 (m, 2H), 6.61 (d, *J* = 8.3 Hz, 1H), 5.10 – 5.01 (m, 1H), 2.63 – 2.48 (m, 1H), 2.09 – 1.91 (m, 2H), 1.81 – 1.46 (m, 6H). ^{13}C NMR (101 MHz, Methylene Chloride-*d*$_2$) δ 169.42, 167.56, 162.00, 135.33, 133.98, 132.38, 129.18, 129.05, 127.52, 124.32, 54.76, 42.90, 29.55, 29.13, 25.76, 25.46. HRMS (ESI): m/z calcd. for $C_{22}H_{20}N_2O_5Na^+$ [M + Na$^+$]: 415.1264, found: 415.1280.

1,3-dioxoisoindolin-2-yl (thiophene-2-carbonyl)alaninate

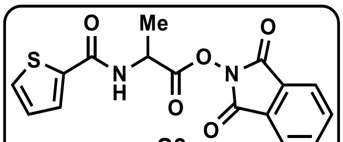

S3

The redox-active NHP ester **S3** was purified by recrystallization as a white solid (10% over 2 steps). ^{1}H NMR (600 MHz, Methylene Chloride-*d*$_2$) δ 7.92 – 7.87 (m, 2H), 7.85 – 7.79 (m, 2H), 7.60 – 7.55 (m, 2H), 7.14 – 7.10 (m, 1H), 6.50 (d, *J* = 7.5 Hz, 1H), 5.16 – 5.08 (m, 1H), 1.74 (d, *J* = 7.3 Hz, 3H). ^{13}C NMR (151 MHz, Methylene Chloride-*d*$_2$) δ 170.17, 161.93, 161.59, 138.00, 135.36, 131.35, 129.09, 128.19, 124.35, 47.19, 18.47. HRMS (ESI): m/z calcd. for $C_{16}H_{12}N_2O_5SNa^+$ [M + Na$^+$]: 367.0359, found: 367.0361.

1,3-dioxoisoindolin-2-yl (2-naphthoyl)alaninate

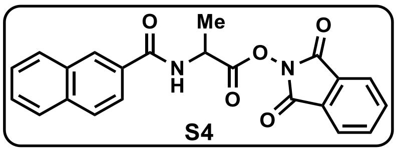

The redox-active NHP ester **S4** was purified by recrystallization as a white solid (22% over 2 steps). ^{1}H NMR (400 MHz, Chloroform-*d*) δ 8.37 – 8.33 (m, 1H), 7.97 – 7.85 (m, 6H), 7.85 – 7.77 (m, 2H), 7.62 – 7.51 (m, 2H), 6.83 (d, *J* = 7.7 Hz, 1H), 5.42 – 5.30 (m, 1H), 1.82 (d, *J* = 7.2 Hz, 3H). ^{13}C NMR (101 MHz, Methylene Chloride-d_2) δ 170.35, 167.31, 162.00, 135.36, 135.33, 132.91, 130.88, 129.33, 129.12, 128.90, 128.32, 128.16, 128.10, 127.27, 124.36, 123.90, 47.44, 18.52. HRMS (ESI): m/z calcd. for $C_{22}H_{16}N_2O_5Na^+$ [M + Na$^+$]: 411.0951, found: 411.0954.

General Procedure for Synthesis of Alkynes

Alkynes **S5-S11** were prepared according to reported method[3-5].

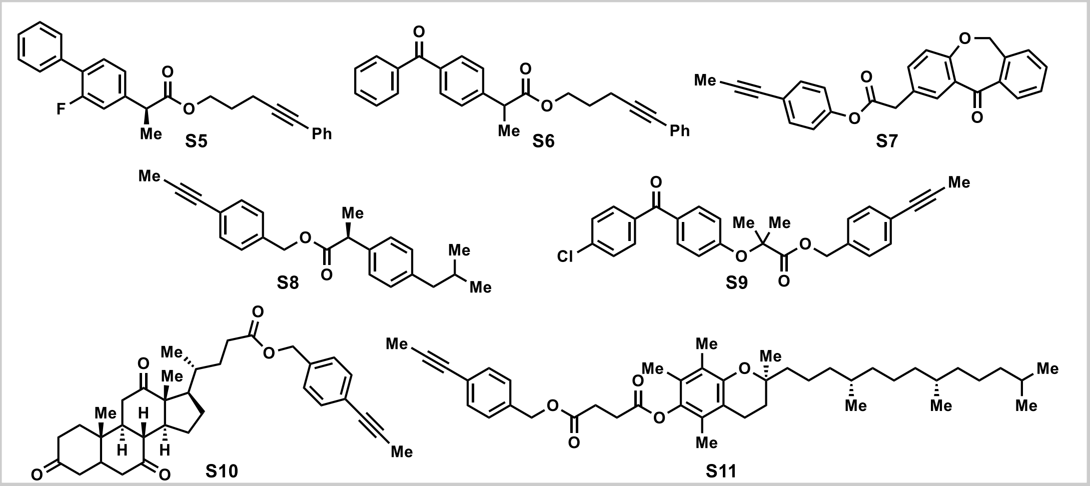

General Procedure 1

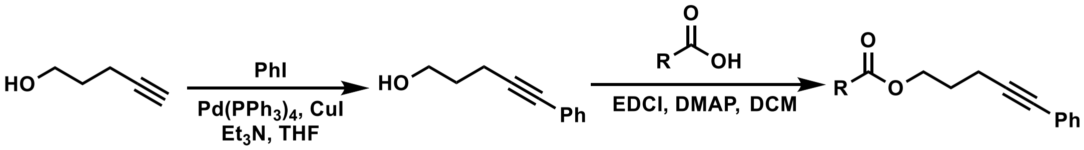

Step 1. $Pd(PPh_3)_4$ (0.3 mmol, 0.01 equiv.) and CuI (0.54 mmol, 0.018 equiv.) were added to the solution of iodobenzene (60 mmol, 2.0 equiv.) and 5-hydroxy pentyne (30 mmol, 1.0 equiv.) in triethylamine (600 mmol, 20 equiv.) and THF (15 mL) under N_2, and then the reaction mixture was stirred at r.t. for 12 h. The mixture was filtered and the filtrate was concentrated under reduced pressure. The product was purified with silica gel chromatography (PE/EA = 5:1) as a yellow oil. The data was consistent with the literature published before[3].

Step 2. A solution of 5-phenylpent-4-yn-1-ol (10 mmol, 1.0 equiv.), DMAP (1 mmol, 0.10 equiv.) and carboxylic acid (12 mmol, 1.2 equiv.) in DCM (30 mL) was cooled to 0 °C for 10 minutes. Then EDCI (20 mmol, 2.0 equiv.) was added, the resulting solution was allowed to warm to r.t. for 12 h.

The reaction mixture was diluted with H_2O and extracted with DCM (2 x 50 mL). The combined organic layers were dried over Na_2SO_4, filtered, and concentrated. The product was purified with silica gel chromatography as a solid or oil.

5-phenylpent-4-yn-1-yl (S)-2-(2-fluoro-[1,1'-biphenyl]-4-yl)propanoate

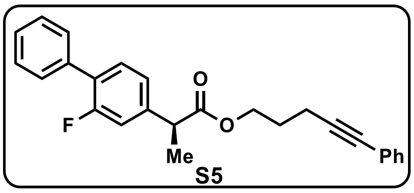

S5

The alkyne **S5** was purified with silica gel chromatography (PE/EA = 20:1) as a colorless oil (88% over 2 steps). ^{1}H NMR (400 MHz, Chloroform-*d*) δ 7.62 – 7.55 (m, 2H), 7.51 – 7.37 (m, 6H), 7.36 – 7.28 (m, 3H), 7.24 – 7.16 (m, 2H), 4.31 (t, *J* = 6.3 Hz, 2H), 3.82 (q, *J* = 7.1 Hz, 1H), 2.48 (t, *J* = 7.0 Hz, 2H), 1.96 (p, *J* = 6.7 Hz, 2H), 1.59 (d, *J* = 7.2 Hz, 3H). ^{19}F NMR (376 MHz, Chloroform-*d*) δ -117.49. ^{13}C NMR (101 MHz, Chloroform-*d*) δ 174.00, 159.72 (d, *J* = 248.3 Hz), 141.91 (d, *J* = 7.7 Hz), 135.52 (d, *J* = 1.4 Hz), 131.61, 130.88 (d, *J* = 4.0 Hz), 129.01 (d, *J* = 2.9 Hz), 128.50, 128.29, 127.80, 127.78, 127.72, 123.65, 123.60 (d, *J* = 3.3 Hz), 115.28 (d, *J* = 23.7 Hz), 88.49, 81.41, 63.75, 45.08, 27.71, 18.39, 16.10. HRMS (ESI): m/z calcd. for $C_{26}H_{23}FO_2H^+$ [M + H$^+$]: 387.1755, found: 387.1759.

5-phenylpent-4-yn-1-yl 2-(4-benzoylphenyl)propanoate

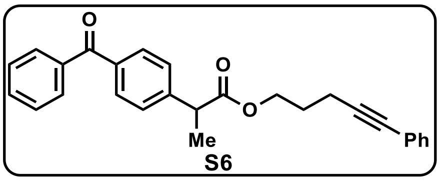

S6

The alkyne **S6** was purified with silica gel chromatography (PE/EA = 10:1) as a colorless oil (91% over 2 steps). ^{1}H NMR (400 MHz, Chloroform-*d*) δ 7.67 – 7.60 (m, 3H), 7.56 – 7.49 (m, 1H), 7.45 – 7.38 (m, 2H), 7.36 – 7.27 (m, 3H), 7.24 – 7.17 (m, 2H), 7.16 – 7.06 (m, 3H), 4.09 (t, *J* = 6.3 Hz, 2H), 3.66 (q, *J* = 7.2 Hz, 1H), 2.25 (t, *J* = 7.0 Hz, 2H), 1.73 (p, *J* = 6.7 Hz, 2H), 1.39 (d, *J* = 7.2 Hz, 3H). ^{13}C NMR (101 MHz, Chloroform-*d*) δ 196.55, 174.06, 140.91, 137.92, 137.50, 132.56, 131.58, 131.54, 130.10, 129.24, 129.08, 128.62, 128.36, 128.26, 127.78, 123.60, 88.45, 81.37, 63.67, 45.43, 27.69, 18.45, 16.06. HRMS (ESI): m/z calcd. for $C_{27}H_{24}O_3Na^+$ [M + Na$^+$]: 419.1618, found: 419.1627.

General Procedure 2

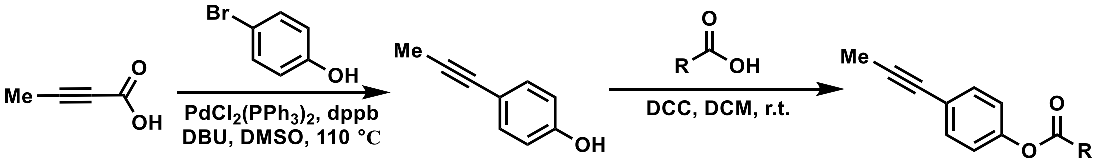

Step 1. To a 100 mL Schlenk tube, $PdCl_2(PPh_3)_2$ (0.1 mmol, 0.01 equiv.), 1,4-bis(diphenylphosphino)butane (dppb) (0.2 mmol, 0.02 equiv.), 2-butynoic acid (12 mmol, 1.2 equiv.), 4-bromophenol (10 mmol, 1.0 equiv.), DMSO (30 mL), and DBU (30 mmol, 3.0 equiv.) were added. The tube was degassed under reduced pressure and refilled with N_2 for three times. The solution was stirred at 110 ˚C for 12 h. After cooling to room temperature, sat. NH_4Cl aq. was poured into the reaction mixture and extracted with DCM. The organic layer was washed with water, dried over Na_2SO_4, and filtered. The filtrate was concentrated in vacuo. The residue was purified by silica gel column chromatography to afford 4-(prop-1-yn-1-yl)phenol. The data was consistent with the literature published before[4].

Step 2. A solution of 4-(prop-1-yn-1-yl)phenol (12 mmol, 1.2 equiv.), carboxylic acid (10 mmol, 1.0 equiv.) and DCC (15 mmol, 1.5 equiv.) in DCM (30 mL) was stirred at r.t. for 16 h. After the reaction was completed, the reaction mixture was filtered and washed with DCM. The filtrate was concentrated in vacuo. The product was purified with silica gel chromatography as a solid or oil.

4-(prop-1-yn-1-yl)phenyl 2-(11-oxo-6,11-dihydrodibenzo[b,e]oxepin-2-yl)acetate

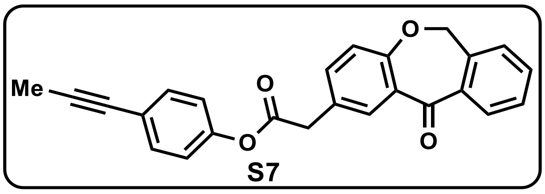

The alkyne **S7** was purified with silica gel chromatography (PE/EA = 10:1) as a white solid (30% over 2 steps). ^{1}H NMR (400 MHz, Chloroform-*d*) δ 8.21 (d, *J* = 2.4 Hz, 1H), 7.90 (dd, *J* = 7.7, 1.4 Hz, 1H), 7.60 – 7.53 (m, 1H), 7.52 – 7.34 (m, 5H), 7.10 – 7.04 (m, 1H), 7.03 – 6.94 (m, 2H), 5.20 (s, 2H), 3.87 (s, 2H), 2.03 (s, 3H). ^{13}C NMR (101 MHz, Chloroform-*d*) δ 190.93, 169.76, 160.77, 149.97, 140.52, 136.39, 135.60, 132.96, 132.68, 132.56, 129.61, 129.42, 127.96, 127.18, 125.34, 123.38, 121.51, 121.40, 86.13, 79.01, 73.75, 40.37, 4.42. HRMS (ESI): m/z calcd. for $C_{25}H_{18}O_4Na^+$ [M + Na$^+$]: 405.1097, found: 405.1100.

General Procedure 3

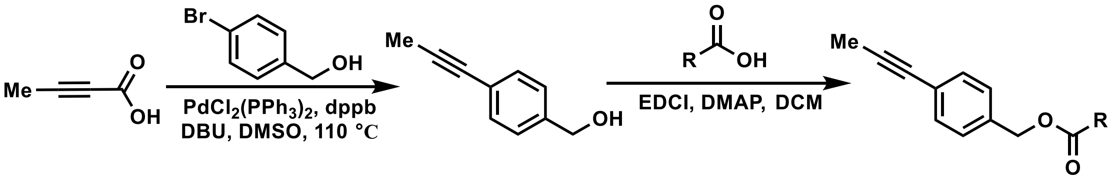

Step 1. To a 100 mL Schlenk tube, $PdCl_2(PPh_3)_2$ (0.1 mmol, 0.01 equiv.), 1,4-bis(diphenylphosphino)butane (dppb) (0.2 mmol, 0.02 equiv.), 2-butynoic acid (12 mmol, 1.2 equiv.), (4-bromophenyl)methanol (10 mmol, 1.0 equiv.), DMSO (30 mL), and DBU (30 mmol, 3.0 equiv.) were added. The tube was degassed under reduced pressure and refilled with N_2 for three times. The solution was stirred at 110 °C for 12 h. After cooling to room temperature, sat. NH_4Cl

aq. was poured into the reaction mixture and extracted with DCM. The organic layer was washed with water, dried over Na_2SO_4, and filtered. The filtrate was concentrated in vacuo. The residue was purified by silica gel column chromatography to afford (4-(prop-1-yn-1-yl)phenyl)methanol. The data was consistent with the literature published before[5].

Step 2. A solution of (4-(prop-1-yn-1-yl)phenyl)methanol (10 mmol, 1.0 equiv.), DMAP (1 mmol, 0.10 equiv.) and carboxylic acid (12 mmol, 1.2 equiv.) in DCM (30 mL) was cooled to 0 °C for 10 minutes. Then EDCI (20 mmol, 2.0 equiv.) was added, the resulting solution was allowed to warm to r.t. for 12 h. The reaction mixture was diluted with H_2O and extracted with DCM (2 x 50 mL). The combined organic layers were dried over Na_2SO_4, filtered, and concentrated. The product was purified with silica gel chromatography as a solid or oil.

4-(prop-1-yn-1-yl)benzyl (S)-2-(4-isobutylphenyl)propanoate

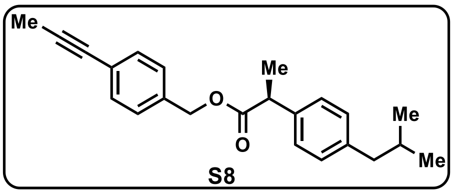

S8

The alkyne **S8** was purified with silica gel chromatography (PE/EA = 25:1) as a white solid (65% over 2 steps). ^{1}H NMR (500 MHz, Chloroform-*d*) δ 7.31 (d, *J* = 7.9 Hz, 2H), 7.19 (d, *J* = 7.8 Hz, 2H), 7.11 (dd, *J* = 18.3, 8.0 Hz, 4H), 5.07 (s, 2H), 3.75 (q, *J* = 7.1 Hz, 1H), 2.45 (d, *J* = 7.2 Hz, 2H), 2.05 (s, 3H), 1.91 – 1.79 (m, 1H), 1.51 (d, *J* = 7.2 Hz, 3H), 0.91 (d, *J* = 6.7 Hz, 6H). ^{13}C NMR (126 MHz, Chloroform-*d*) δ 174.60, 140.75, 137.64, 135.50, 131.65, 129.46, 127.72, 127.33, 123.86, 86.34, 79.52, 66.03, 45.24, 45.15, 30.34, 22.51, 18.50, 4.48. HRMS (ESI): m/z calcd. for $C_{23}H_{26}O_2Na^+$ [M + Na$^+$]: 357.1825, found: 357.1828.

4-(prop-1-yn-1-yl)benzyl 2-(4-(4-chlorobenzoyl)phenoxy)-2-methylpropanoate

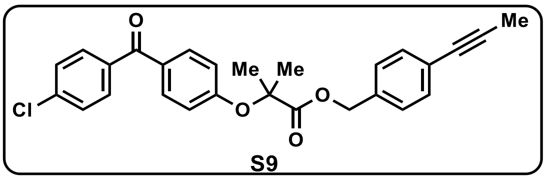

S9

The alkyne **S9** was purified with silica gel chromatography (PE/EA = 20:1) as a white solid (63% over 2 steps). ^{1}H NMR (500 MHz, Chloroform-*d*) δ 7.72 – 7.66 (m, 2H), 7.66 – 7.61 (m, 2H), 7.49 – 7.43 (m, 2H), 7.30 (d, *J* = 8.2 Hz, 2H), 7.16 (d, *J* = 8.0 Hz, 2H), 6.79 – 6.73 (m, 2H), 5.16 (s, 2H), 2.03 (s, 3H), 1.67 (s, 6H). ^{13}C NMR (126 MHz, Chloroform-*d*) δ 194.26, 173.55, 159.57, 138.46, 136.43, 134.39, 132.08, 131.69, 131.31, 130.45, 128.67, 128.48, 124.43, 117.28, 86.93, 79.48, 79.34, 67.05, 25.53, 4.46. HRMS (ESI): m/z calcd. for $C_{27}H_{23}ClO_4H^+$ [M + H$^+$]: 447.1358, found: 447.1369.

4-(prop-1-yn-1-yl)benzyl (4R)-4-((8R,9S,10S,13R,14S,17R)-10,13-dimethyl-3,7,12-trioxohexadecahydro-1H-cyclopenta[a]phenanthren-17-yl)pentanoate

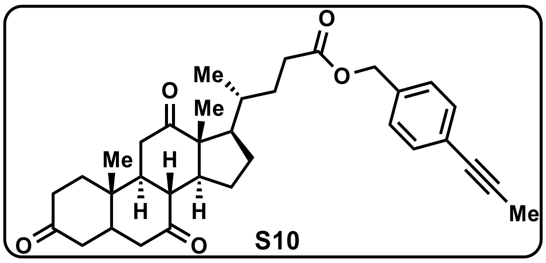

The alkyne **S10** was purified with silica gel chromatography (PE/EA = 4:1) as a white solid (45% over 2 steps). ^{1}H NMR (500 MHz, Chloroform-*d*) δ 7.39 – 7.34 (m, 2H), 7.28 – 7.23 (m, 2H), 5.12 – 5.02 (m, 2H), 2.95 – 2.79 (m, 3H), 2.48 – 2.39 (m, 1H), 2.37 – 2.18 (m, 7H), 2.18 – 2.07 (m, 2H), 2.04 (s, 3H), 2.03 – 1.92 (m, 4H), 1.90 – 1.78 (m, 2H), 1.65 – 1.54 (m, 1H), 1.39 (s, 3H), 1.45 – 1.34 (m, 1H), 1.30 – 1.19 (m, 3H), 1.02 (s, 3H), 0.83 (d, *J* = 6.7 Hz, 3H). ^{13}C NMR (126 MHz, Chloroform-*d*) δ 212.10, 209.26, 208.88, 173.94, 135.46, 131.74, 128.21, 124.06, 86.49, 79.49, 65.84, 56.98, 51.82, 49.09, 46.97, 45.74, 45.62, 45.10, 42.91, 38.74, 36.62, 36.13, 35.52, 35.38, 31.62, 30.51, 27.69, 25.23, 22.02, 18.73, 11.94, 4.50. HRMS (ESI): m/z calcd. for $C_{34}H_{42}O_5Na^+$ [M + Na$^+$]: 553.2924, found: 553.2933.

4-(prop-1-yn-1-yl)benzyl ((S)-2,5,7,8-tetramethyl-2-((4S,8S)-4,8,12-trimethyltridecyl)chroman-6-yl) succinate

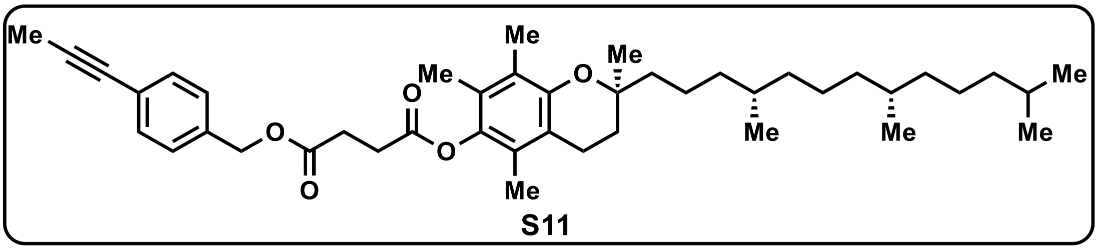

The alkyne **S11** was purified with silica gel chromatography (PE/EA = 10:1) as a white solid (62% over 2 steps). ^{1}H NMR (400 MHz, Chloroform-*d*) δ 7.42 – 7.35 (m, 2H), 7.31 – 7.26 (m, 2H), 5.15 (s, 2H), 3.01 – 2.93 (m, 2H), 2.87 – 2.79 (m, 2H), 2.61 (t, *J* = 6.8 Hz, 2H), 2.13 (s, 3H), 2.07 (s, 3H), 2.03 (s, 3H), 1.98 (s, 3H), 1.90 – 1.74 (m, 2H), 1.64 – 1.52 (m, 3H), 1.52 – 1.22 (m, 15H), 1.22 – 1.06 (m, 6H), 0.94 – 0.86 (m, 12H). ^{13}C NMR (101 MHz, Chloroform-*d*) δ 171.99, 170.94, 149.46, 140.48, 135.06, 131.68, 128.07, 126.72, 124.99, 124.10, 123.04, 117.39, 86.37, 79.46, 75.05, 66.24, 39.43, 37.51, 37.49, 37.34, 32.85, 32.75, 29.20, 28.86, 28.04, 24.87, 24.51, 22.80, 22.71, 21.07, 20.64, 19.83, 19.73, 12.97, 12.10, 11.87, 4.37. HRMS (ESI): m/z calcd. for $C_{43}H_{62}O_5Na^+$ [M + Na$^+$]: 681.4489, found: 681.4495.

Supplementary Discussion

Mechanism Studies

Regioselectivity-Determining Step

Regioselectivity depends on the electronic effect of alkynes

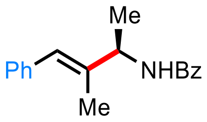

3, 71% yield, single r.r., 96% ee

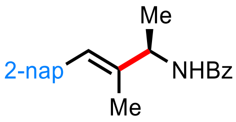

4, 61% yield, single r.r., 91% ee

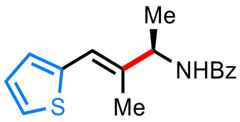

16, 40% yield, single r.r., 88% ee

In glove box, NiCl$_2$•DME (0.01 mmol, 10 mol %), L6 (0.012 mmol, 12 mol%), Ca(OAc)$_2$ (0.30 mmol, 3.0 equiv.) and alkyl NHP ester **1a** (0.10 mmol, 1.0 equiv.) were combined in a 5 mL oven-dried sealing tube. The vessel was evacuated and backfilled with Ar (repeated for 3 times). Alkyne (0.20 mmol, 2.0 equiv.), (MeO)$_3$SiH (0.60 mmol, 6.0 equiv.) and NMP/THF (v/v = 2/1, 0.5 mL) were then added via syringe. The tube was sealed with a Teflon lined cap and stirred at 0 ºC for 24 h. As shown above, the single regioselectivity of anti-Markovnikov products were obtained when different alkynes with Ph (**3**), 2-nap (**4**) or thiophene (**16**) group were examined. The regioselectivity (r.r.) was analyzed by ^{1}H NMR.

Regioselectivity depends on steric effect of alkyl group

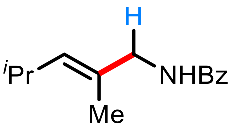

55, 42% yield, 3.2 : 1 r.r.

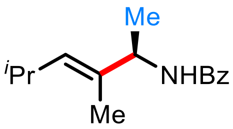

26, 35% yield, 4 : 1 r.r., 96% ee

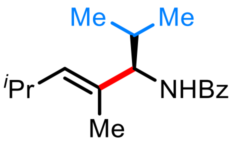

27, 25% yield, 10 : 1 r.r., 96% ee

In glove box, NiCl$_2$•DME (0.01 mmol, 10 mol %), L6 (0.012 mmol, 12 mol%), Ca(OAc)$_2$ (0.30 mmol, 3.0 equiv.) and alkyl NHP ester **1a** (0.10 mmol, 1.0 equiv.) were combined in a 5 mL oven-dried sealing tube. The vessel was evacuated and backfilled with Ar (repeated for 3 times). Internal alkyne, 4-methylpent-2-yne (0.20 mmol, 2.0 equiv.), (MeO)$_3$SiH (0.60 mmol, 6.0 equiv.) and NMP/THF (v/v = 2/1, 0.5 mL) were then added via syringe. The tube was sealed with a Teflon lined cap and stirred at 0 ºC for 24 h. As shown above, as the alkyl group steric hindrance increases, the regioselectivity of the reaction gradually increases (**55**, **26**, **27**). The regioselectivity (r.r.) was analyzed by ^{1}H NMR.

(E)-N-(2,4-dimethylpent-2-en-1-yl)benzamide

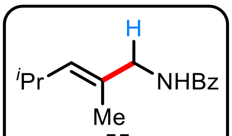

55

The product **55** was purified with silica gel chromatography (PE/EA = 10:1) as a white solid (42%, 3.2 : 1 r.r.). ^{1}H NMR (400 MHz, Chloroform-*d*) δ 7.82 – 7.73 (m, 2H), 7.54 – 7.46 (m, 1H), 7.46 – 7.38 (m, 2H), 6.17 – 5.96 (m, 1H), 5.48 – 5.33 (m, 0.24H), 5.23 – 5.16 (m, 0.76H), 4.04 – 3.94 (m, 2H), 2.98 – 2.80 (m, 0.24H), 2.62 – 2.45 (m, 0.76H), 1.70 – 1.62 (m, 3H), 1.07 (d, *J* = 7.0 Hz, 1.4H), 0.95 (d, *J* = 6.6 Hz, 4.6H). ^{13}C NMR (126 MHz, Chloroform-*d*) δ 167.52, 167.15, 141.51, 135.20, 134.93, 131.51, 131.48, 129.54, 128.70, 127.02, 126.96, 120.74, 47.54, 41.82, 28.03, 27.17, 23.03, 21.07, 14.62, 12.93. HRMS (ESI): m/z calcd. for $C_{14}H_{19}NONa^{+}$ [M + Na^{+}]: 240.1359, found: 240.1366.

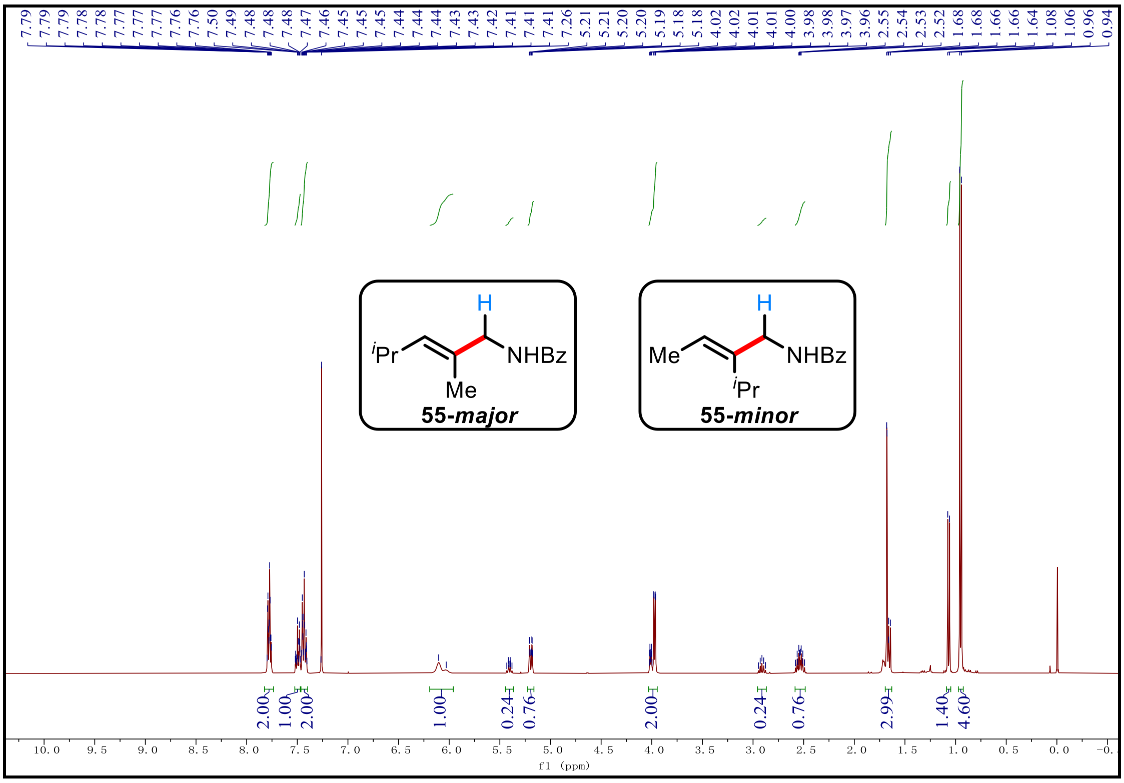

Supplementary Figure 1. ^{1}H NMR spectra of **55** in regioselectivity-determining step

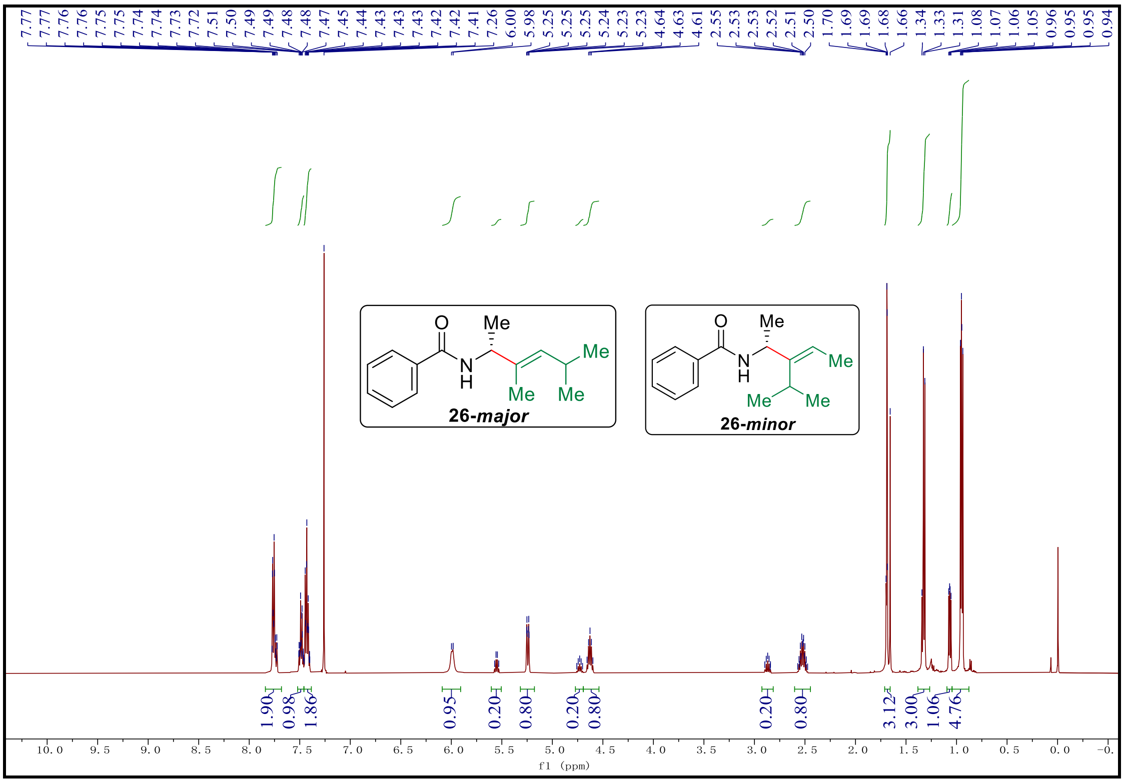

Supplementary Figure 2. ^{1}H NMR spectra of **26** in regioselectivity-determining step

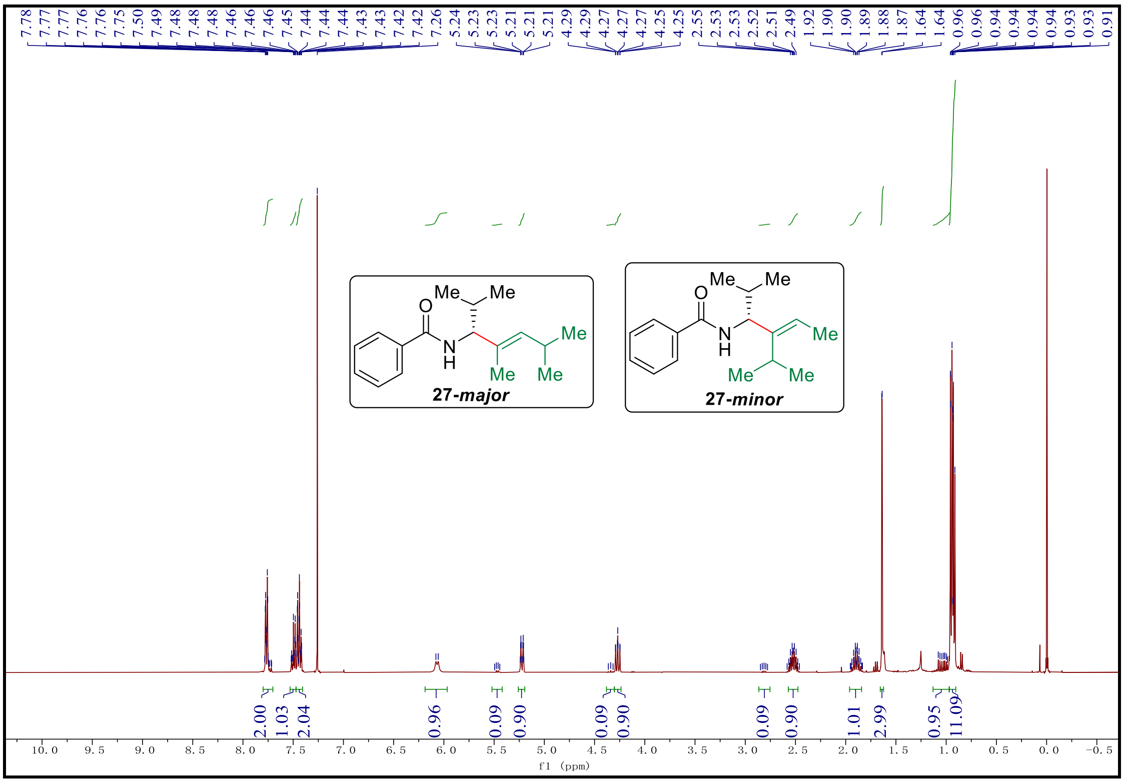

Supplementary Figure 3. ^{1}H NMR spectra of **27** in regioselectivity-determining step

Deuterium-Labeling Experiments

Deuterium-labeling experiment with $PhSiD_3$

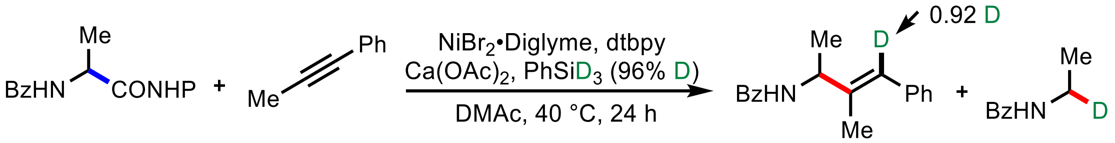

1a **2a** **56**, 48% yield **57**, N.D.

In glove box, $NiBr_2$•Diglyme (0.01 mmol, 10 mol %), dtbpy (0.012 mmol, 12 mol%), $Ca(OAc)_2$ (0.30 mmol, 3.0 equiv.) and alkyl NHP ester **1a** (0.10 mmol, 1.0 equiv.) were combined in a 5 mL oven-dried sealing tube. The vessel was evacuated and backfilled with Ar (repeated for 3 times). Alkyne **2a** (0.20 mmol, 2.0 equiv.), $PhSiD_3$ (96% D, 0.60 mmol, 6.0 equiv.) and DMAc (0.5 mL) were then added via syringe. The tube was sealed with a Teflon lined cap and stirred at 40 ºC for 24 h. The deuterium-containing product **56** was obtained in 48% yield and **57** was not detected at all. As shown below, incorporation of deuterium was analyzed by ^{1}H NMR. For ^{1}H NMR, when deuterium is incorporated, the peak shape and integral of the corresponding position (alkenyl protons, δ 6.54 ppm) will change. Percent deuterium incorporation was derived from the integration value of this signal.

(S,E)-N-(3-methyl-4-phenylbut-3-en-2-yl-4-d)benzamide

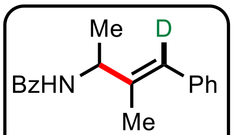

56

The product **56** was purified with silica gel chromatography (PE/EA = 10:1) as a white solid (48%). ^{1}H NMR (400 MHz, Chloroform-*d*) δ 7.86 – 7.77 (m, 2H), 7.54 – 7.48 (m, 1H), 7.48 – 7.41 (m, 2H), 7.36 – 7.30 (m, 2H), 7.29 – 7.25 (m, 2H), 7.24 – 7.16 (m, 1H), 6.54 (s, 0.08H), 6.18 (d, *J* = 8.3 Hz, 1H), 4.90 – 4.79 (m, 1H), 1.94 (s, 3H), 1.46 (d, *J* = 6.8 Hz, 3H). ^{13}C NMR (101 MHz, Chloroform-*d*) δ 166.84, 138.79, 137.60, 134.89, 131.59, 129.13, 128.73, 128.21, 127.03, 126.59, 124.91 (t, *J* = 23.3 Hz), 52.06, 19.99, 15.55. HRMS (ESI): m/z calcd. for $C_{18}H_{18}DNOH^+$ [M + H^+]: 267.1602, found: 267.1611.

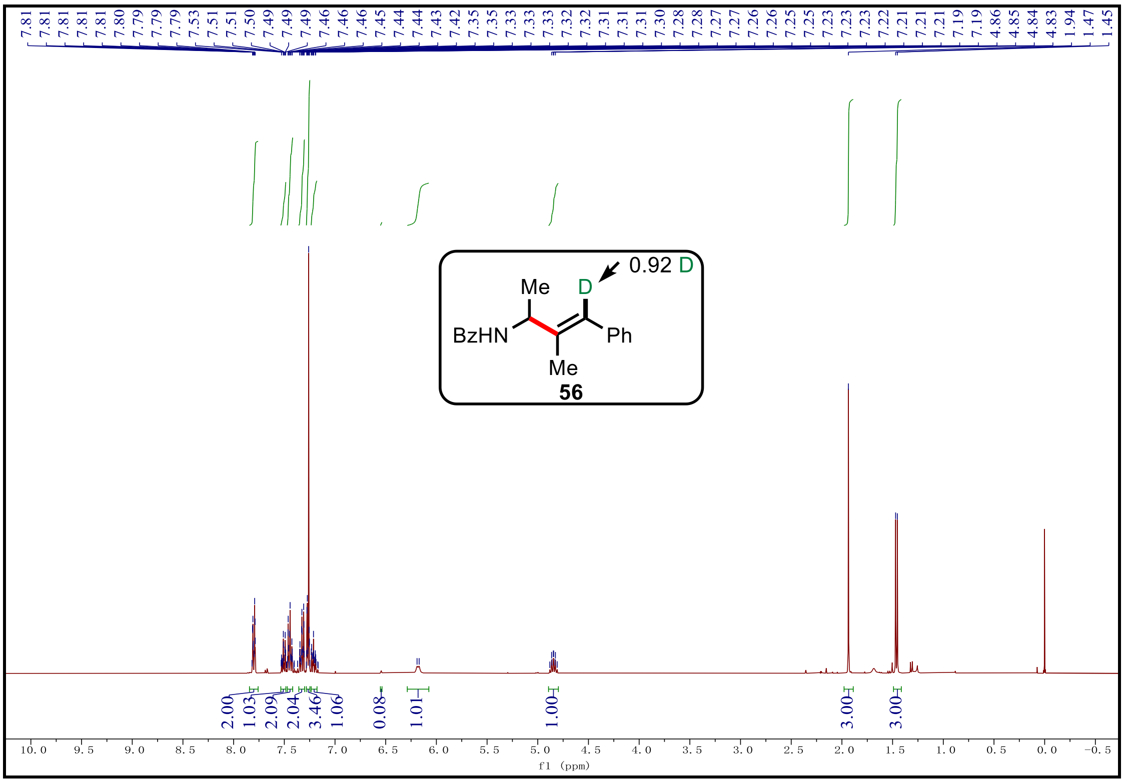

Supplementary Figure 4. ^{1}H NMR spectra of **56** in deuterium-labeling experiment with $PhSiD_3$

Deuterium-labeling experiment without 2a

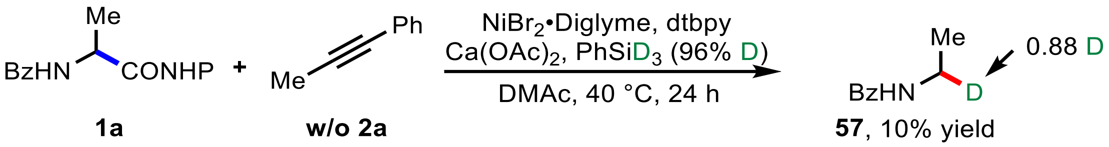

In glove box, $NiBr_2$•Diglyme (0.01 mmol, 10 mol %), dtbpy (0.012 mmol, 12 mol%), $Ca(OAc)_2$ (0.30 mmol, 3.0 equiv.) and alkyl NHP ester **1a** (0.10 mmol, 1.0 equiv.) were combined in a 5 mL oven-dried sealing tube. The vessel was evacuated and backfilled with Ar (repeated for 3 times). $PhSiD_3$ (96% D, 0.60 mmol, 6.0 equiv.) and DMAc (0.5 mL) were then added via syringe. The tube was sealed with a Teflon lined cap and stirred at 40 ºC for 24 h. The deuterium-containing product **57** was obtained in 10% yield. As shown below, incorporation of deuterium was analyzed by ^{1}H NMR. For ^{1}H NMR, when deuterium is incorporated, the peak shape and integral of the corresponding position (alkyl protons, δ 3.56 – 3.42 ppm) will change. Percent deuterium incorporation was derived from the integration value of this signal.

(R)-N-(ethyl-1-d)benzamide

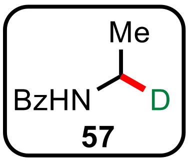

The product **57** was purified with silica gel chromatography (PE/EA = 10:1) as a white solid (10%).

^{1}H NMR (400 MHz, Chloroform-*d*) δ 7.79 – 7.72 (m, 2H), 7.53 – 7.46 (m, 1H), 7.46 – 7.38 (m, 2H), 6.11 (s, 1H), 3.56 – 3.42 (m, 1H), 1.33 – 1.21 (m, 3H). ^{13}C NMR (126 MHz, Chloroform-*d*) δ 167.59, 134.97, 131.46, 128.68, 126.95, 35.07 (C-H), 34.79 (t, *J* = 21.5 Hz, C-D), 14.95. HRMS (ESI): m/z calcd. for $C_9H_{10}DNOH^+$ [M + H^+]: 151.0976, found: 151.0978.

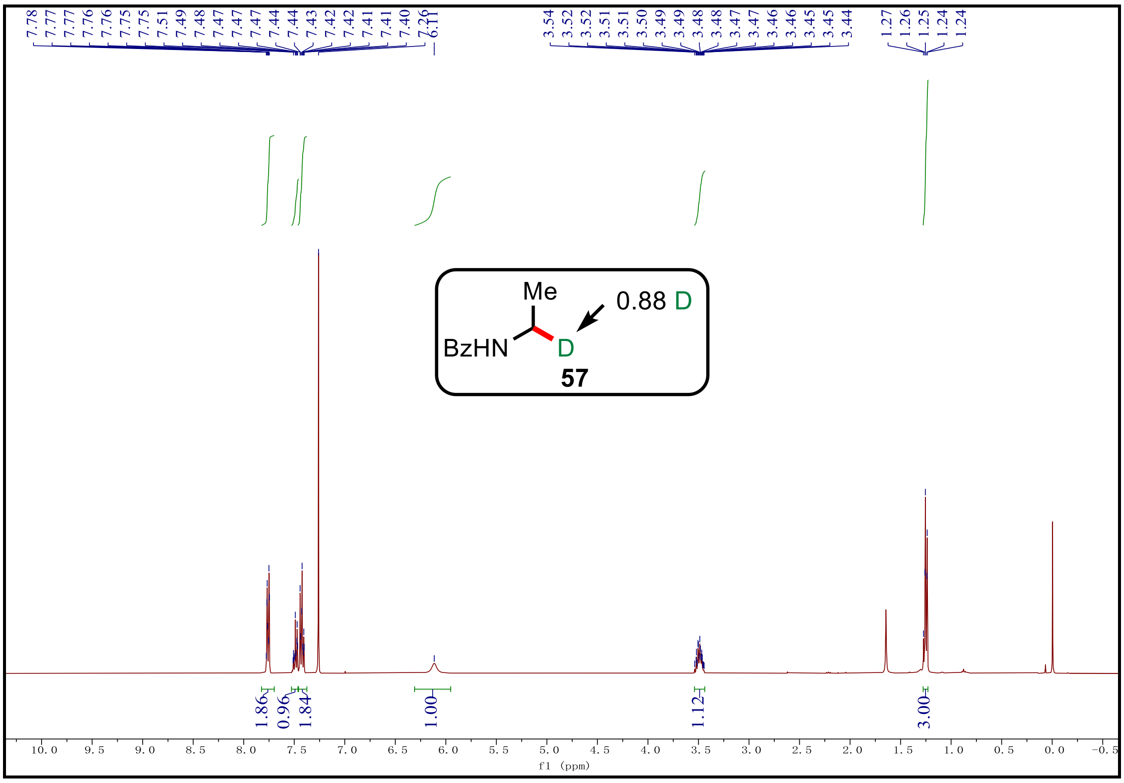

Supplementary Figure 5. ^{1}H NMR spectra of **57** in deuterium-labeling experiment without **2a**

Deuteroxide quenching experiment

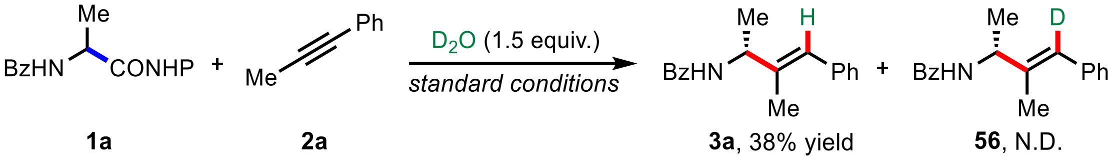

In glove box, $NiCl_2$•DME (0.01 mmol, 10 mol %), L6 (0.012 mmol, 12 mol%), $Ca(OAc)_2$ (0.30 mmol, 3.0 equiv.) and alkyl NHP ester **1a** (0.10 mmol, 1.0 equiv.) were combined in a 5 mL oven-dried sealing tube. The vessel was evacuated and backfilled with Ar (repeated for 3 times). Alkyne **2a** (0.20 mmol, 2.0 equiv.), $(MeO)_3SiH$ (0.60 mmol, 6.0 equiv.), D_2O (0.15 mmol, 1.5 equiv.) and NMP/THF (v/v = 2/1, 0.5 mL) were then added via syringe. The tube was sealed with a Teflon lined cap and stirred at 0 °C for 24 h. The subjection of D_2O into the standard conditions as a quenching reagent afforded only non-deuterated product **3a** (38% yield) but none of the deuterium-containing product **56**. As shown below, incorporation of deuterium was analyzed by ^{1}H NMR. For ^{1}H NMR, when deuterium is incorporated, the peak shape and integral of the corresponding position (alkenyl protons, δ 6.54 ppm) will change. Percent deuterium incorporation was derived from the integration

value of this signal.

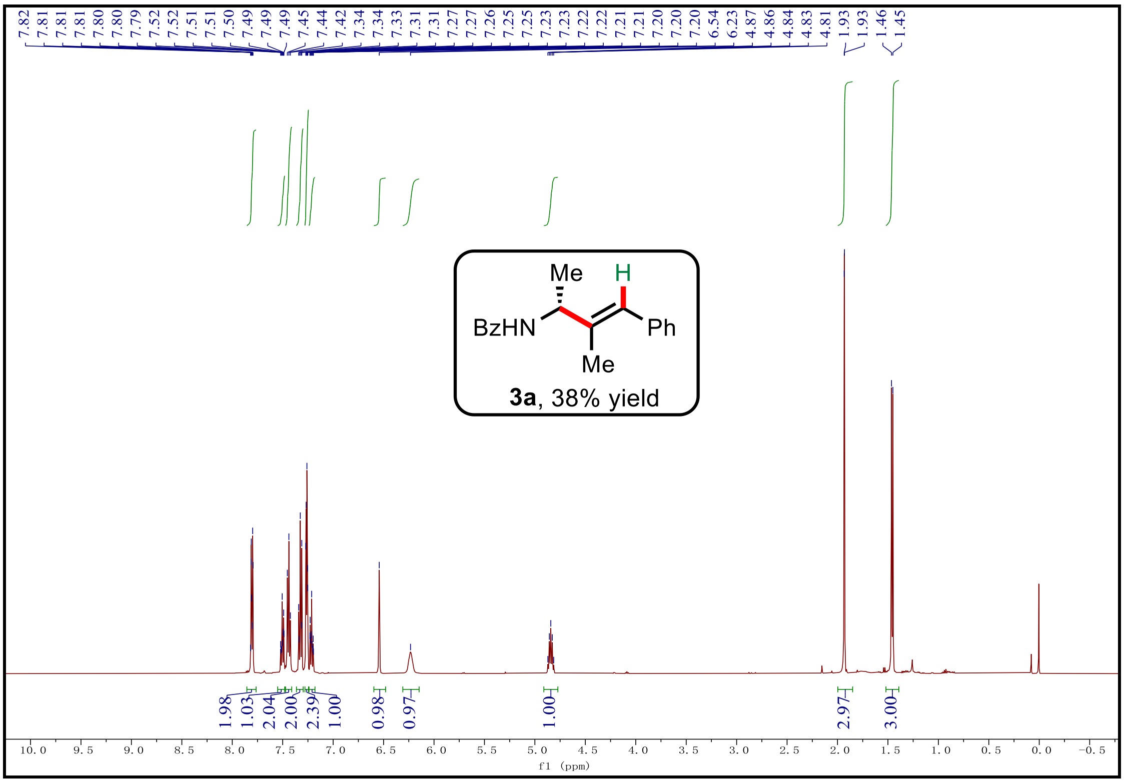

Supplementary Figure 6. ^{1}H NMR spectra of **3a** in deuteroxide quenching experiment

Radical Experiments.

Radical trapping experiment

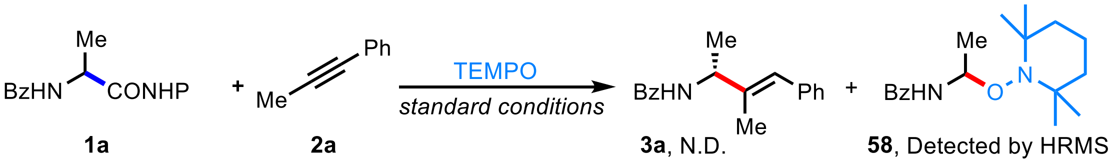

In glove box, $NiCl_2$•DME (0.01 mmol, 10 mol%), L6 (0.012 mmol, 12 mol%), $Ca(OAc)_2$ (0.30 mmol, 3.0 equiv.), alkyl NHP ester **1a** (0.10 mmol, 1.0 equiv.) and TEMPO (0.20 mmol, 2.0 equiv.) were combined in a 5 mL oven-dried sealing tube. The vessel was evacuated and backfilled with Ar (repeated for 3 times). Alkyne **2a** (0.20 mmol, 2.0 equiv.), $(MeO)_3SiH$ (0.60 mmol, 6.0 equiv.) and NMP/THF (v/v = 2/1, 0.5 mL) were then added via syringe. The tube was sealed with a Teflon lined cap and stirred at 0 °C for 24 h. The addition of radical inhibitor TEMPO shut down the productive reactivity and no product was obtained. And the TEMPO-captured product **58** was detected by HRMS-ESI. HRMS (ESI): m/z calcd. for $C_{18}H_{28}N_2O_2H^+$ [M + H^+]: 305.2224, found: 305.2200.

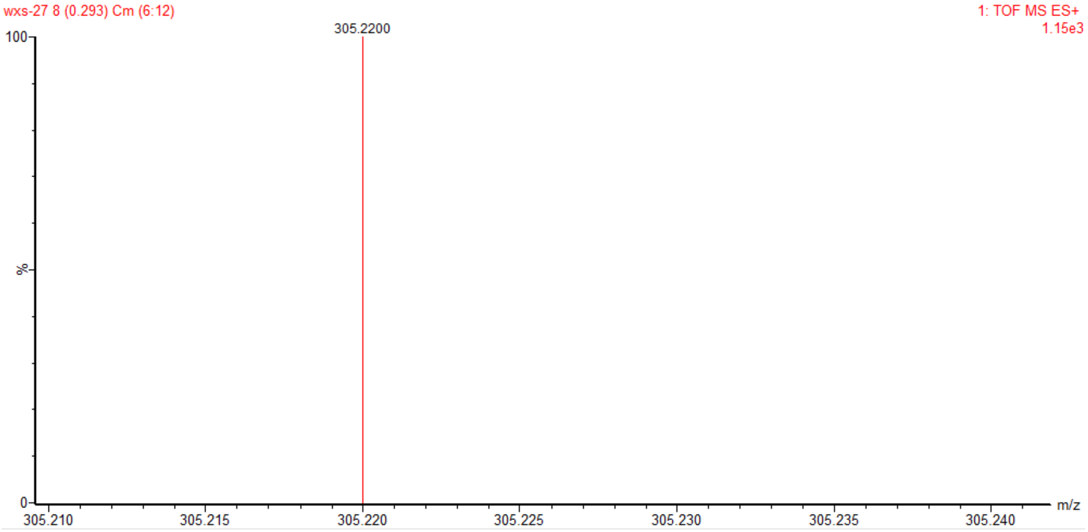

Supplementary Figure 7. Local HRMS spectra of radical inhibition reaction

Competition experiment

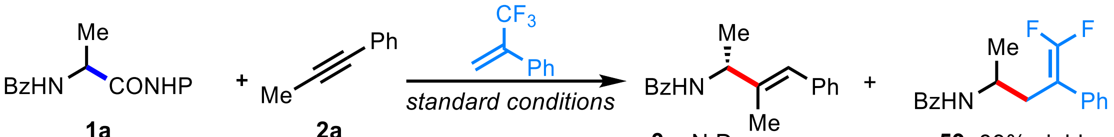

In glove box, $NiCl_2$•DME (0.01 mmol, 10 mol %), L6 (0.012 mmol, 12 mol%), $Ca(OAc)_2$ (0.30 mmol, 3.0 equiv.) and alkyl NHP ester **1a** (0.10 mmol, 1.0 equiv.) were combined in a 5 mL oven-dried sealing tube. The vessel was evacuated and backfilled with Ar (repeated for 3 times). Alkyne **2a** (0.20 mmol, 2.0 equiv.), $(MeO)_3SiH$ (0.60 mmol, 6.0 equiv.), (3,3,3-trifluoroprop-1-en-2-yl) benzene (0.20 mmol, 2.0 equiv.) and NMP/THF (v/v = 2/1, 0.5 mL) were then added via syringe. The tube was sealed with a Teflon lined cap and stirred at 0 ºC for 24 h. As shown above, in the presence of an electrondeficient olefin, a racemic adduct **59** was generated in 33% yield, indicating the intermediacy of α-amino radicals.

(S)-N-(5,5-difluoro-4-phenylpent-4-en-2-yl)benzamide

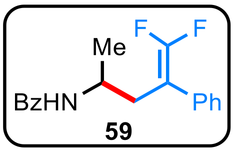

The product **59** was purified with silica gel chromatography (PE/EA = 10:1) as a colorless oil (33%). ^{1}H NMR (400 MHz, Chloroform-*d*) δ 7.49 – 7.42 (m, 3H), 7.40 – 7.31 (m, 6H), 7.29 – 7.24 (m, 1H), 5.84 (s, 1H), 4.36 – 4.21 (m, 1H), 2.82 – 2.71 (m, 1H), 2.70 – 2.60 (m, 1H), 1.24 (d, *J* = 6.7 Hz, 3H). ^{19}F NMR (376 MHz, Chloroform-*d*) δ -90.38. ^{13}C NMR (101 MHz, Chloroform-*d*) δ 166.77, 154.59 (t, *J* = 290.5 Hz), 134.59, 133.51, 131.44, 128.92, 128.53, 128.44 (t, *J* = 3.1 Hz), 127.71, 126.79, 89.86 (t, *J* = 18.0 Hz), 44.96 (t, *J* = 2.8 Hz), 34.50, 20.20. HRMS (ESI): m/z calcd. for $C_{18}H_{17}F_2NONa^+$ [M + Na^+]: 324.1170, found: 324.1175.

Radical clock experiment

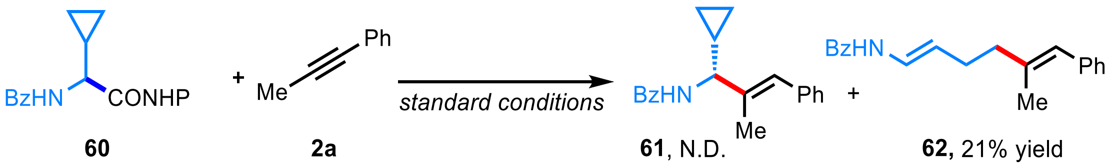

60 **2a** **61**, N.D. **62**, 21% yield

In glove box, $NiCl_2$•DME (0.01 mmol, 10 mol%), L6 (0.012 mmol, 12 mol%), $Ca(OAc)_2$ (0.30 mmol, 3.0 equiv.) and alkyl NHP ester **60** (0.10 mmol, 1.0 equiv.) were combined in a 5 mL oven-dried sealing tube. The vessel was evacuated and backfilled with Ar (repeated for 3 times). Alkyne **2a** (0.20 mmol, 2.0 equiv.), $(MeO)_3SiH$ (0.60 mmol, 6.0 equiv.) and NMP/THF (v/v = 2/1, 0.5 mL) were then added via syringe. The tube was sealed with a Teflon lined cap and stirred at 0 ºC for 24 h. As shown above, a "radical clock" alkyl NHP ester **60** was prepared to test the reaction, the ring-opened product **62** was obtained. It was revealed that the activation of alkyl NHP ester proceeded through a radical pathway.

N-((1E,5E)-5-methyl-6-phenylhexa-1,5-dien-1-yl)benzamide

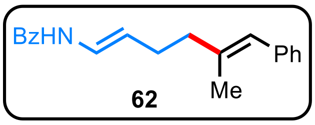

62

The product **62** was purified with silica gel chromatography (PE/EA = 10:1) as a colorless oil (21%). ^{1}H NMR (400 MHz, Chloroform-*d*) δ 7.83 – 7.70 (m, 2H), 7.62 (d, *J* = 10.6 Hz, 1H), 7.59 – 7.50 (m, 1H), 7.53 – 7.41 (m, 2H), 7.36 – 7.29 (m, 2 H), 7.25 – 7.14 (m, 3H), 7.09 – 6.94 (m, 1H), 6.29 (s, 1H), 5.40 – 5.27 (m, 1H), 2.38 – 2.06 (m, 4H), 1.88 (d, *J* = 1.4 Hz, 3H). ^{13}C NMR (126 MHz, Chloroform-*d*) δ 164.38, 138.53, 138.21, 133.96, 132.00, 128.98, 128.86, 128.19, 127.13, 126.09, 125.67, 123.25, 113.43, 41.20, 28.53, 17.90. HRMS (ESI): m/z calcd. for $C_{20}H_{21}NONa^+$ [M + Na$^+$]: 314.1515, found: 314.1522.

Control Experiments.

Supplementary Table 8. Table of control experiments

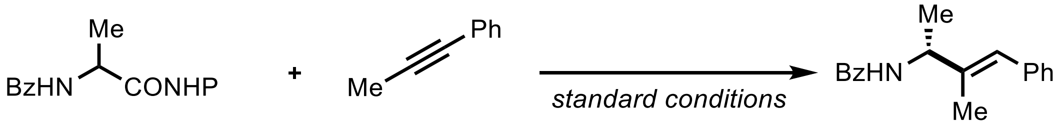

1a **2a** **3a**

Entry	Reaction conditions	Yield	*ee*
1	10 mol% $NiCl_2$•DME, w/o [Si]-H	N.D.	-
2	10 mol% $NiCl_2$•DME, in the dark	70%	97%
3	10 mol% $Ni(COD)_2$ instead of $NiCl_2$•DME, with or w/o [Si]-H	N.D.	-
4	100 mol% $Ni(COD)_2$ instead of $NiCl_2$•DME	N.D.	-
5	10 mol% $Ni(COD)_2$ and 10 mol% $NiCl_2$•DME	52%	97%
6	50 mol% $Ni(COD)_2$ and 50 mol% $NiCl_2$•DME	38%	97%

For entry 1, in glove box, $NiCl_2$•DME (0.01 mmol, 10 mol%), L6 (0.012 mmol, 12 mol%), $Ca(OAc)_2$ (0.3 mmol, 3.0 equiv.) and alkyl NHP ester **1a** (0.10 mmol, 1.0 equiv.) were combined in a 5 mL oven-dried sealing tube. The vessel was evacuated and backfilled with Ar (repeated for 3 times). Alkyne **2a** (0.20 mmol, 2.0 equiv.) and NMP/THF (v/v = 2/1, 0.5 mL) were then added via syringe. The tube was sealed with a Teflon lined cap and stirred at 0 ˚C for 24 h. The result was showed in the Supplementary Table 8.

For entry 2, in glove box, $NiCl_2$•DME (0.01 mmol, 10 mol%), L6 (0.012 mmol, 12 mol%), $Ca(OAc)_2$ (0.3 mmol, 3.0 equiv.) and alkyl NHP ester **1a** (0.10 mmol, 1.0 equiv.) were combined in a 5 mL oven-dried sealing tube. The vessel was evacuated and backfilled with Ar (repeated for 3 times). Alkyne **2a** (0.20 mmol, 2.0 equiv.), $(MeO)_3SiH$ (0.60 mmol, 6.0 equiv.) and NMP/THF (v/v = 2/1, 0.5 mL) were then added via syringe. The tube was sealed with a Teflon lined cap and stirred at 0 ˚C in the dark for 24 h. The result was showed in the Supplementary Table 8.

For entry 3, in glove box, $Ni(COD)_2$ (0.01 mmol, 10 mol%), L6 (0.012 mmol, 12 mol%), $Ca(OAc)_2$ (0.3 mmol, 3.0 equiv.) and alkyl NHP ester **1a** (0.10 mmol, 1.0 equiv.) were combined in a 5 mL oven-dried sealing tube. The vessel was evacuated and backfilled with Ar (repeated for 3 times). Alkyne **2a** (0.20 mmol, 2.0 equiv.), $(MeO)_3SiH$ (0.60 mmol, 6.0 equiv.) (or not) and NMP/THF (v/v = 2/1, 0.5 mL) were then added via syringe. The tube was sealed with a Teflon lined cap and stirred at 0 ˚C for 24 h. The results were showed in the Supplementary Table 8.

For entry 4-6, in glove box, Ni Source, L6, $Ca(OAc)_2$ (0.3 mmol, 3.0 equiv.) and alkyl NHP ester **1a** (0.10 mmol, 1.0 equiv.) were combined in a 5 mL oven-dried sealing tube. The vessel was evacuated and backfilled with Ar (repeated for 3 times). Alkyne **2a** (0.20 mmol, 2.0 equiv.), $(MeO)_3SiH$ (0.60 mmol, 6.0 equiv.) and NMP/THF (v/v = 2/1, 0.5 mL) were then added via syringe. The tube was sealed with a Teflon lined cap and stirred at 0 ˚C for 24 h. The results were showed in the Supplementary Table 8.

X-ray Crystallographic Data of 28

Compound 28: (The crystal structure of compound **28** has been deposited at the Cambridge Crystallographic Data Centre (**CCDC 2308329**). Copies of the data can be obtained free of charge via www.ccdc.cam.ac.uk/data_request/cif.

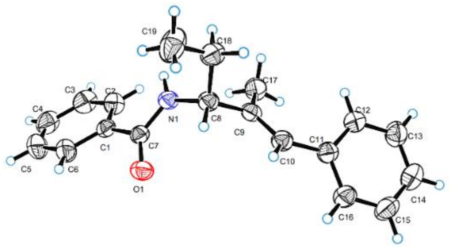

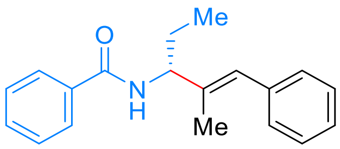

Supplementary Table 9. Crystal data and structure refinement for compound **28**.

Identification code	GQ-03092_auto
Empirical formula	C19H21NO
Formula weight	279.37
Temperature/K	293(2)
Crystal system	monoclinic
Space group	P21
a/Å	10.29262(11)
b/Å	10.09721(9)
c/Å	15.51835(16)
α /°	90
β /°	90.0650(10)
γ /°	90
Volume/Å3	1612.77(3)
Z	4
ρ calcg/cm3	1.151
μ /mm-1	0.545
F(000)	600.0
Crystal size/mm3	0.26 × 0.22 × 0.21
Radiation	Cu K α (λ = 1.54184)
2 Θ range for data collection/°	8.59 to 145.89
Index ranges	-11 ≤ h ≤ 12, -12 ≤ k ≤ 12, -18 ≤ l ≤ 18
Reflections collected	12182
Independent reflections	5997 [Rint = 0.0264, Rsigma = 0.0269]
Data/restraints/parameters	5997/1/388
Goodness-of-fit on F2	1.040
Final R indexes [I>=2 σ (I)]	R1 = 0.0335, wR2 = 0.0963
Final R indexes [all data]	R1 = 0.0366, wR2 = 0.1000
Largest diff. peak/hole / e Å-3	0.10/-0.11
Flack parameter	0.00(11)

Supplementary Figures

NMR Spectra of New Compounds

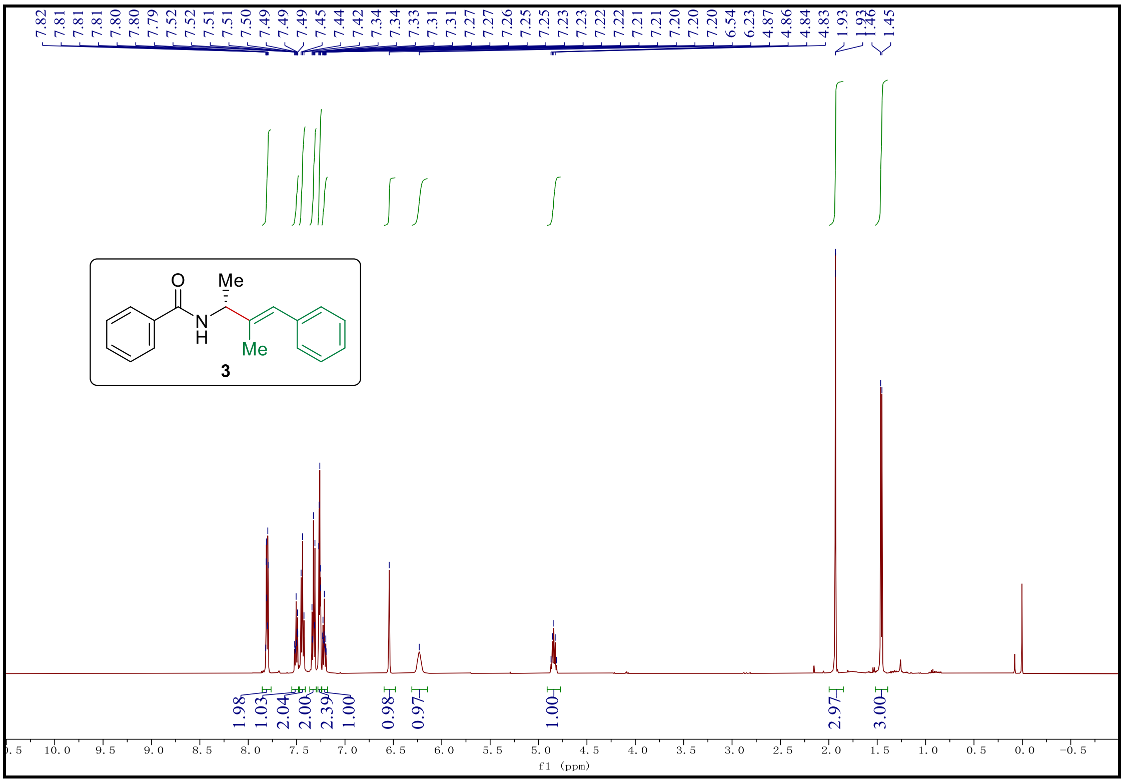

Supplementary Figure 8. ^{1}H NMR Spectrum of Compound **3** (500 MHz, $CDCl_3$, 25 ˚C)

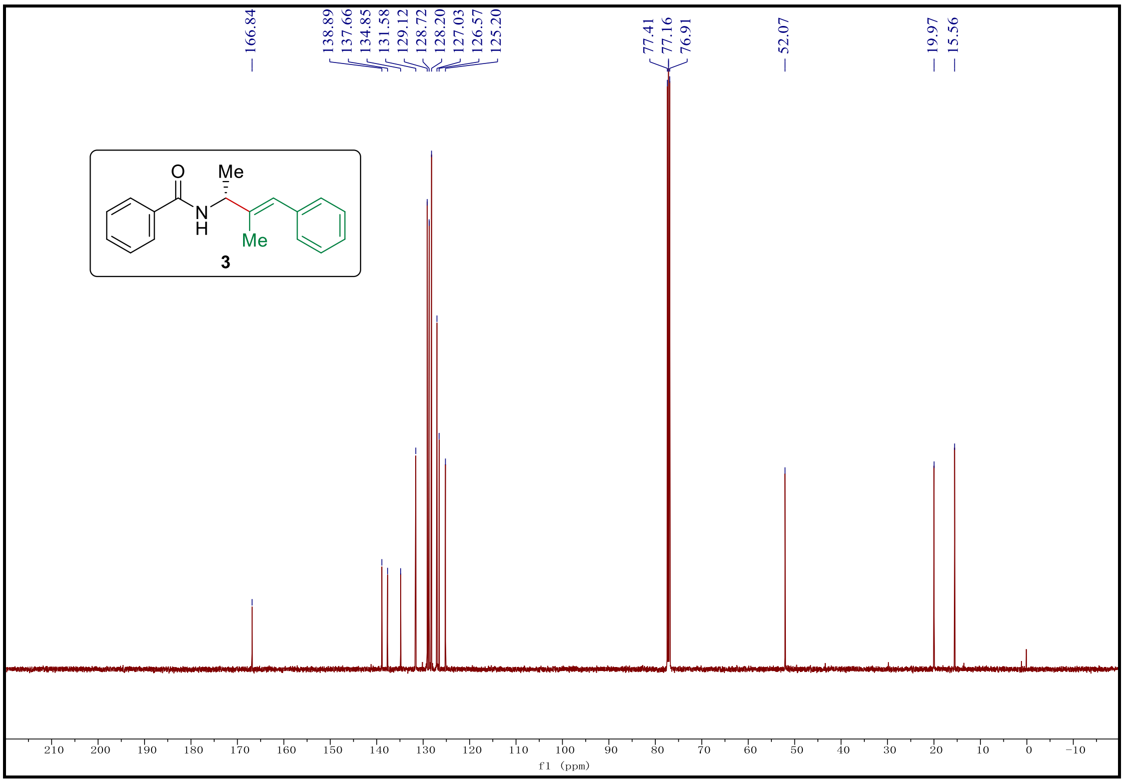

Supplementary Figure 9. ^{13}C NMR Spectrum of Compound **3** (126 MHz, $CDCl_3$, 25 ˚C)

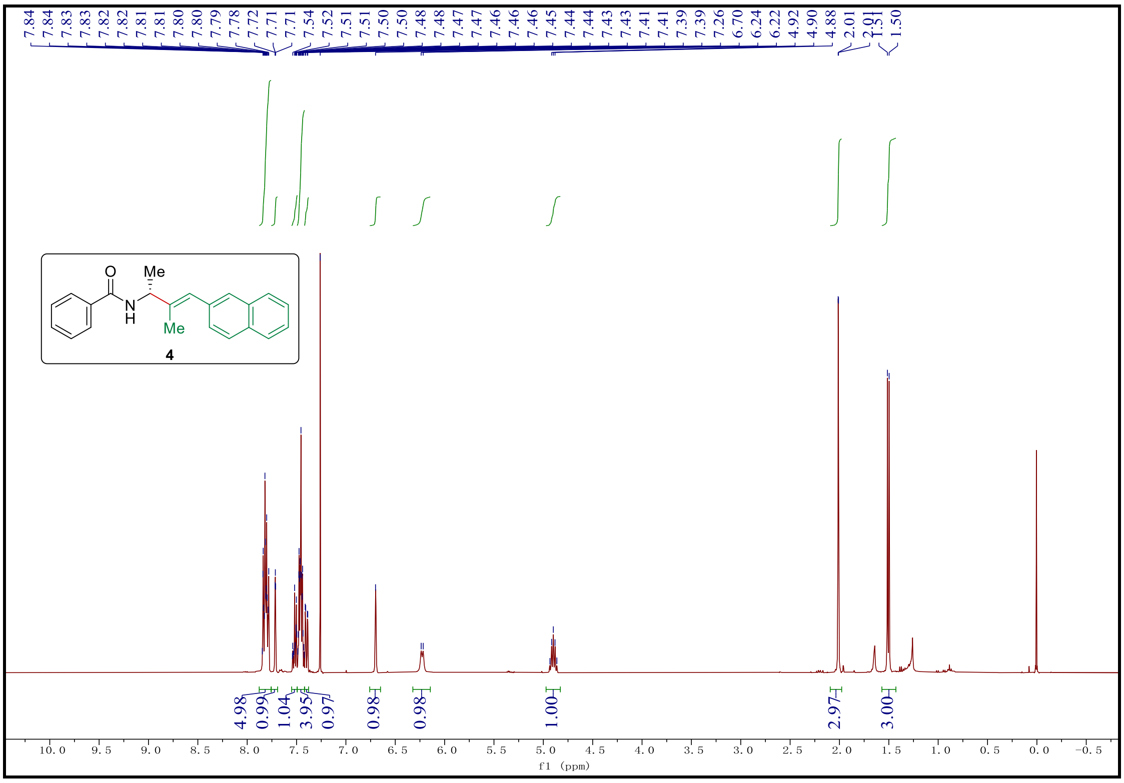

Supplementary Figure 10. ^{1}H NMR Spectrum of Compound **4** (400 MHz, $CDCl_3$, 25 ˚C)

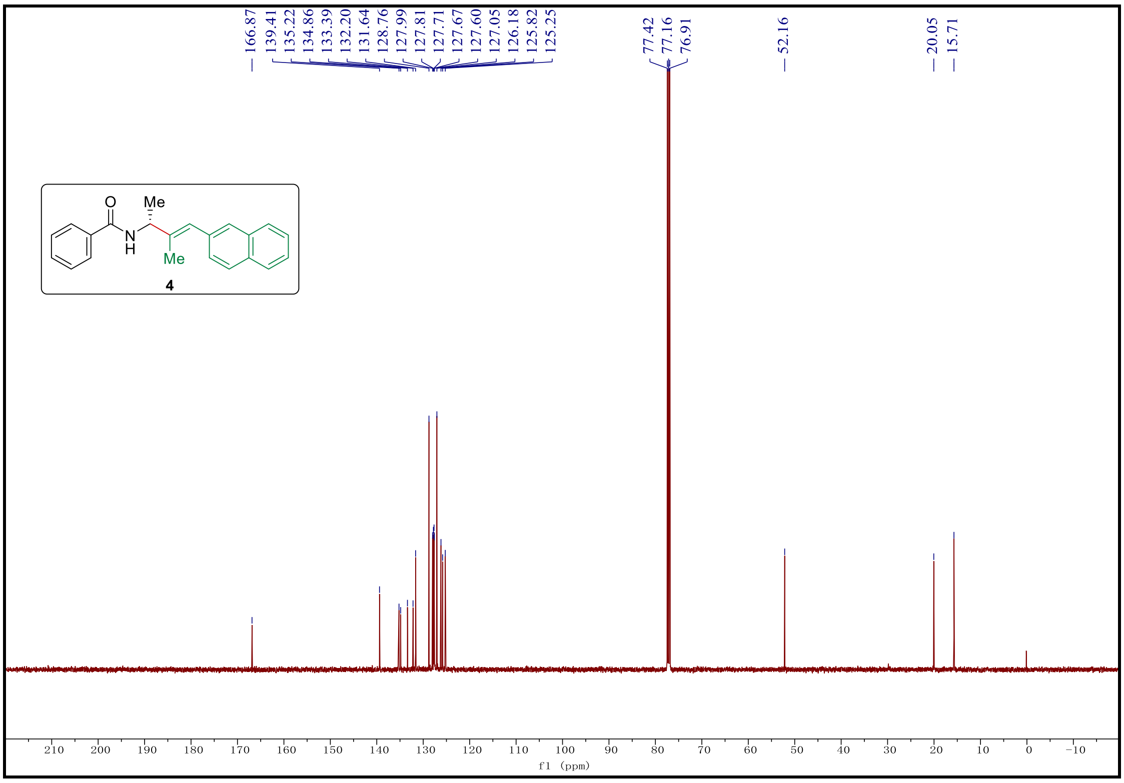

Supplementary Figure 11. ^{13}C NMR Spectrum of Compound **4** (126 MHz, $CDCl_3$, 25 ˚C)

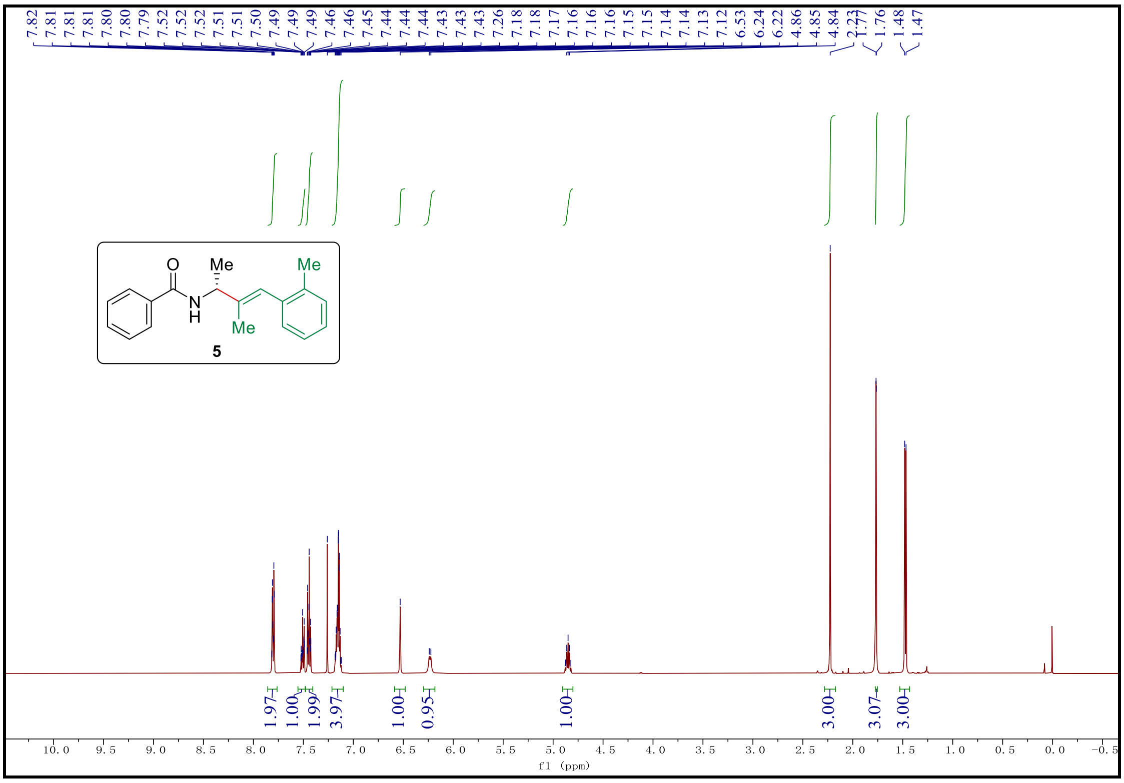

Supplementary Figure 12. ^{1}H NMR Spectrum of Compound **5** (500 MHz, $CDCl_3$, 25 ˚C)

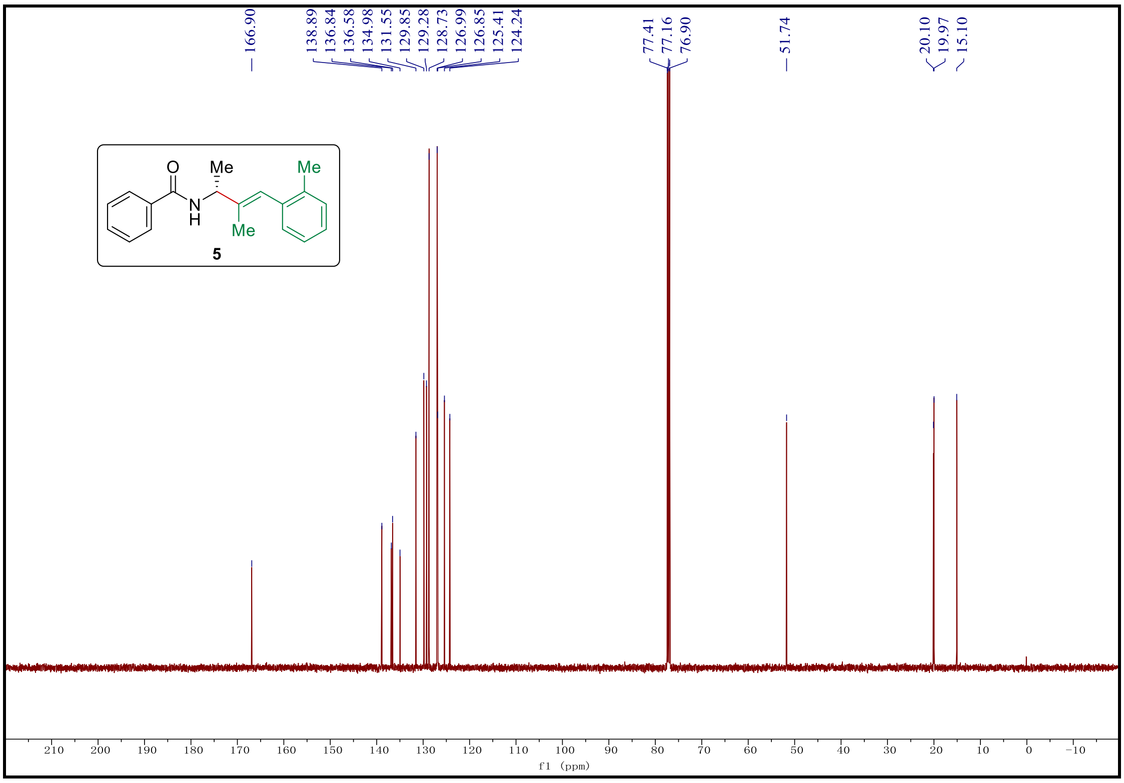

Supplementary Figure 13. ^{13}C NMR Spectrum of Compound **5** (126 MHz, $CDCl_3$, 25 ˚C)

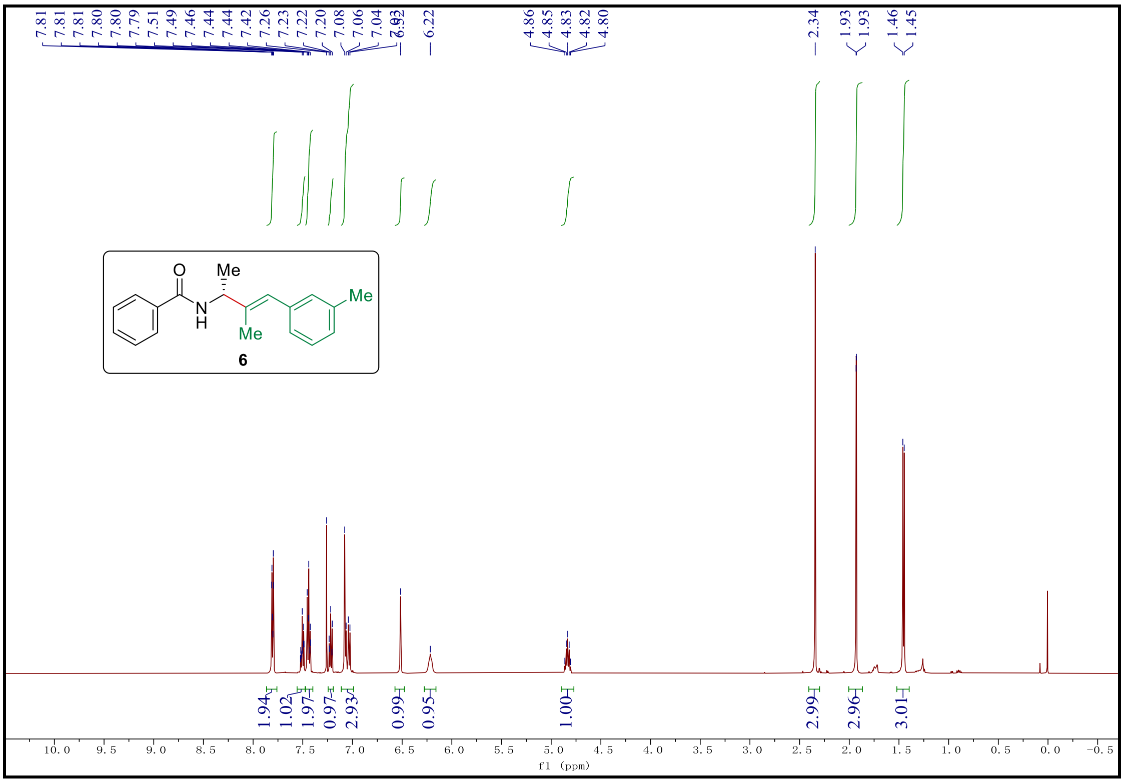

Supplementary Figure 14. ^{1}H NMR Spectrum of Compound **6** (500 MHz, $CDCl_3$, 25 ˚C)

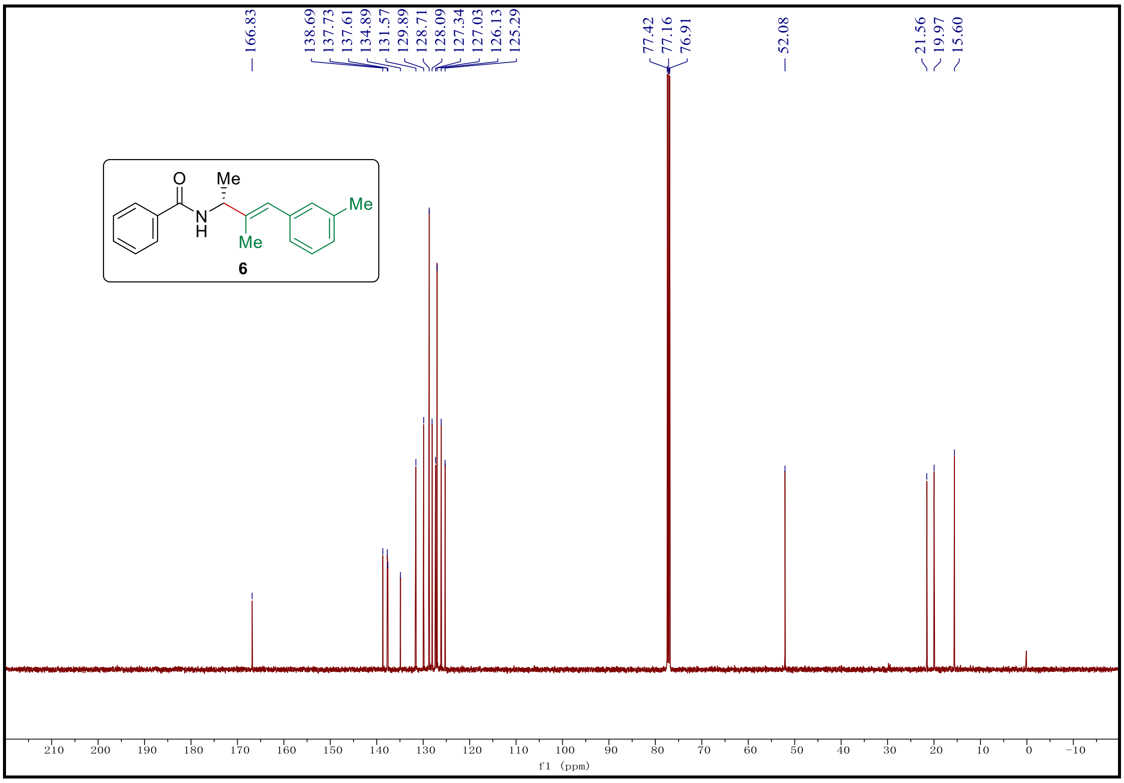

Supplementary Figure 15. ^{13}C NMR Spectrum of Compound **6** (126 MHz, $CDCl_3$, 25 ˚C)

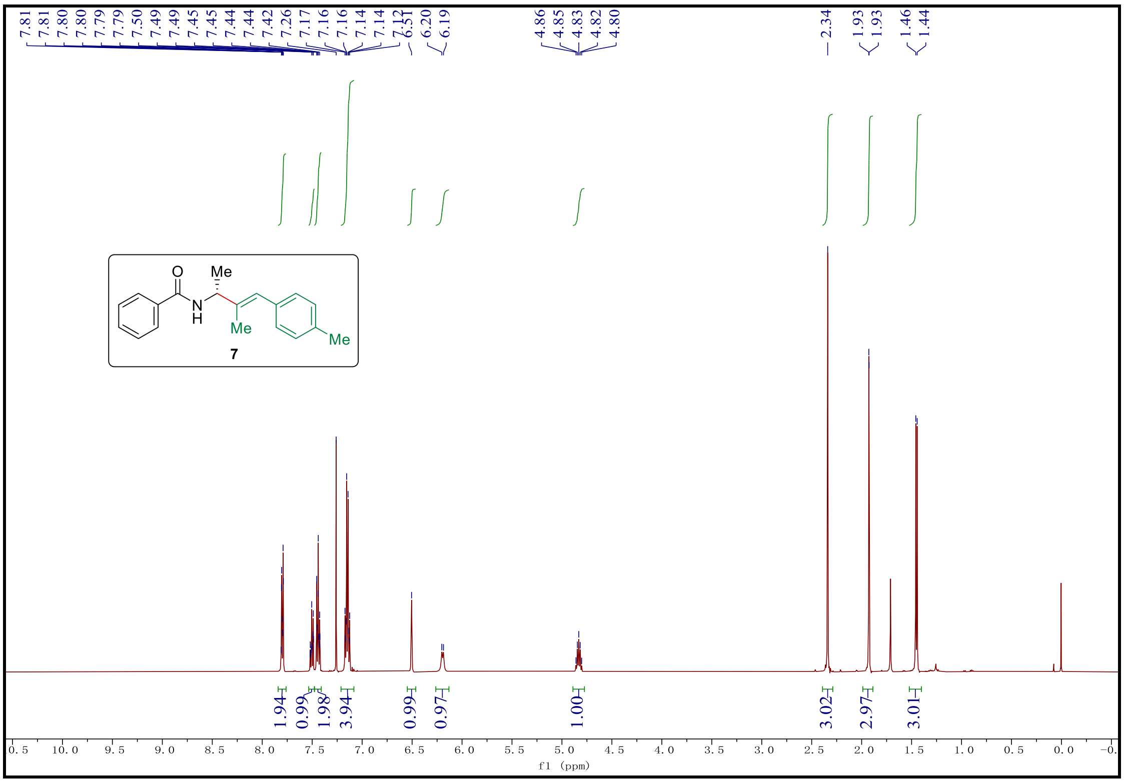

Supplementary Figure 16. ^{1}H NMR Spectrum of Compound **7** (500 MHz, $CDCl_3$, 25 ˚C)

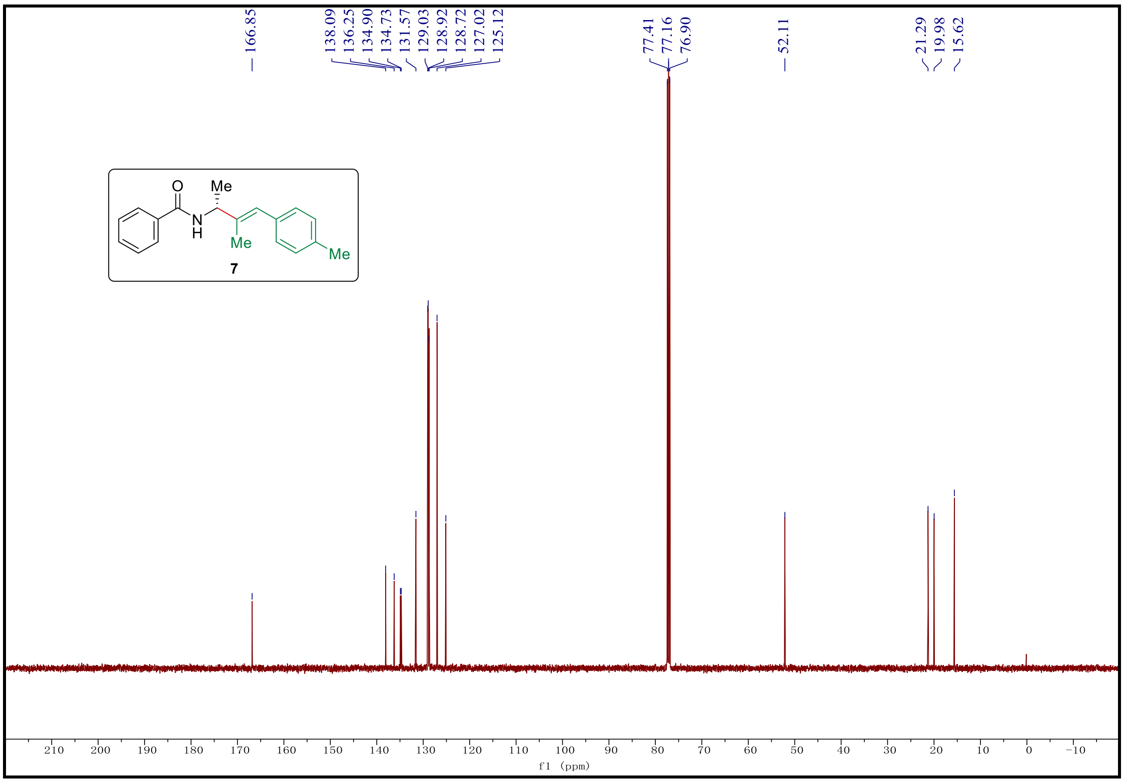

Supplementary Figure 17. ^{13}C NMR Spectrum of Compound **7** (126 MHz, $CDCl_3$, 25 ˚C)

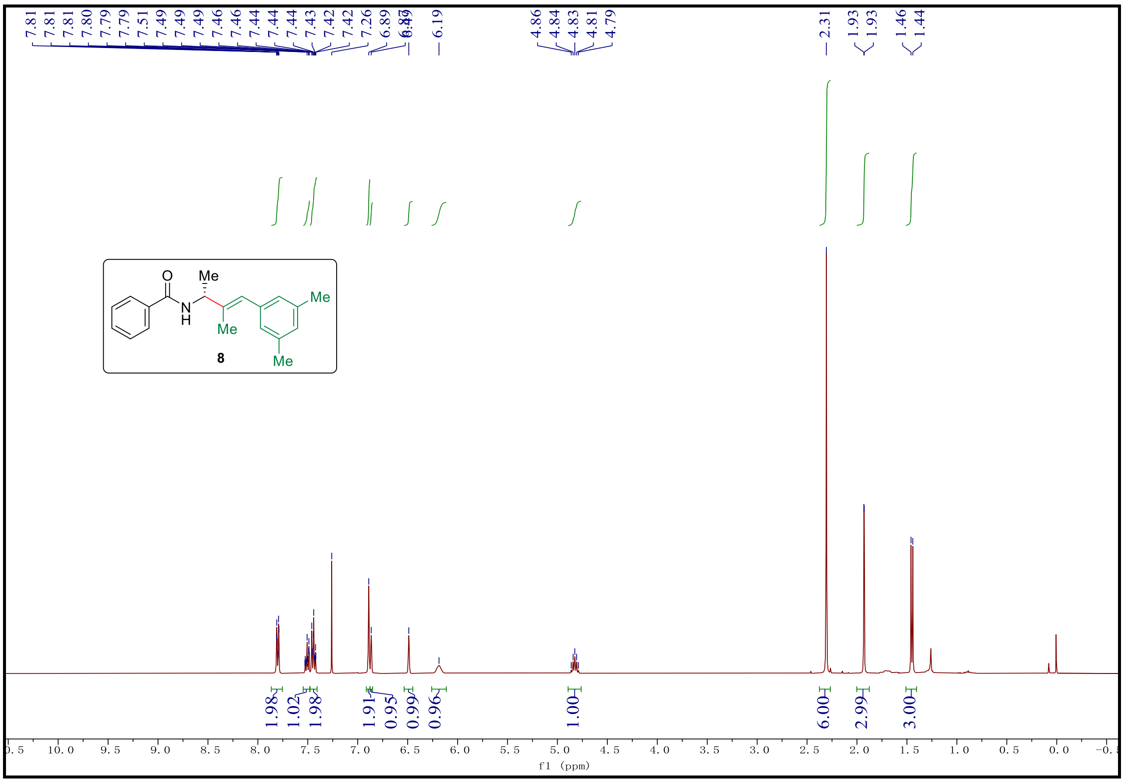

Supplementary Figure 18. ^{1}H NMR Spectrum of Compound **8** (400 MHz, $CDCl_3$, 25 ˚C)

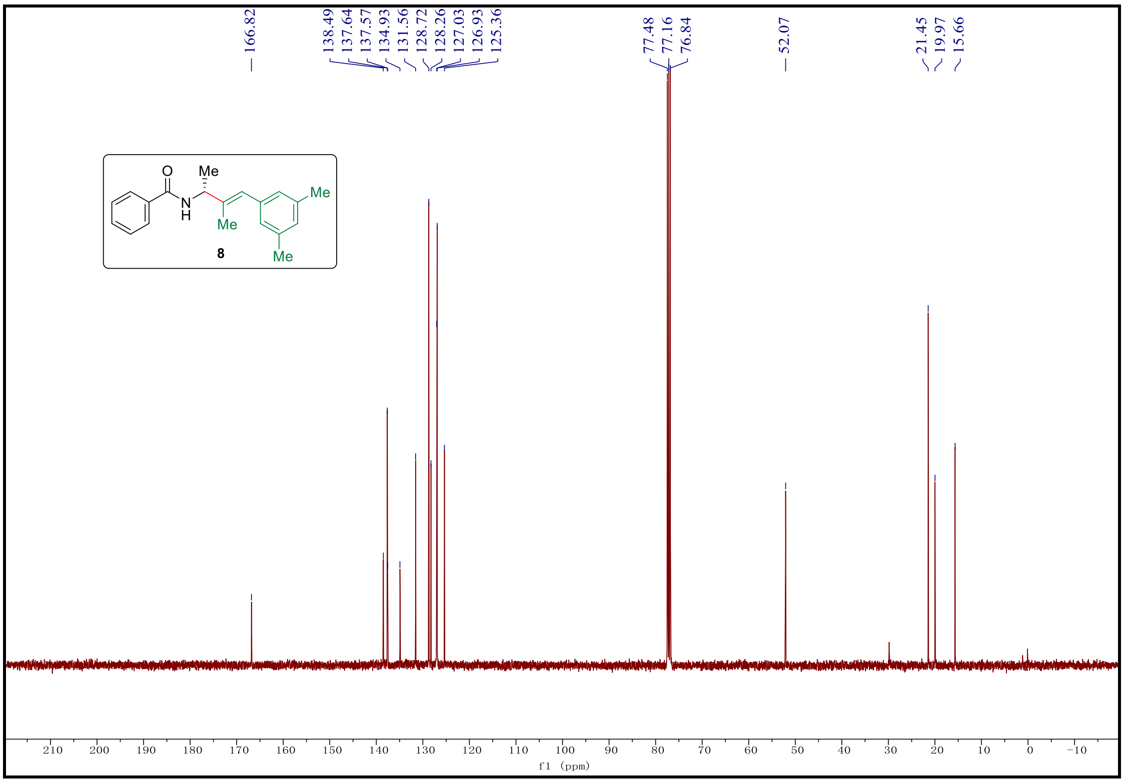

Supplementary Figure 19. ^{13}C NMR Spectrum of Compound **8** (101 MHz, $CDCl_3$, 25 ˚C)

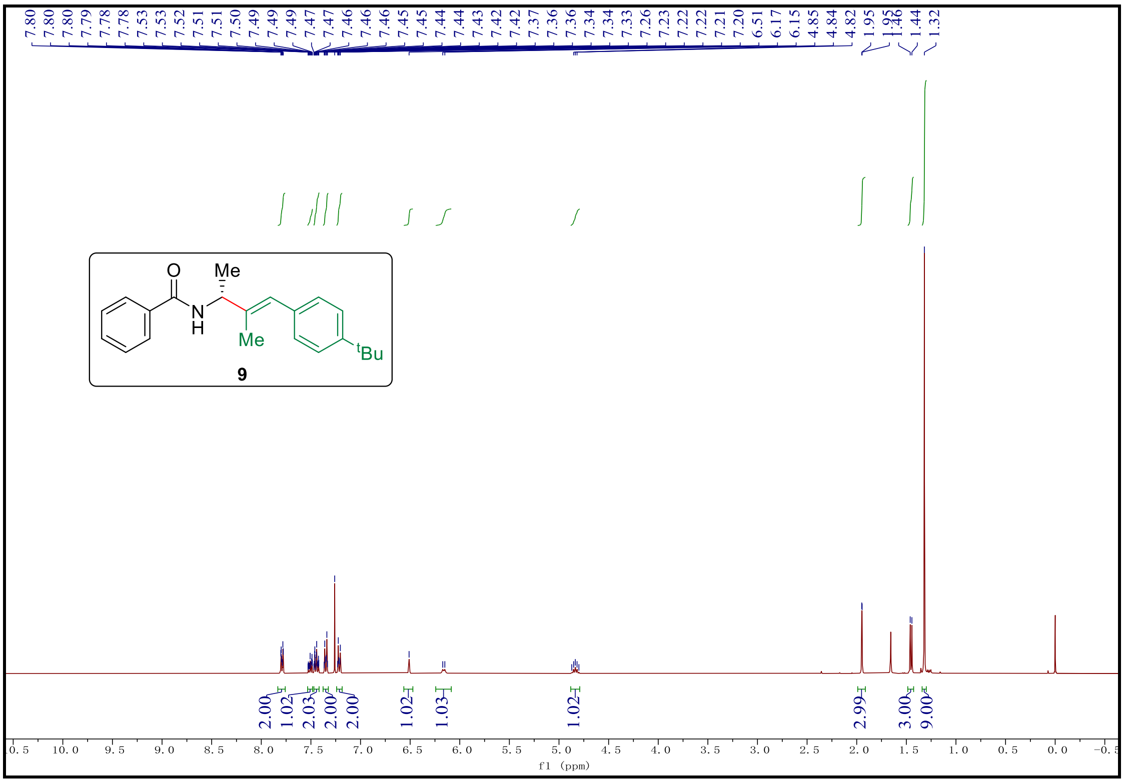

Supplementary Figure 20. ^{1}H NMR Spectrum of Compound **9** (400 MHz, $CDCl_3$, 25 °C)

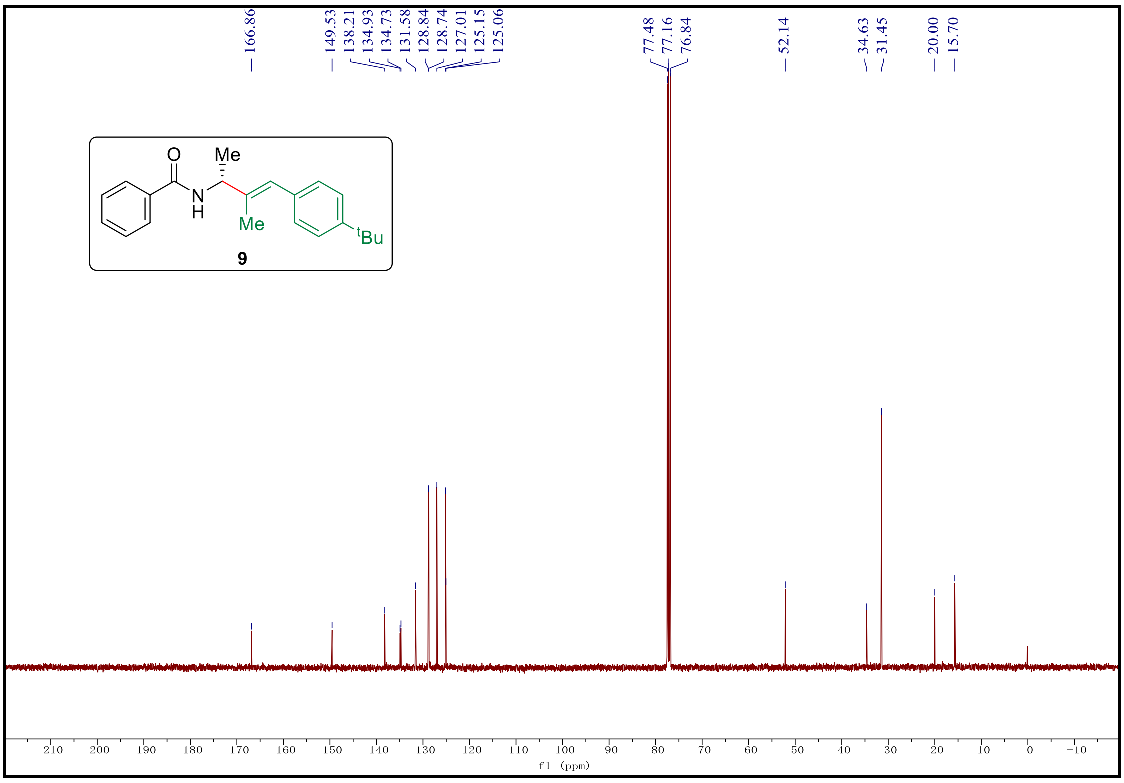

Supplementary Figure 21. ^{13}C NMR Spectrum of Compound **9** (101 MHz, $CDCl_3$, 25 °C)

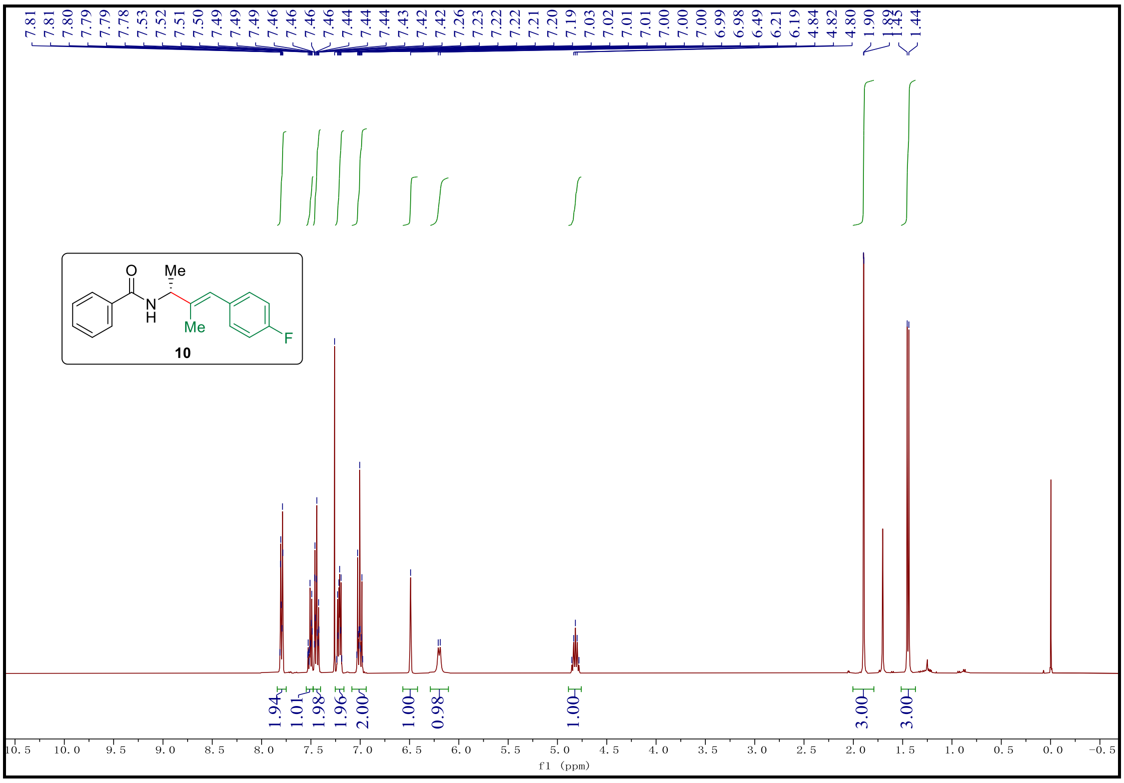

Supplementary Figure 22. ^{1}H NMR Spectrum of Compound **10** (400 MHz, $CDCl_3$, 25 ˚C)

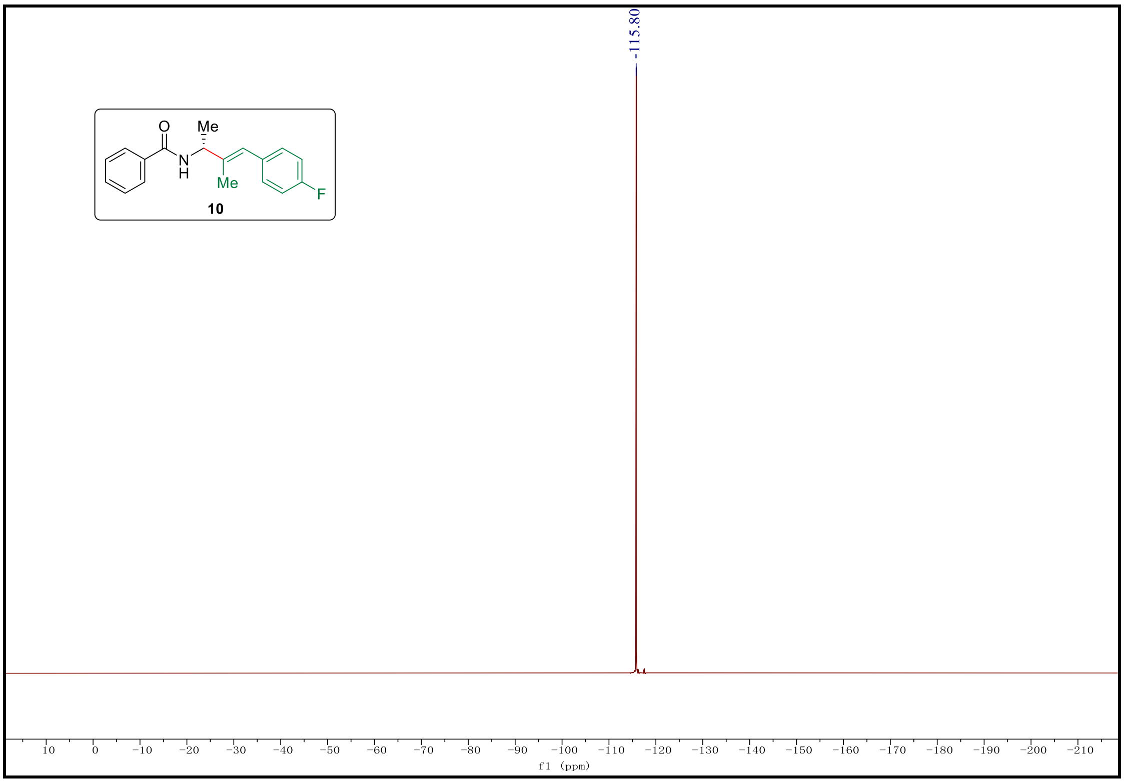

Supplementary Figure 23. ^{19}F NMR Spectrum of Compound **10** (376 MHz, $CDCl_3$, 25 ˚C)

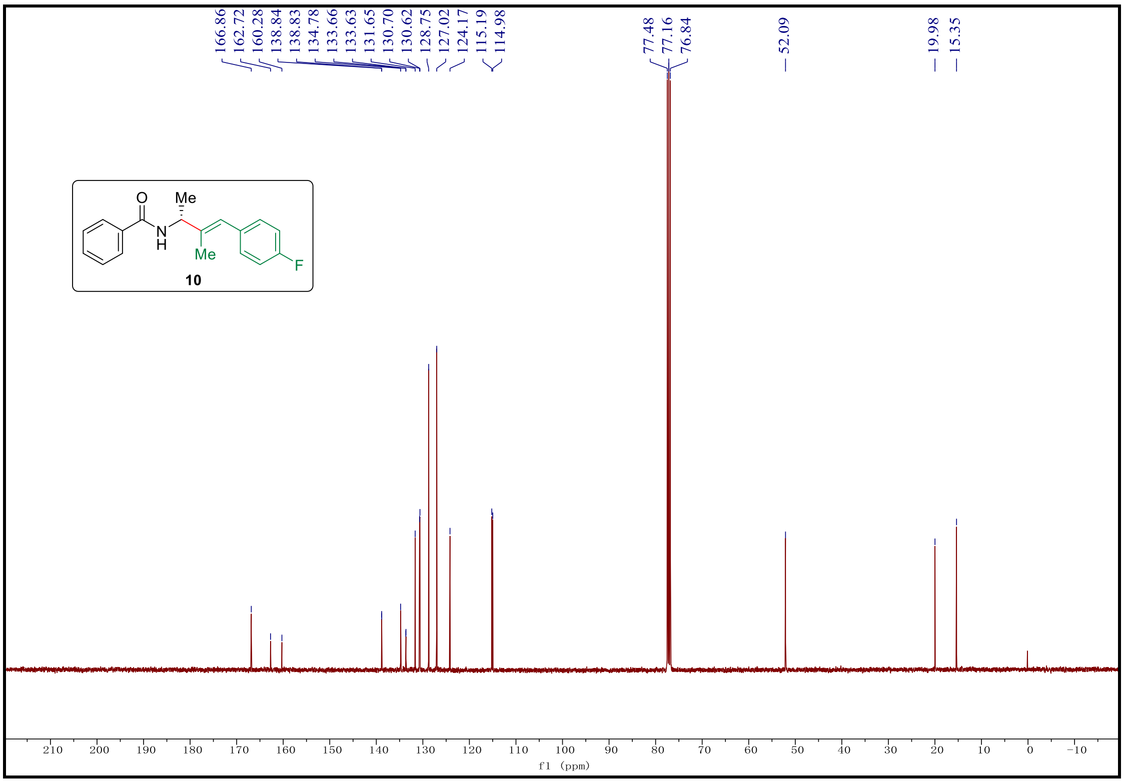

Supplementary Figure 24. ^{13}C NMR Spectrum of Compound **10** (101 MHz, $CDCl_3$, 25 ˚C)

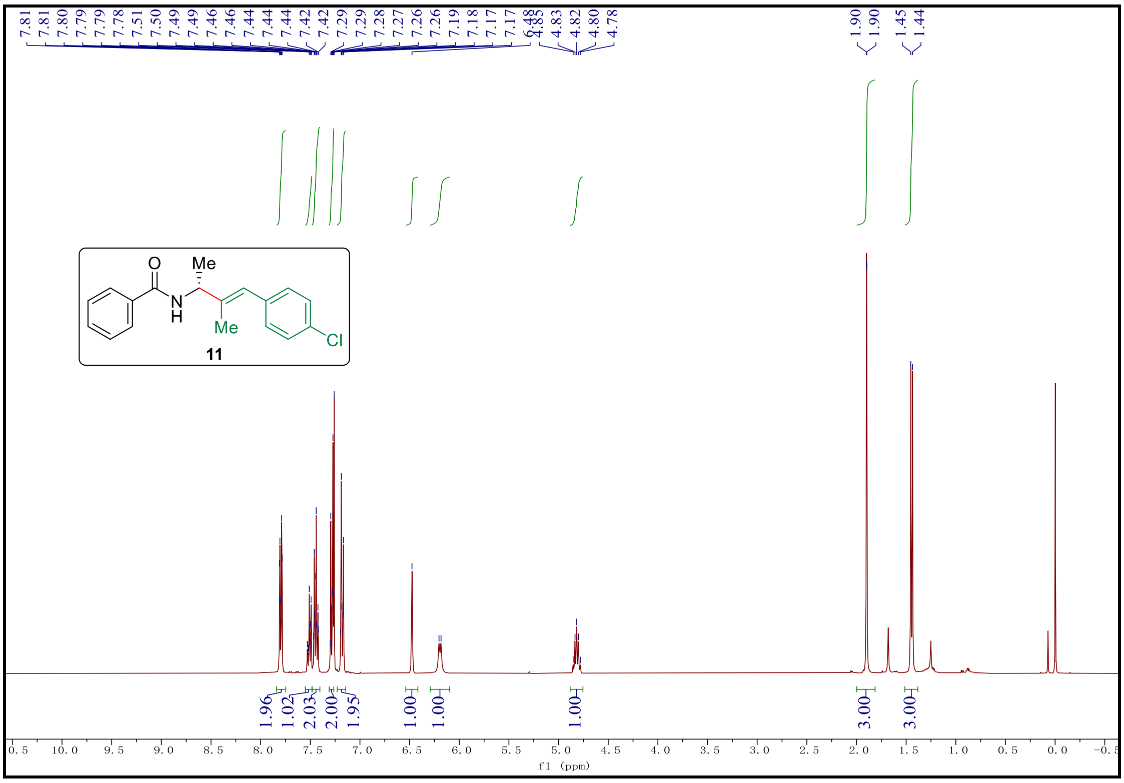

Supplementary Figure 25. ^{1}H NMR Spectrum of Compound **11** (400 MHz, $CDCl_3$, 25 ˚C)

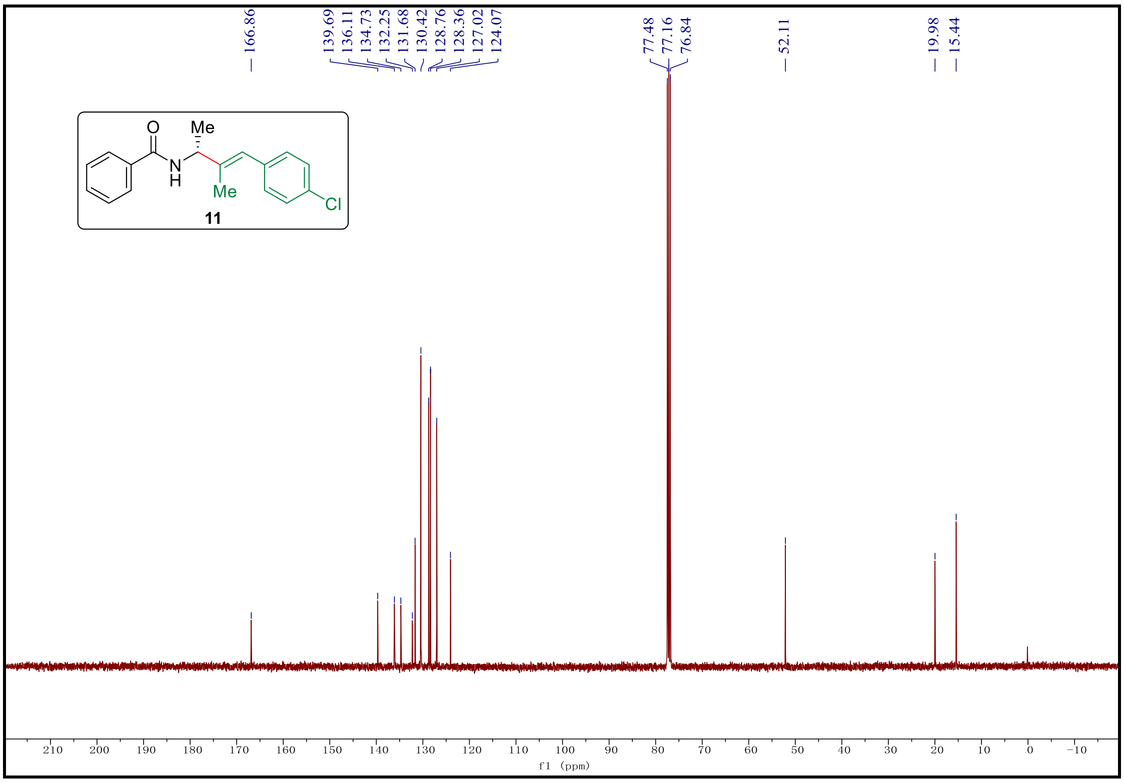

Supplementary Figure 26. ^{13}C NMR Spectrum of Compound **11** (101 MHz, $CDCl_3$, 25 ˚C)

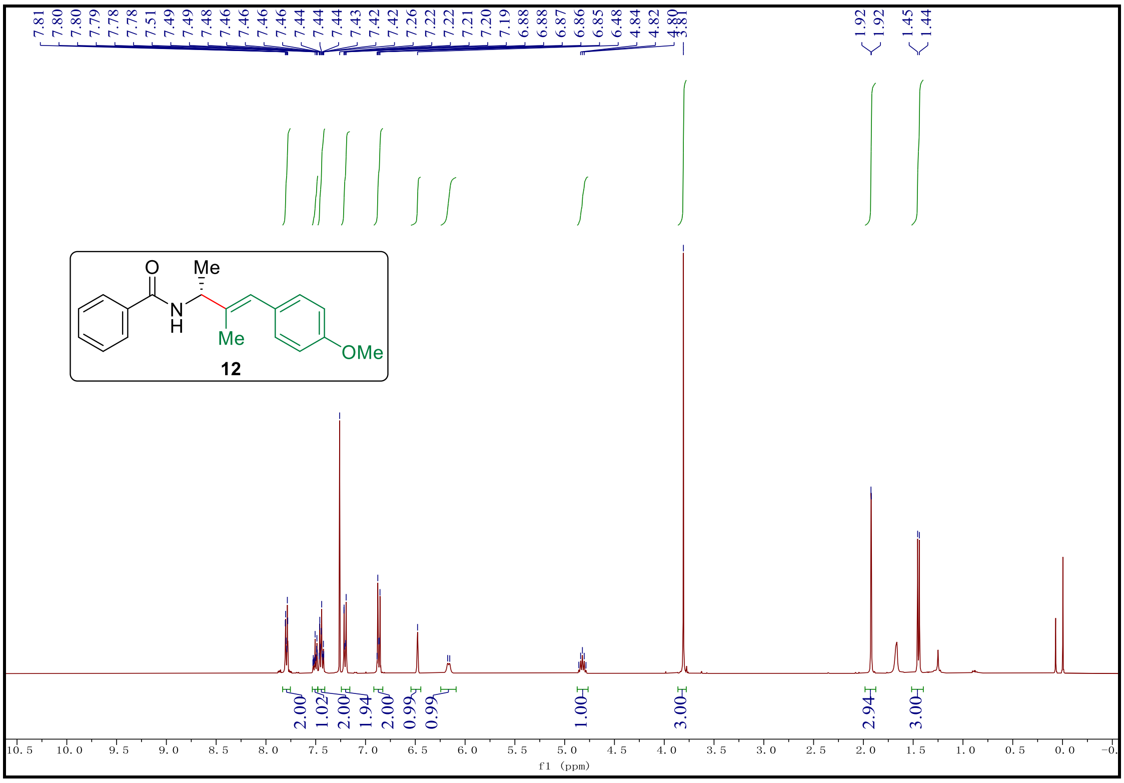

Supplementary Figure 27. ^{1}H NMR Spectrum of Compound **12** (400 MHz, $CDCl_3$, 25 ˚C)

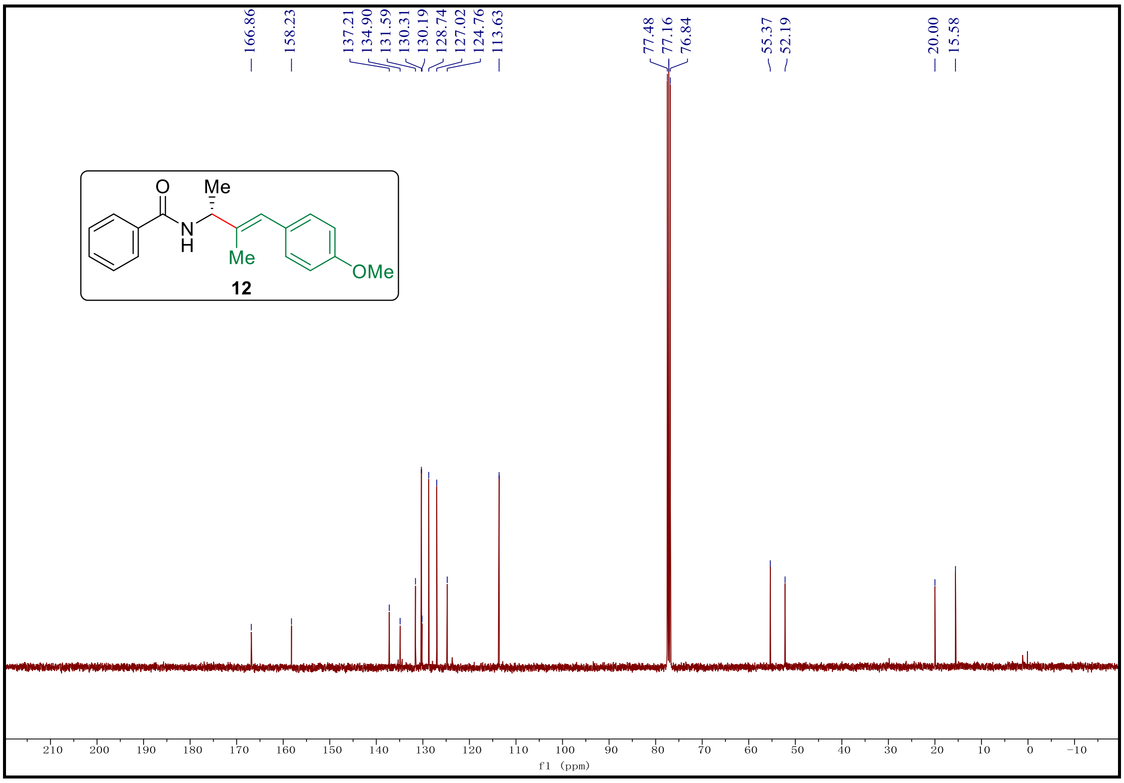

Supplementary Figure 28. ^{13}C NMR Spectrum of Compound **12** (101 MHz, $CDCl_3$, 25 ˚C)

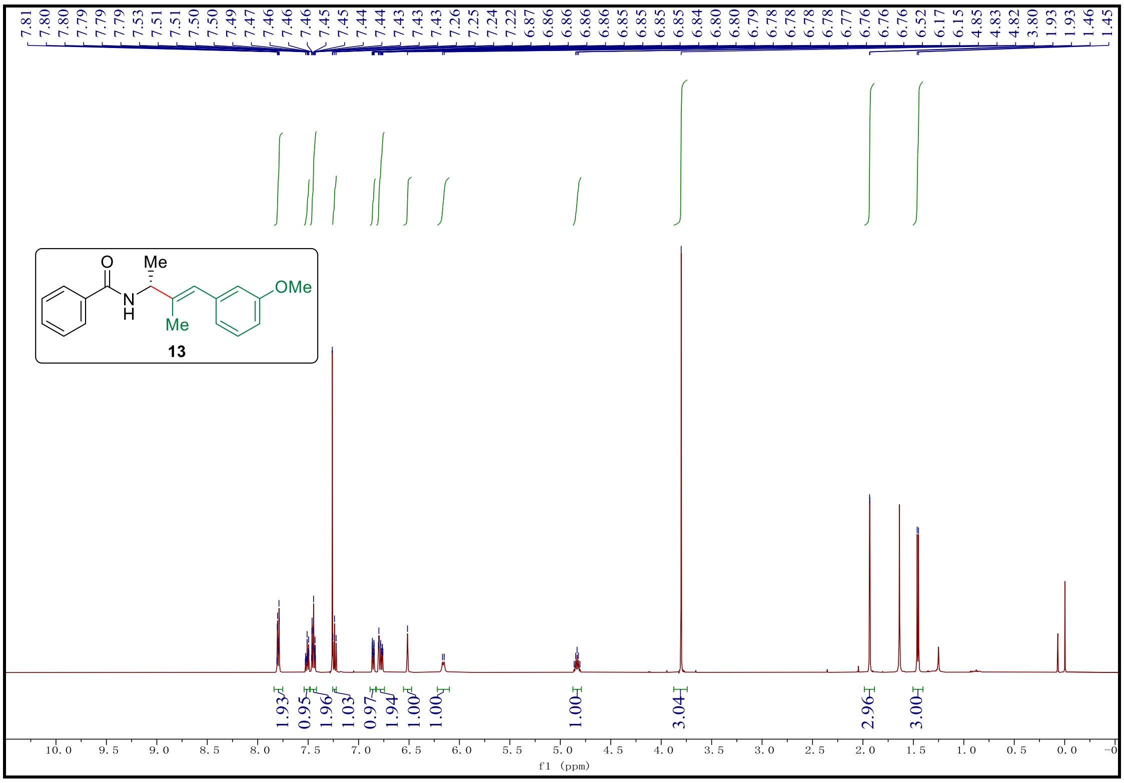

Supplementary Figure 29. ^{1}H NMR Spectrum of Compound **13** (500 MHz, $CDCl_3$, 25 ˚C)

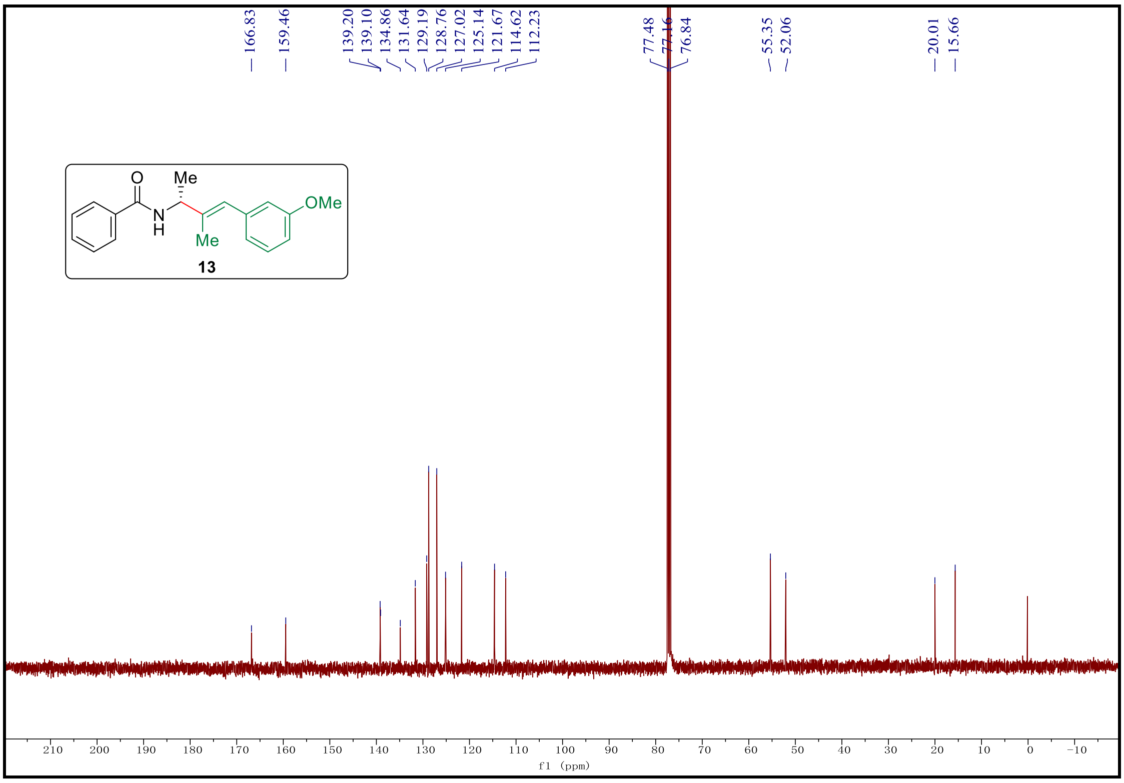

Supplementary Figure 30. ^{13}C NMR Spectrum of Compound **13** (101 MHz, $CDCl_3$, 25 ˚C)

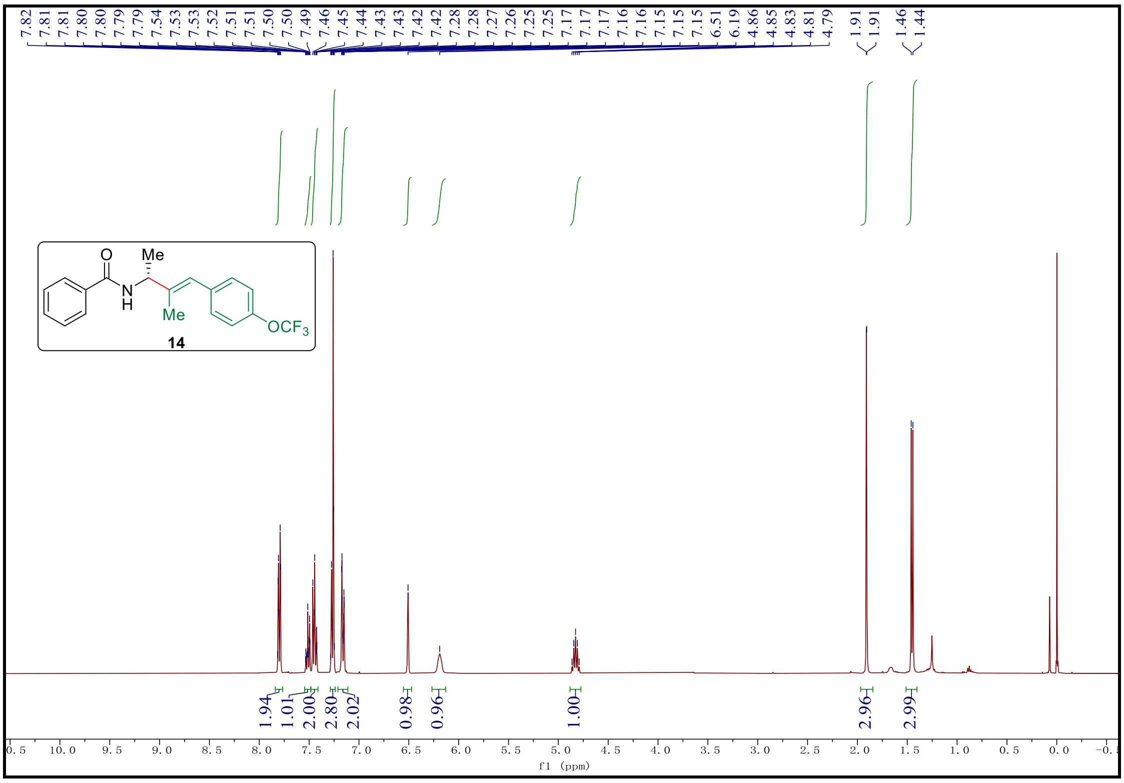

Supplementary Figure 31. ^{1}H NMR Spectrum of Compound **14** (400 MHz, $CDCl_3$, 25 ˚C)

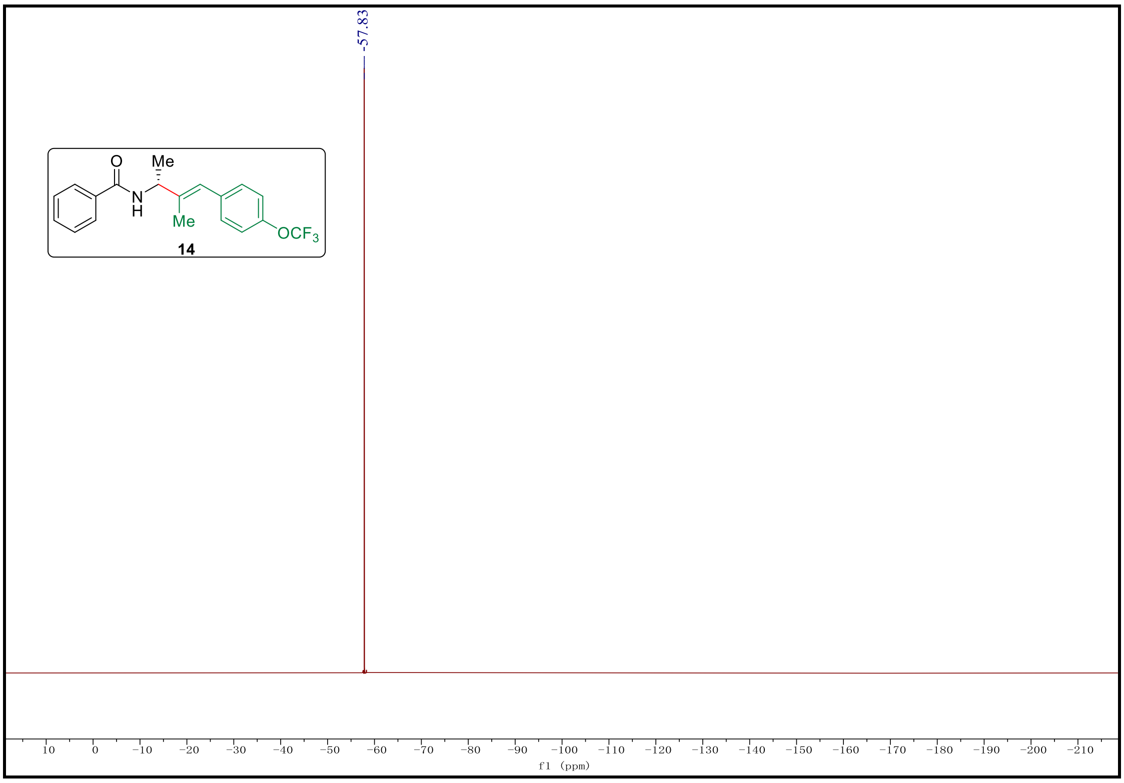

Supplementary Figure 32. ^{19}F NMR Spectrum of Compound **14** (376 MHz, $CDCl_3$, 25 ˚C)

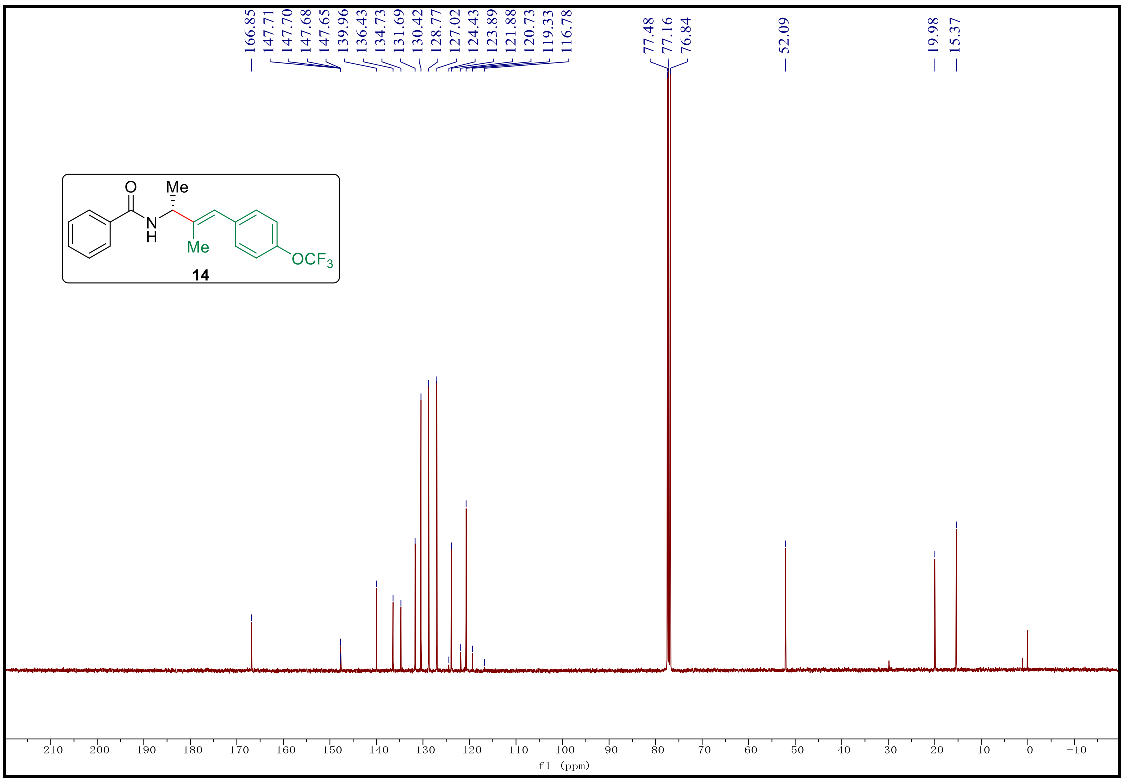

Supplementary Figure 33. ^{13}C NMR Spectrum of Compound **14** (101 MHz, $CDCl_3$, 25 ˚C)

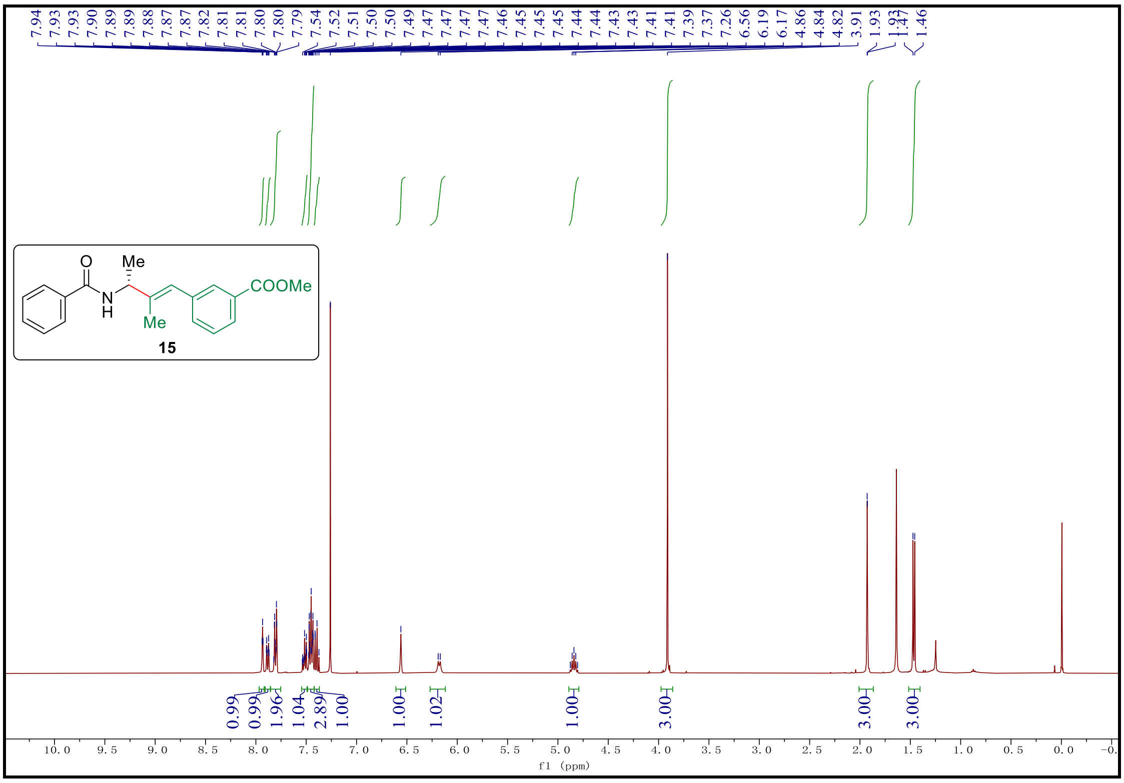

Supplementary Figure 34. ^{1}H NMR Spectrum of Compound **15** (400 MHz, $CDCl_3$, 25 ˚C)

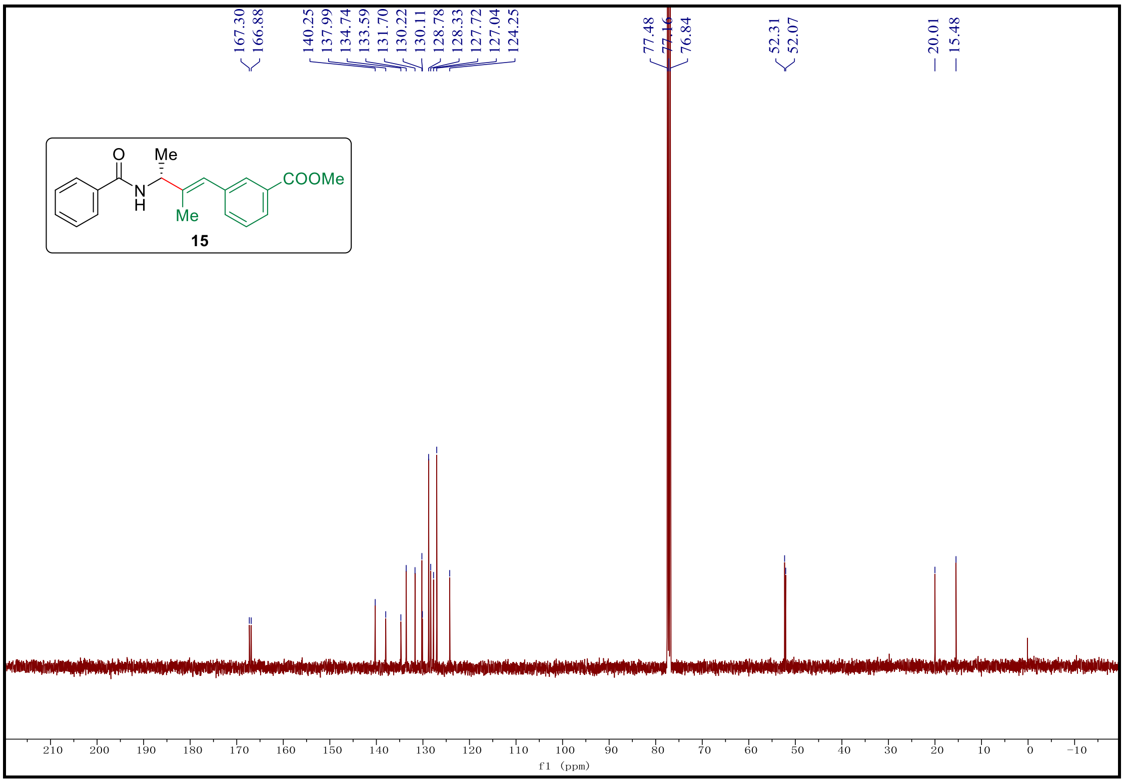

Supplementary Figure 35. ^{13}C NMR Spectrum of Compound **15** (101 MHz, $CDCl_3$, 25 ˚C)

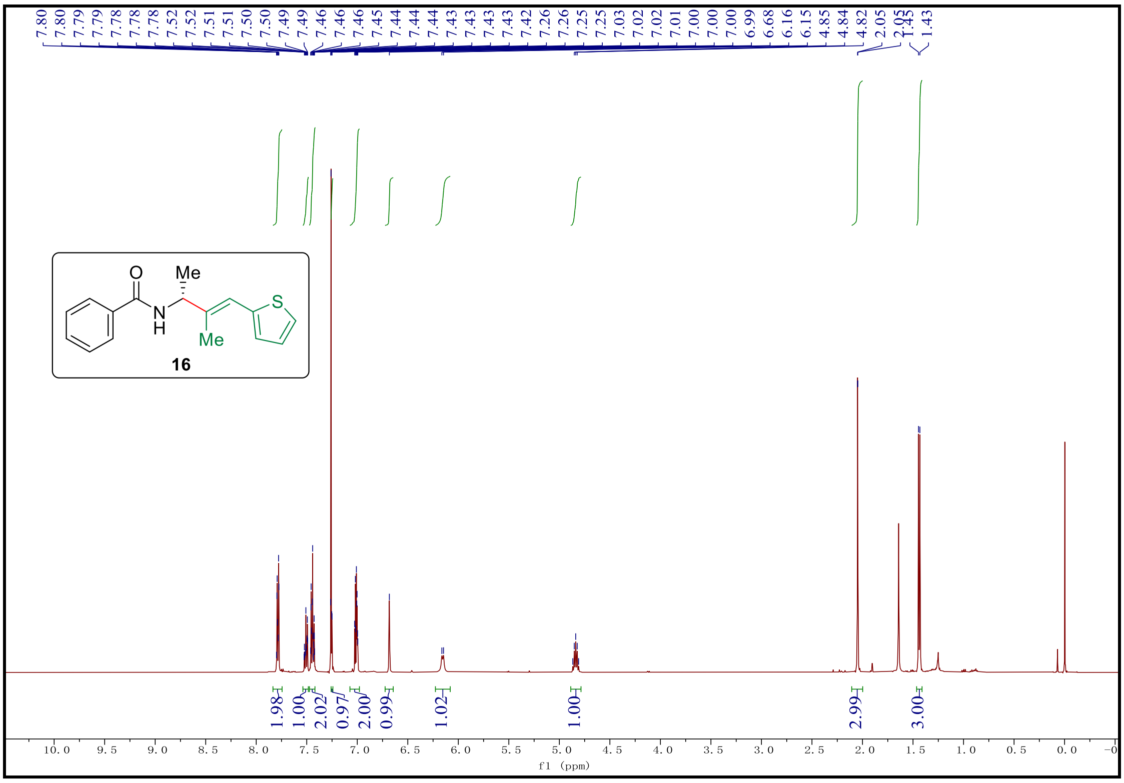

Supplementary Figure 36. ^{1}H NMR Spectrum of Compound **16** (500 MHz, $CDCl_3$, 25 ˚C)

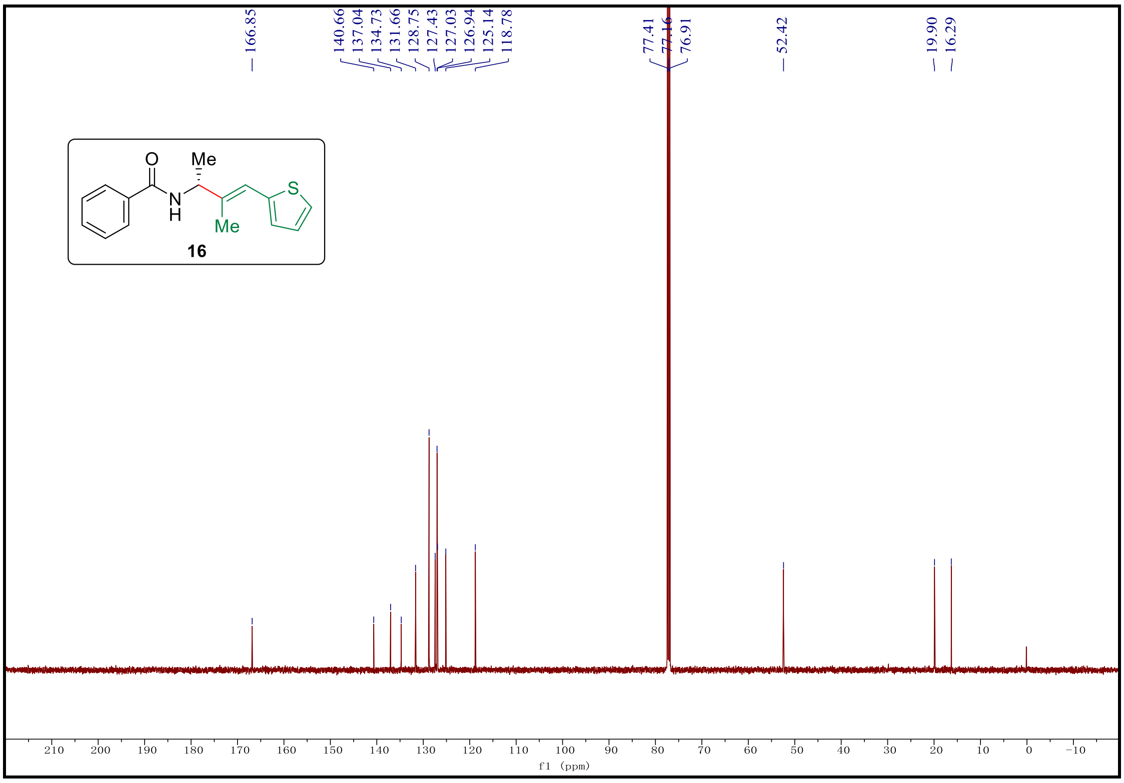

Supplementary Figure 37. ^{13}C NMR Spectrum of Compound **16** (126 MHz, $CDCl_3$, 25 ˚C)

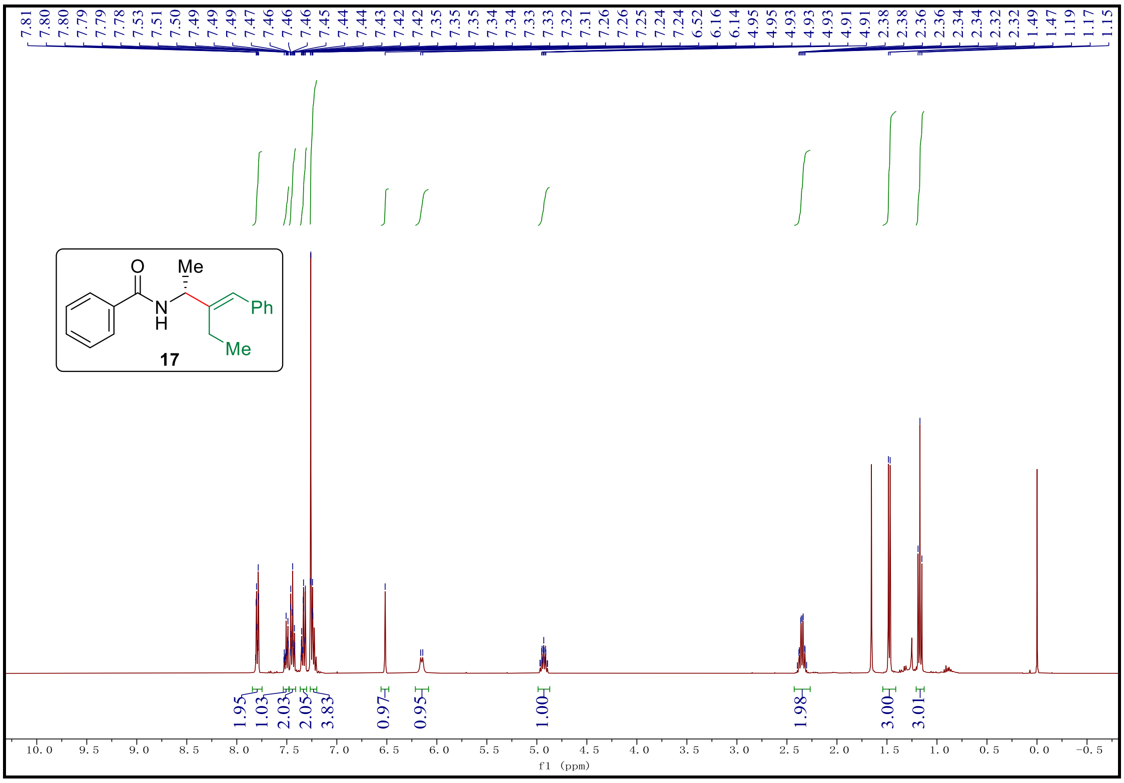

Supplementary Figure 38. ^{1}H NMR Spectrum of Compound **17** (400 MHz, $CDCl_3$, 25 ˚C)

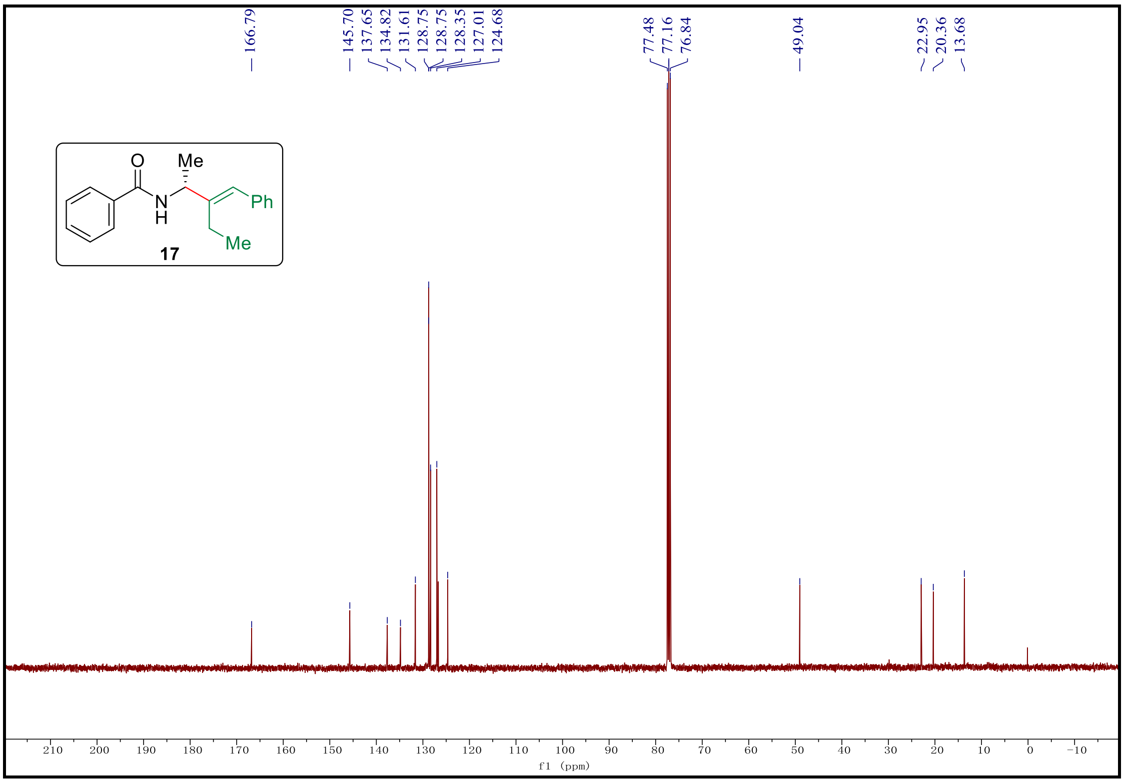

Supplementary Figure 39. ^{13}C NMR Spectrum of Compound **17** (101 MHz, $CDCl_3$, 25 ˚C)

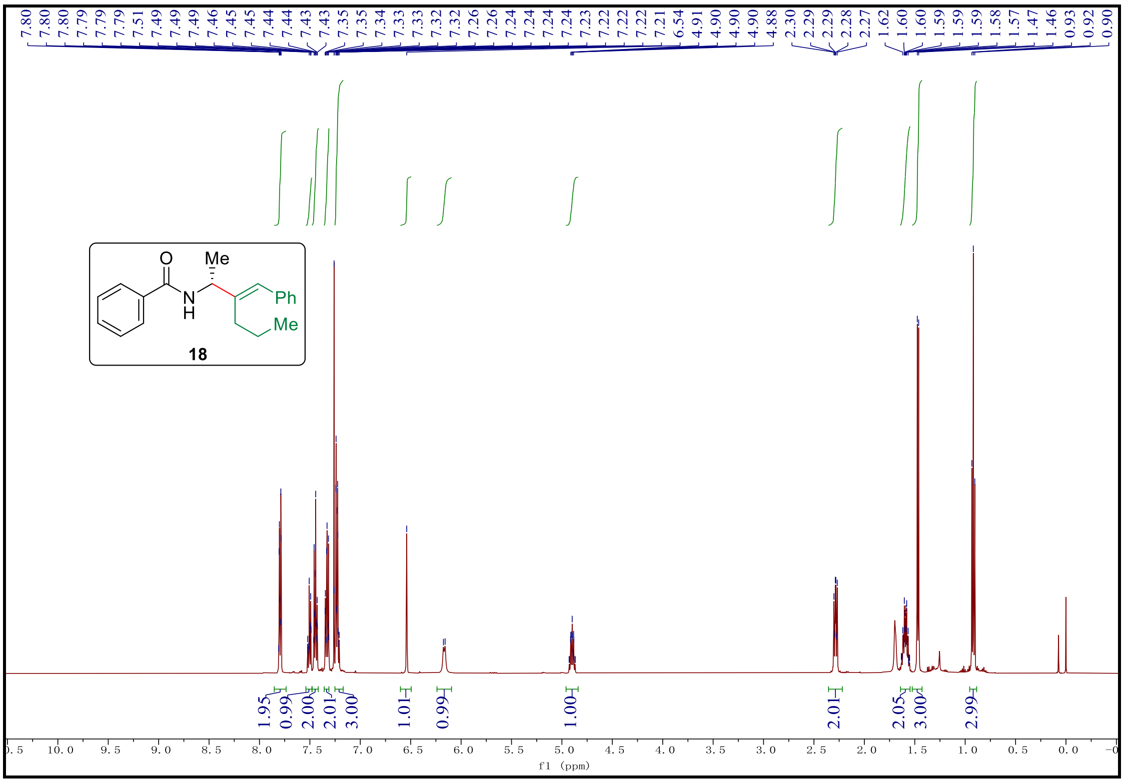

Supplementary Figure 40. ^{1}H NMR Spectrum of Compound **18** (500 MHz, $CDCl_3$, 25 ˚C)

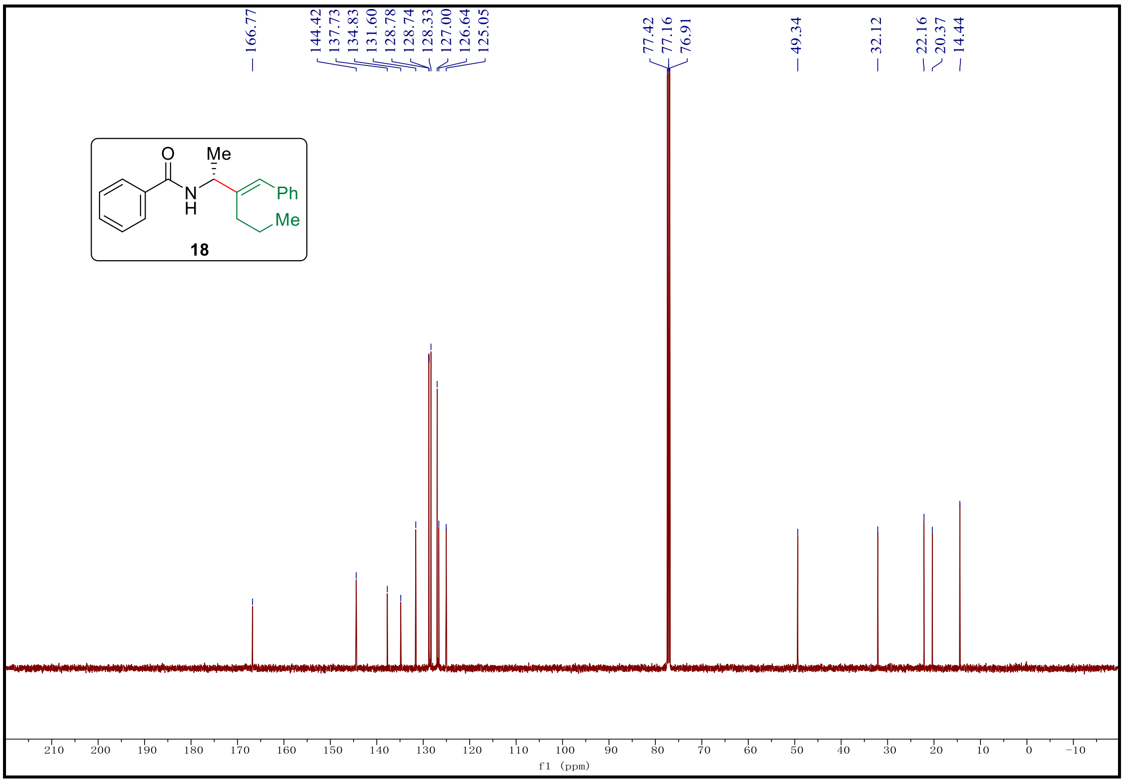

Supplementary Figure 41. ^{13}C NMR Spectrum of Compound **18** (126 MHz, $CDCl_3$, 25 ˚C)

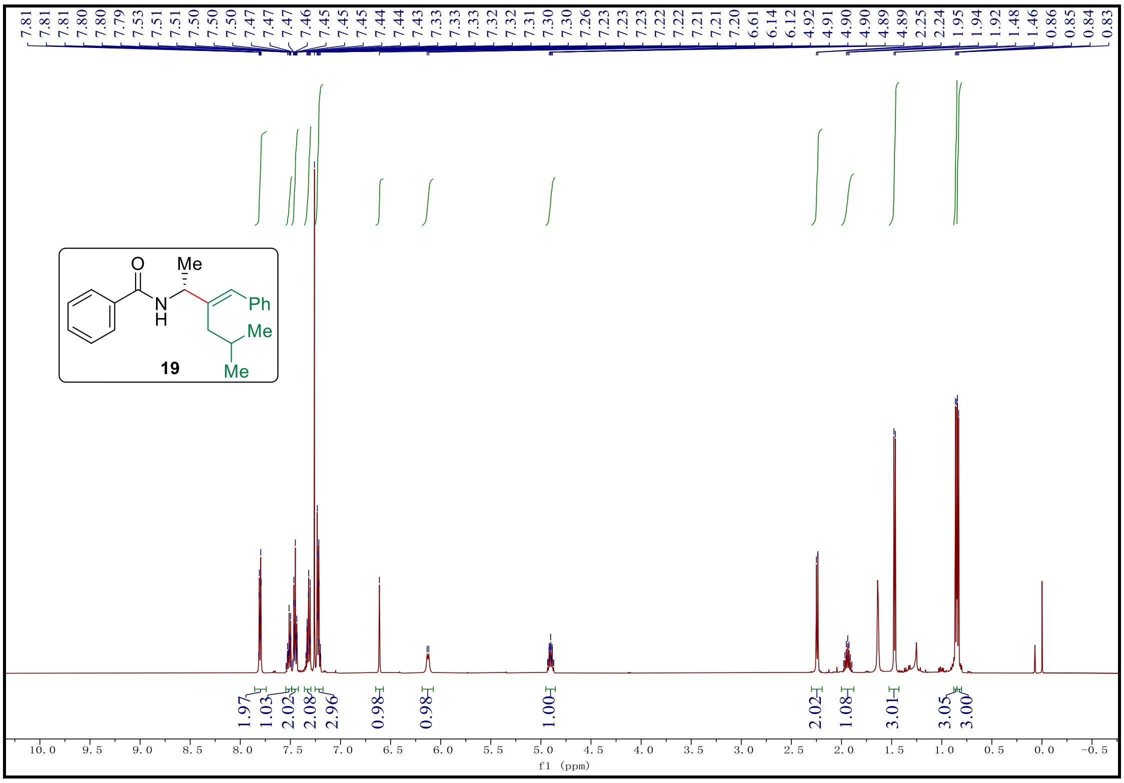

Supplementary Figure 42. ^{1}H NMR Spectrum of Compound **19** (500 MHz, $CDCl_3$, 25 ˚C)

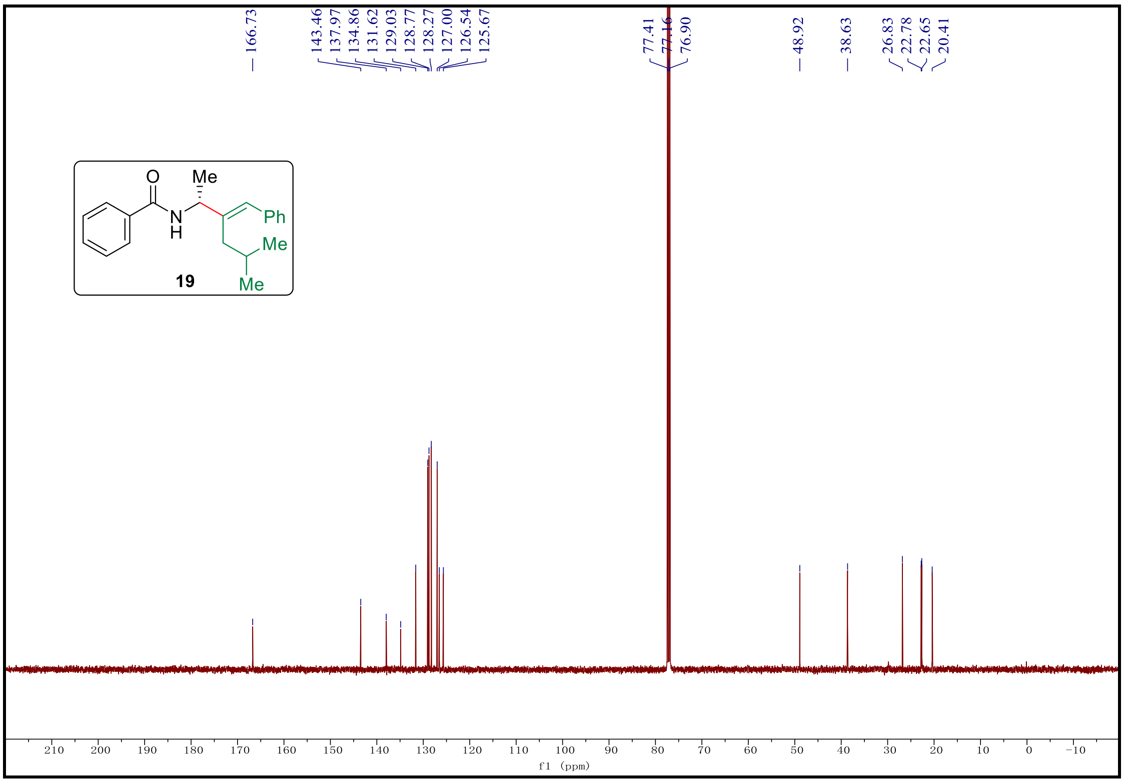

Supplementary Figure 43. ^{13}C NMR Spectrum of Compound **19** (126 MHz, $CDCl_3$, 25 ˚C)

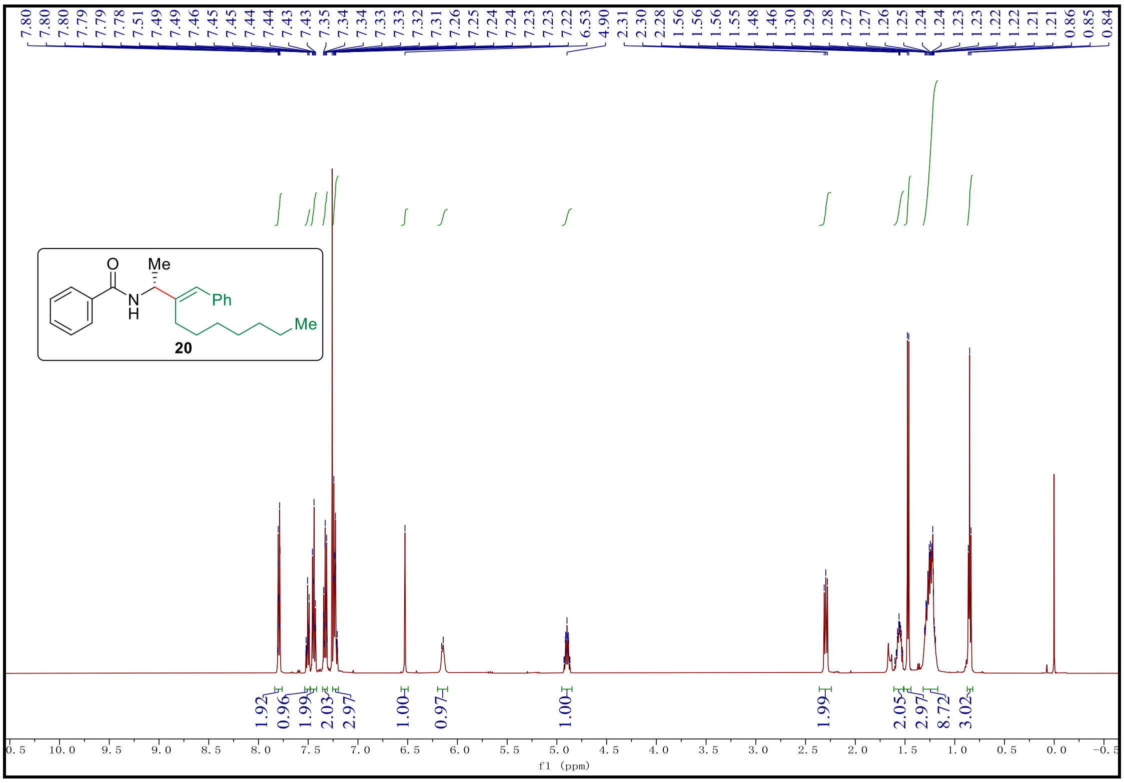

Supplementary Figure 44. ^{1}H NMR Spectrum of Compound **20** (500 MHz, $CDCl_3$, 25 ˚C)

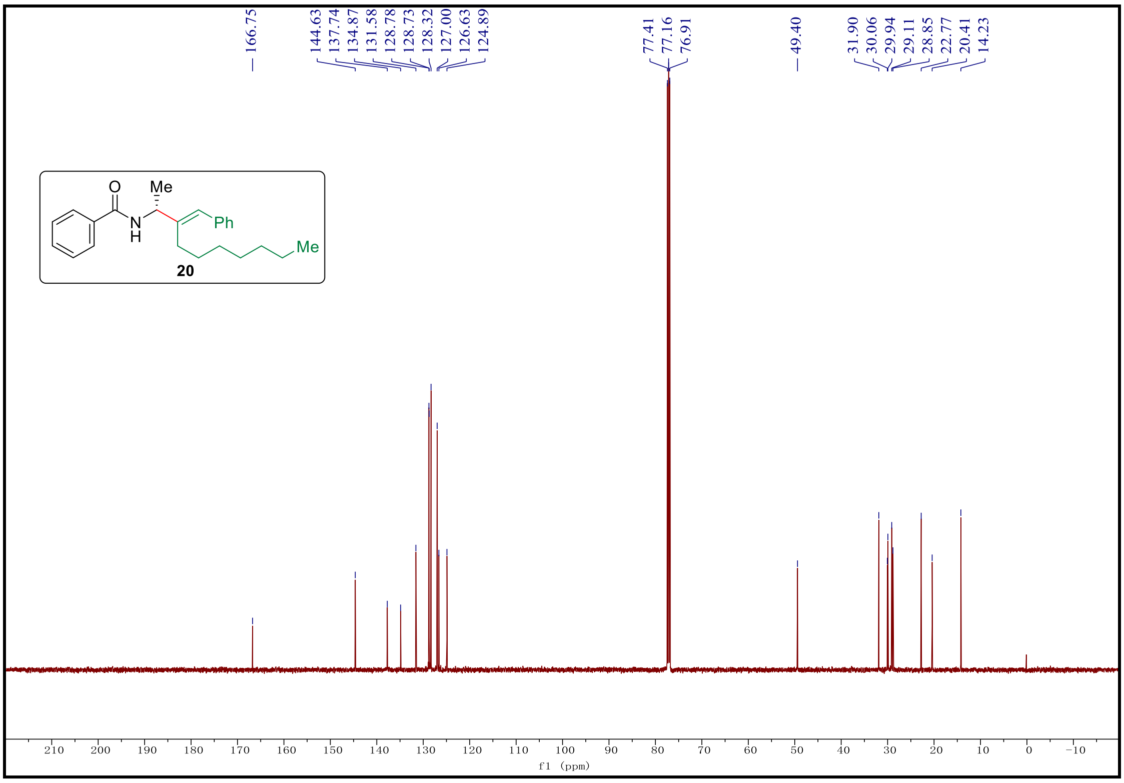

Supplementary Figure 45. ^{13}C NMR Spectrum of Compound **20** (126 MHz, $CDCl_3$, 25 ˚C)

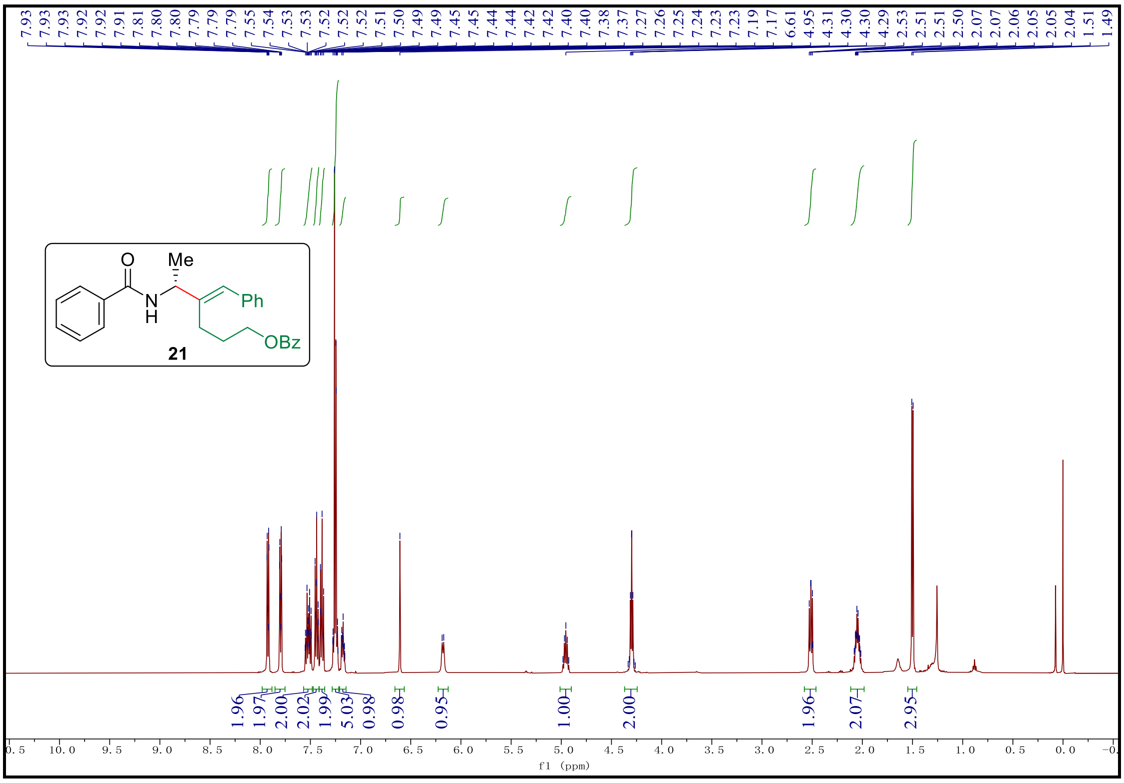

Supplementary Figure 46. ^{1}H NMR Spectrum of Compound **21** (500 MHz, $CDCl_3$, 25 ˚C)

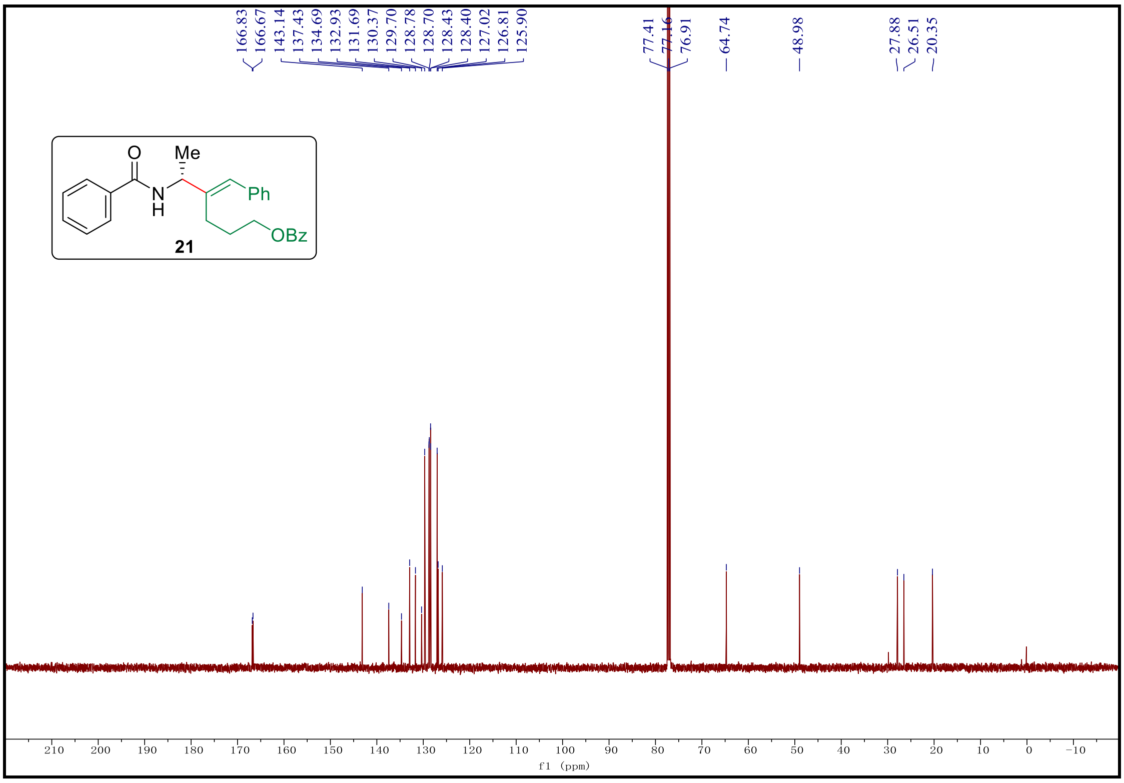

Supplementary Figure 47. ^{13}C NMR Spectrum of Compound **21** (126 MHz, $CDCl_3$, 25 ˚C)

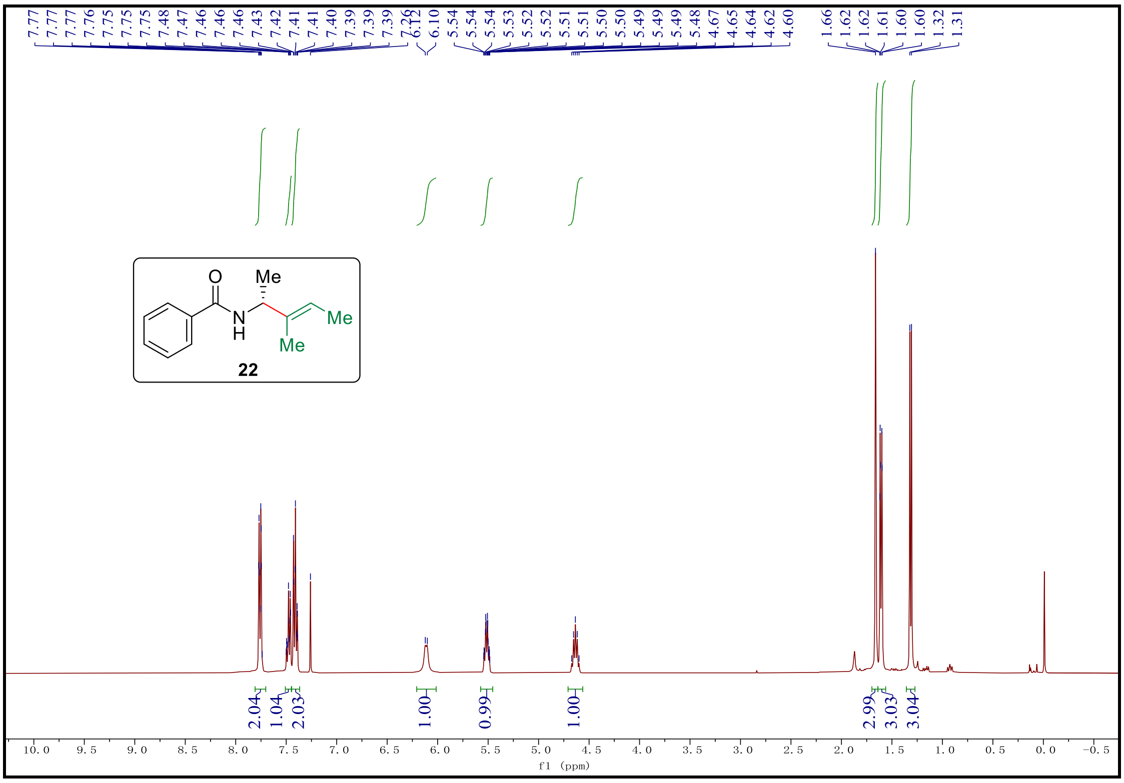

Supplementary Figure 48. ^{1}H NMR Spectrum of Compound **22** (400 MHz, $CDCl_3$, 25 ˚C)

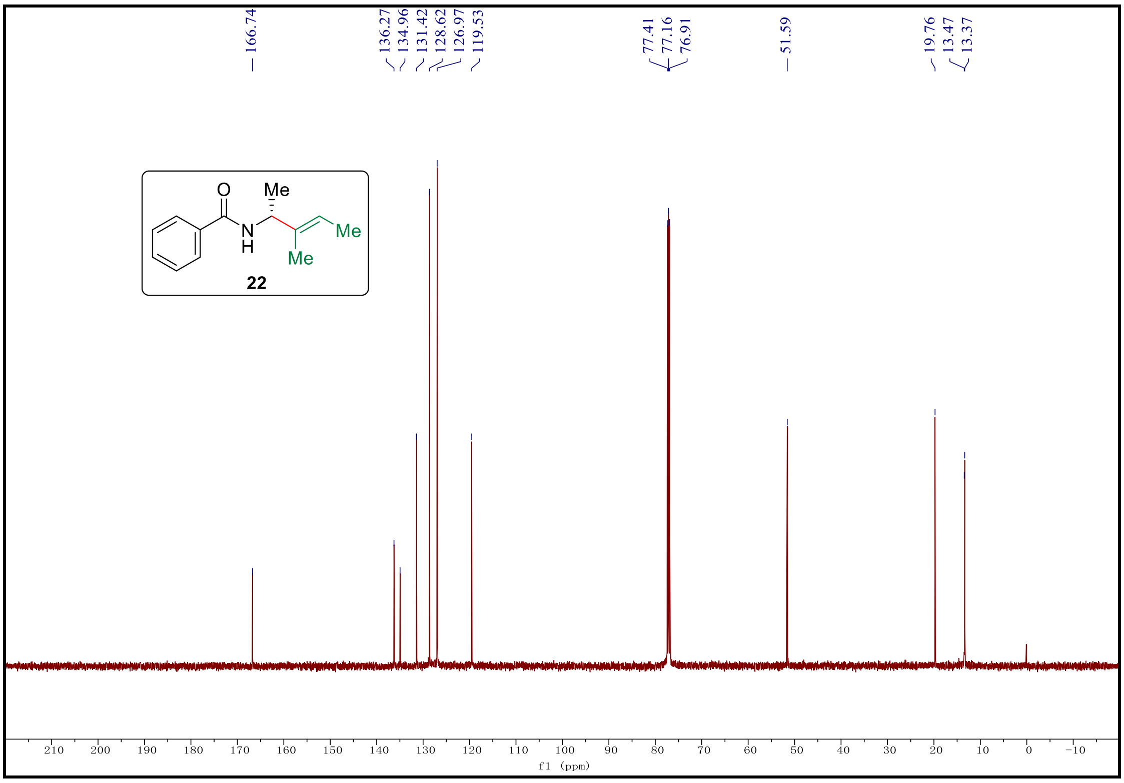

Supplementary Figure 49. ^{13}C NMR Spectrum of Compound **22** (126 MHz, $CDCl_3$, 25 ˚C)

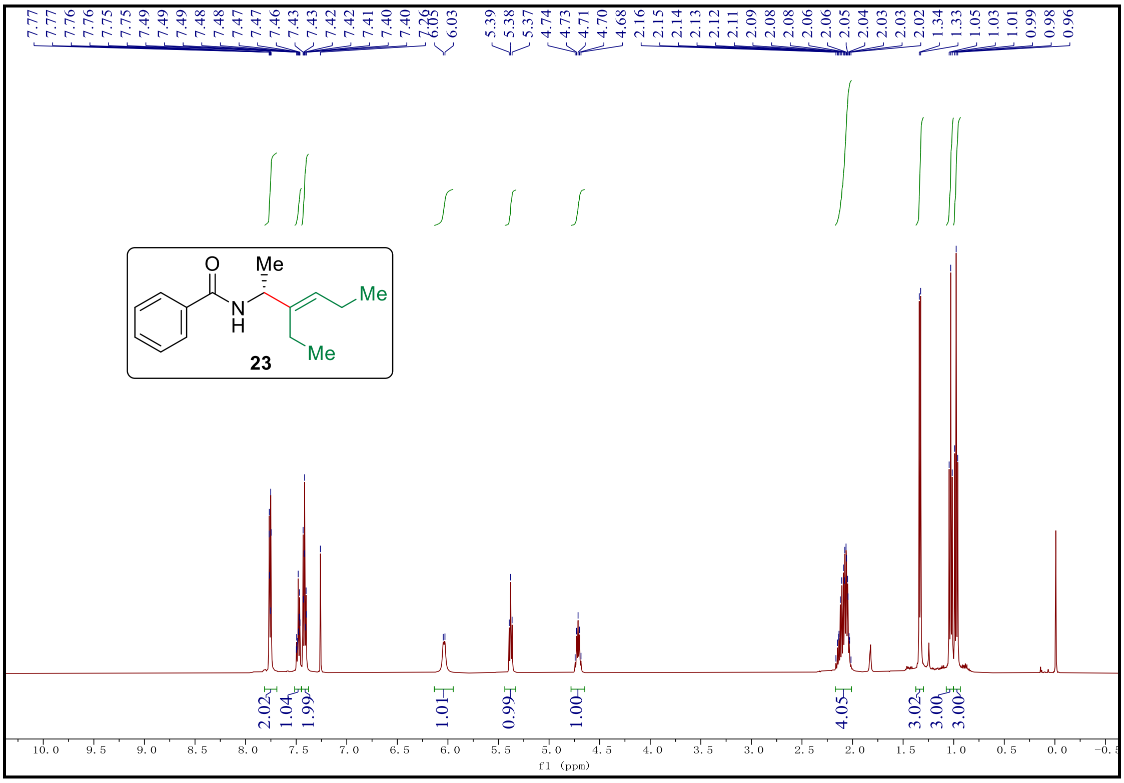

Supplementary Figure 50. ^{1}H NMR Spectrum of Compound **23** (500 MHz, $CDCl_3$, 25 ˚C)

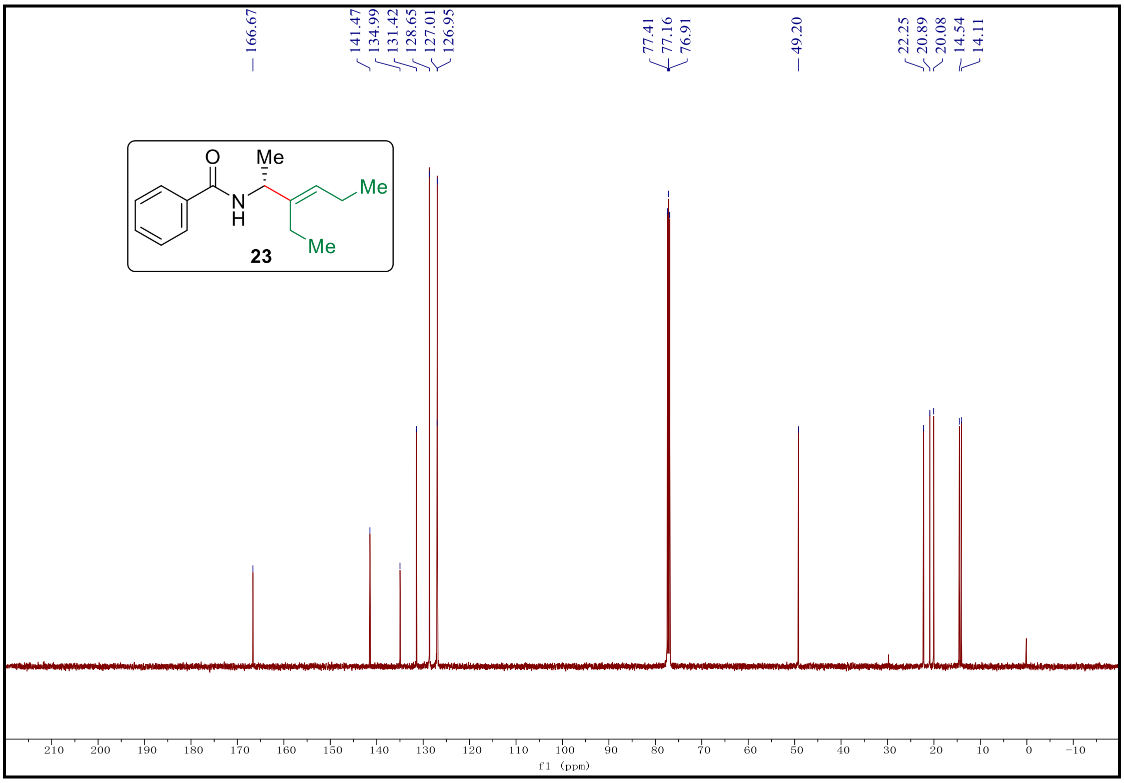

Supplementary Figure 51. ^{13}C NMR Spectrum of Compound **23** (126 MHz, $CDCl_3$, 25 ˚C)

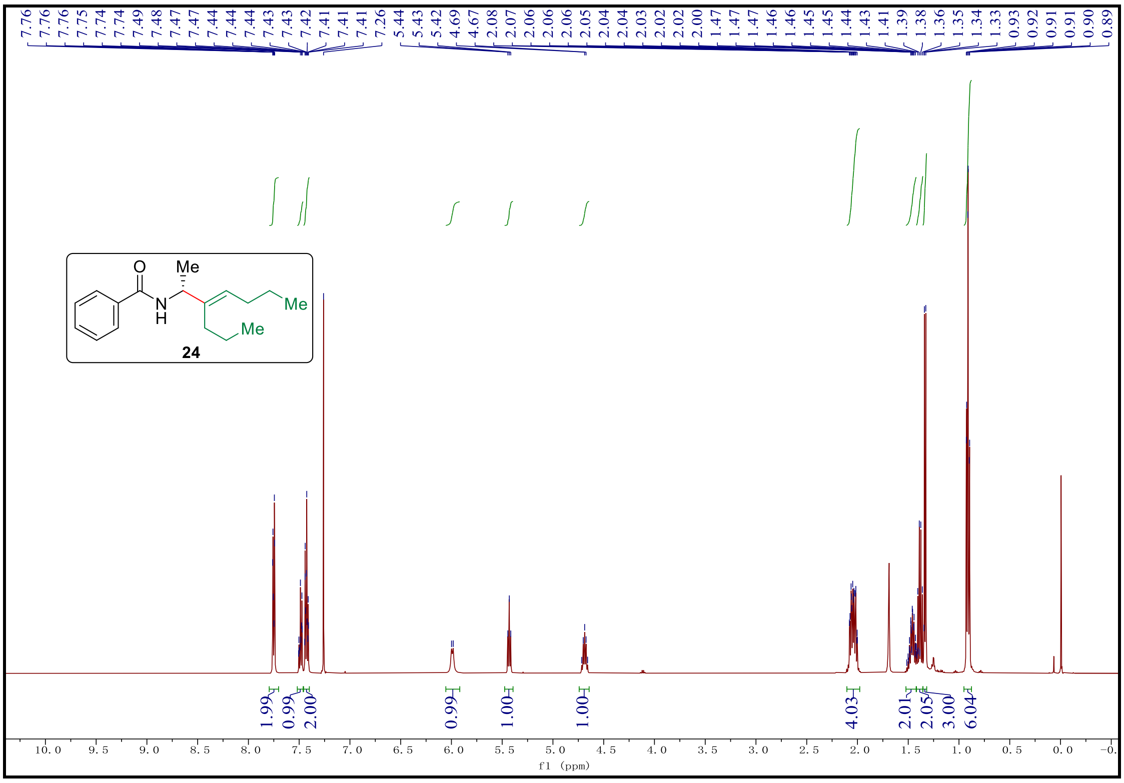

Supplementary Figure 52. ^{1}H NMR Spectrum of Compound **24** (500 MHz, $CDCl_3$, 25 ˚C)

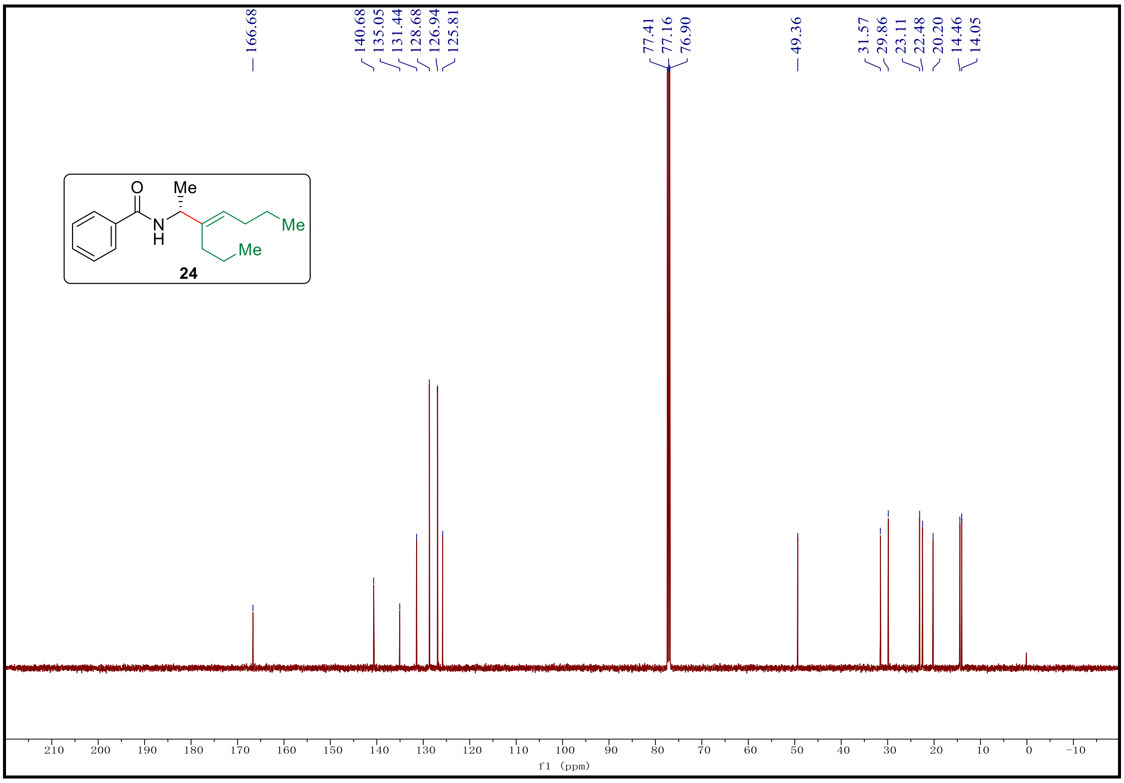

Supplementary Figure 53. ^{13}C NMR Spectrum of Compound **24** (126 MHz, $CDCl_3$, 25 ˚C)

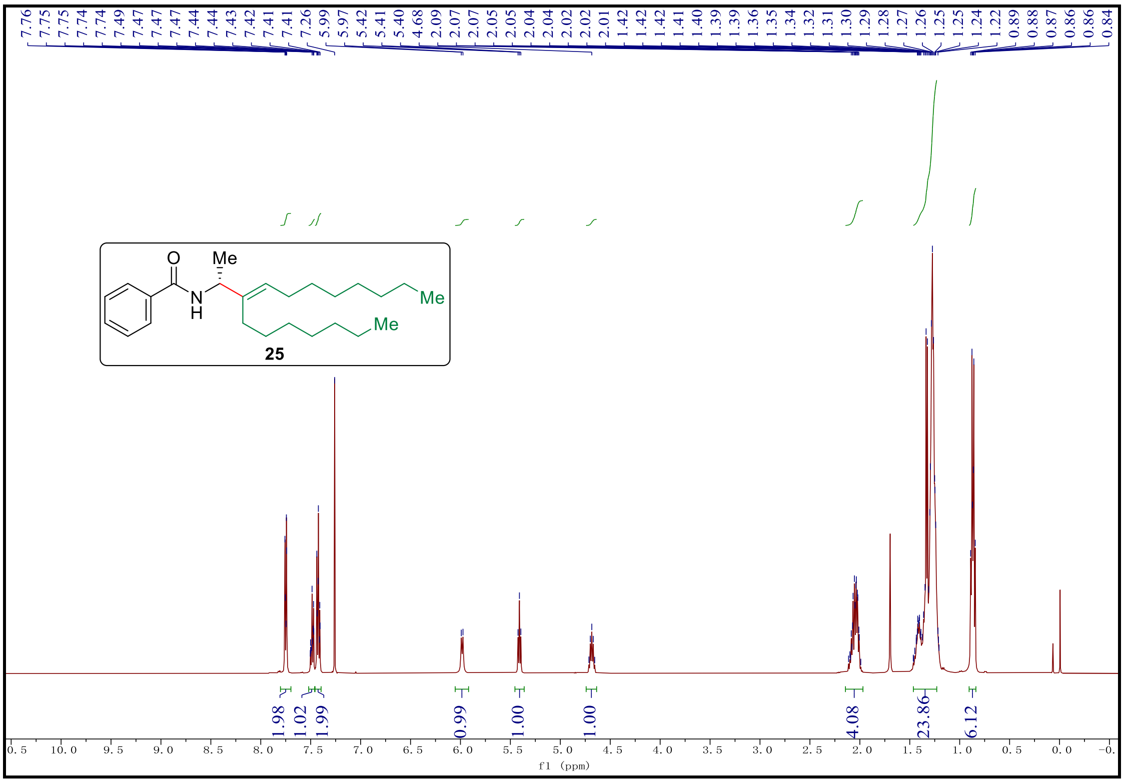

Supplementary Figure 54. ^{1}H NMR Spectrum of Compound **25** (500 MHz, $CDCl_3$, 25 ˚C)

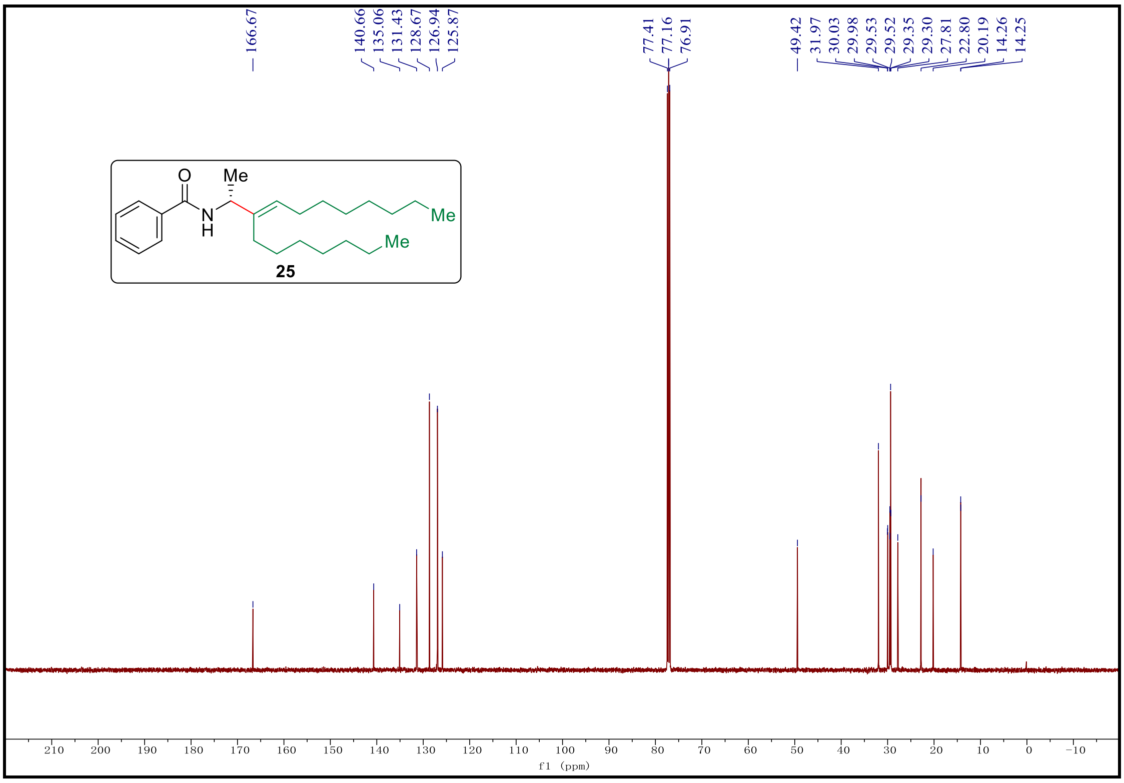

Supplementary Figure 55. ^{13}C NMR Spectrum of Compound **25** (126 MHz, $CDCl_3$, 25 ˚C)

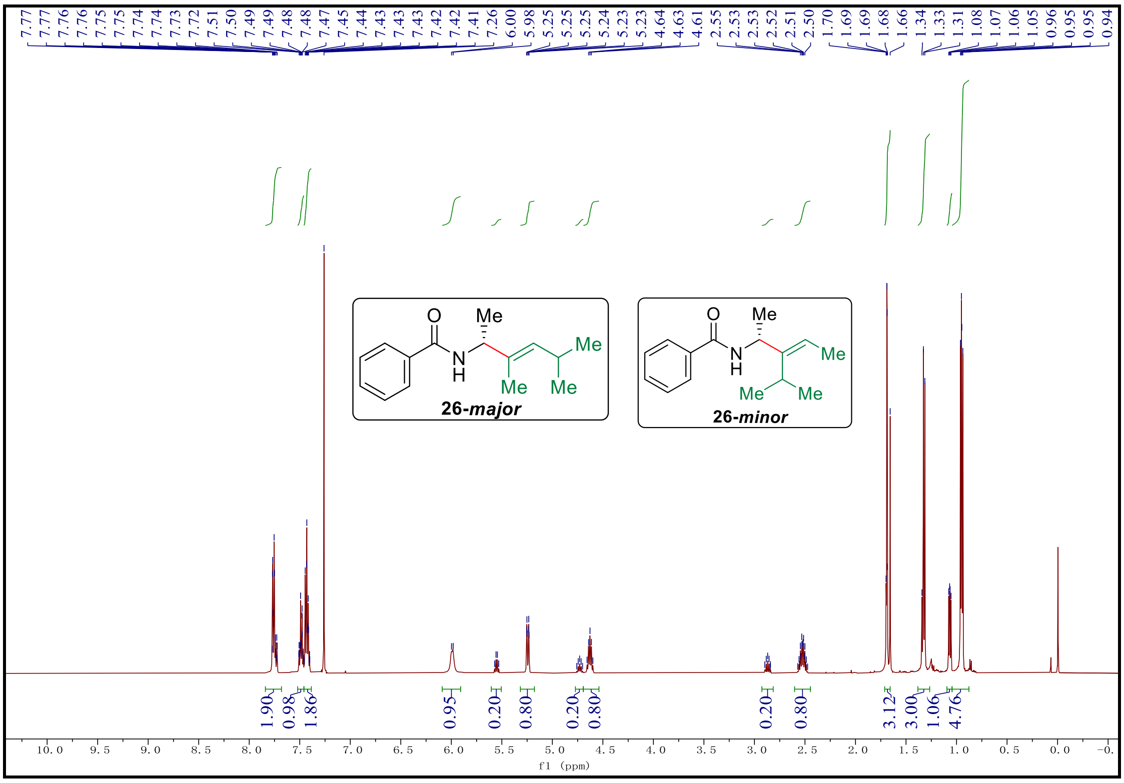

Supplementary Figure 56. ^{1}H NMR Spectrum of Compound **26** (500 MHz, $CDCl_3$, 25 °C)

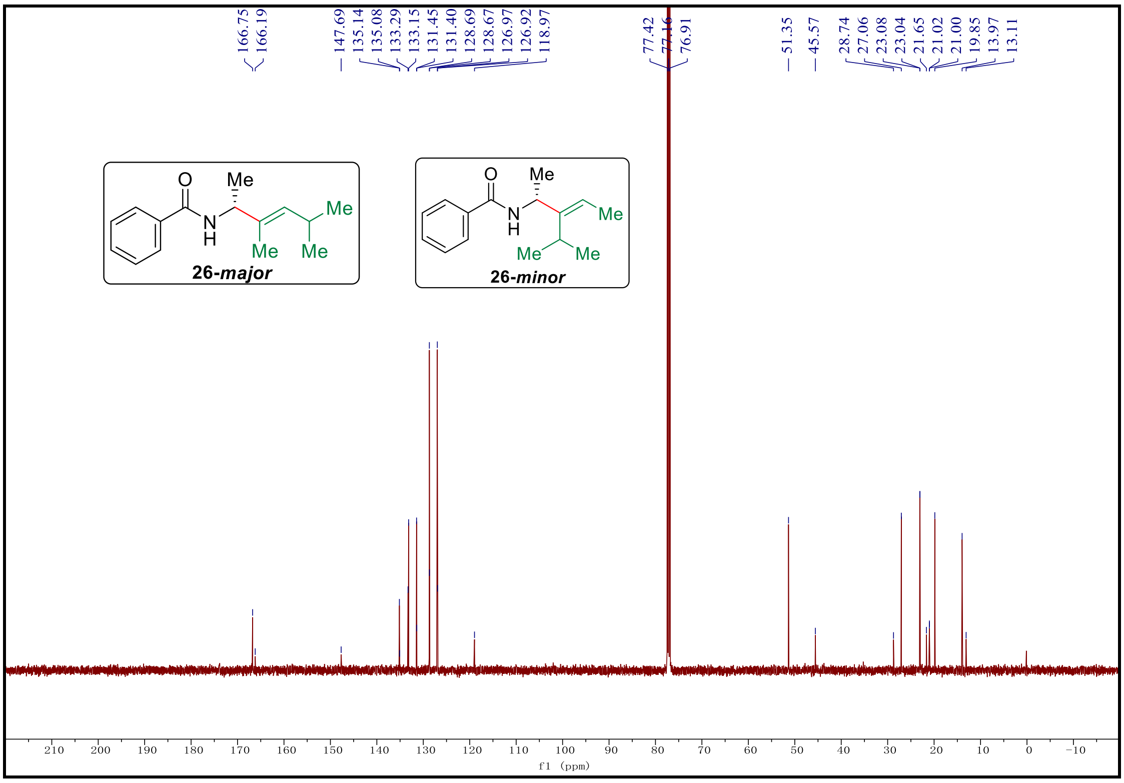

Supplementary Figure 57. ^{13}C NMR Spectrum of Compound **26** (126 MHz, $CDCl_3$, 25 °C)

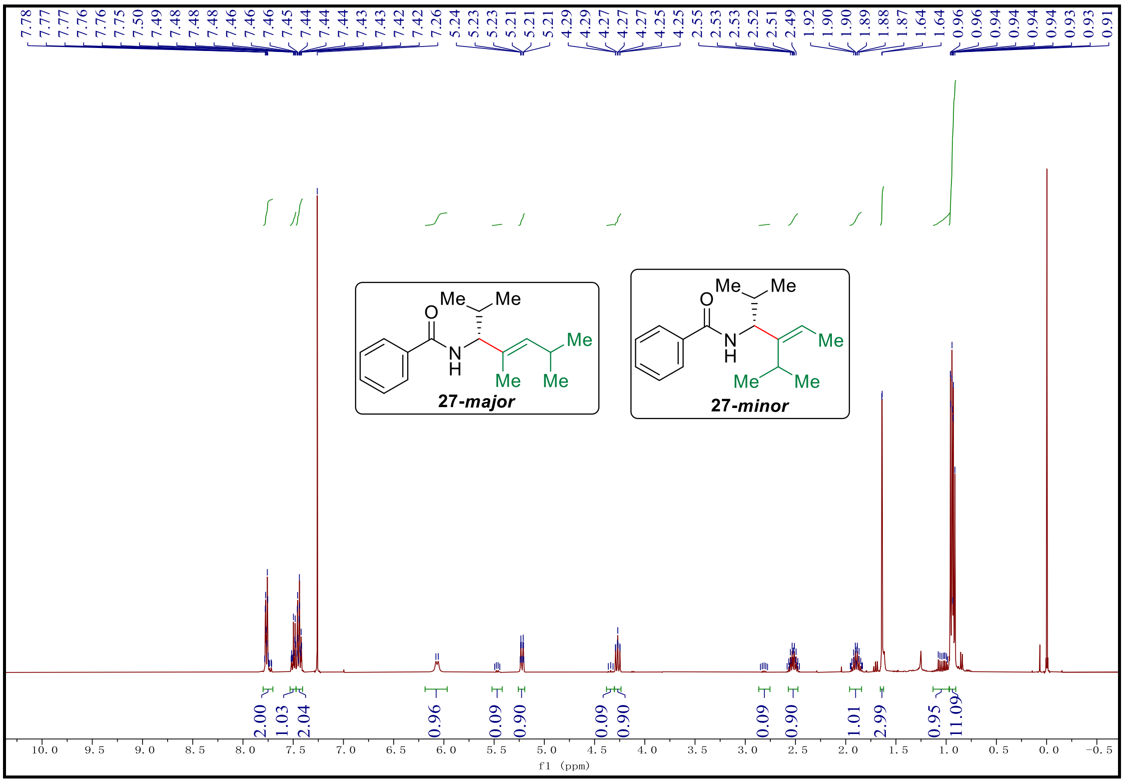

Supplementary Figure 58. ^{1}H NMR Spectrum of Compound **27** (400 MHz, $CDCl_3$, 25 ˚C)

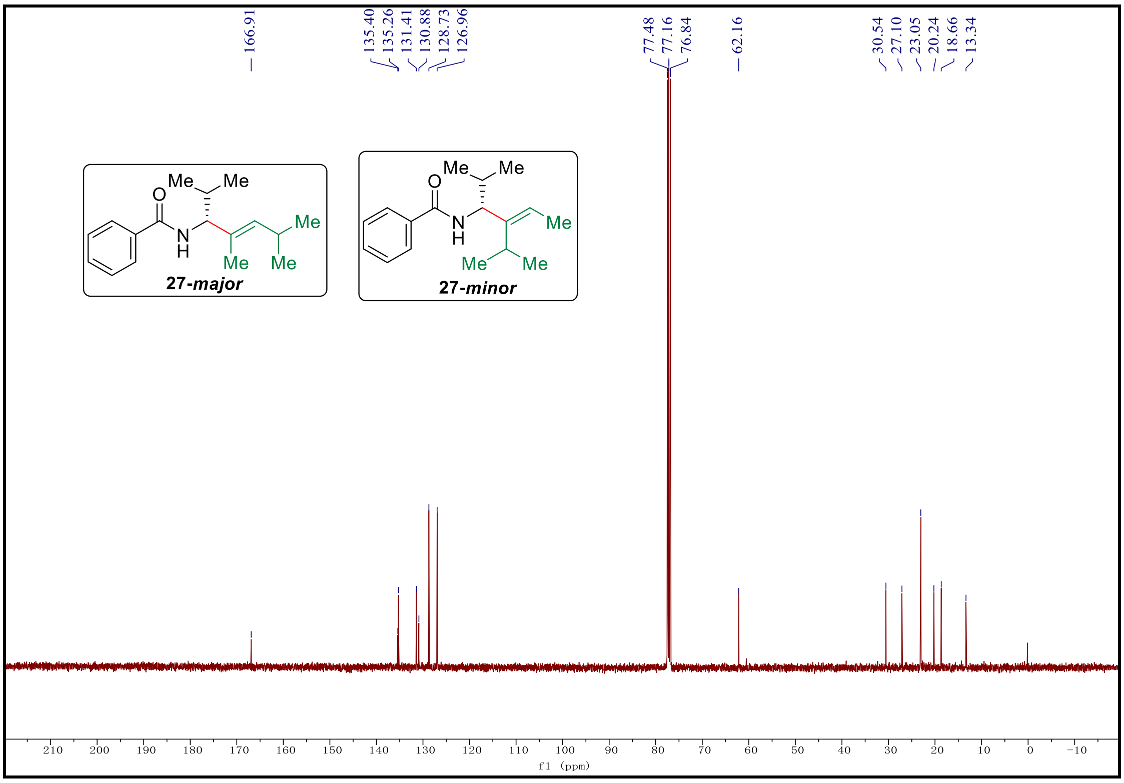

Supplementary Figure 59. ^{13}C NMR Spectrum of Compound **27** (101 MHz, $CDCl_3$, 25 ˚C)

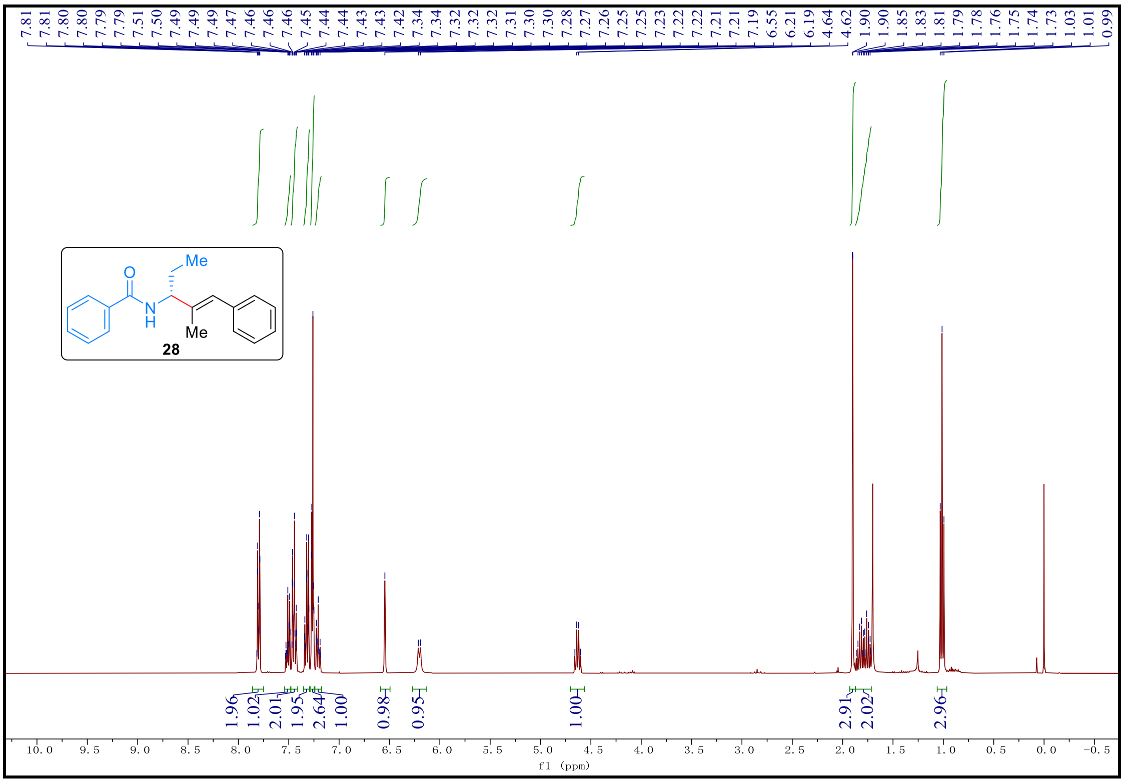

Supplementary Figure 60. ^{1}H NMR Spectrum of Compound **28** (400 MHz, $CDCl_3$, 25 ˚C)

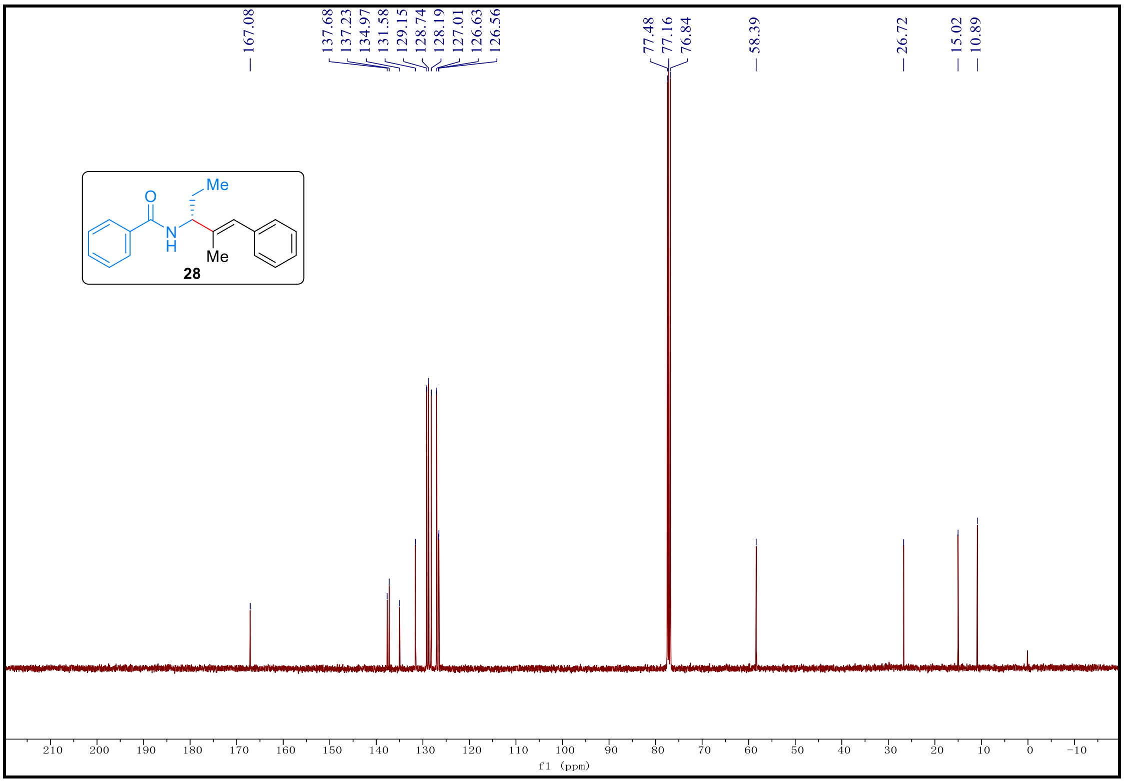

Supplementary Figure 61. ^{13}C NMR Spectrum of Compound **28** (101 MHz, $CDCl_3$, 25 ˚C)

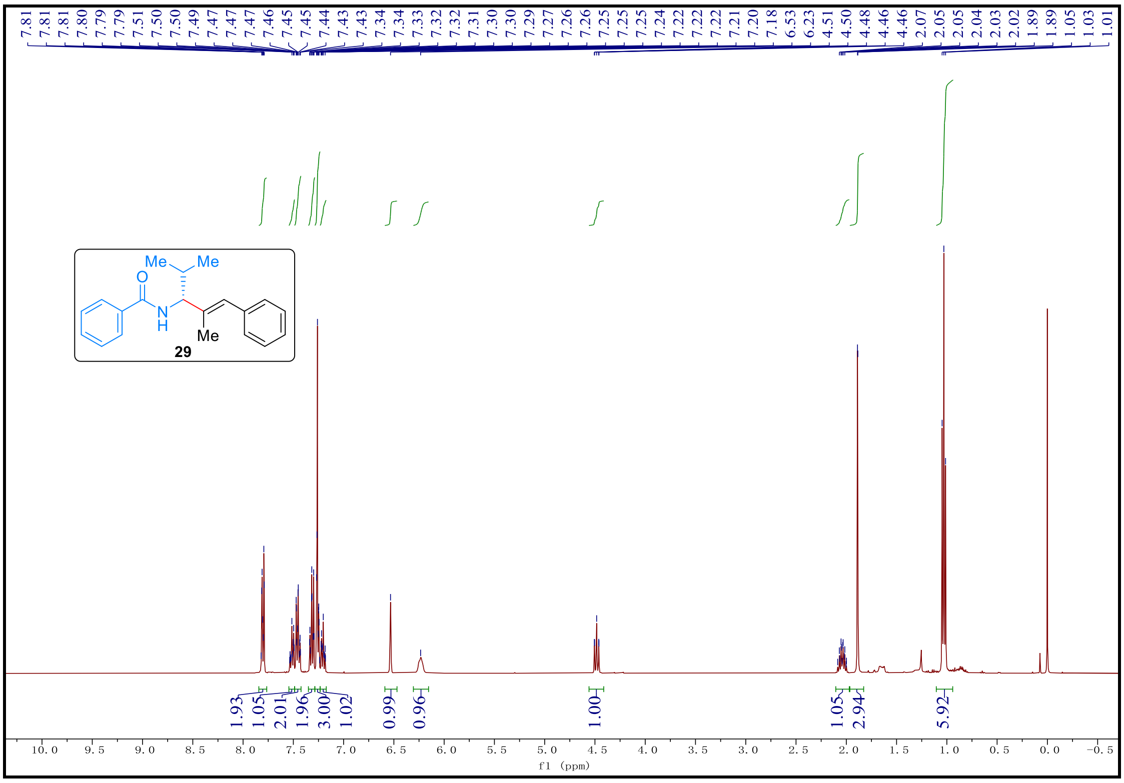

Supplementary Figure 62. ^{1}H NMR Spectrum of Compound **29** (400 MHz, $CDCl_3$, 25 ˚C)

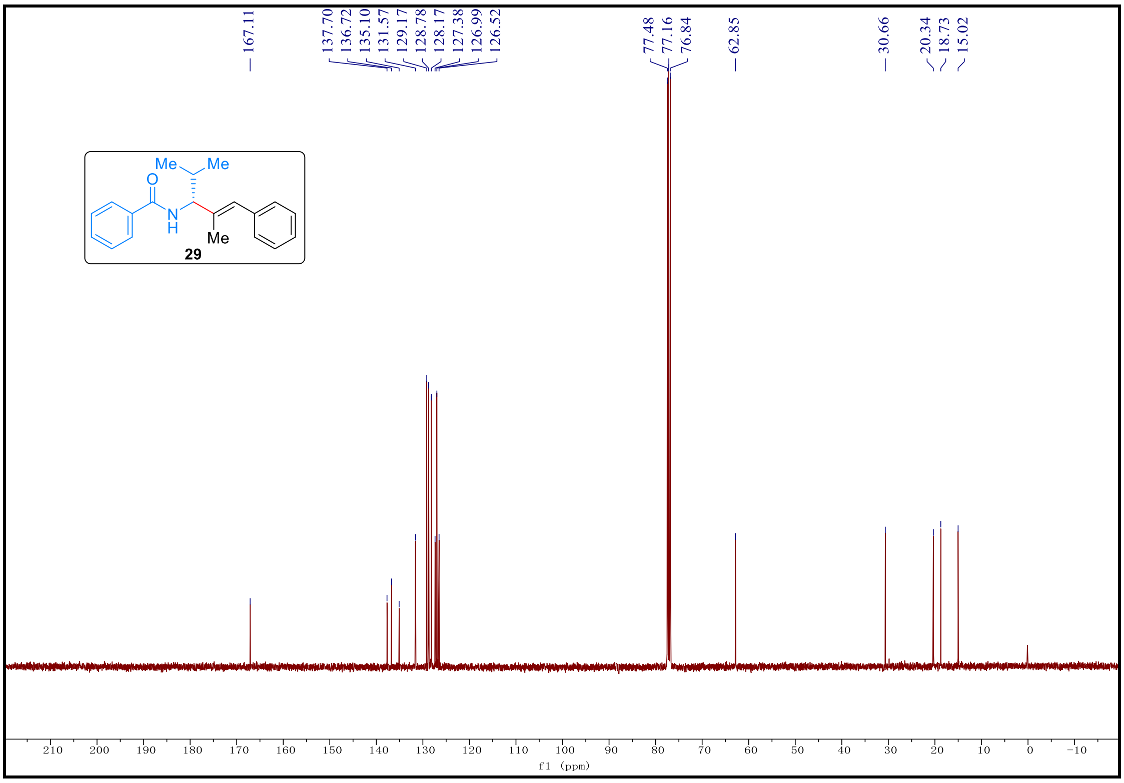

Supplementary Figure 63. ^{13}C NMR Spectrum of Compound **29** (101 MHz, $CDCl_3$, 25 ˚C)

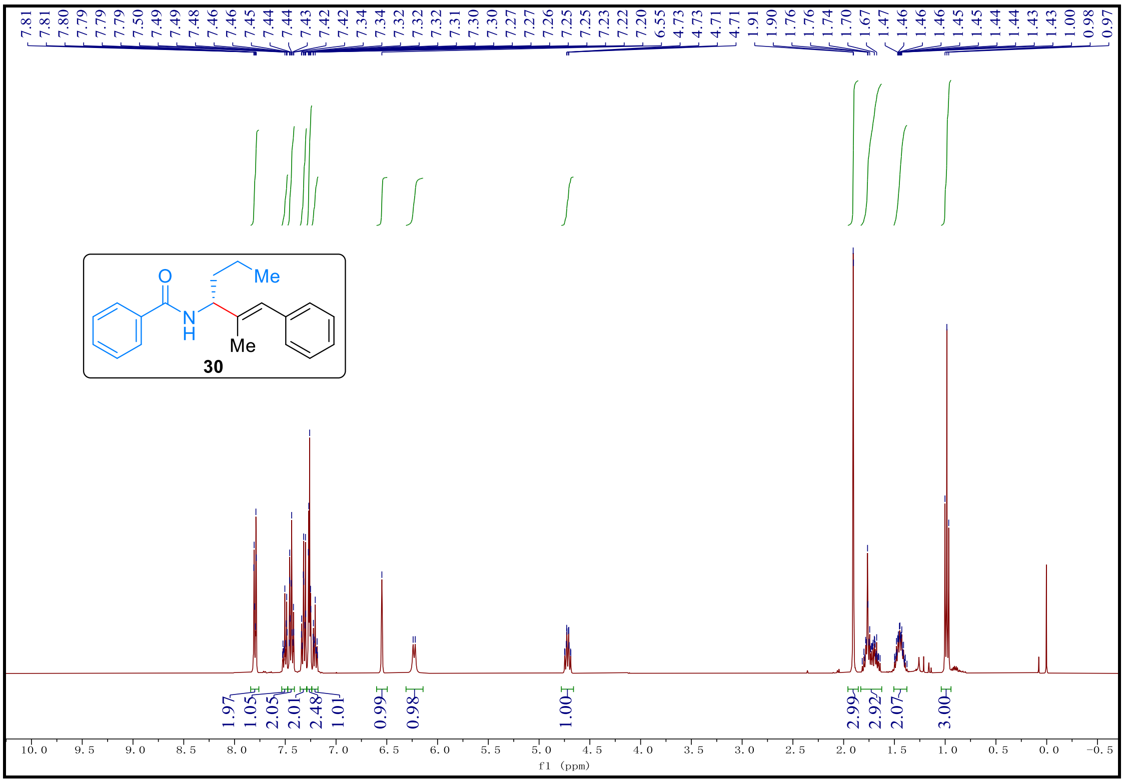

Supplementary Figure 64. ^{1}H NMR Spectrum of Compound **30** (400 MHz, $CDCl_3$, 25 ˚C)

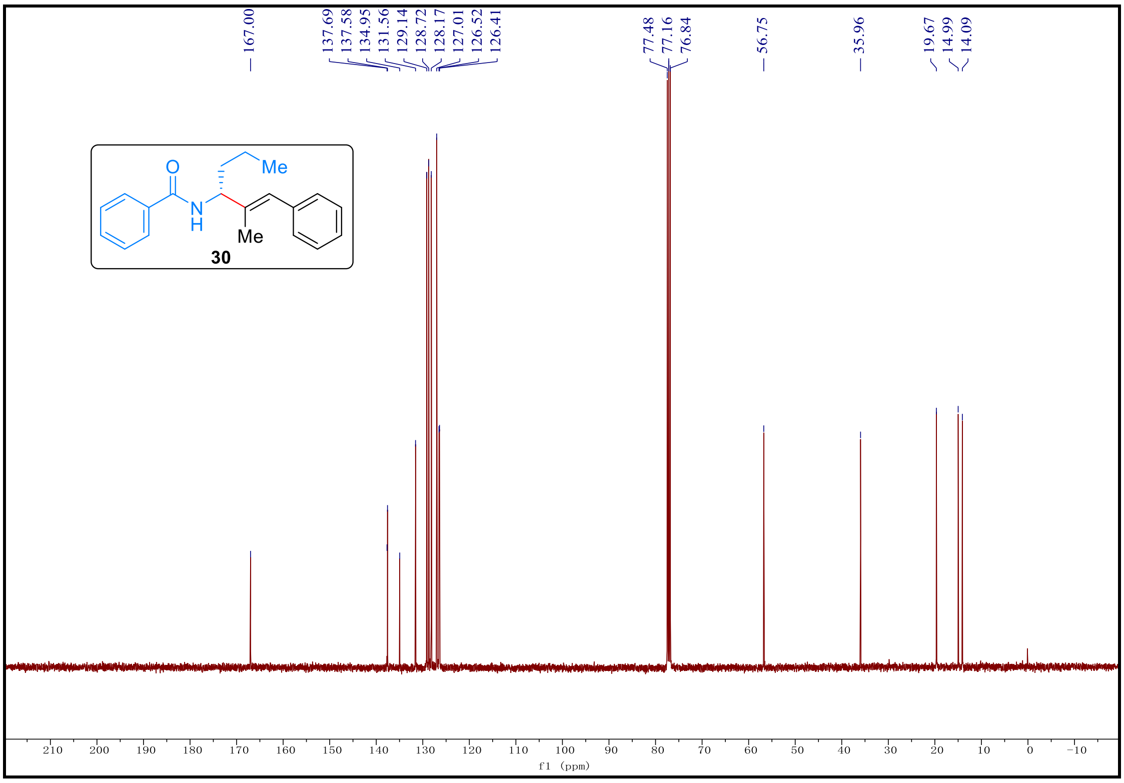

Supplementary Figure 65. ^{13}C NMR Spectrum of Compound **30** (101 MHz, $CDCl_3$, 25 ˚C)

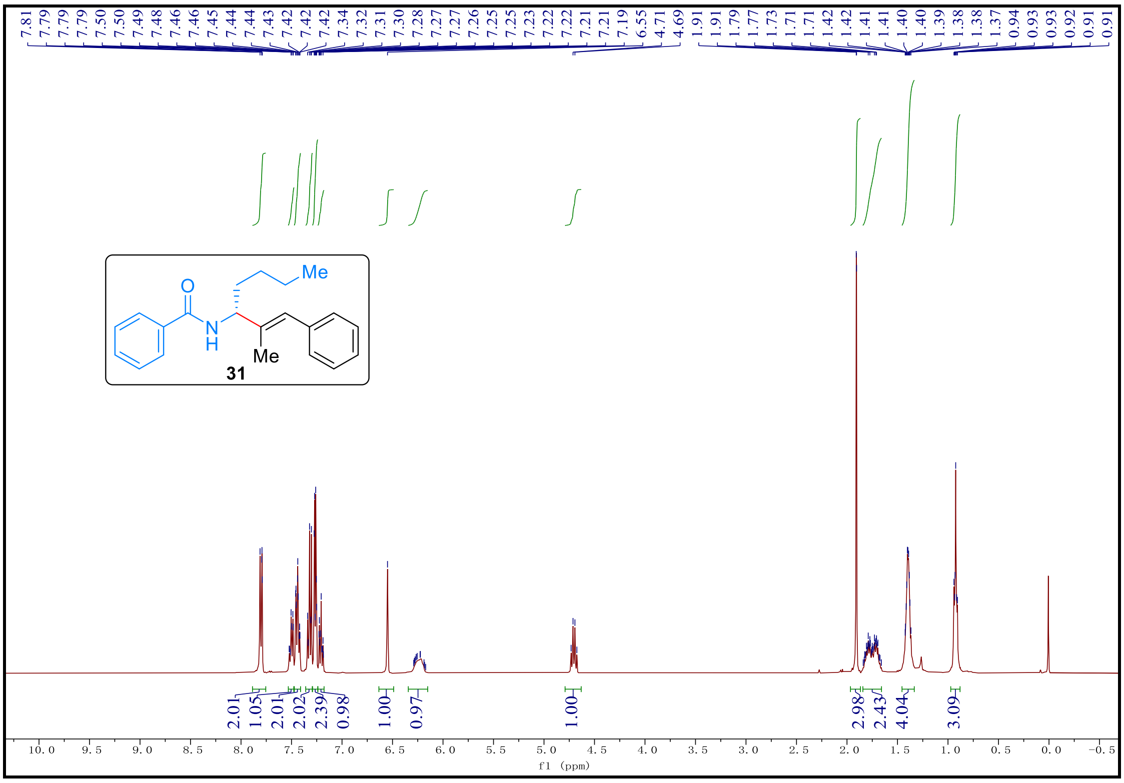

Supplementary Figure 66. ^{1}H NMR Spectrum of Compound **31** (400 MHz, $CDCl_3$, 25 ˚C)

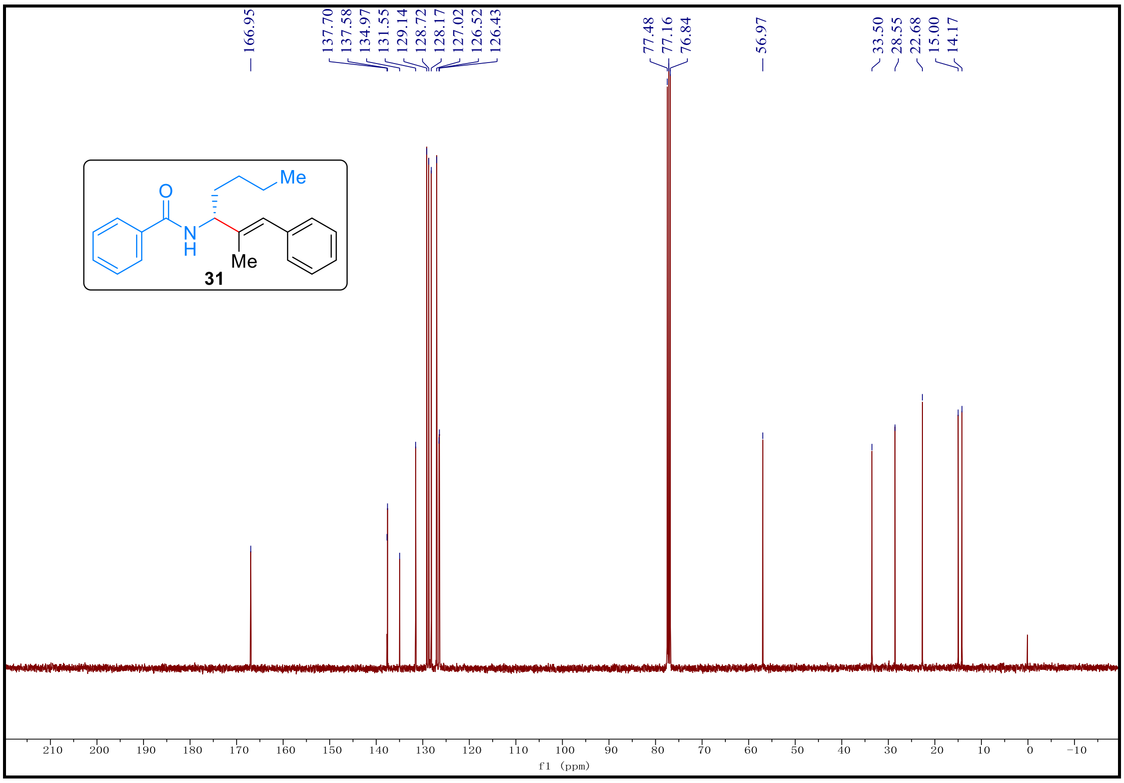

Supplementary Figure 67. ^{13}C NMR Spectrum of Compound **31** (101 MHz, $CDCl_3$, 25 ˚C)

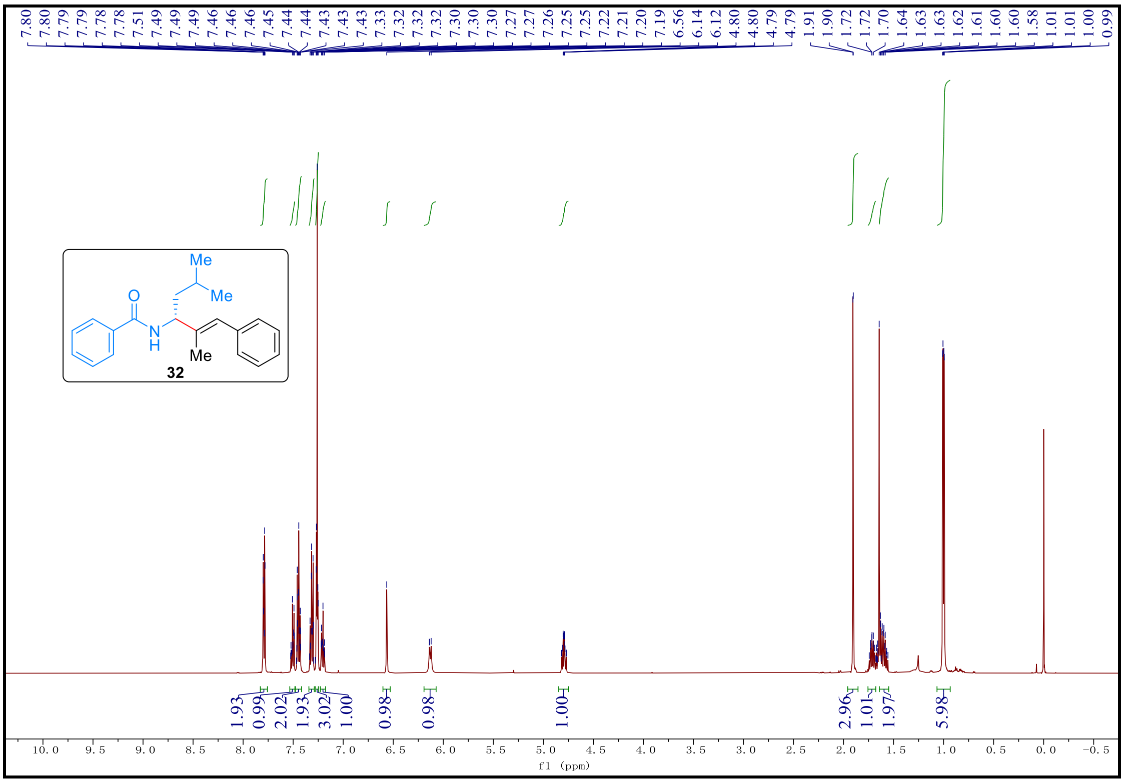

Supplementary Figure 68. ^{1}H NMR Spectrum of Compound **32** (500 MHz, $CDCl_3$, 25 ˚C)

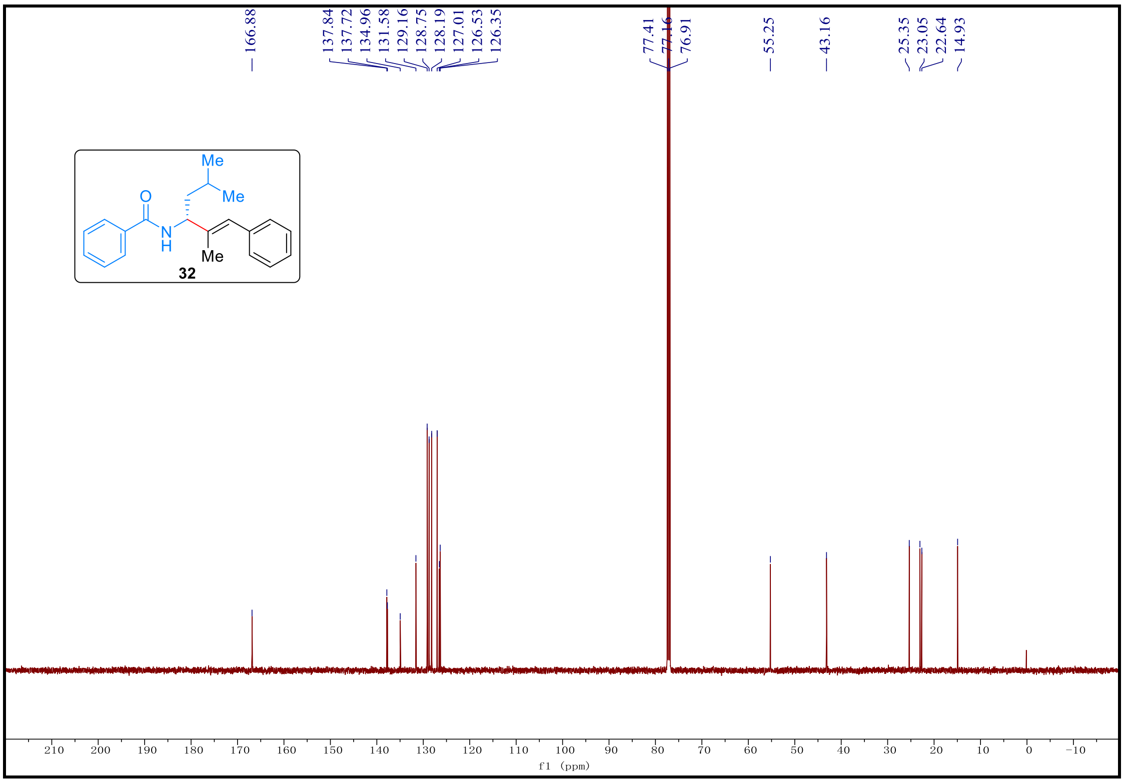

Supplementary Figure 69. ^{13}C NMR Spectrum of Compound **32** (126 MHz, $CDCl_3$, 25 ˚C)

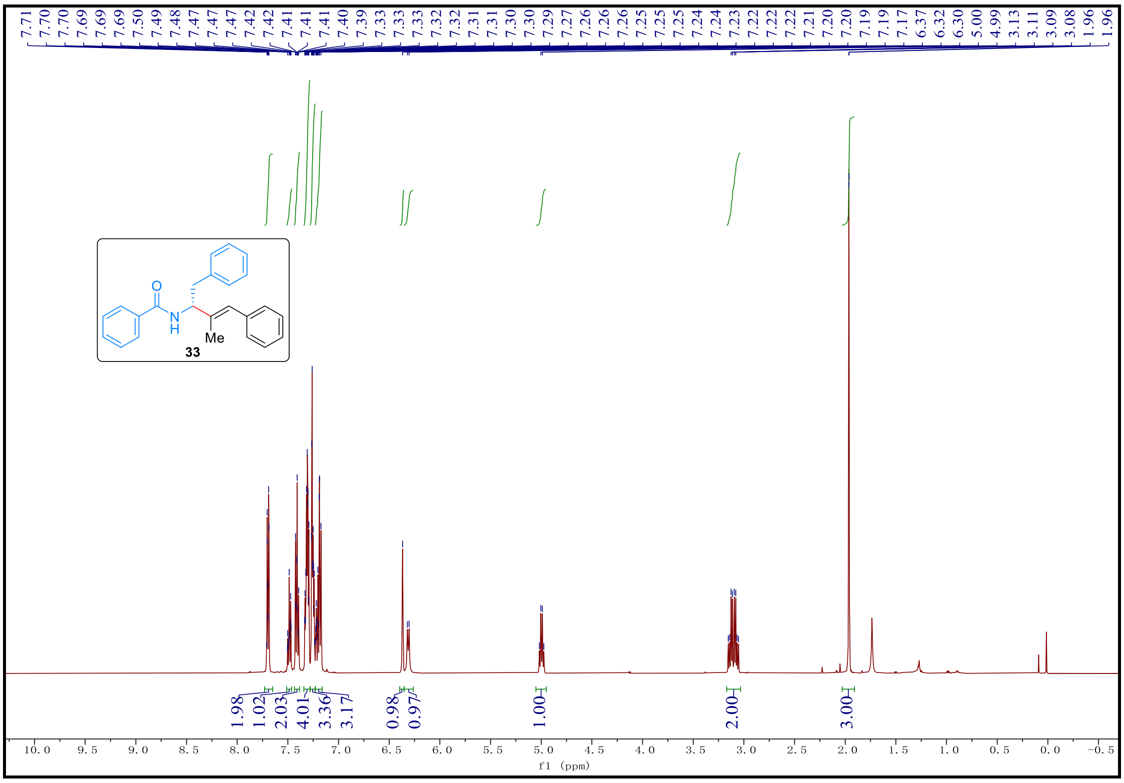

Supplementary Figure 70. ^{1}H NMR Spectrum of Compound **33** (500 MHz, $CDCl_3$, 25 ˚C)

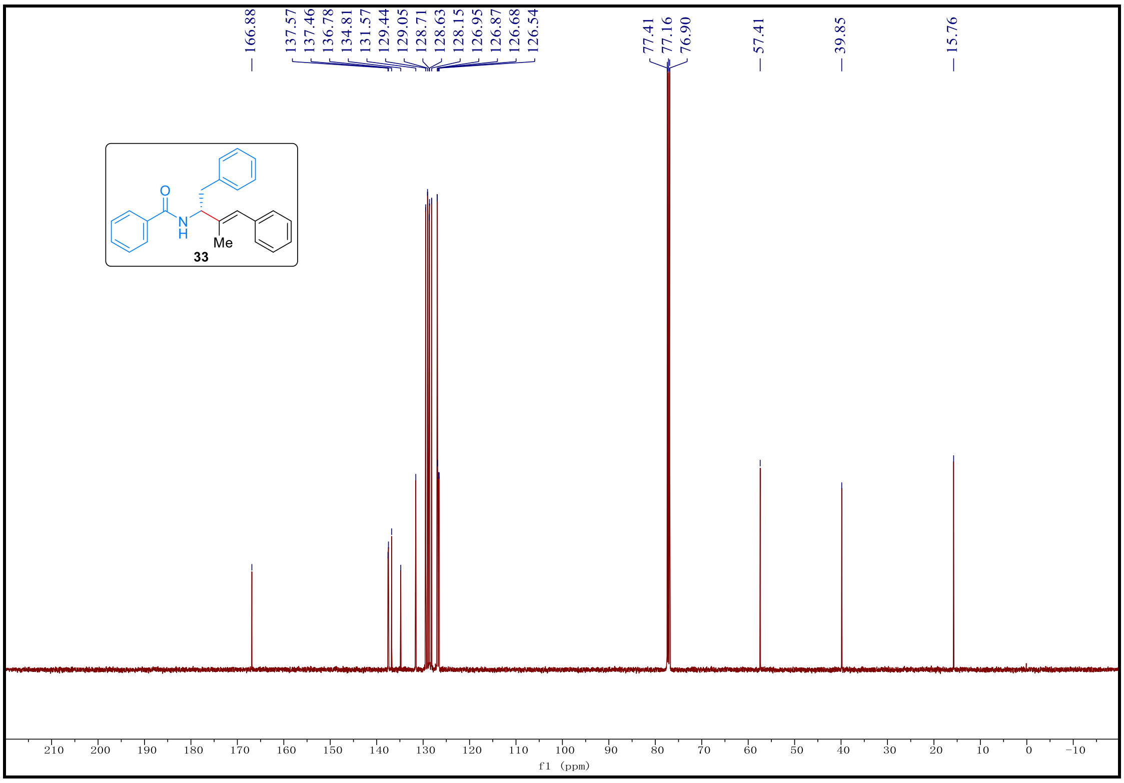

Supplementary Figure 71. ^{13}C NMR Spectrum of Compound **33** (126 MHz, $CDCl_3$, 25 ˚C)

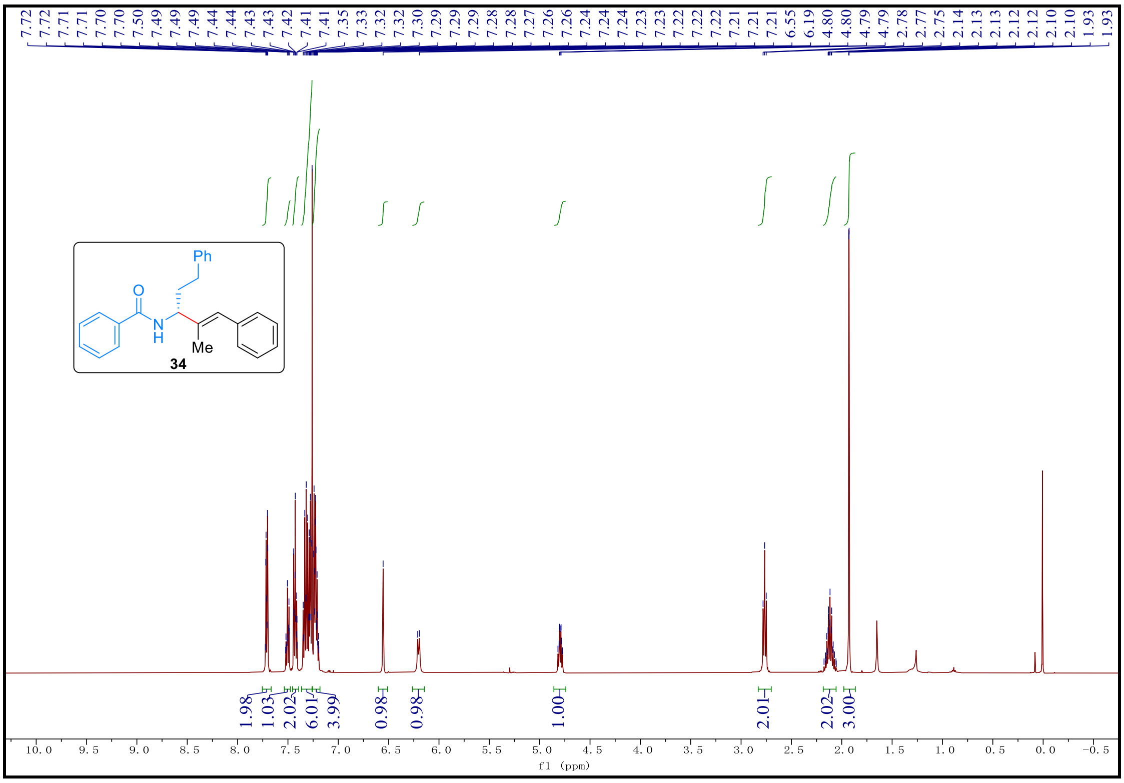

Supplementary Figure 72. ^{1}H NMR Spectrum of Compound **34** (500 MHz, $CDCl_3$, 25 ˚C)

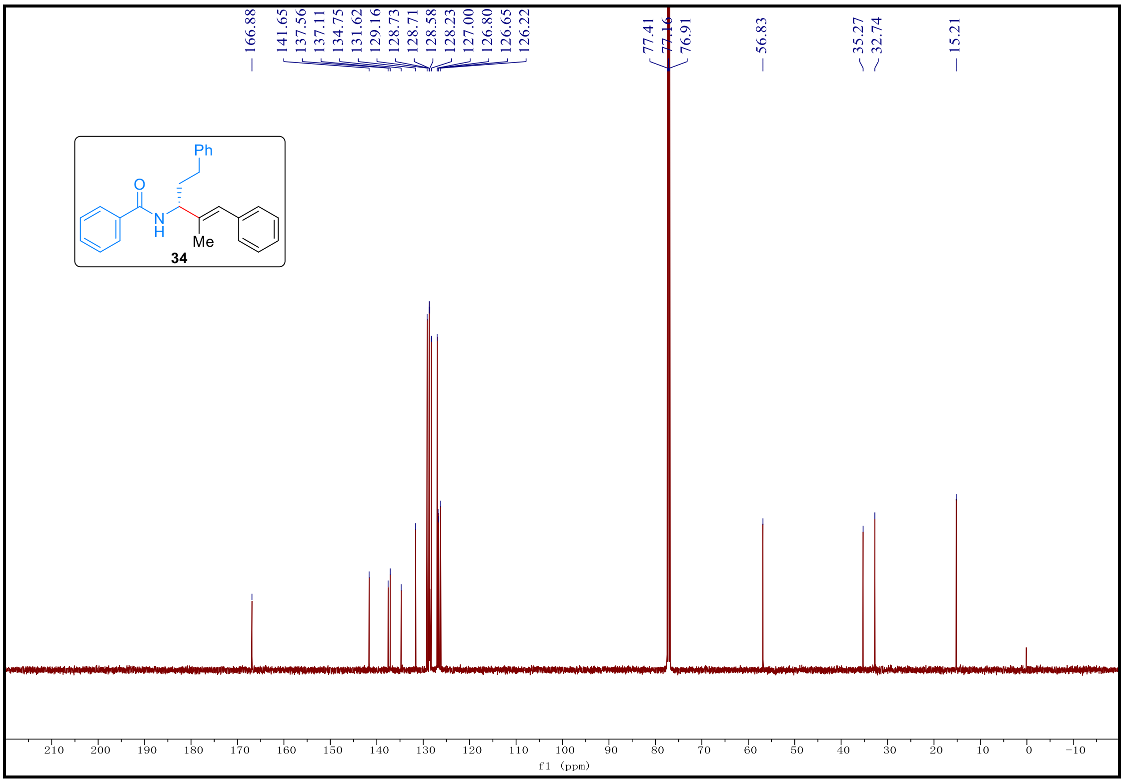

Supplementary Figure 73. ^{13}C NMR Spectrum of Compound **34** (126 MHz, $CDCl_3$, 25 ˚C)

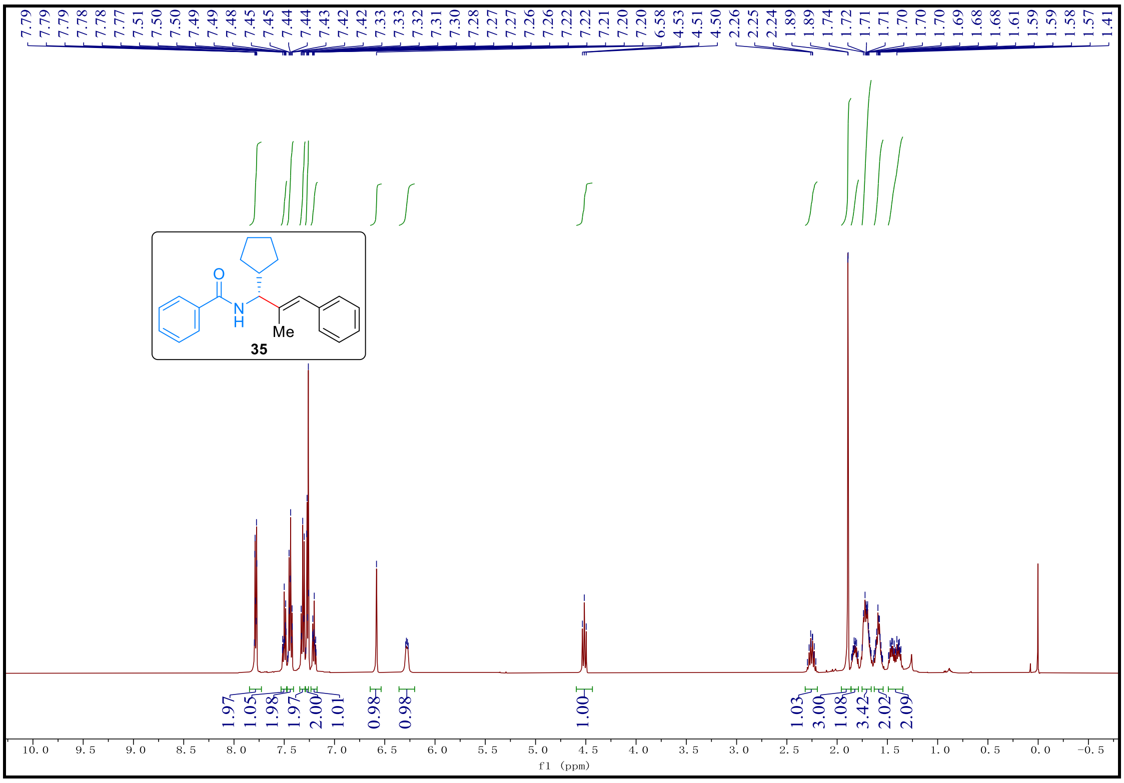

Supplementary Figure 74. ^{1}H NMR Spectrum of Compound **35** (500 MHz, $CDCl_3$, 25 °C)

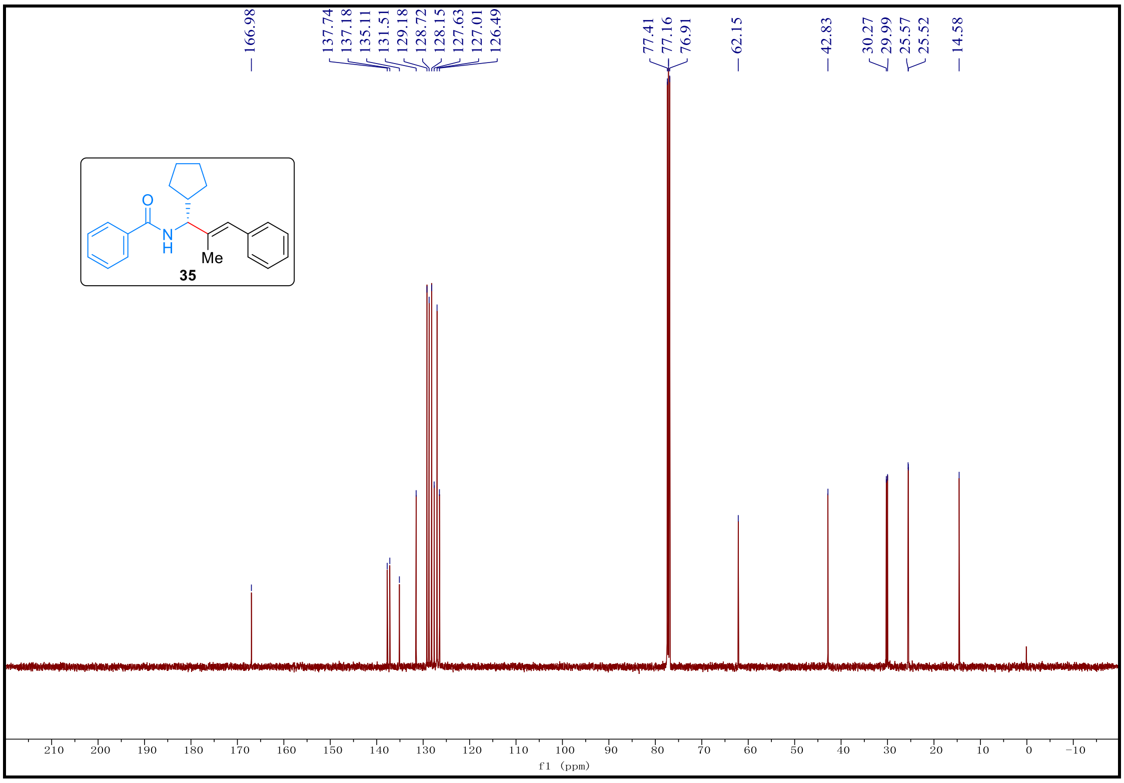

Supplementary Figure 75. ^{13}C NMR Spectrum of Compound **35** (126 MHz, $CDCl_3$, 25 °C)

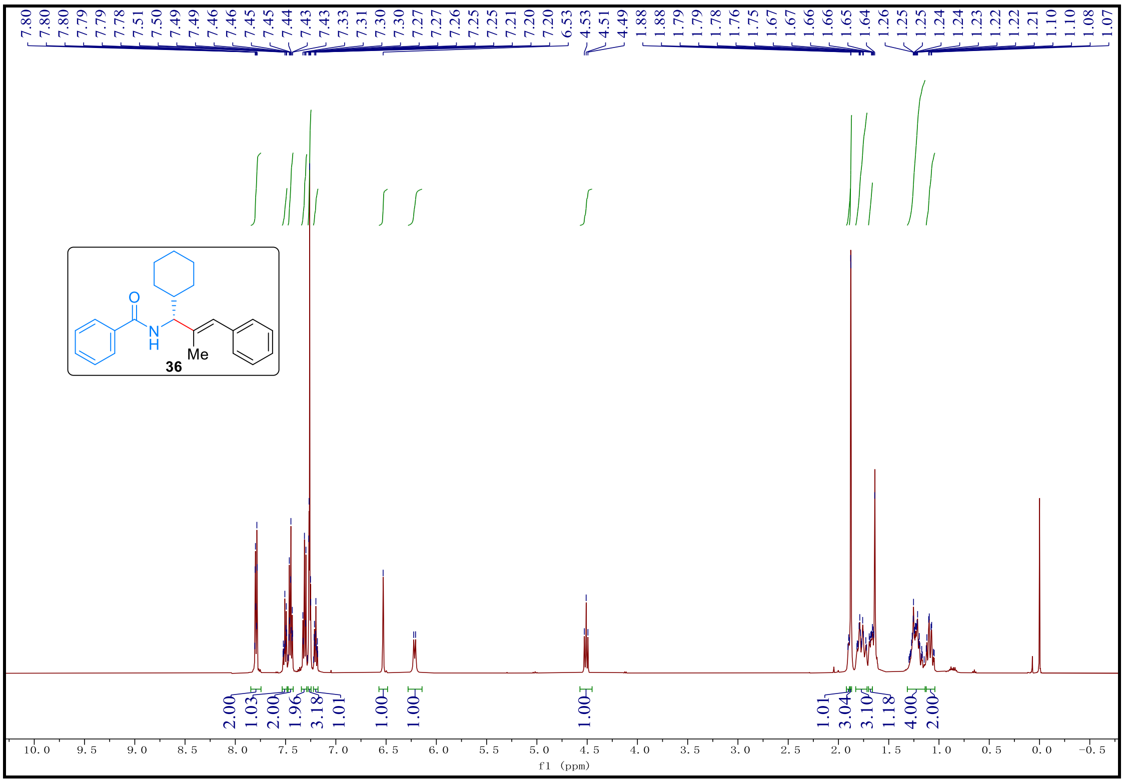

Supplementary Figure 76. ^{1}H NMR Spectrum of Compound **36** (500 MHz, $CDCl_3$, 25 °C)

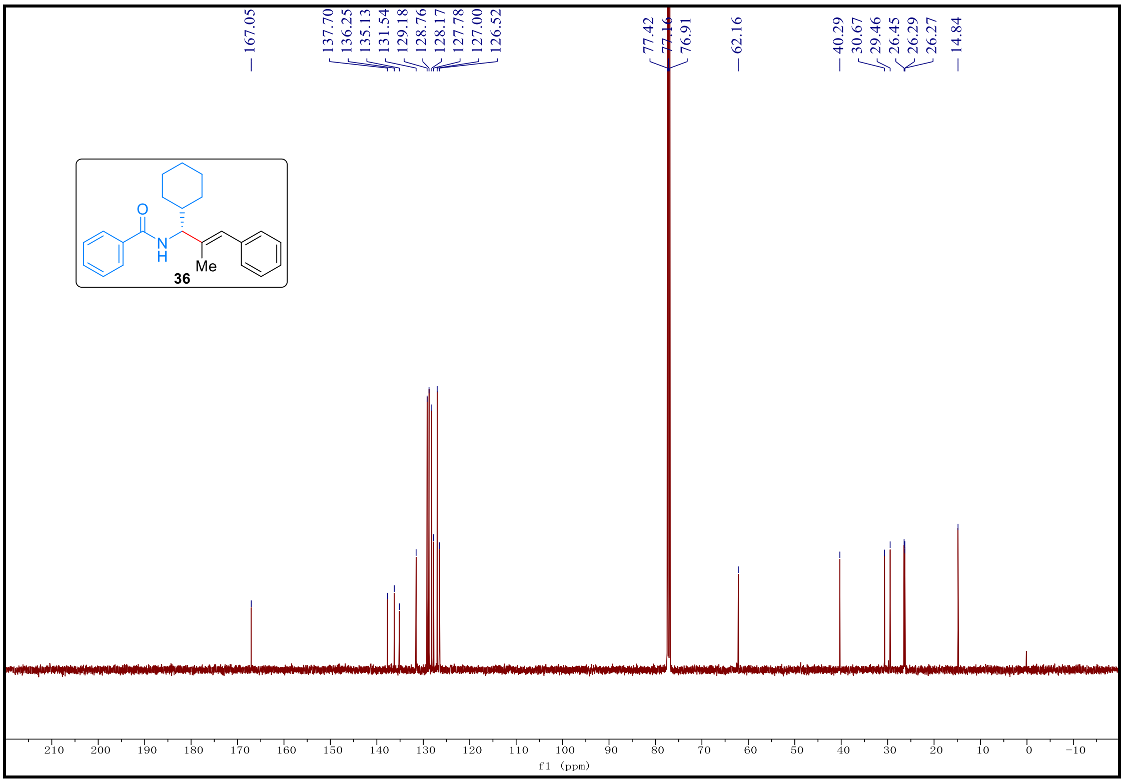

Supplementary Figure 77. ^{13}C NMR Spectrum of Compound **36** (126 MHz, $CDCl_3$, 25 °C)

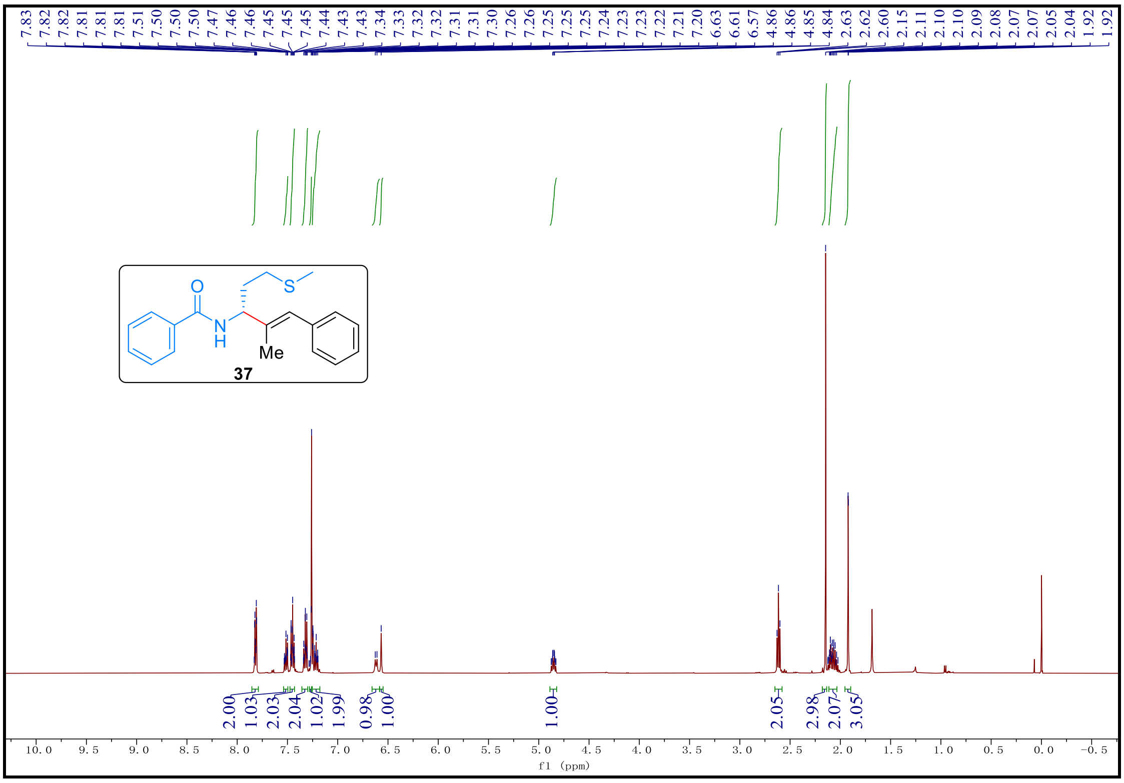

Supplementary Figure 78. ^{1}H NMR Spectrum of Compound **37** (500 MHz, $CDCl_3$, 25 ˚C)

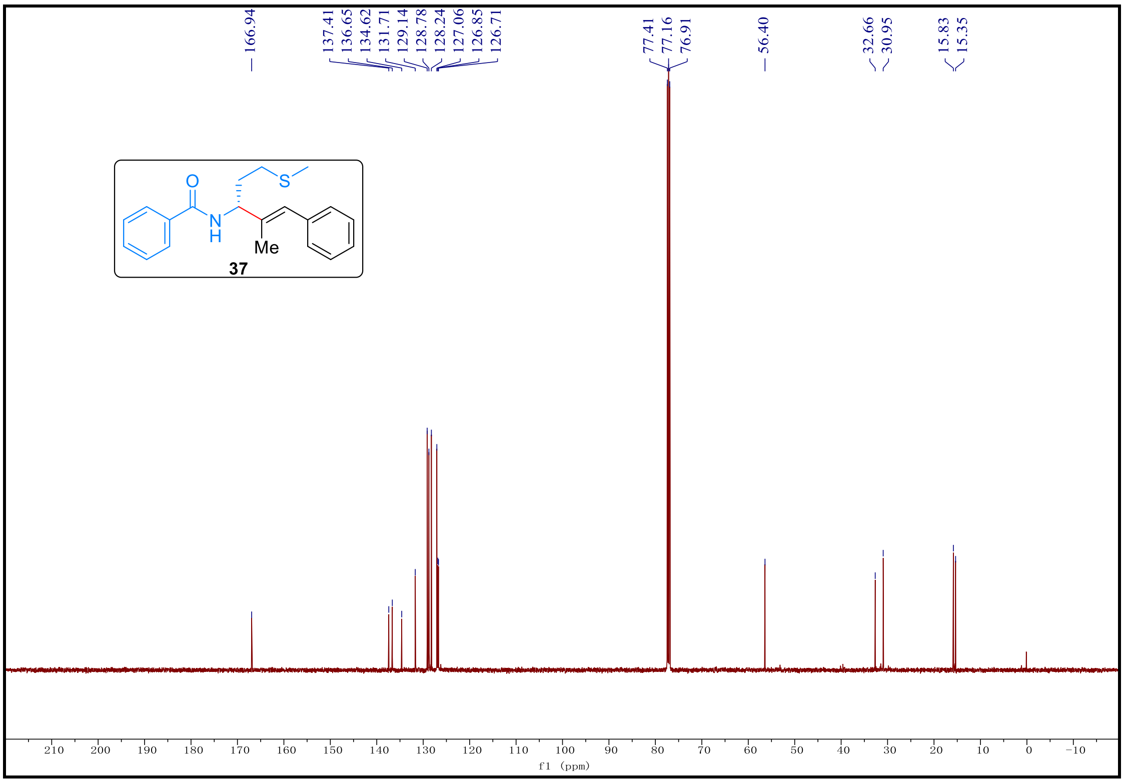

Supplementary Figure 79. ^{13}C NMR Spectrum of Compound **37** (126 MHz, $CDCl_3$, 25 ˚C)

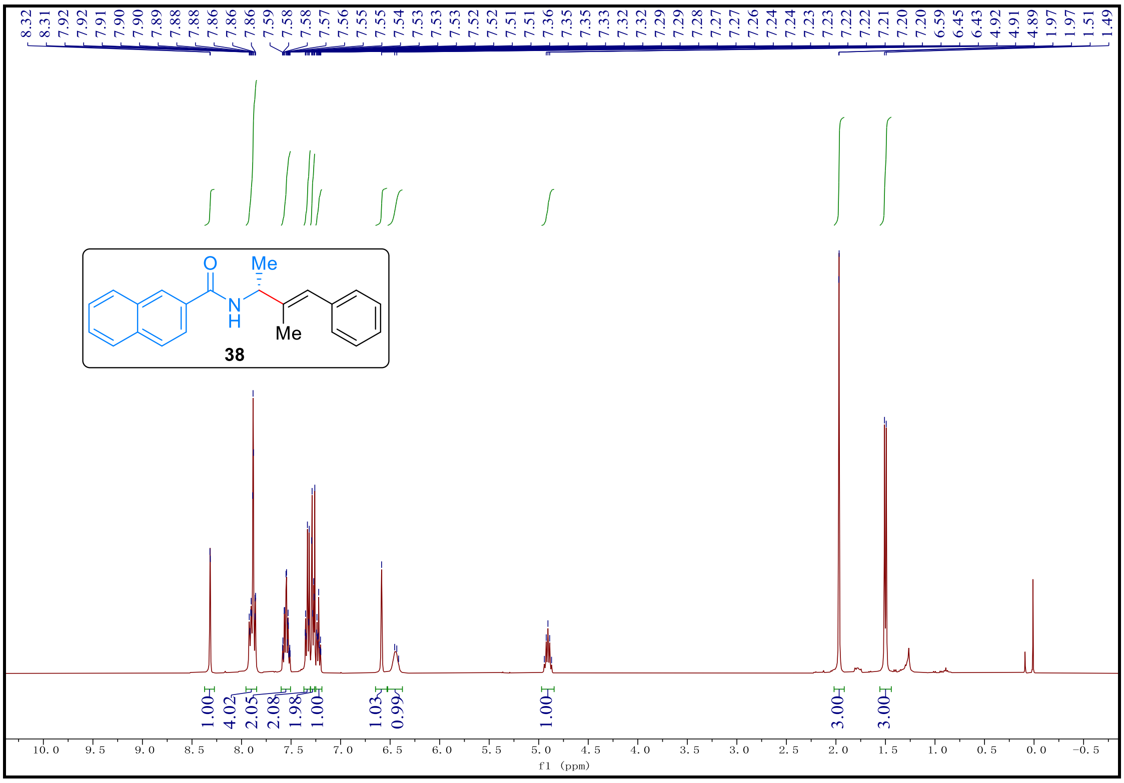

Supplementary Figure 80. ^{1}H NMR Spectrum of Compound **38** (400 MHz, $CDCl_3$, 25 ˚C)

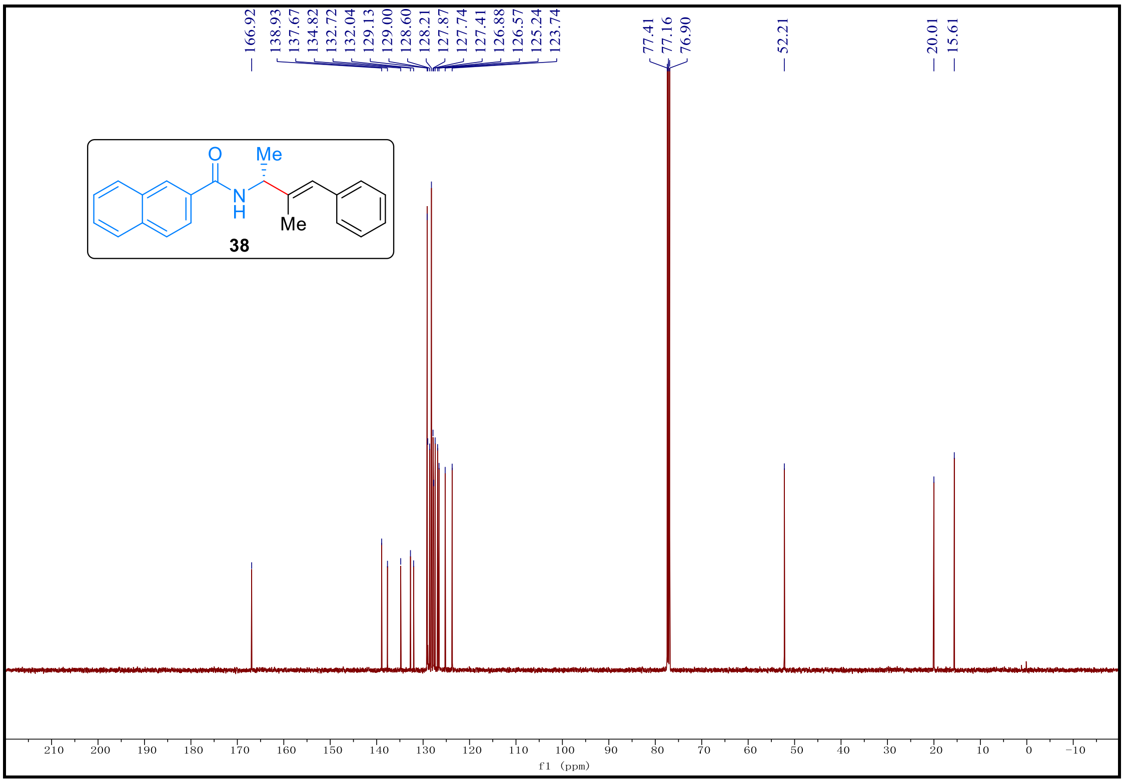

Supplementary Figure 81. ^{13}C NMR Spectrum of Compound **38** (126 MHz, $CDCl_3$, 25 ˚C)

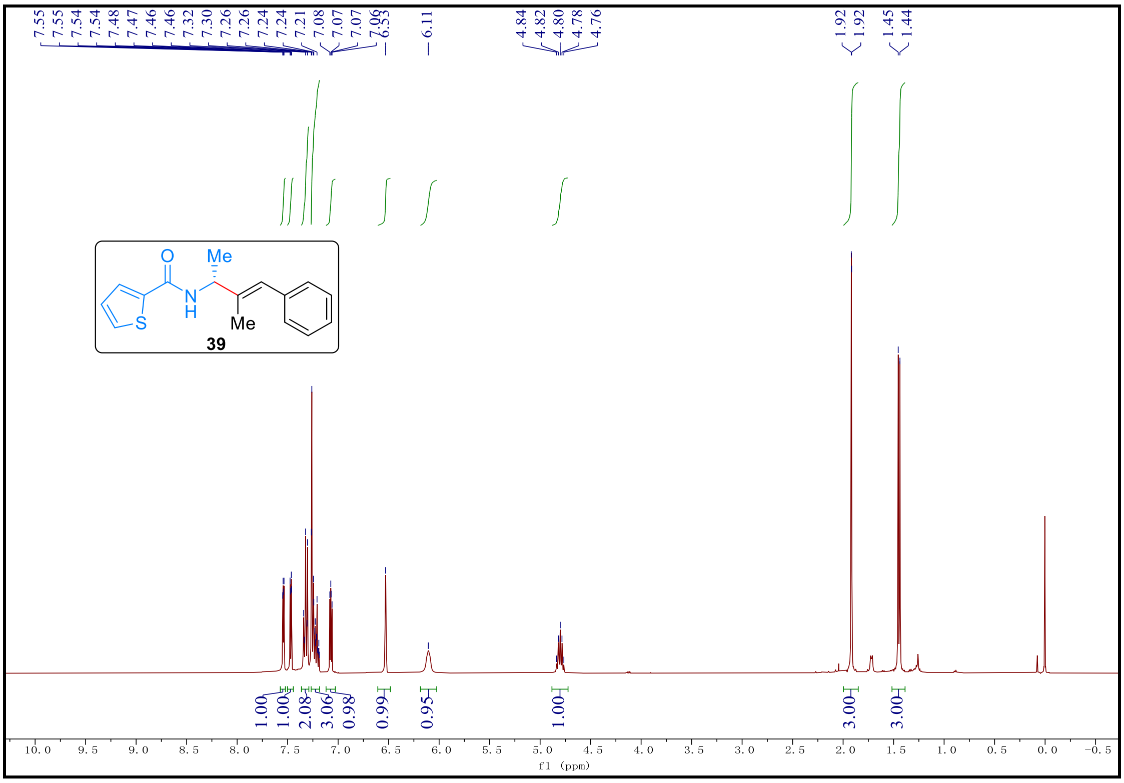

Supplementary Figure 82. ^{1}H NMR Spectrum of Compound **39** (400 MHz, $CDCl_3$, 25 ˚C)

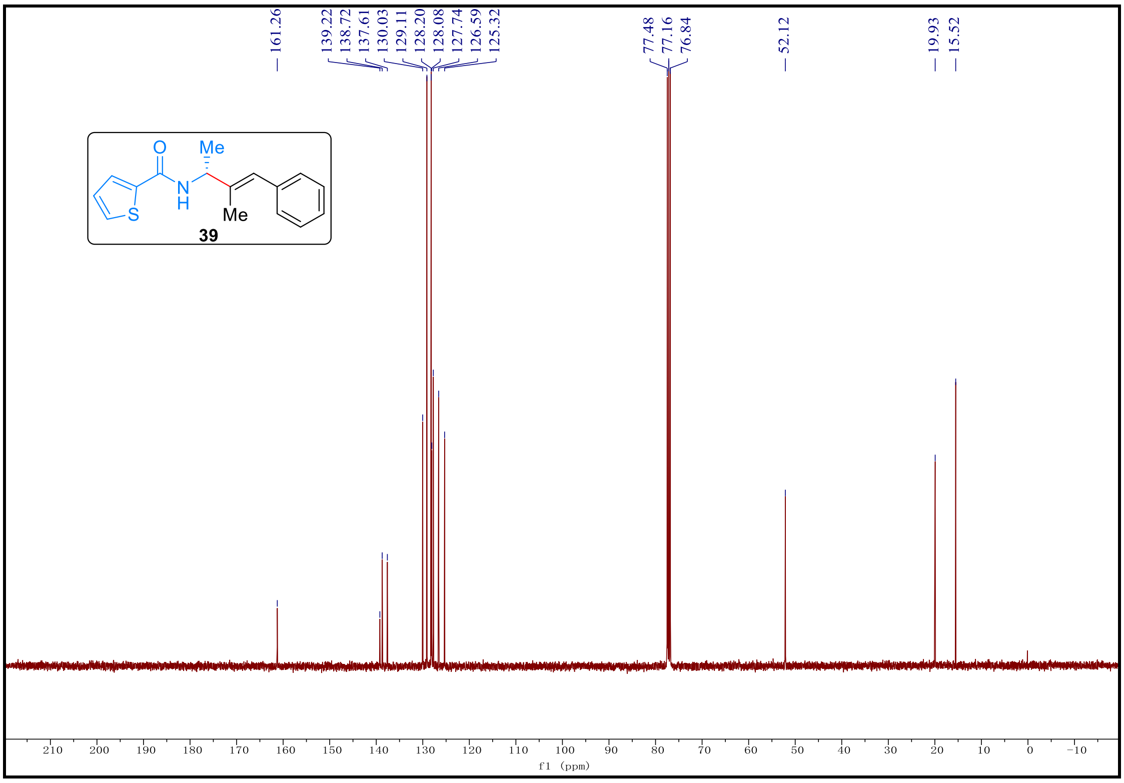

Supplementary Figure 83. ^{13}C NMR Spectrum of Compound **39** (101 MHz, $CDCl_3$, 25 ˚C)

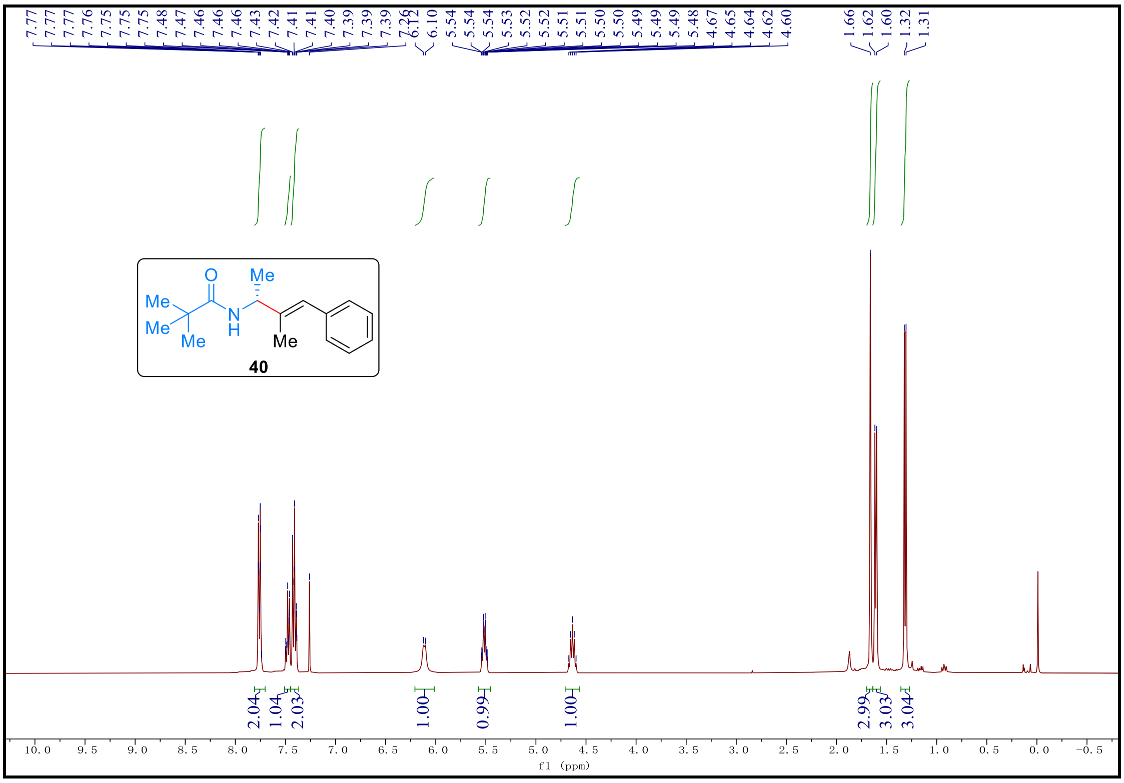

Supplementary Figure 84. ^{1}H NMR Spectrum of Compound **40** (400 MHz, $CDCl_3$, 25 °C)

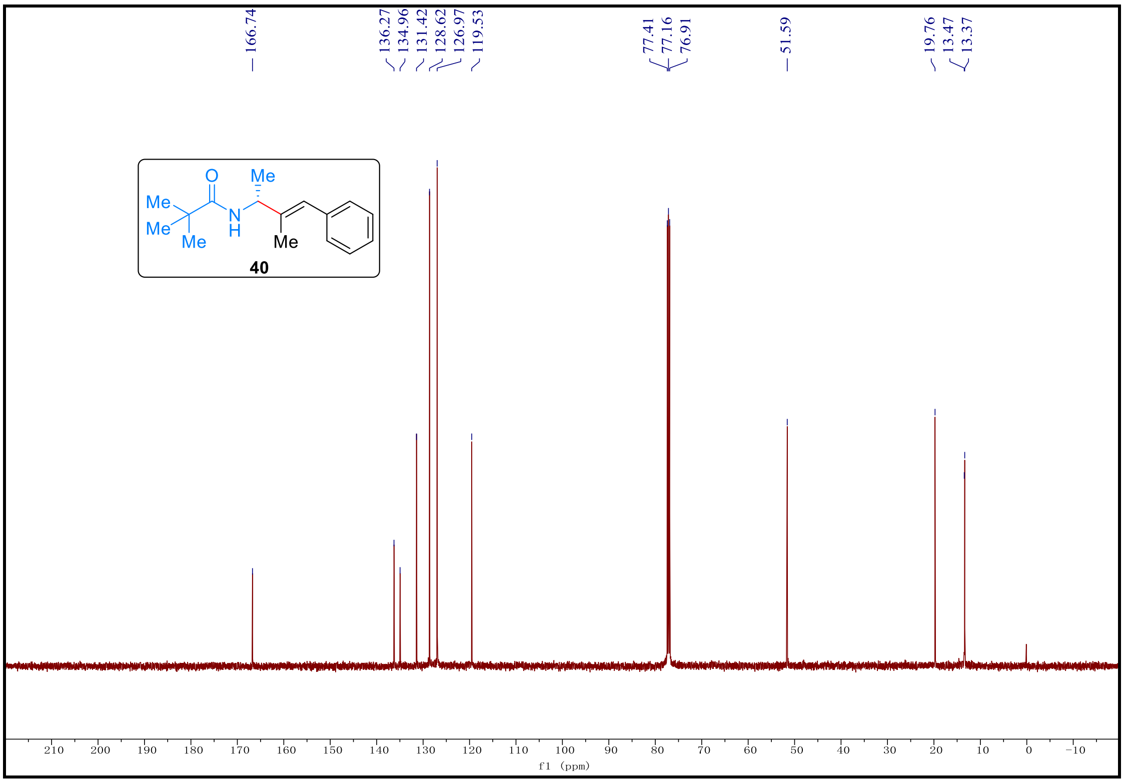

Supplementary Figure 85. ^{13}C NMR Spectrum of Compound **40** (101 MHz, $CDCl_3$, 25 °C)

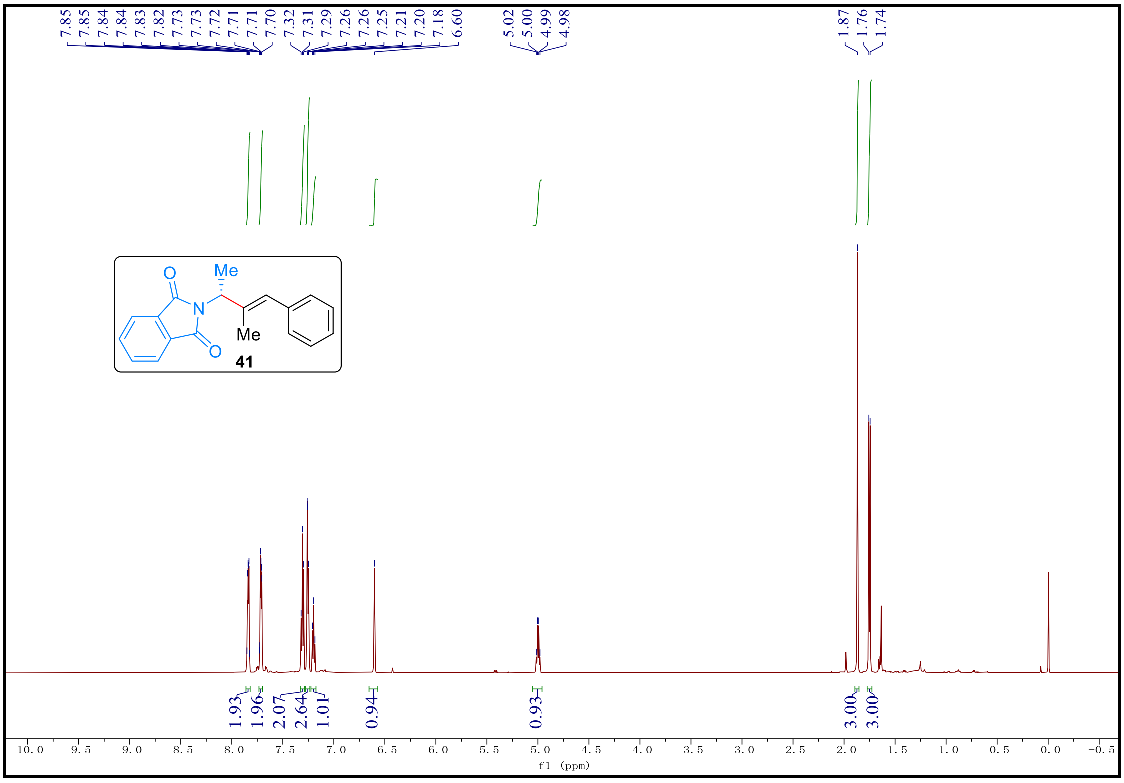

Supplementary Figure 86. ^{1}H NMR Spectrum of Compound **41** (600 MHz, $CDCl_3$, 25 °C)

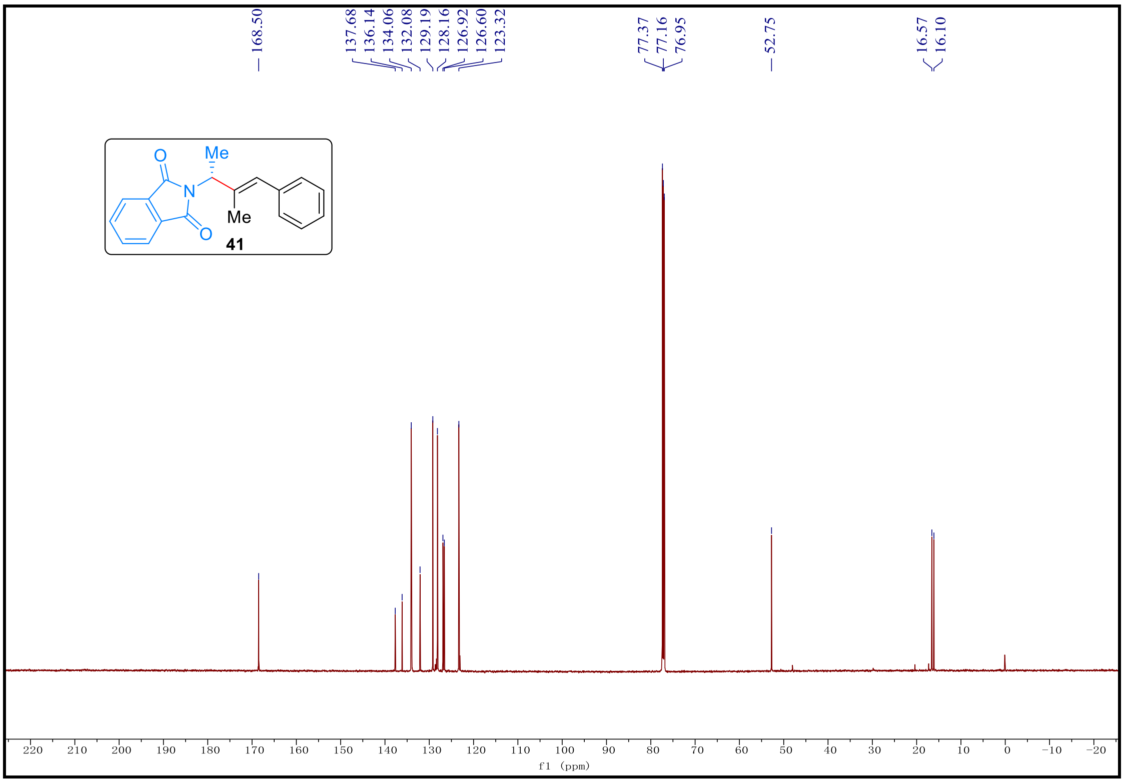

Supplementary Figure 87. ^{13}C NMR Spectrum of Compound **41** (151 MHz, $CDCl_3$, 25 °C)

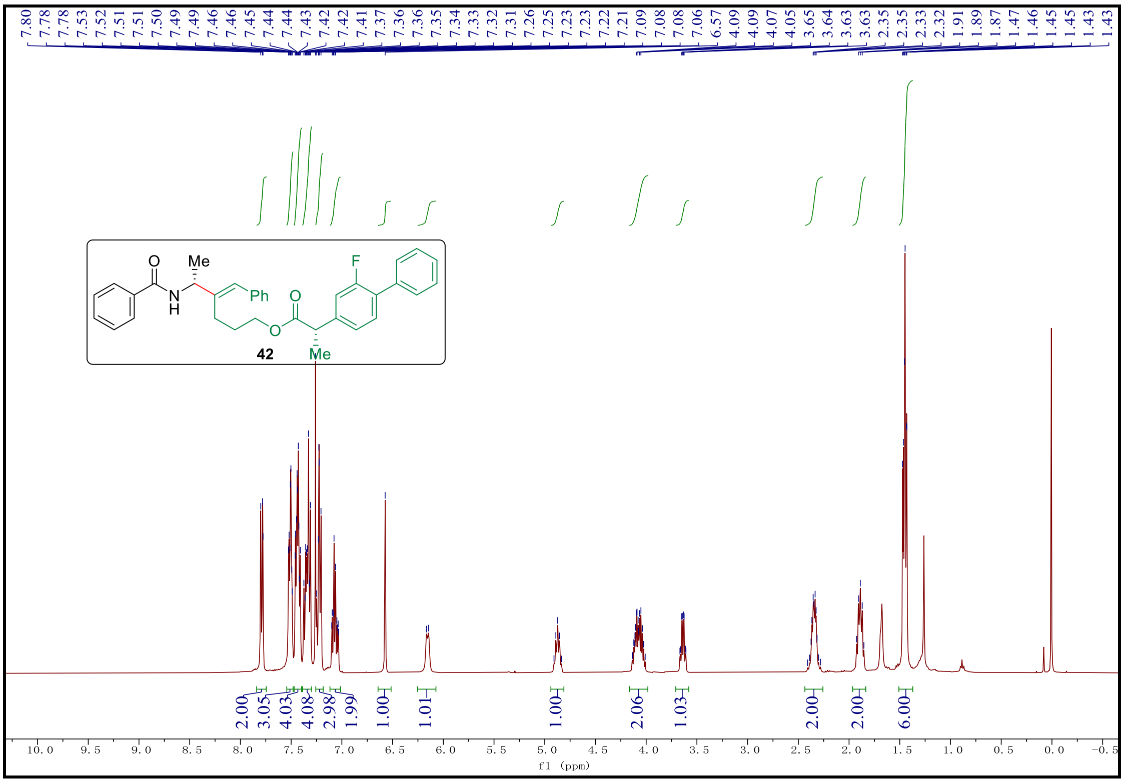

Supplementary Figure 88. ^{1}H NMR Spectrum of Compound **42** (400 MHz, $CDCl_3$, 25 ˚C)

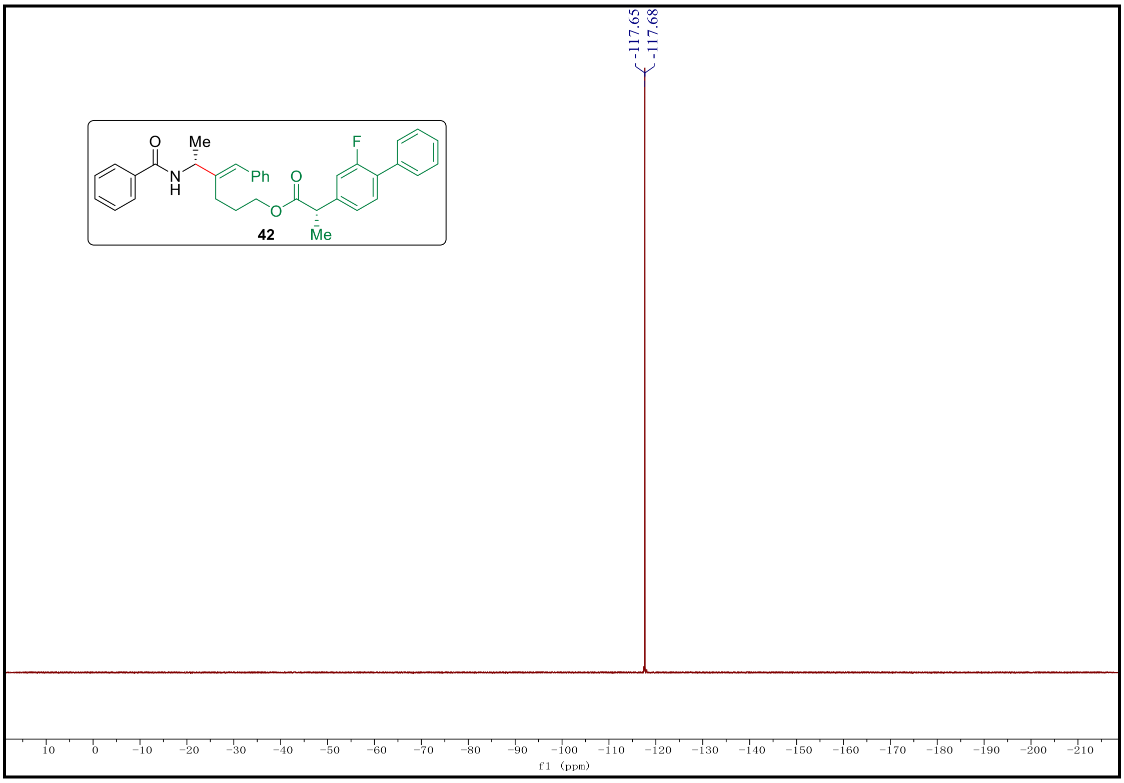

Supplementary Figure 89. ^{19}F NMR Spectrum of Compound **42** (376 MHz, $CDCl_3$, 25 ˚C)

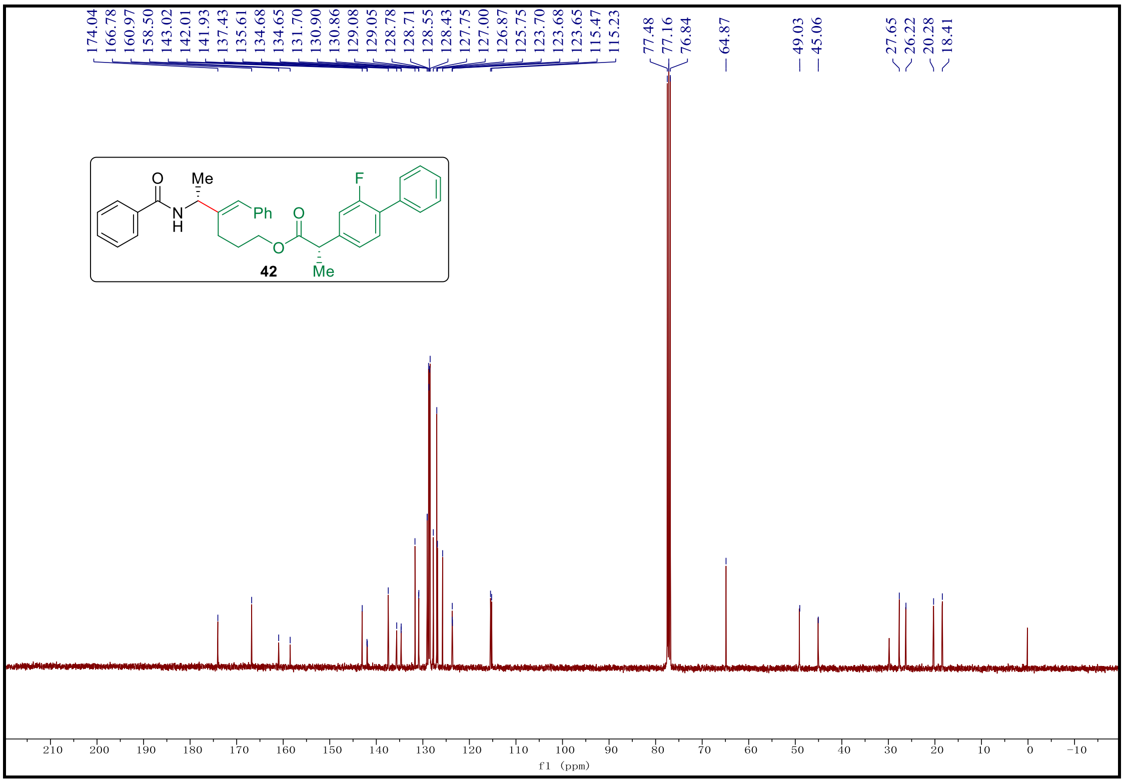

Supplementary Figure 90. ^{13}C NMR Spectrum of Compound **42** (101 MHz, $CDCl_3$, 25 ˚C)

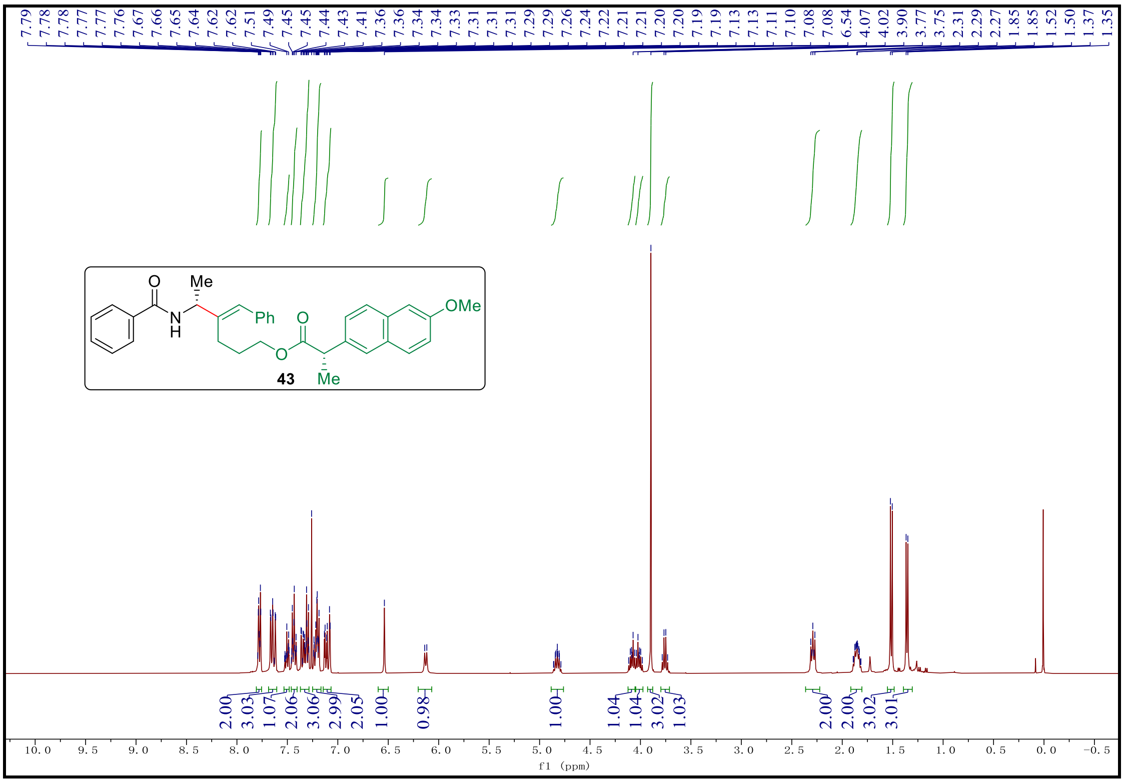

Supplementary Figure 91. ^{1}H NMR Spectrum of Compound **43** (400 MHz, $CDCl_3$, 25 ˚C)

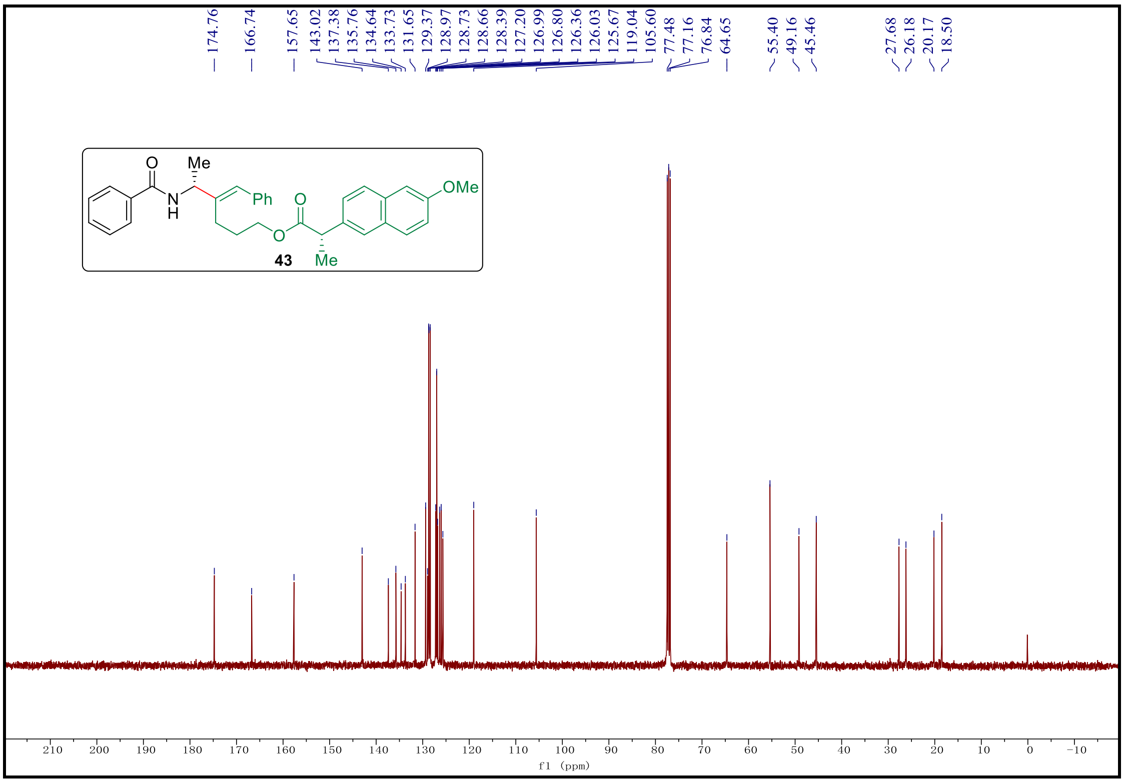

Supplementary Figure 92. ^{13}C NMR Spectrum of Compound **43** (101 MHz, $CDCl_3$, 25 ˚C)

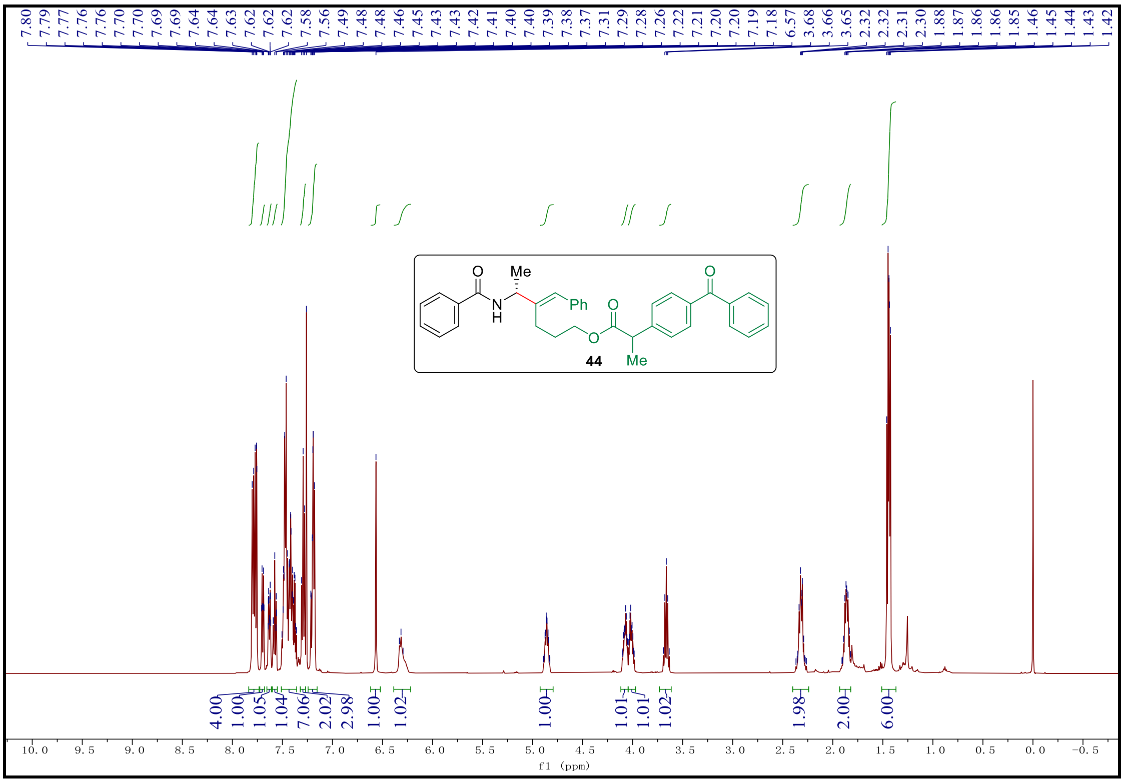

Supplementary Figure 93. ^{1}H NMR Spectrum of Compound **44** (500 MHz, $CDCl_3$, 25 ˚C)

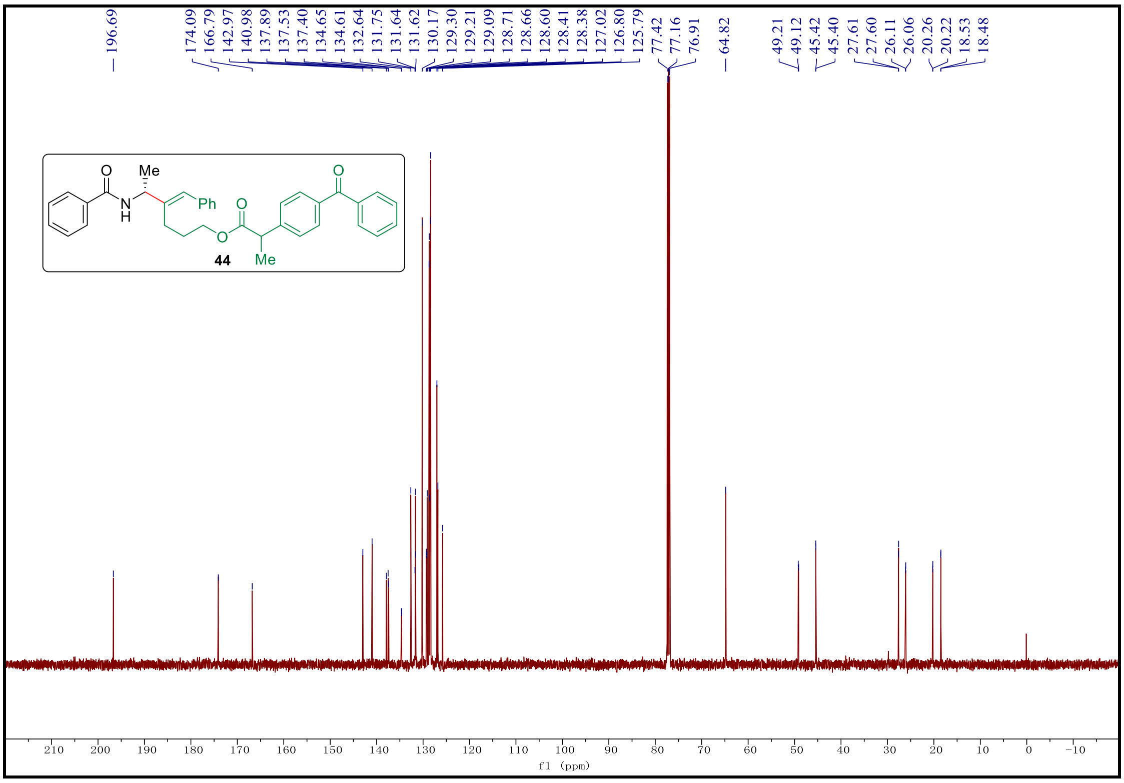

Supplementary Figure 94. ^{13}C NMR Spectrum of Compound **44** (126 MHz, CDCl$_3$, 25 ˚C)

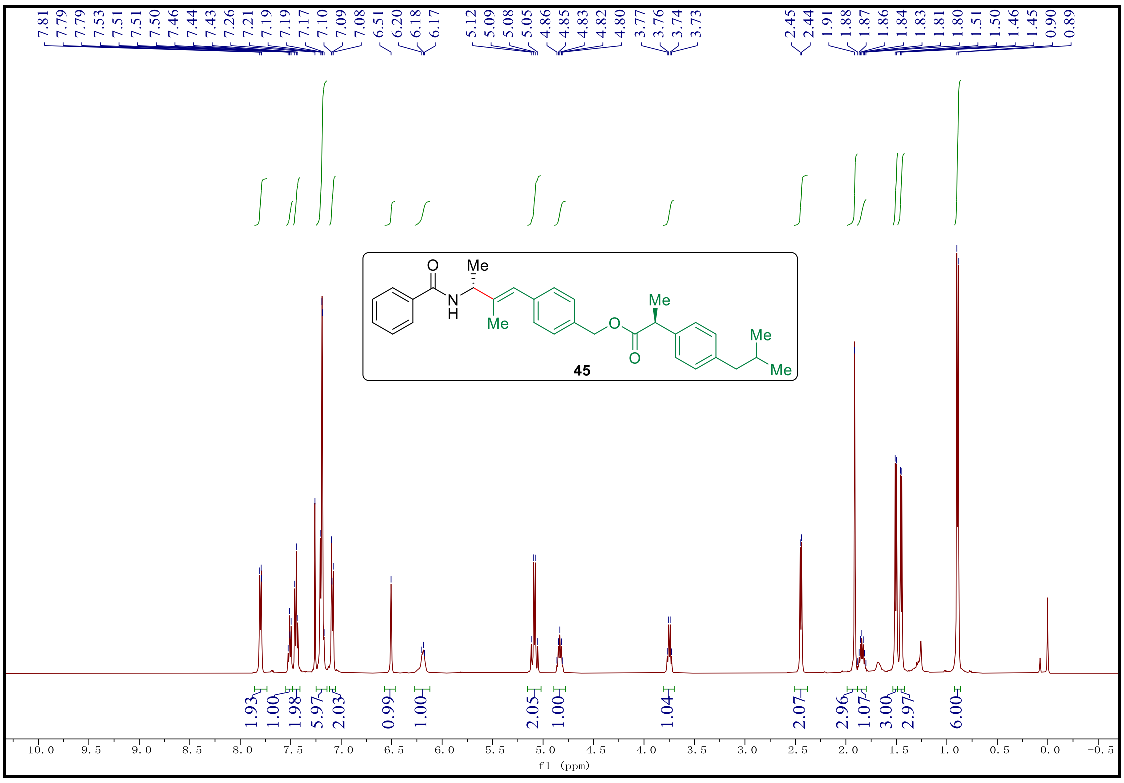

Supplementary Figure 95. ^{1}H NMR Spectrum of Compound **45** (500 MHz, CDCl$_3$, 25 ˚C)

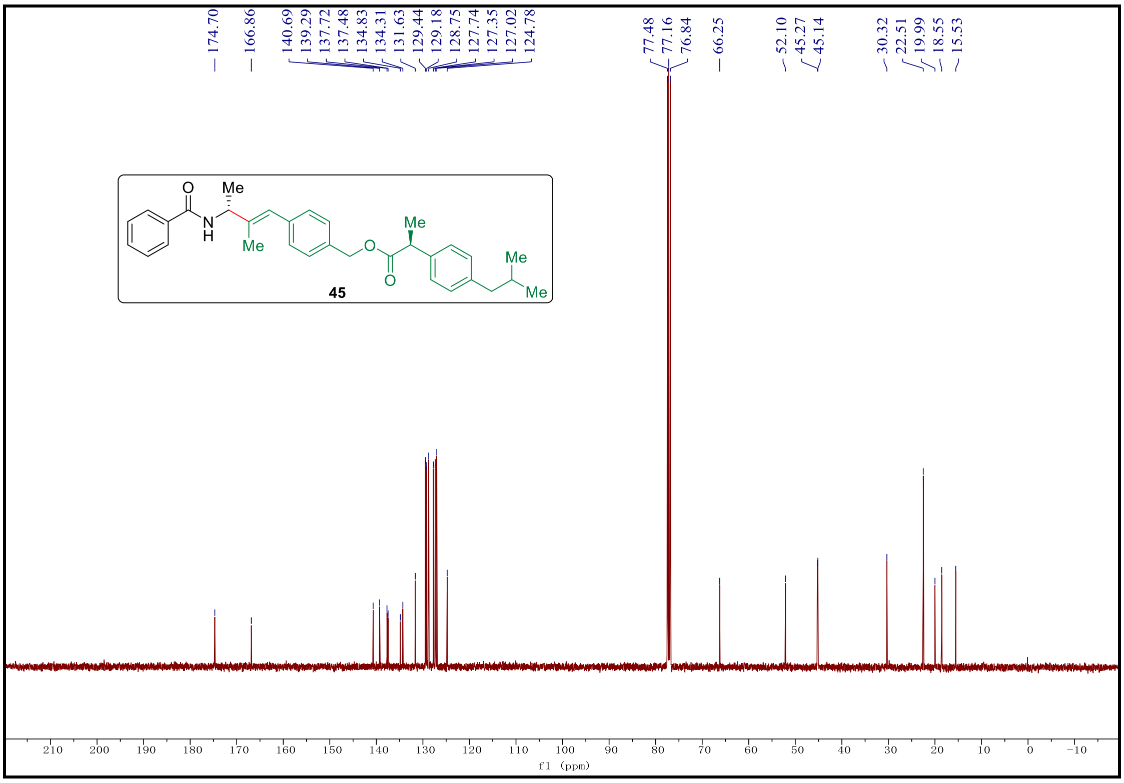

Supplementary Figure 96. ^{13}C NMR Spectrum of Compound **45** (101 MHz, $CDCl_3$, 25 ˚C)

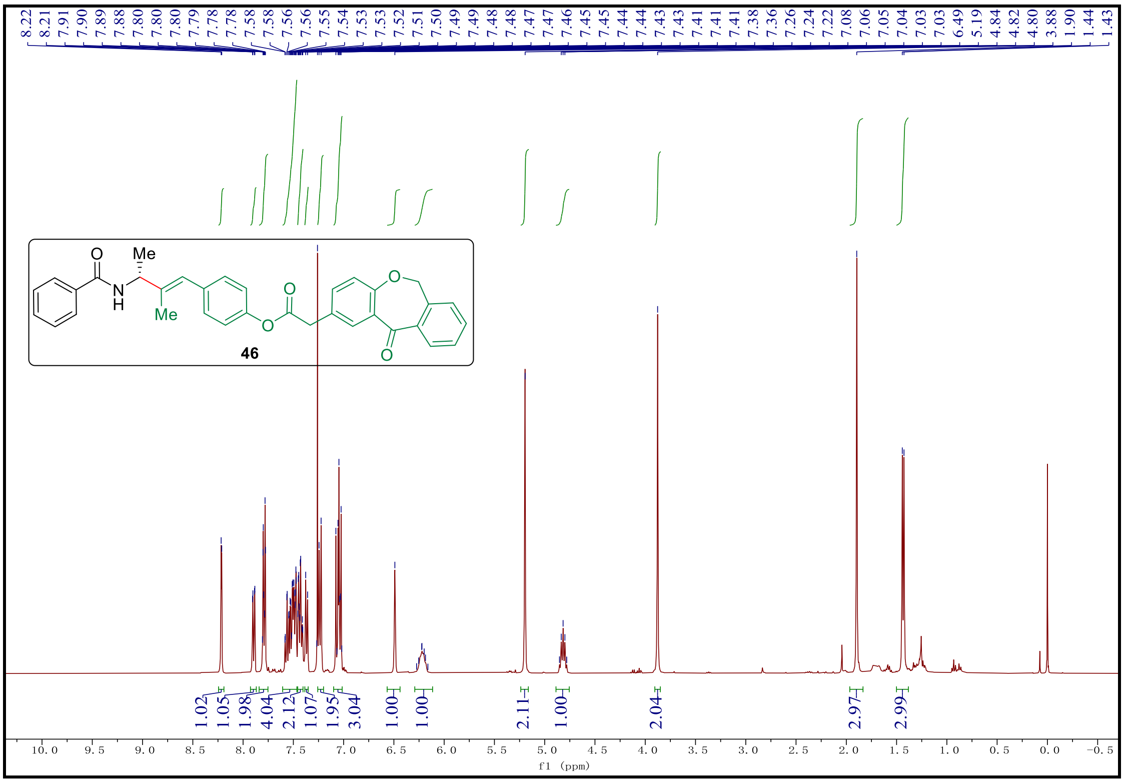

Supplementary Figure 97. ^{1}H NMR Spectrum of Compound **46** (400 MHz, $CDCl_3$, 25 ˚C)

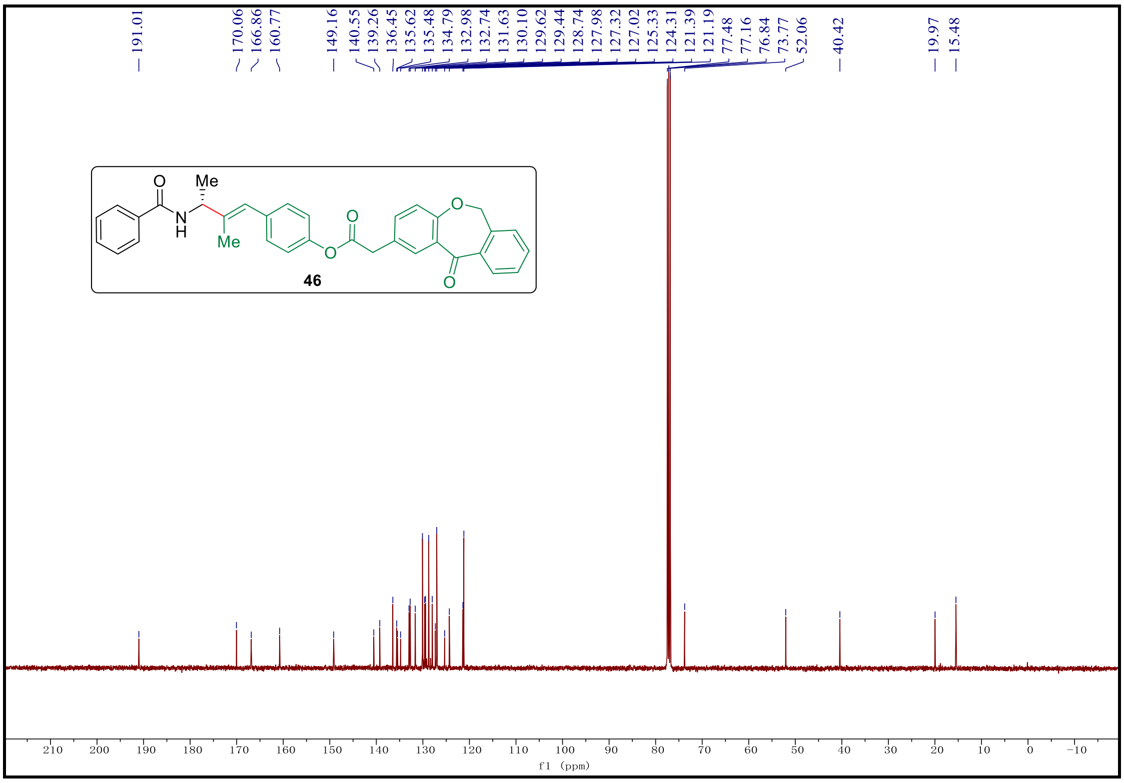

Supplementary Figure 98. ^{13}C NMR Spectrum of Compound **46** (101 MHz, $CDCl_3$, 25 ˚C)

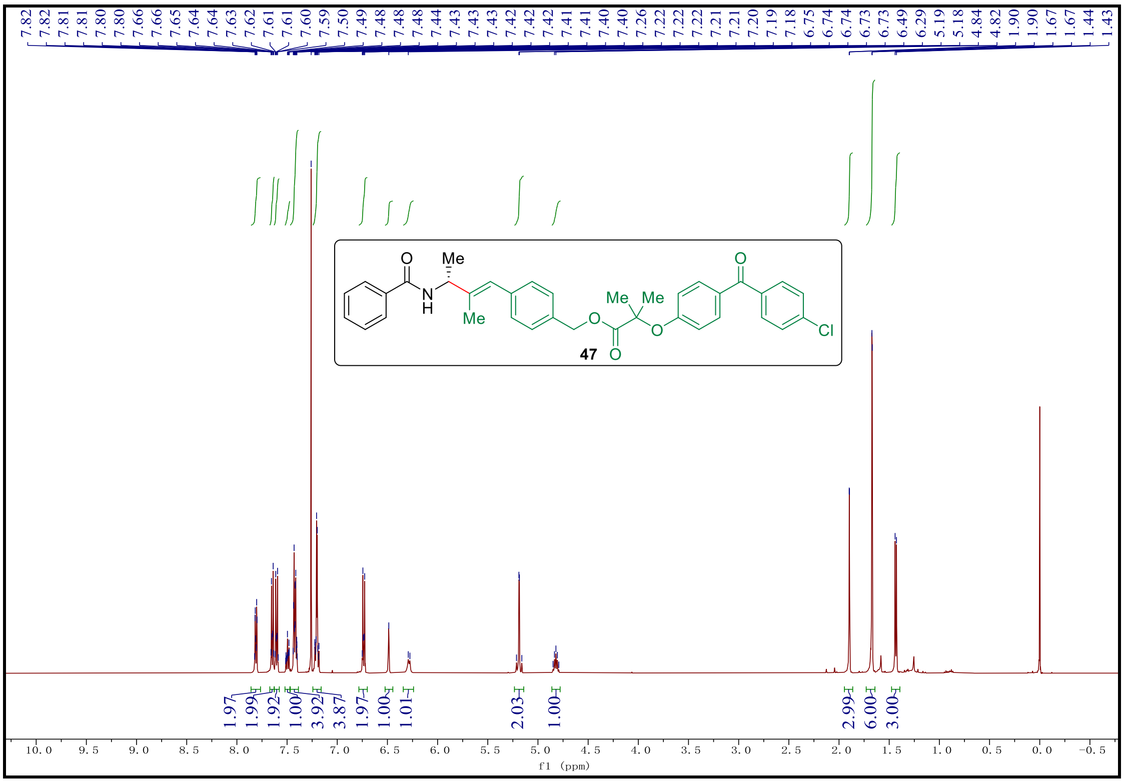

Supplementary Figure 99. ^{1}H NMR Spectrum of Compound **47** (500 MHz, $CDCl_3$, 25 ˚C)

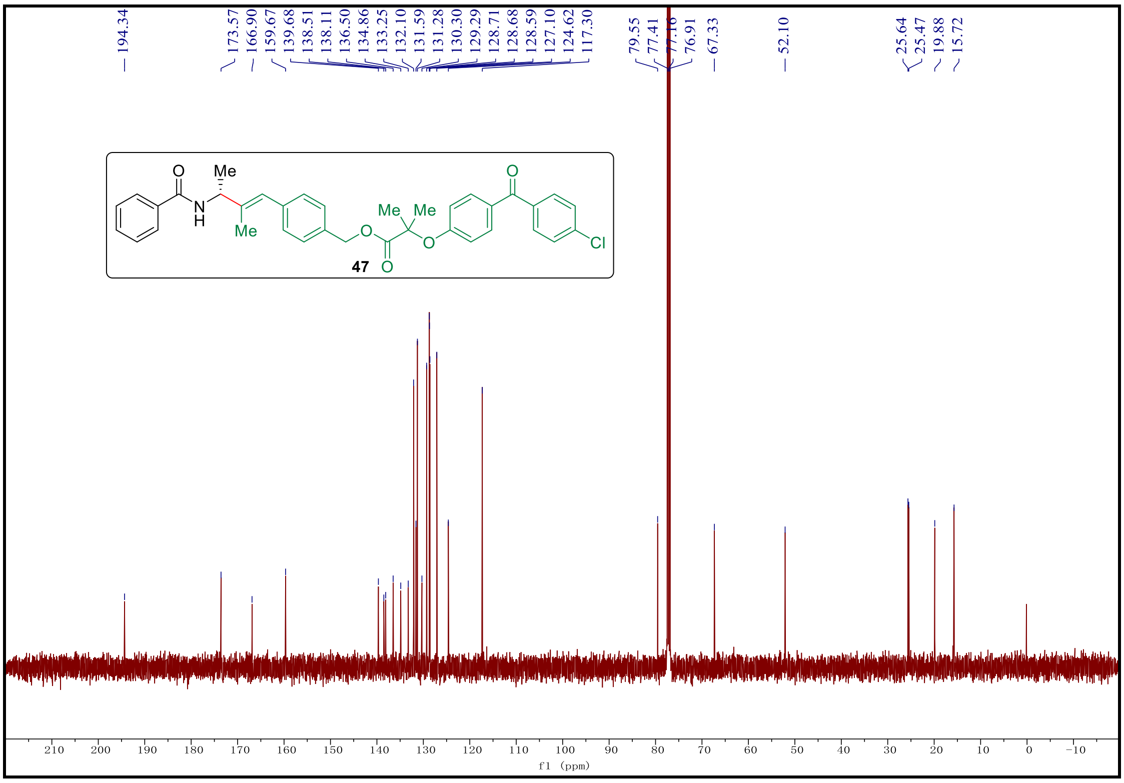

Supplementary Figure 100. ^{13}C NMR Spectrum of Compound **47** (126 MHz, $CDCl_3$, 25 ˚C)

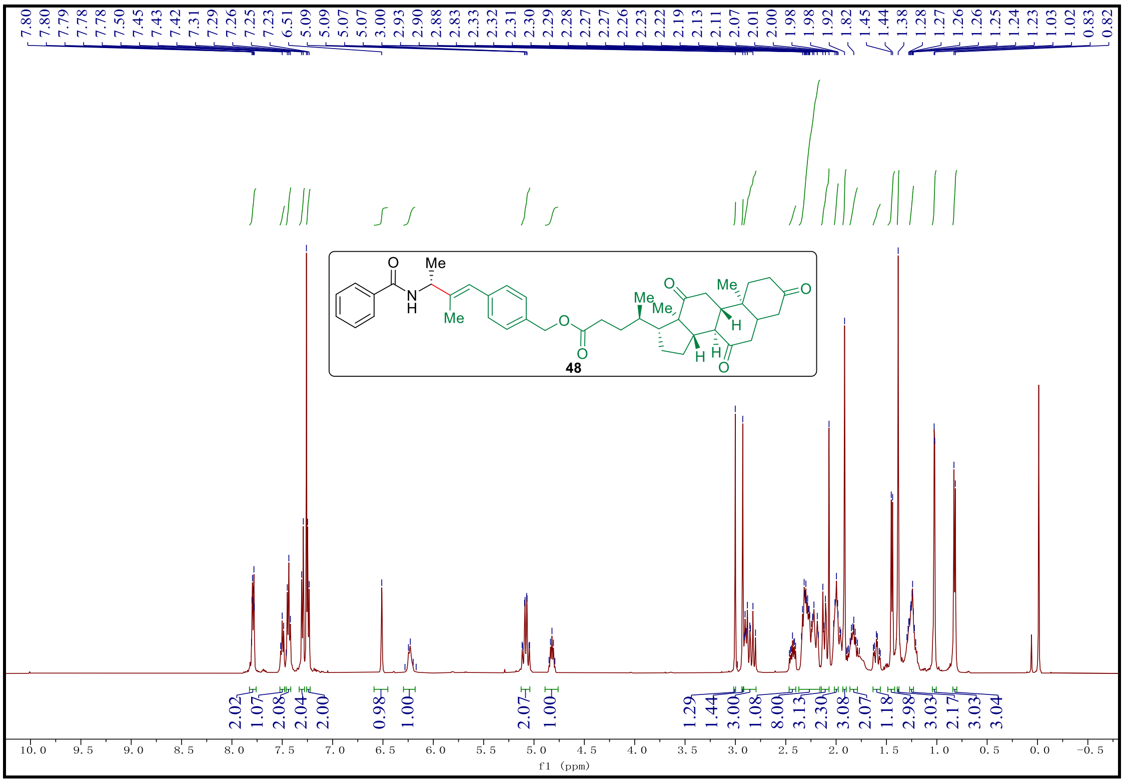

Supplementary Figure 101. ^{1}H NMR Spectrum of Compound **48** (500 MHz, $CDCl_3$, 25 ˚C)

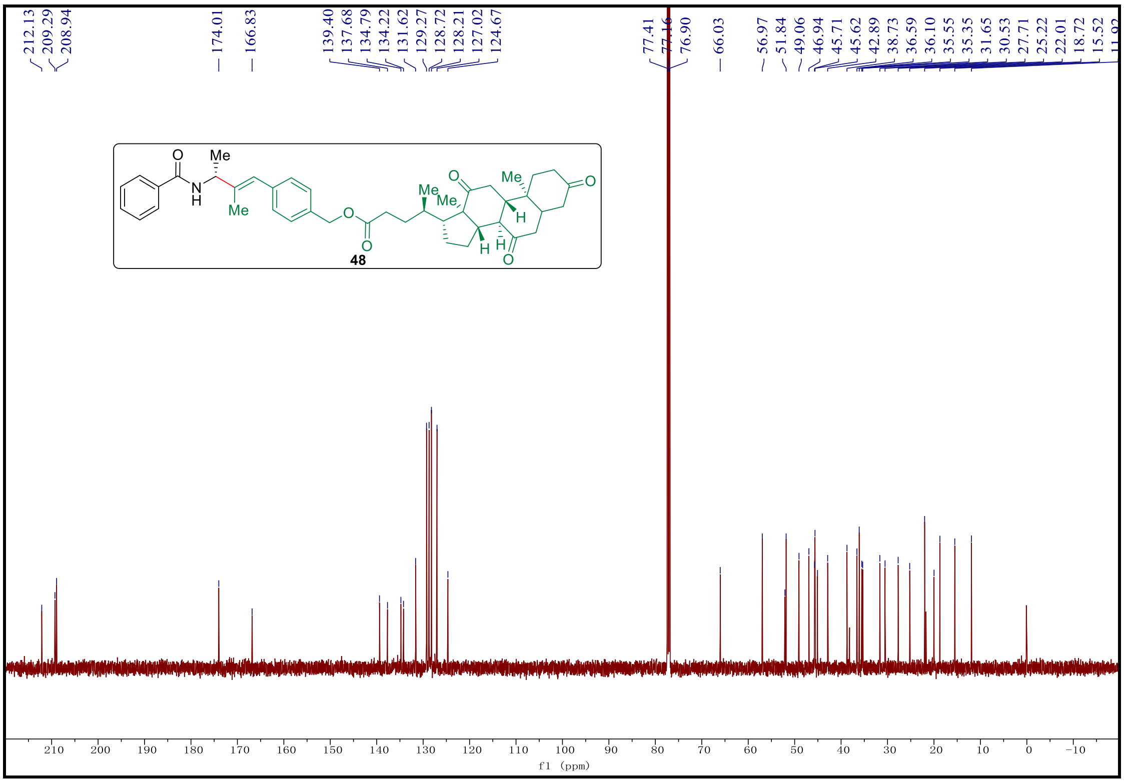

Supplementary Figure 102. ^{13}C NMR Spectrum of Compound **48** (126 MHz, $CDCl_3$, 25 ˚C)

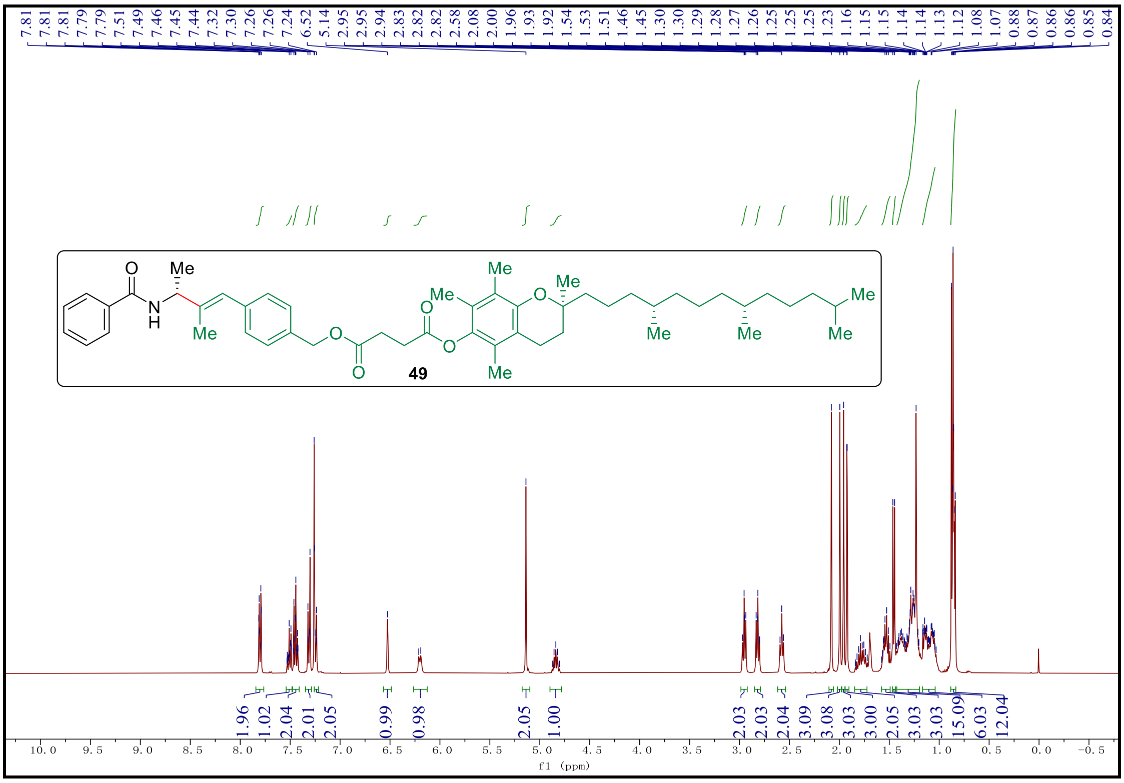

Supplementary Figure 103. ^{1}H NMR Spectrum of Compound **49** (400 MHz, $CDCl_3$, 25 ˚C)

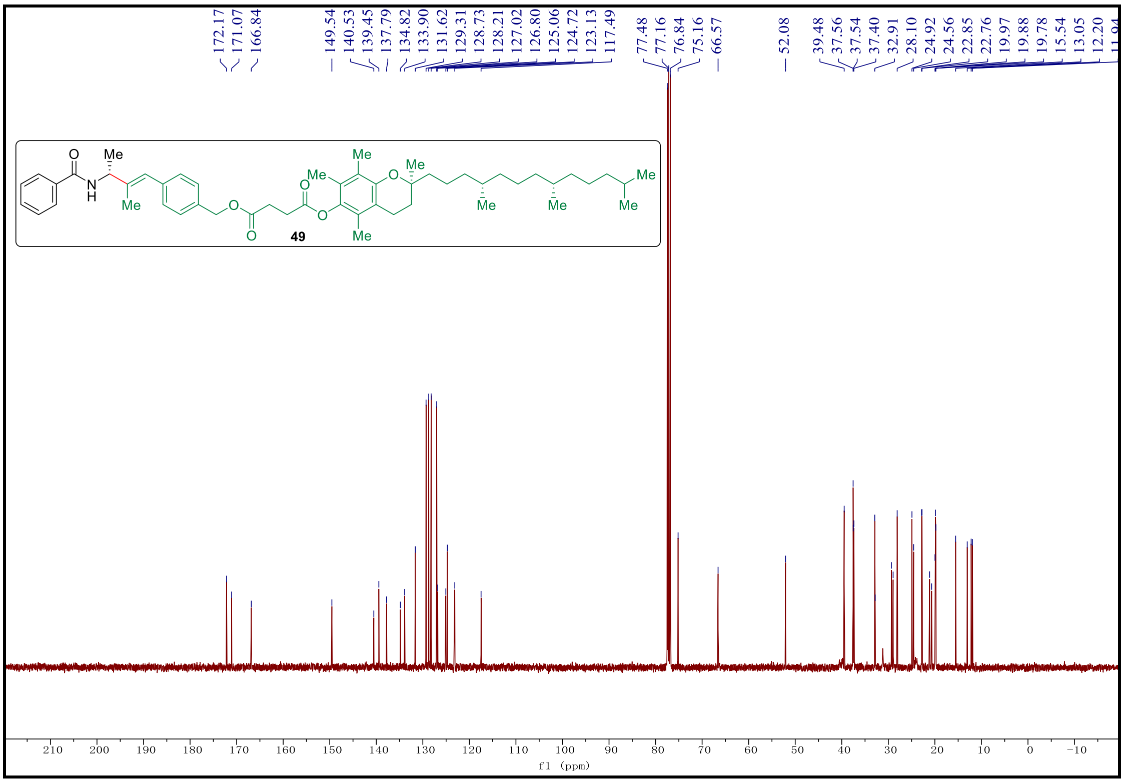

Supplementary Figure 104. ^{13}C NMR Spectrum of Compound **49** (101 MHz, $CDCl_3$, 25 ˚C)

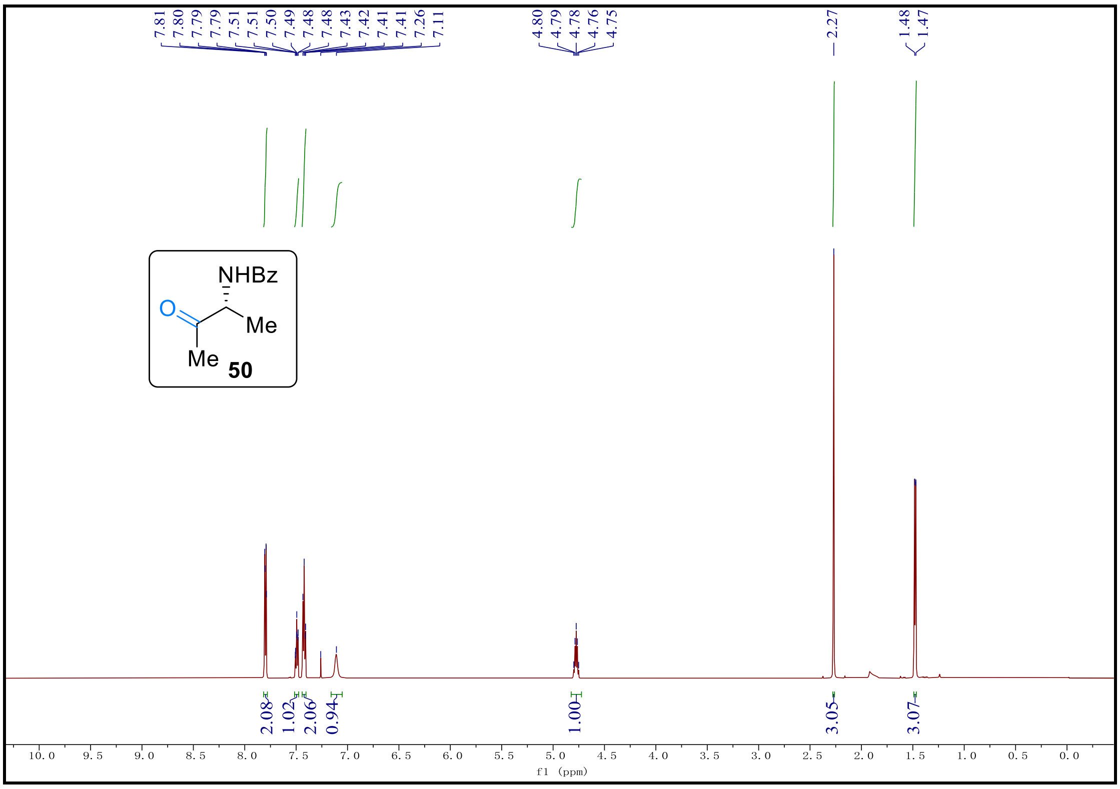

Supplementary Figure 105. ^{1}H NMR Spectrum of Compound **50** (600 MHz, $CDCl_3$, 25 ˚C)

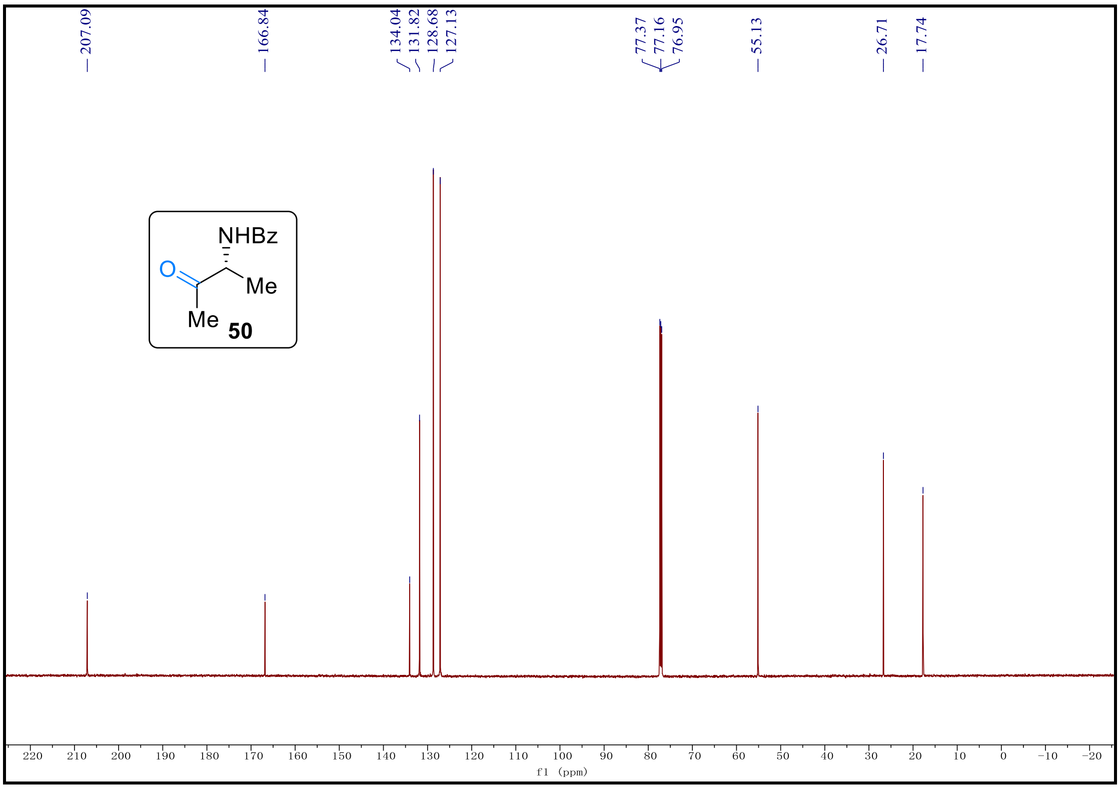

Supplementary Figure 106.^{13}C NMR Spectrum of Compound **50** (151 MHz, $CDCl_3$, 25 ˚C)

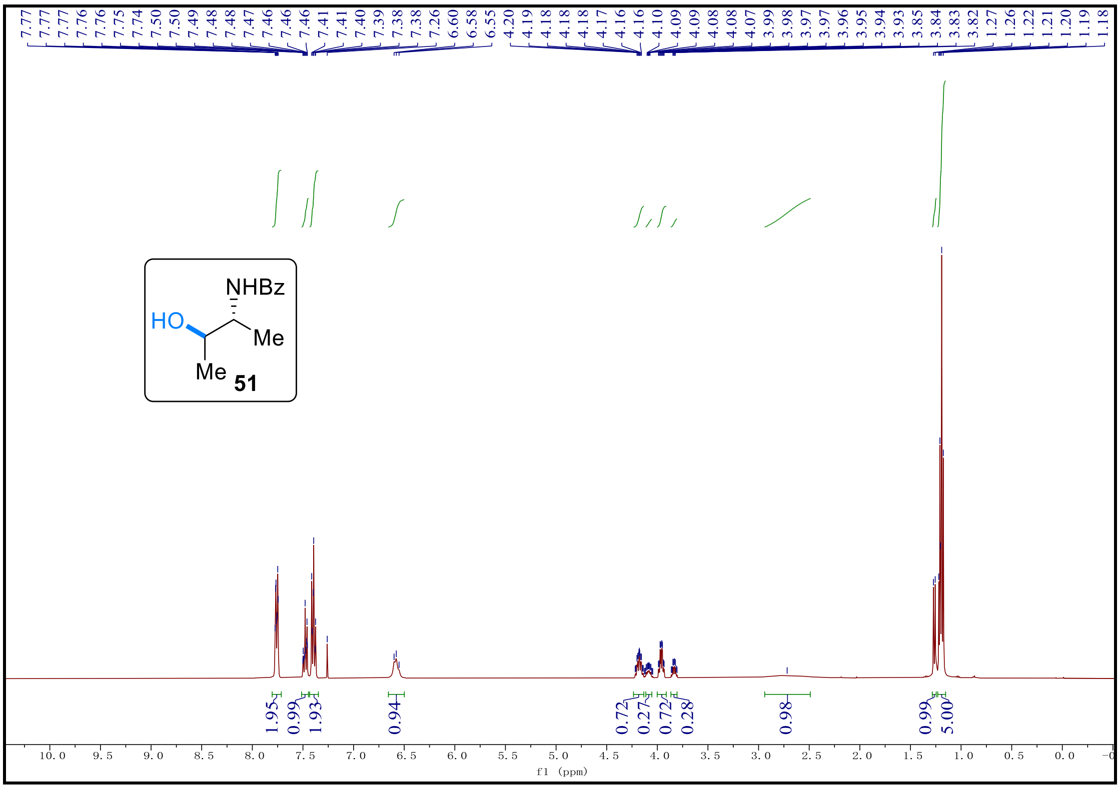

Supplementary Figure 107. ^{1}H NMR Spectrum of Compound **51** (400 MHz, $CDCl_3$, 25 ˚C)

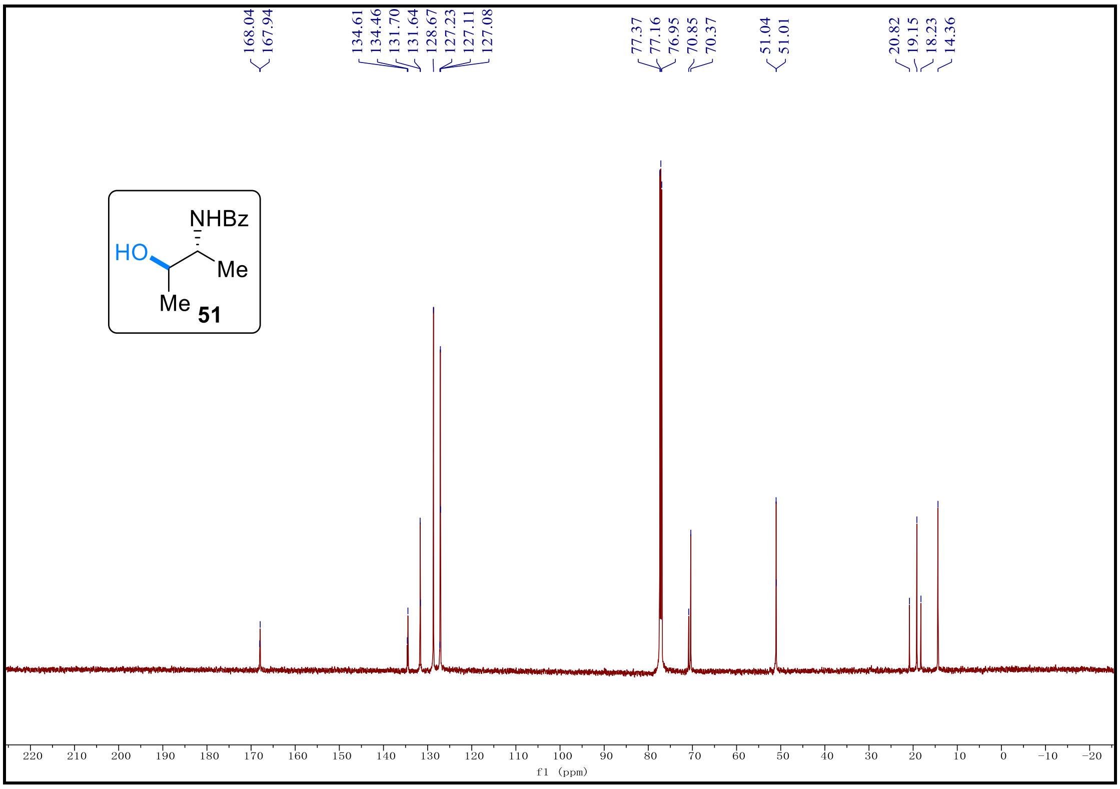

Supplementary Figure 108. ^{13}C NMR Spectrum of Compound **51** (151 MHz, $CDCl_3$, 25 ˚C)

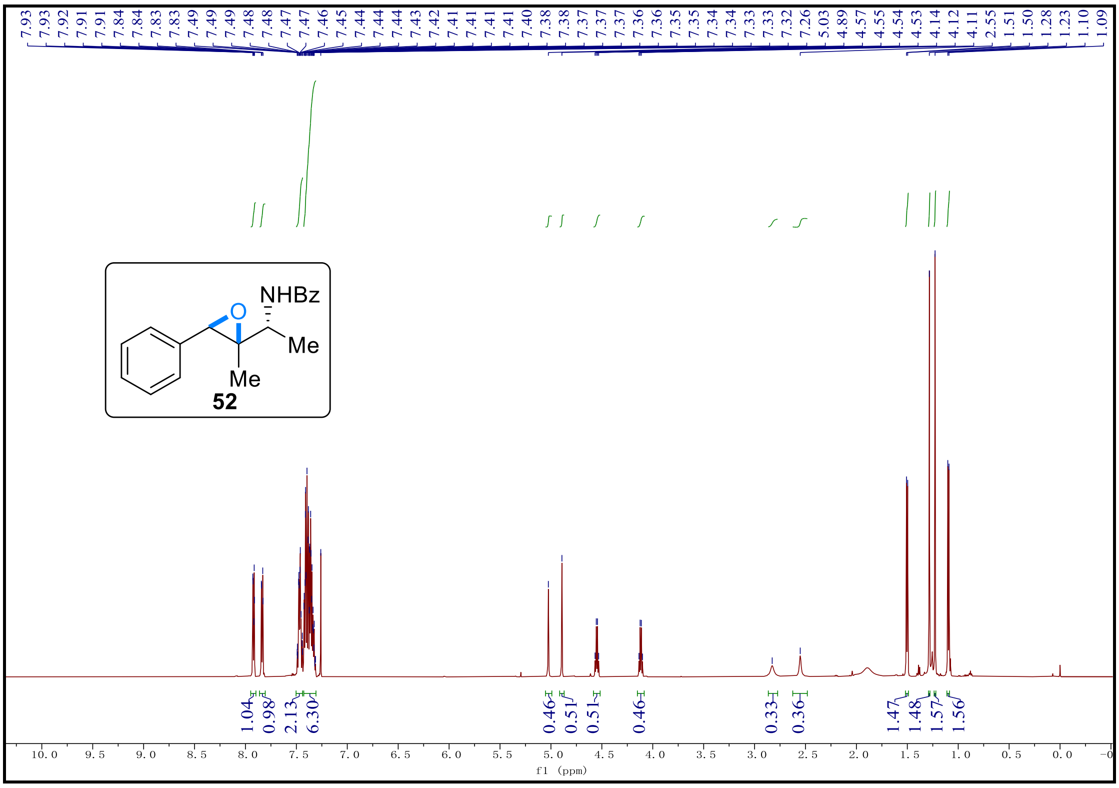

Supplementary Figure 109. ^{1}H NMR Spectrum of Compound **52** (600 MHz, $CDCl_3$, 25 ˚C)

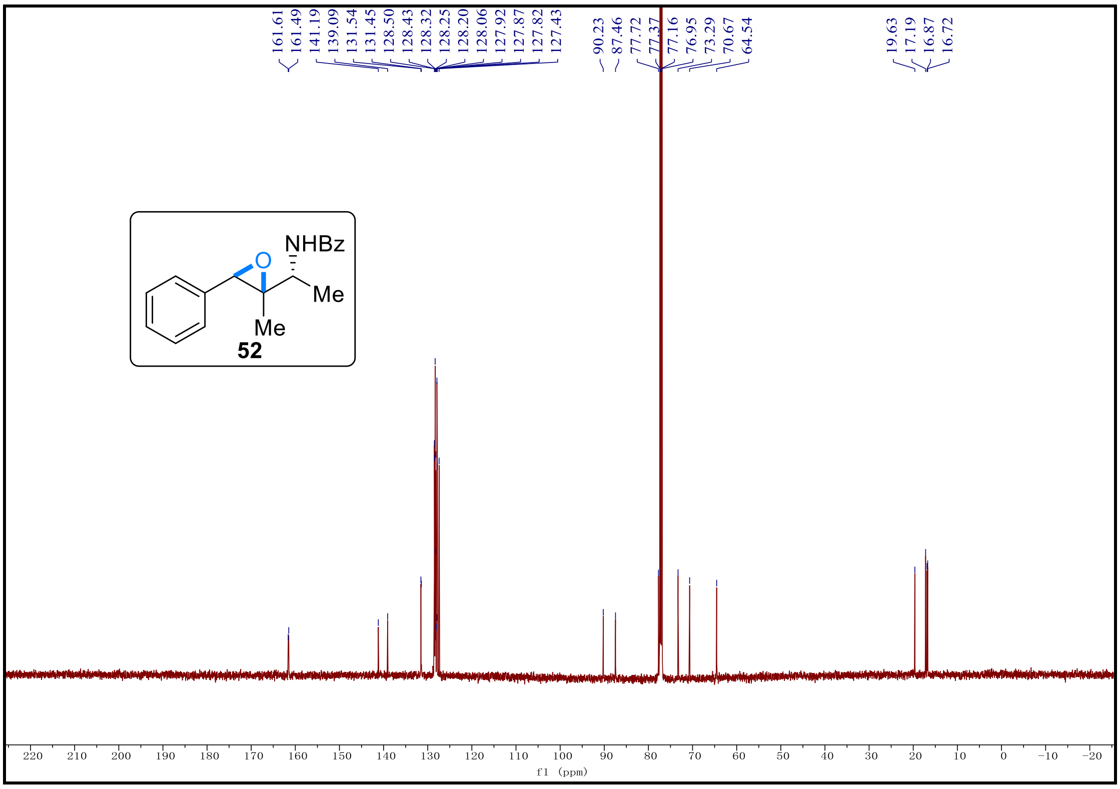

Supplementary Figure 110. ^{13}C NMR Spectrum of Compound **52** (151 MHz, $CDCl_3$, 25 °C)

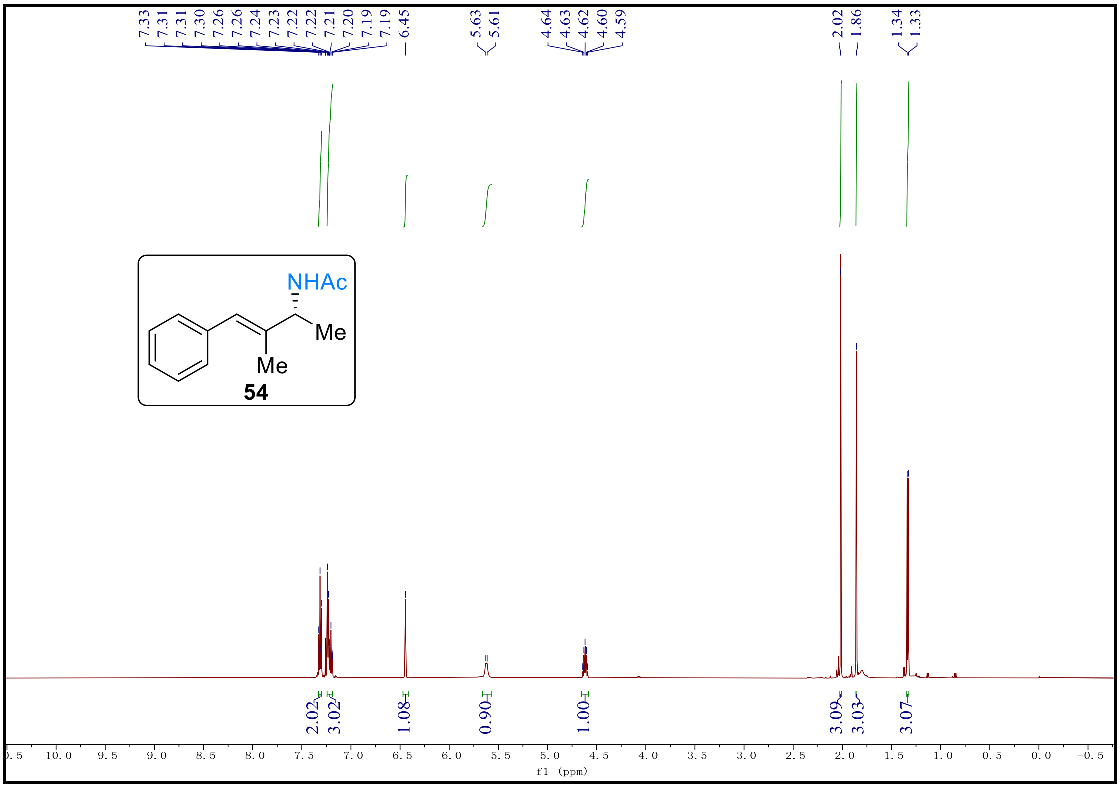

Supplementary Figure 111. ^{1}H NMR Spectrum of Compound **54** (600 MHz, $CDCl_3$, 25 °C)

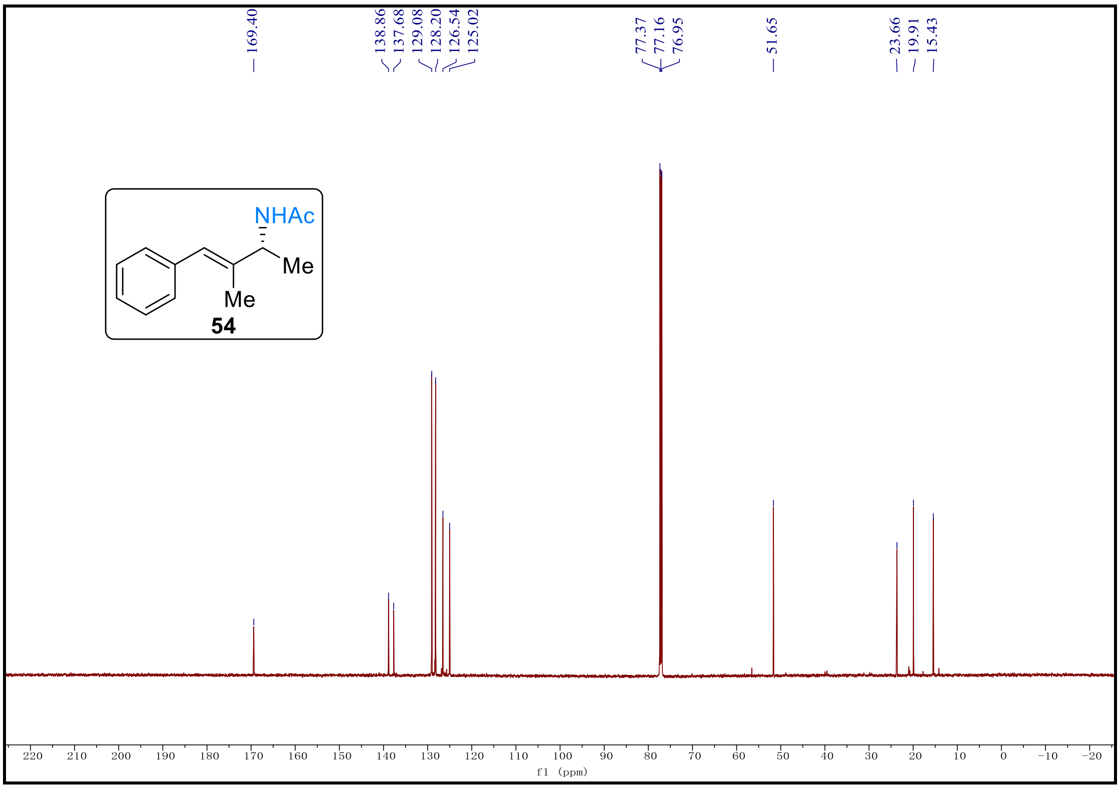

Supplementary Figure 112. ^{13}C NMR Spectrum of Compound **54** (151 MHz, $CDCl_3$, 25 ˚C)

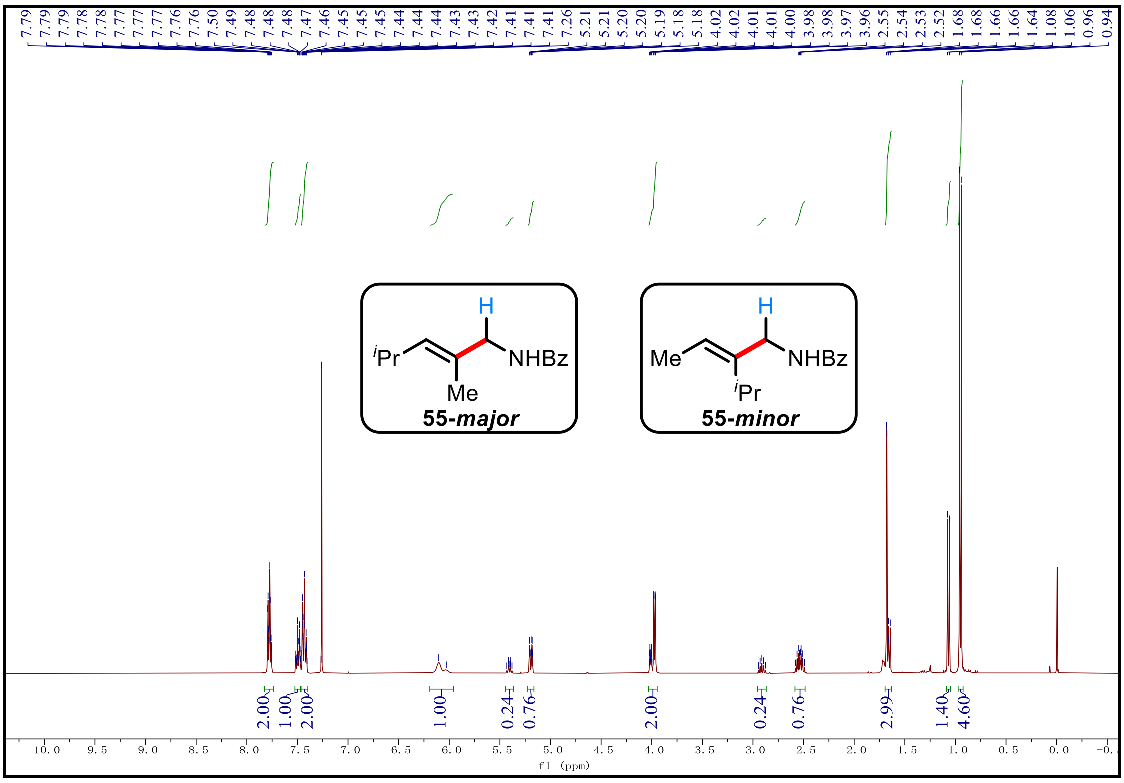

Supplementary Figure 113. ^{1}H NMR Spectrum of Compound **55** (400 MHz, $CDCl_3$, 25 ˚C)

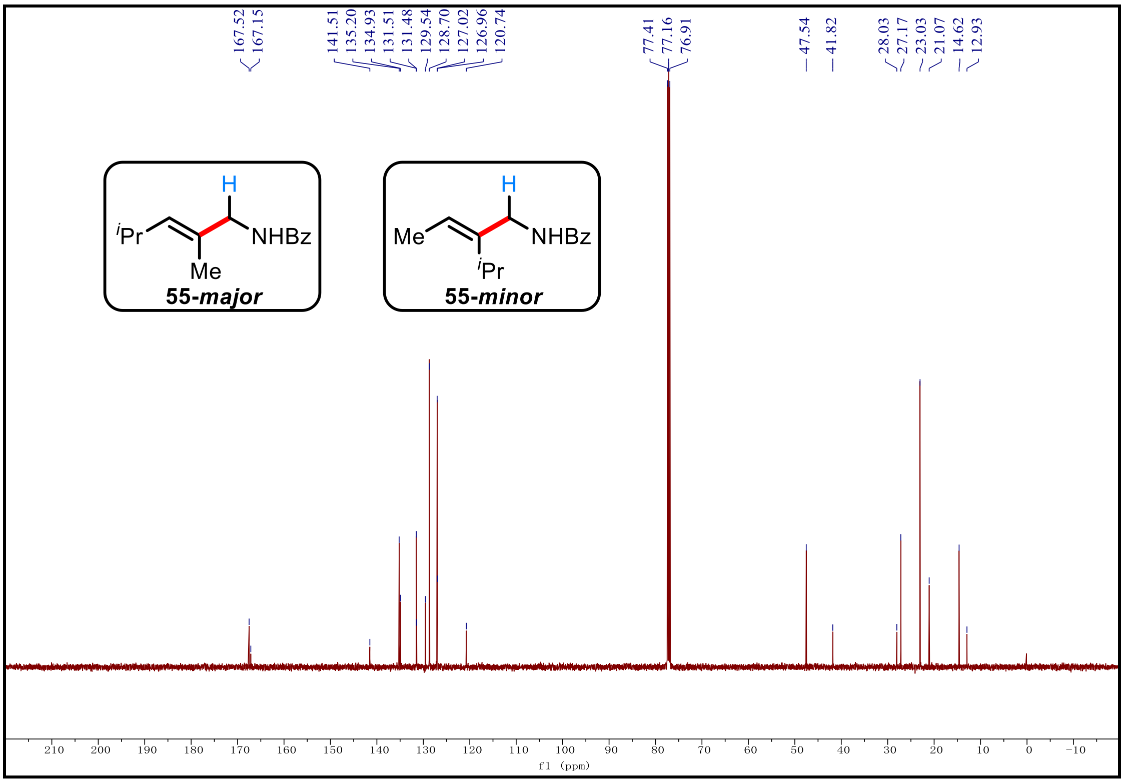

Supplementary Figure 114. ^{13}C NMR Spectrum of Compound **55** (126 MHz, $CDCl_3$, 25 ˚C)

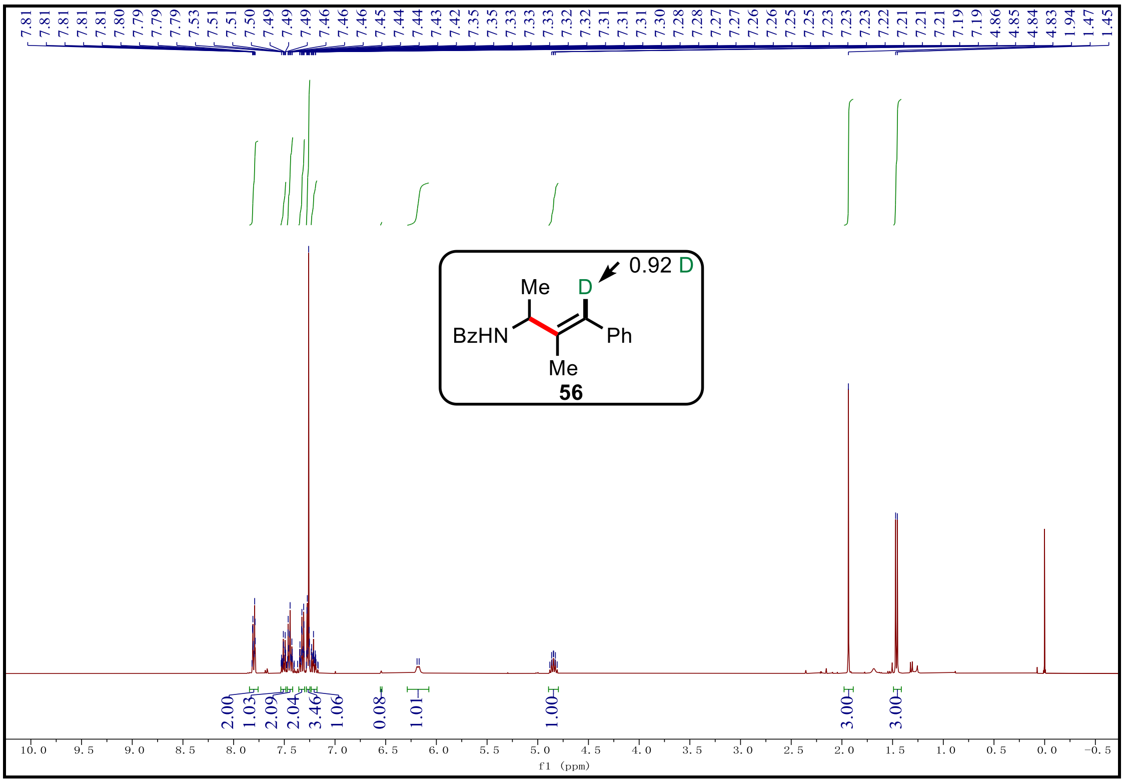

Supplementary Figure 115. ^{1}H NMR Spectrum of Compound **56** (400 MHz, $CDCl_3$, 25 ˚C)

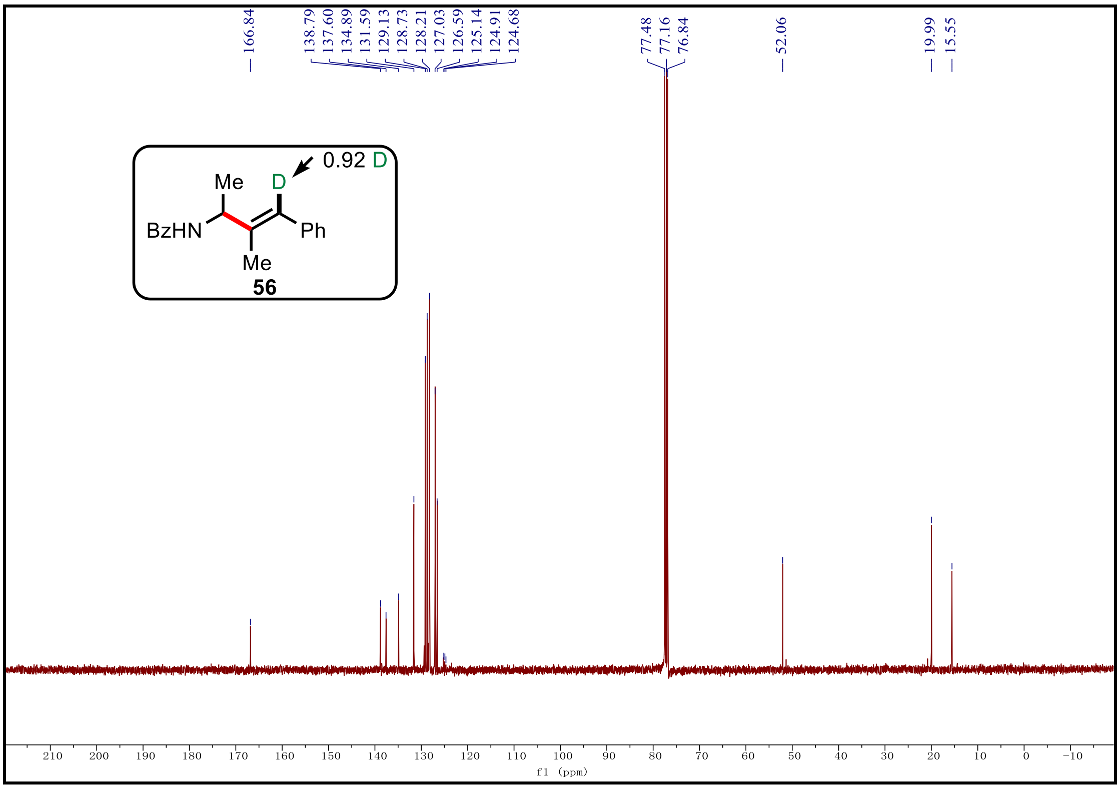

Supplementary Figure 116. ^{13}C NMR Spectrum of Compound **56** (101 MHz, $CDCl_3$, 25 ˚C)

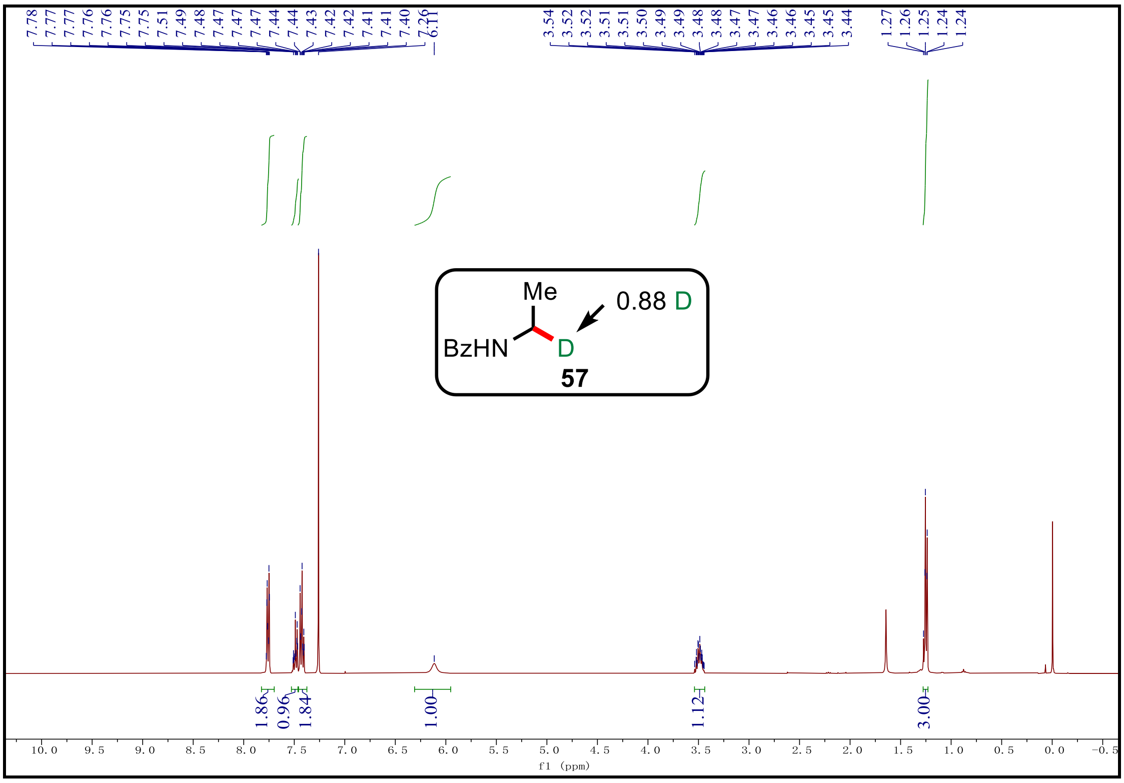

Supplementary Figure 117. ^{1}H NMR Spectrum of Compound **57** (400 MHz, $CDCl_3$, 25 ˚C)

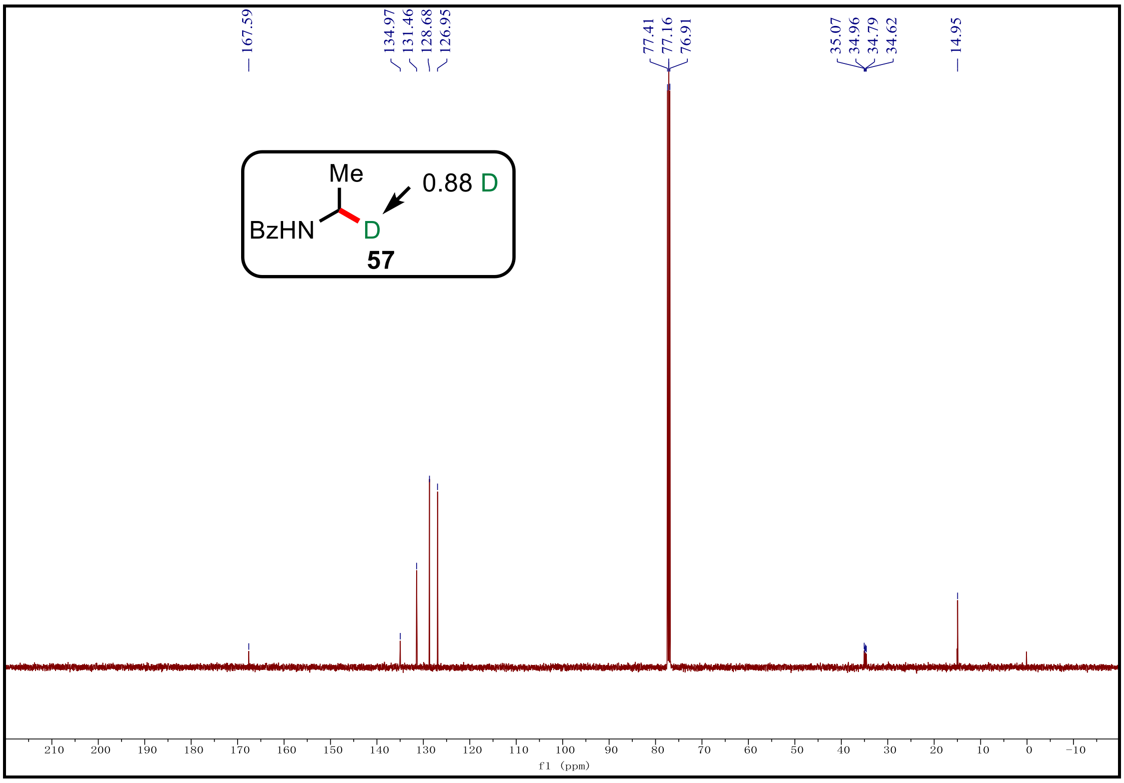

Supplementary Figure 118. ^{13}C NMR Spectrum of Compound **57** (126 MHz, $CDCl_3$, 25 ˚C)

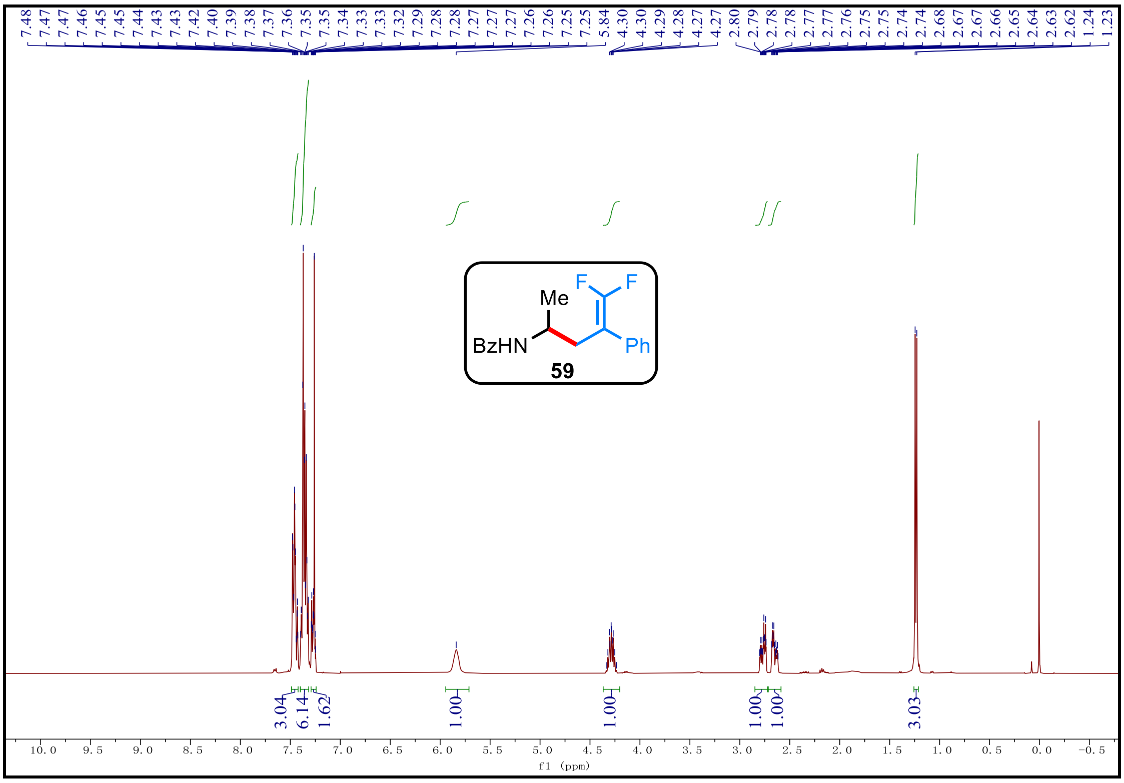

Supplementary Figure 119. ^{1}H NMR Spectrum of Compound **59** (400 MHz, $CDCl_3$, 25 ˚C)

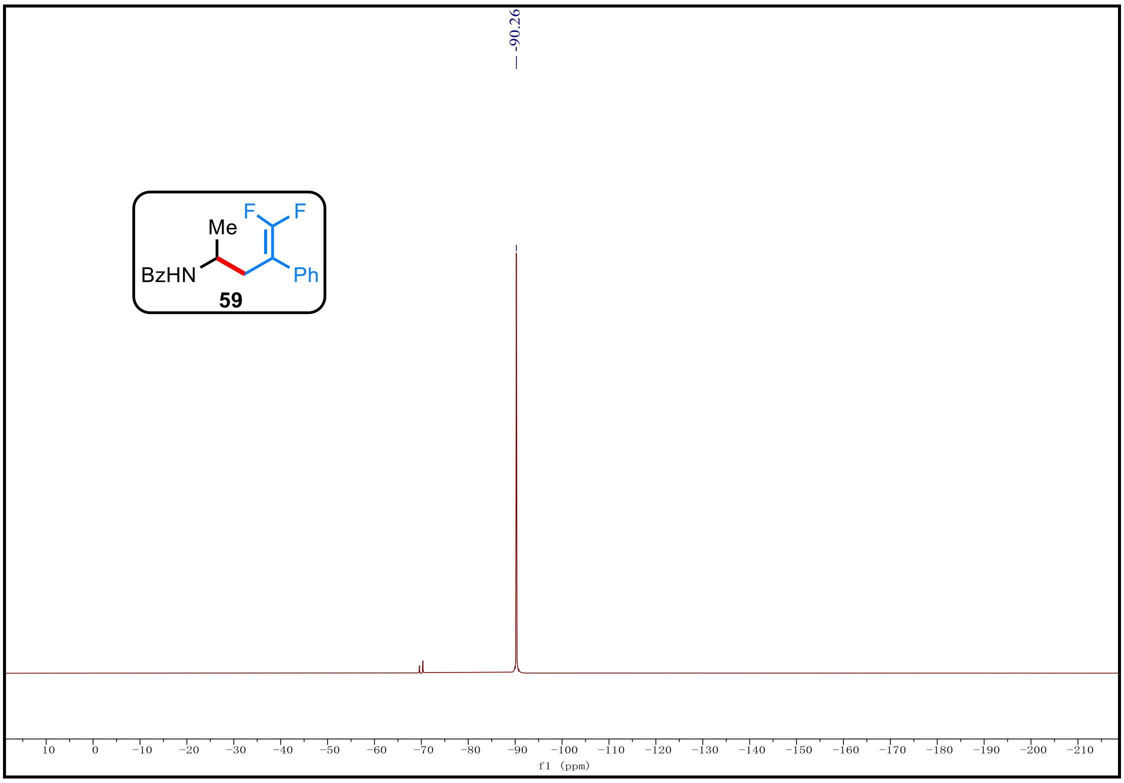

Supplementary Figure 120. ^{19}F NMR Spectrum of Compound **59** (376 MHz, $CDCl_3$, 25 ˚C)

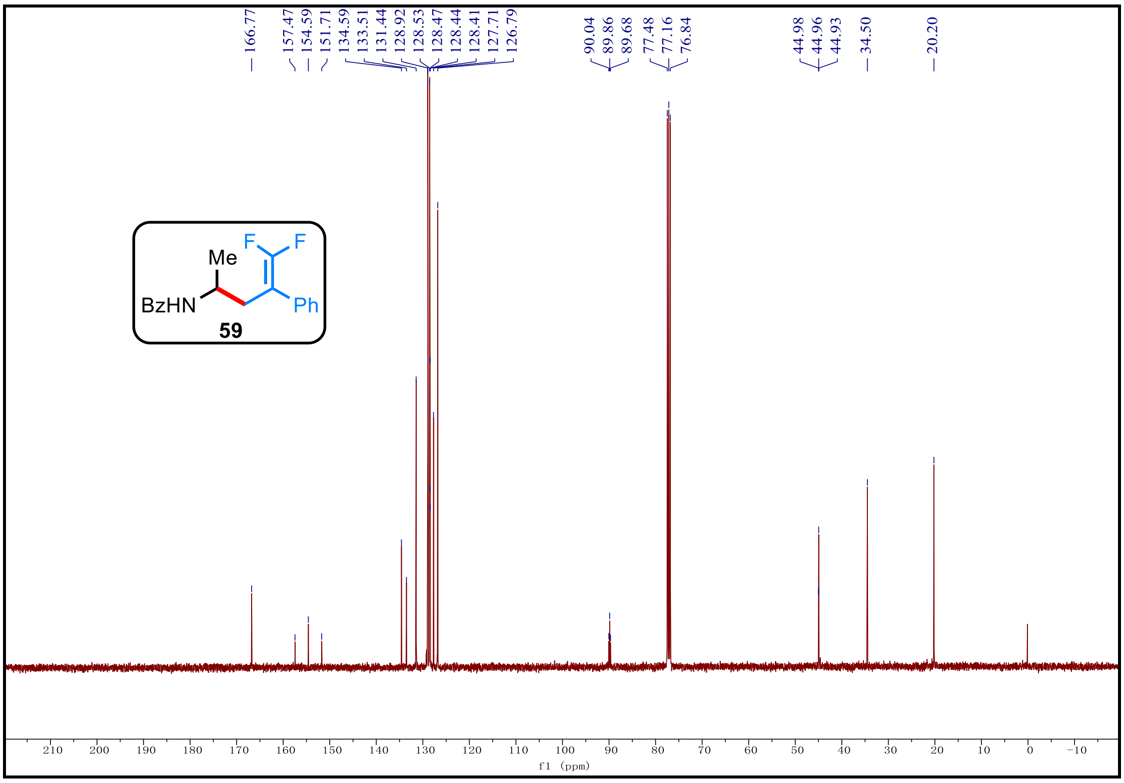

Supplementary Figure 121. ^{13}C NMR Spectrum of Compound **59** (101 MHz, $CDCl_3$, 25 ˚C)

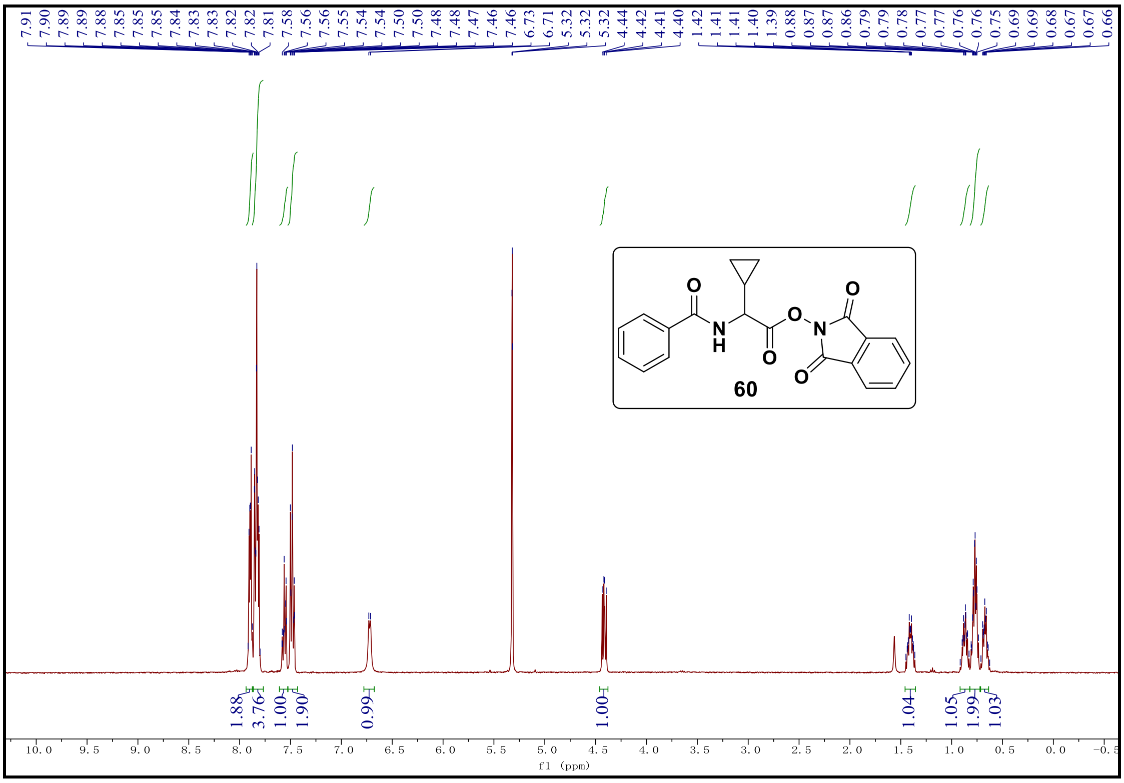

Supplementary Figure 122. ^{1}H NMR Spectrum of Compound **60** (400 MHz, CD_2Cl_2, 25 ˚C)

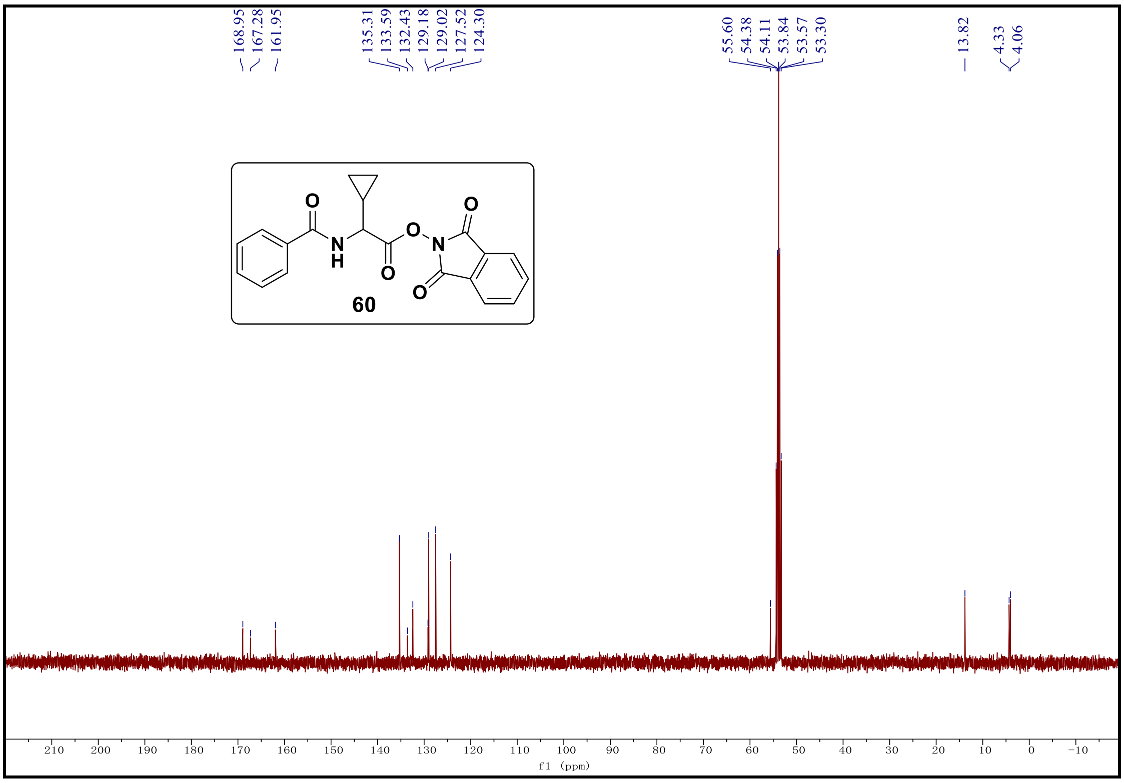

Supplementary Figure 123. ^{13}C NMR Spectrum of Compound **60** (101 MHz, CD_2Cl_2, 25 ˚C)

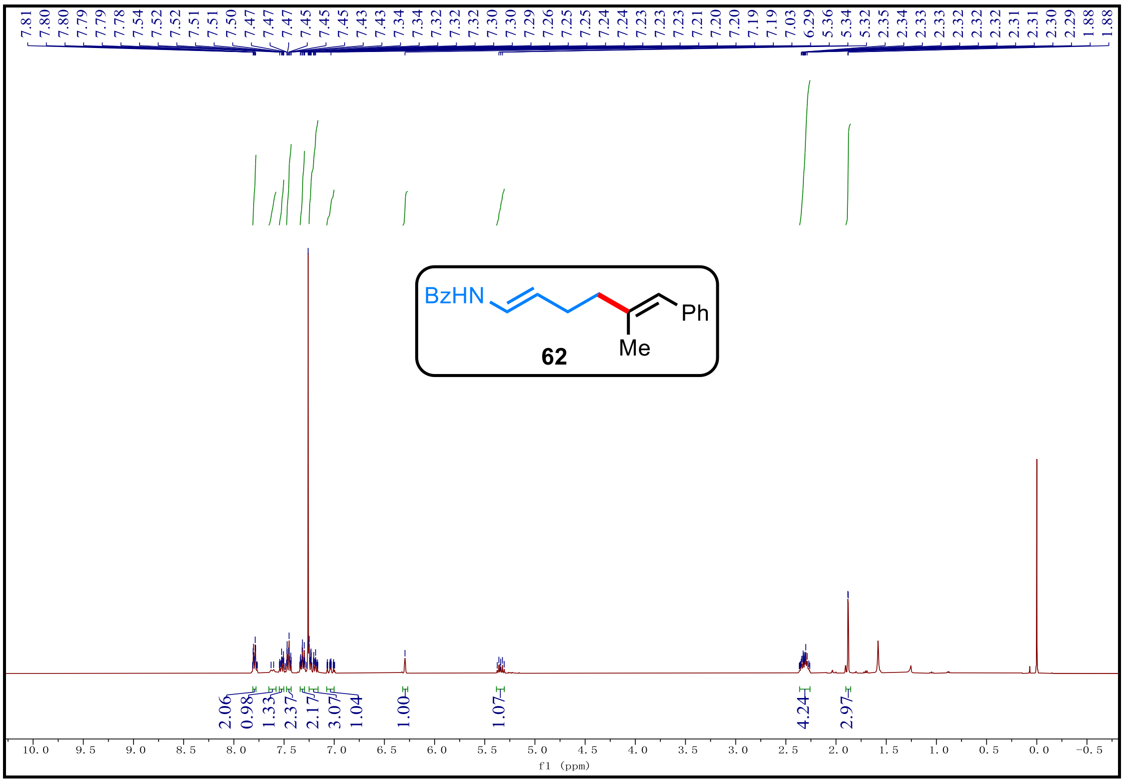

Supplementary Figure 124. ^{1}H NMR Spectrum of Compound **62** (400 MHz, $CDCl_3$, 25 ˚C)

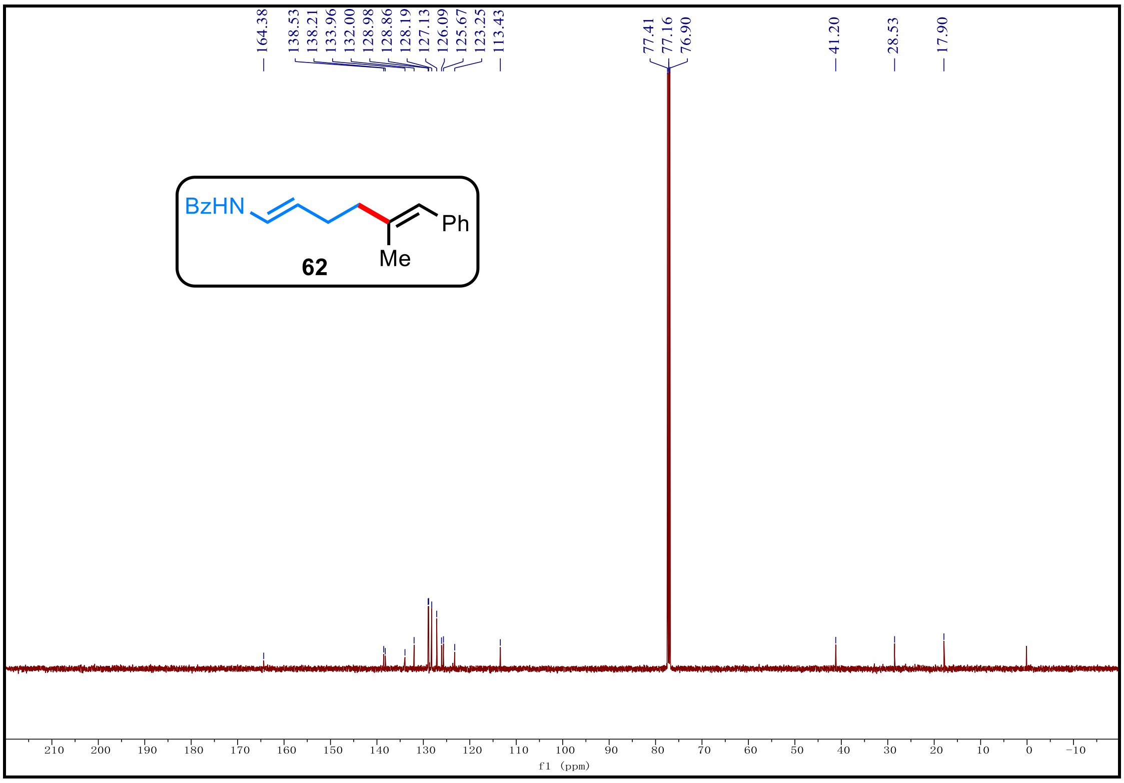

Supplementary Figure 125. ^{13}C NMR Spectrum of Compound **62** (126 MHz, $CDCl_3$, 25 ˚C)

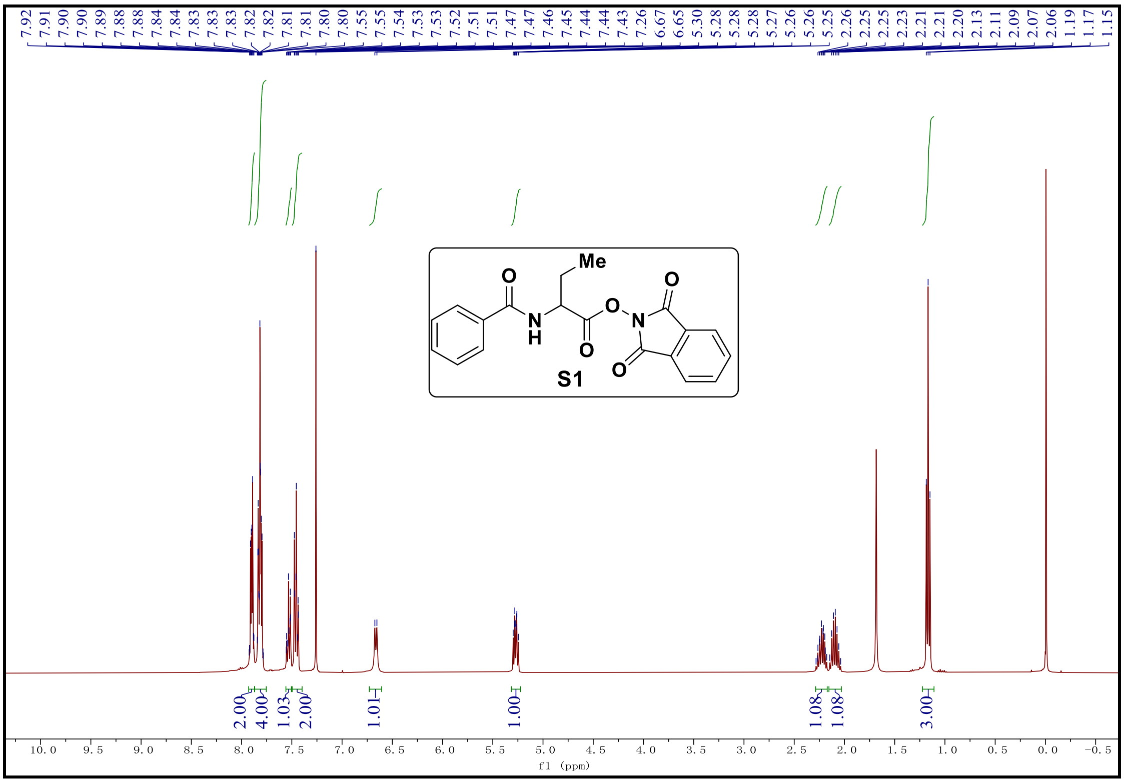

Supplementary Figure 126. ^{1}H NMR Spectrum of Compound **S1** (400 MHz, $CDCl_3$, 25 ˚C)

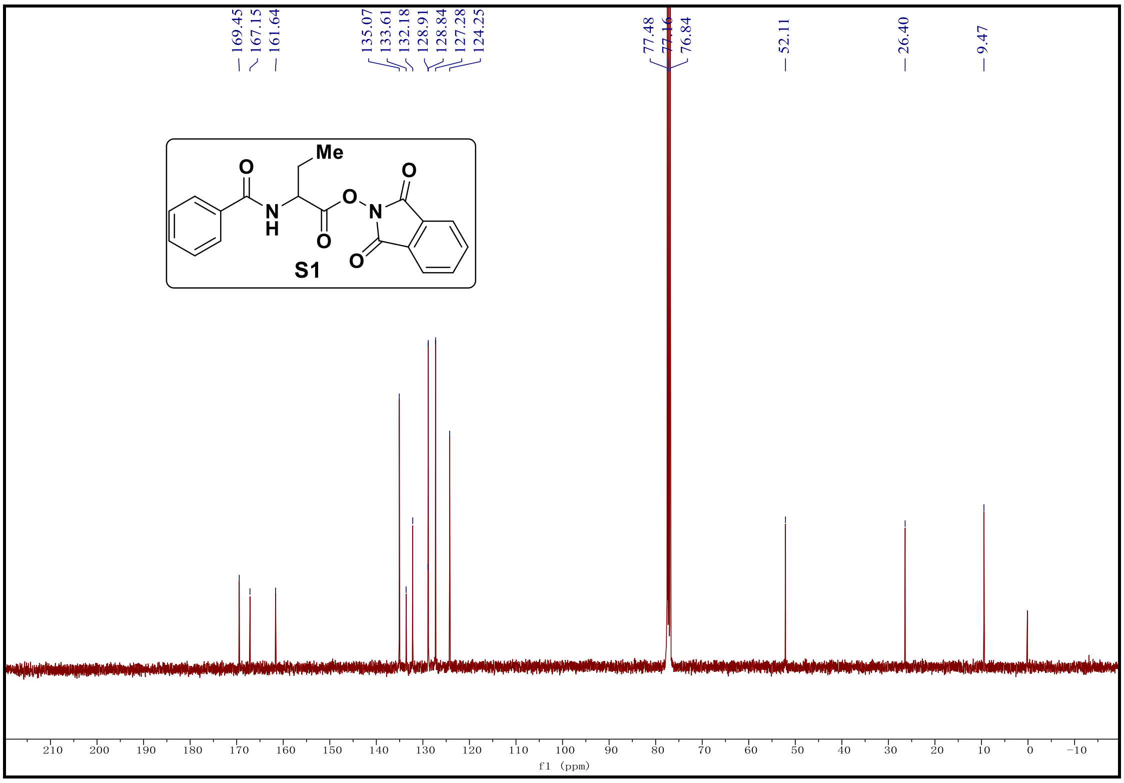

Supplementary Figure 127. ^{13}C NMR Spectrum of Compound **S1** (101 MHz, $CDCl_3$, 25 ˚C)

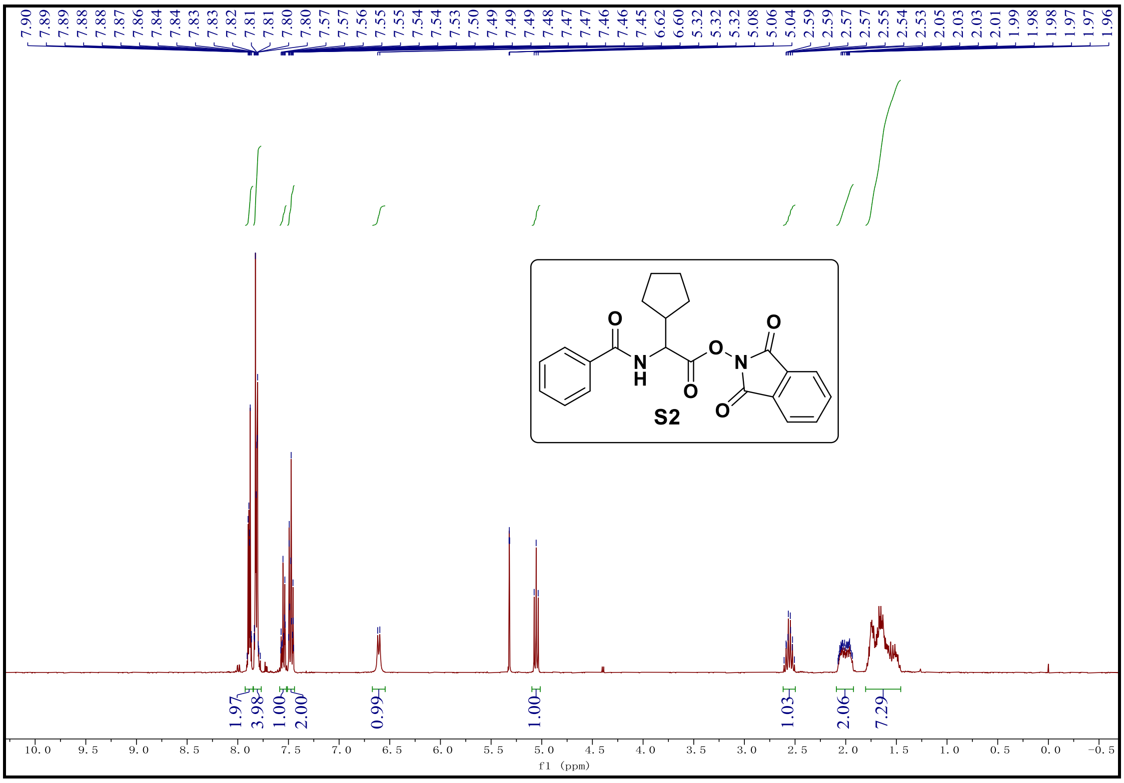

Supplementary Figure 128. ^{1}H NMR Spectrum of Compound **S2** (400 MHz, CD_2Cl_2, 25 ˚C)

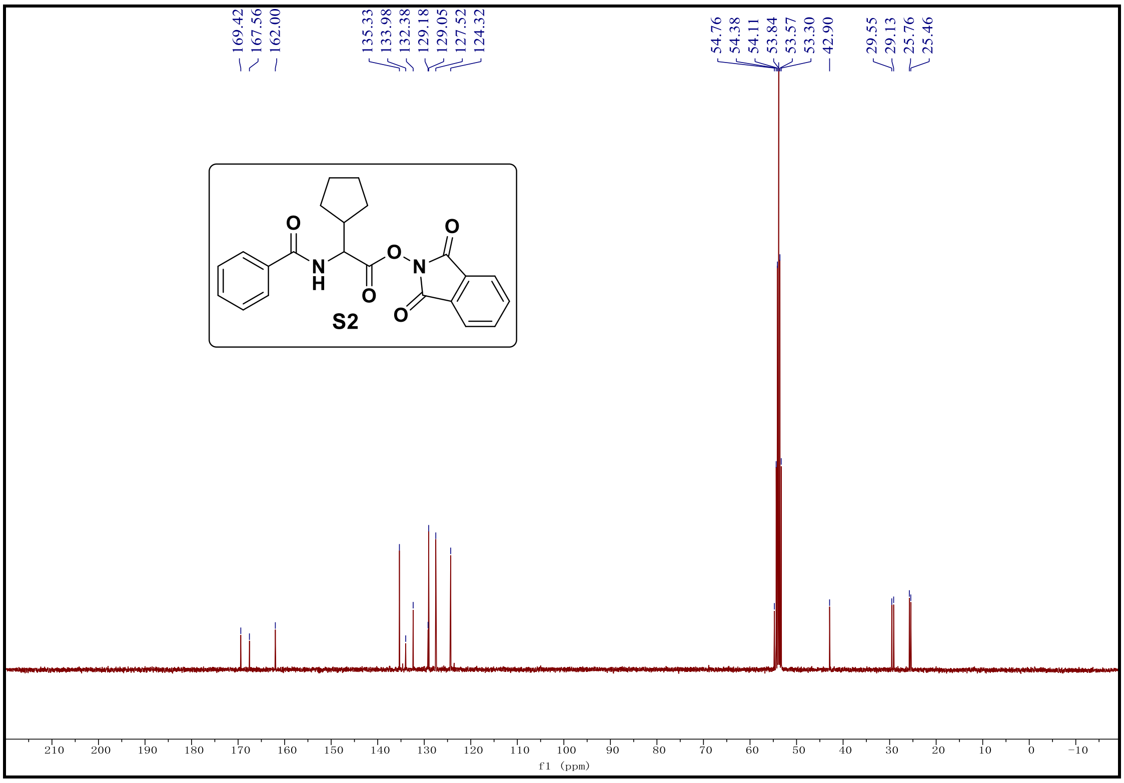

Supplementary Figure 129. ^{13}C NMR Spectrum of Compound **S2** (101 MHz, CD_2Cl_2, 25 ˚C)

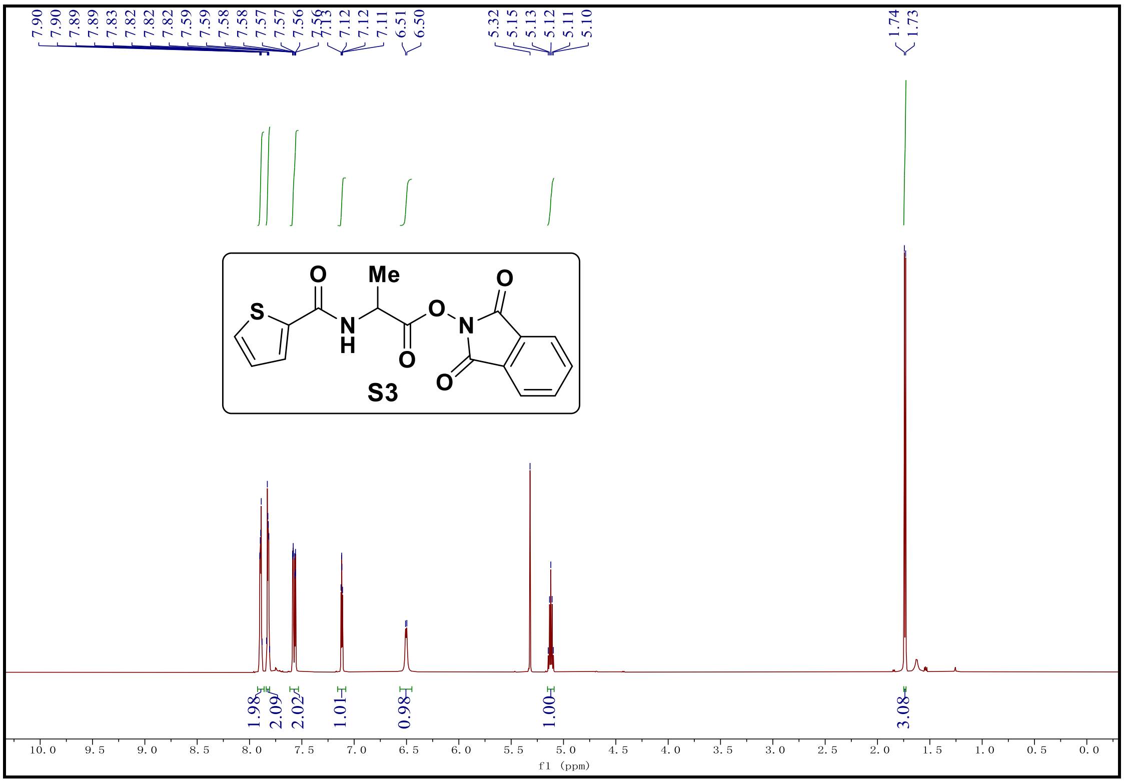

Supplementary Figure 130. ^{1}H NMR Spectrum of Compound **S3** (600 MHz, CD_2Cl_2, 25 ˚C)

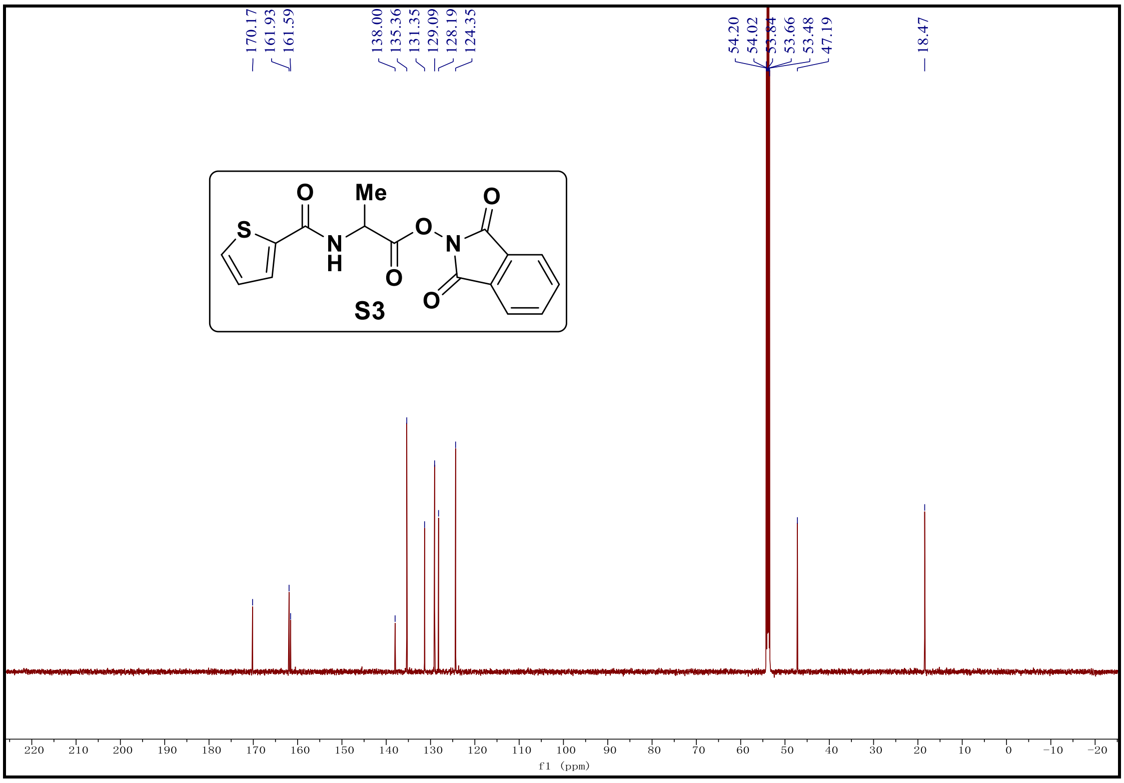

Supplementary Figure 131. ^{13}C NMR Spectrum of Compound **S3** (151 MHz, CD_2Cl_2, 25 ˚C)

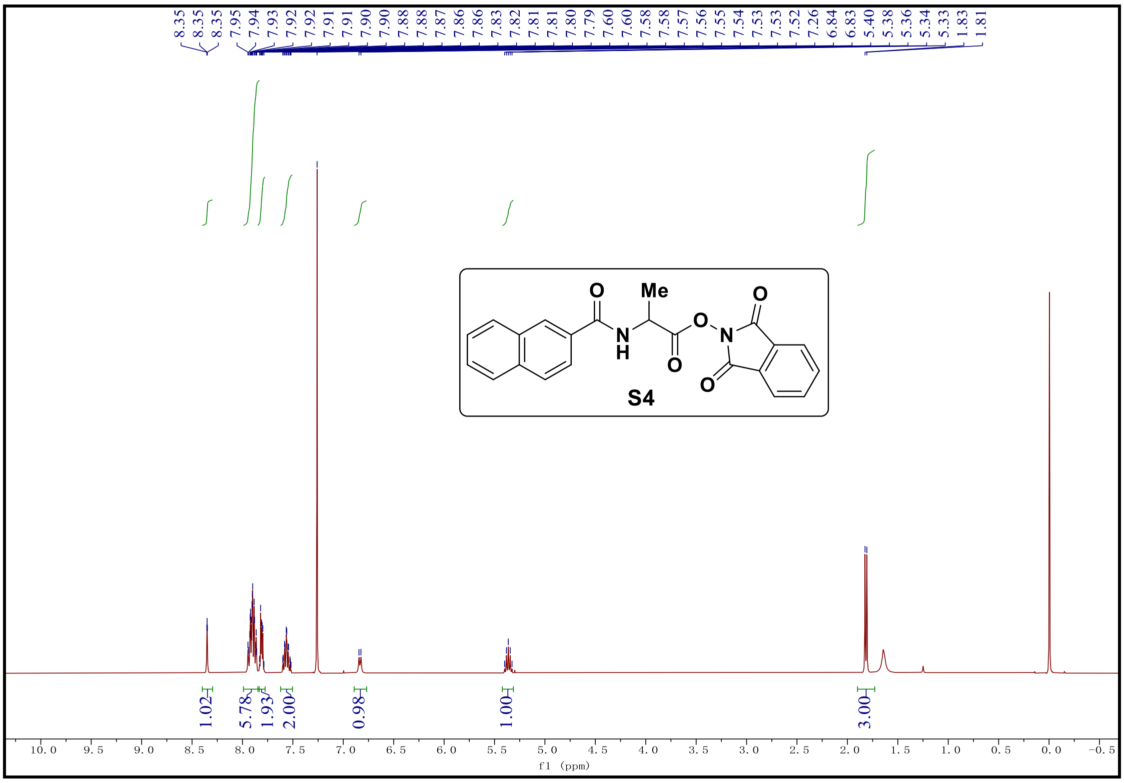

Supplementary Figure 132. ^{1}H NMR Spectrum of Compound **S4** (400 MHz, $CDCl_3$, 25 ˚C)

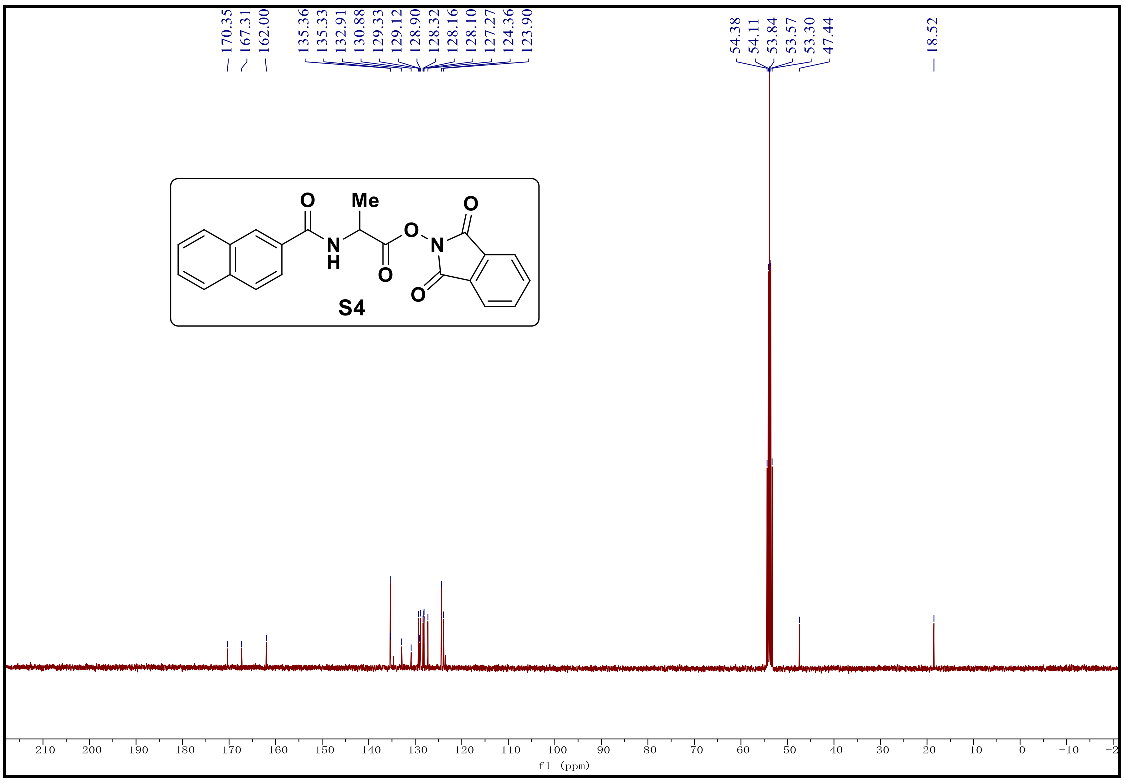

Supplementary Figure 133. ^{13}C NMR Spectrum of Compound **S4** (101 MHz, CD_2Cl_2, 25 ˚C)

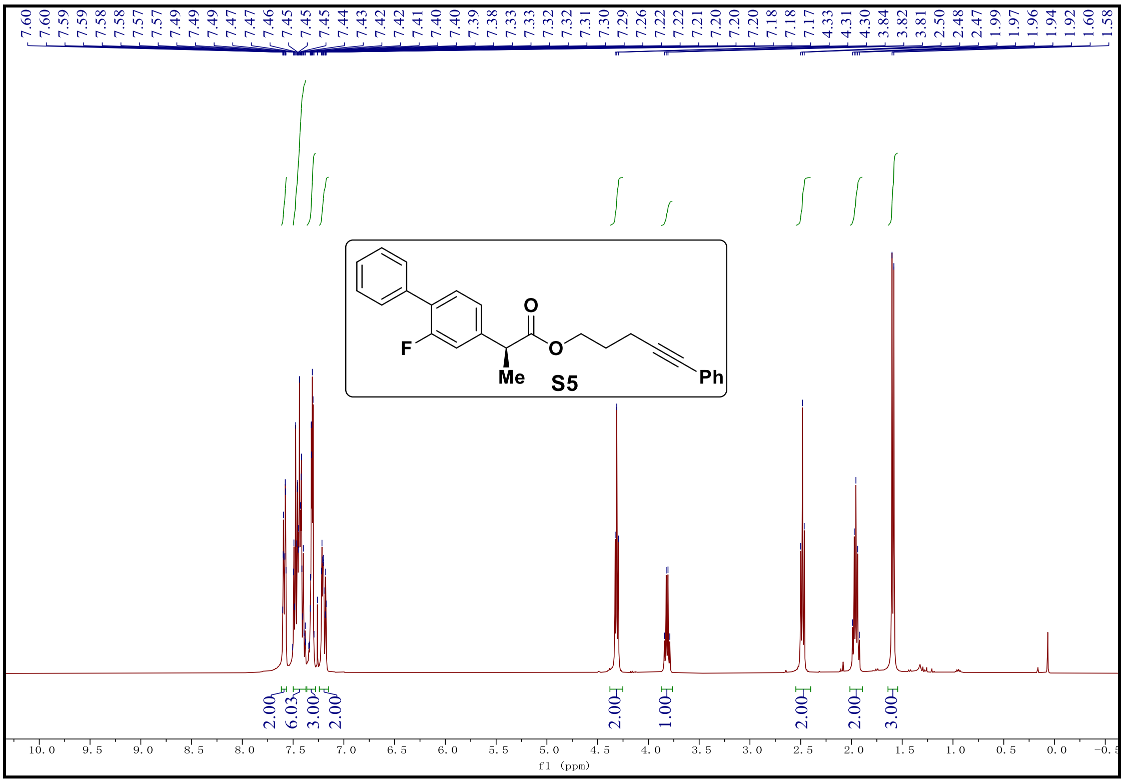

Supplementary Figure 134. ^{1}H NMR Spectrum of Compound **S5** (400 MHz, $CDCl_3$, 25 °C)

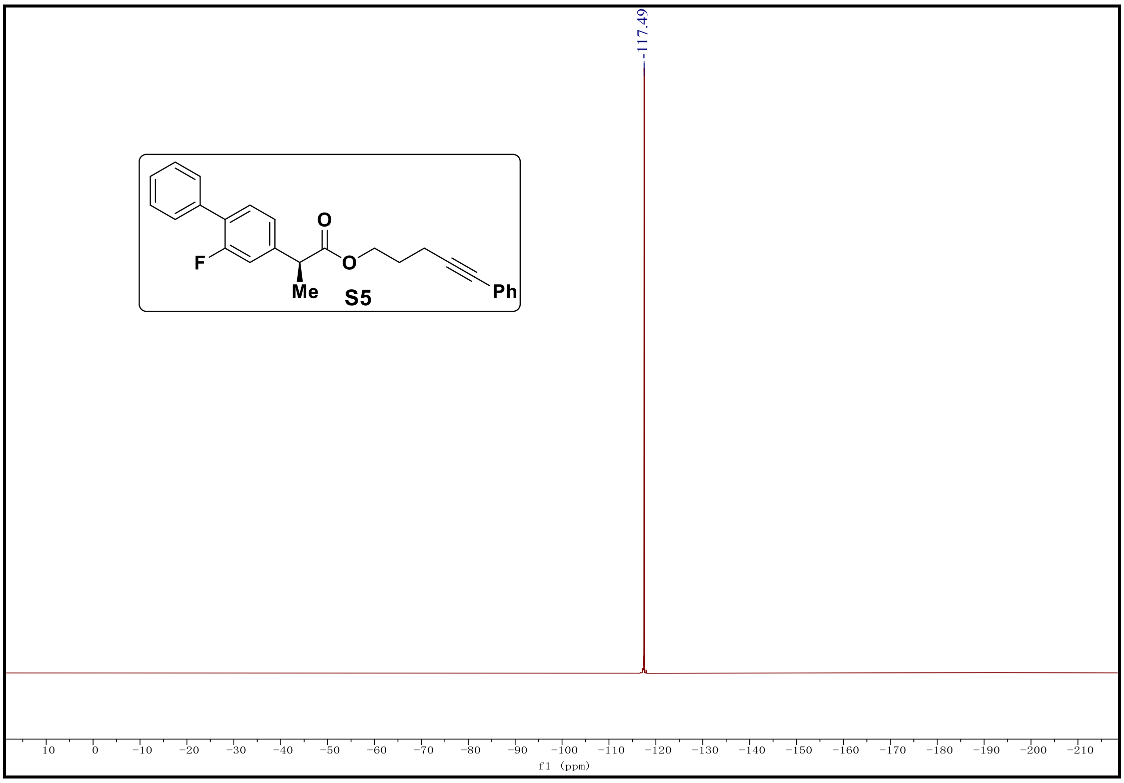

Supplementary Figure 135. ^{19}F NMR Spectrum of Compound **S5** (376 MHz, $CDCl_3$, 25 °C)

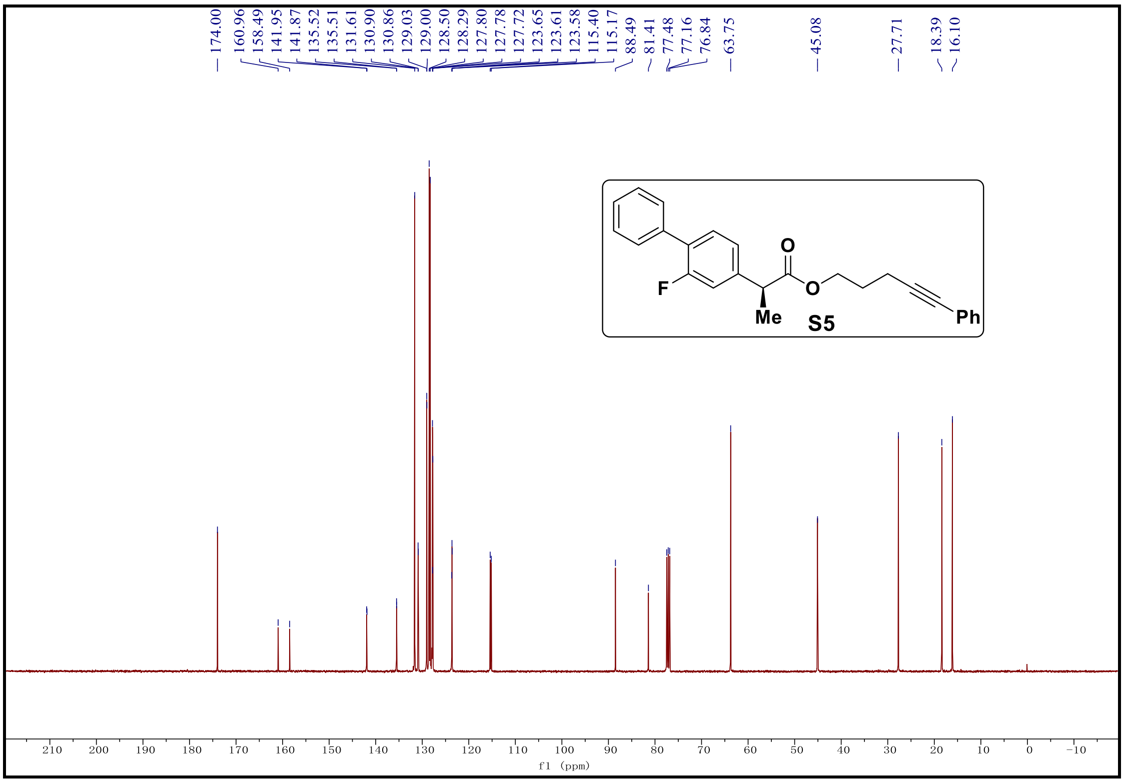

Supplementary Figure 136. ^{13}C NMR Spectrum of Compound **S5** (101 MHz, $CDCl_3$, 25 °C)

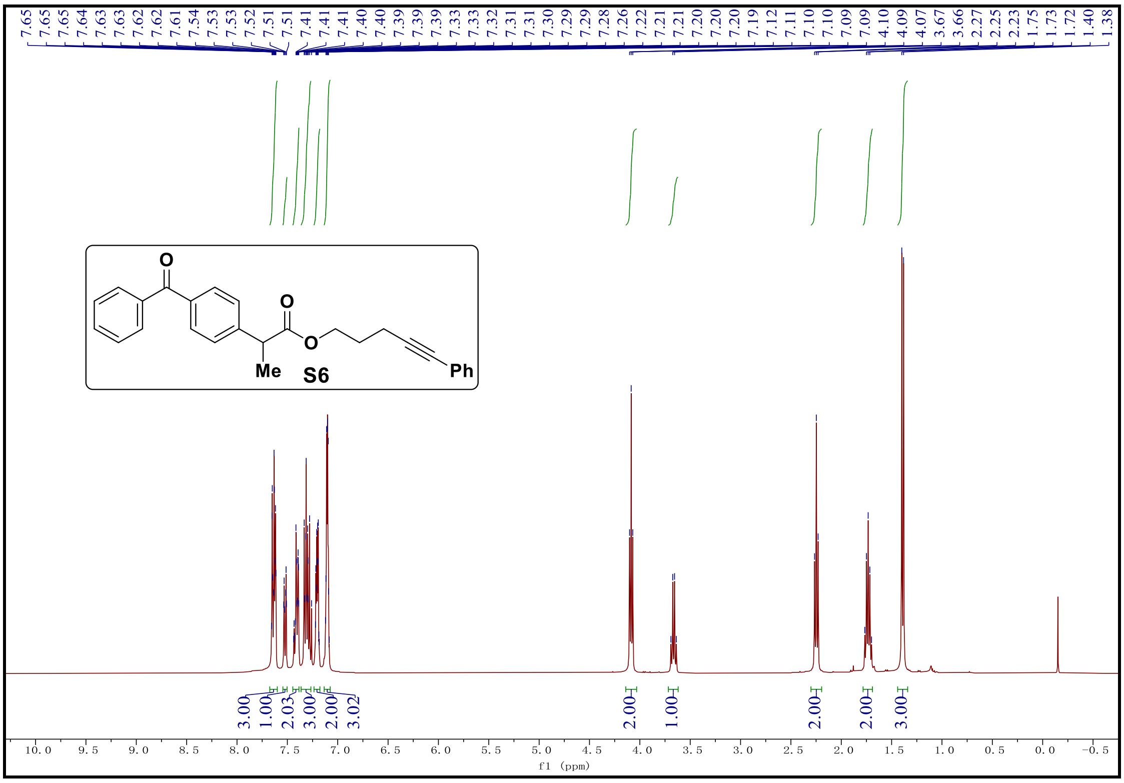

Supplementary Figure 137. ^{1}H NMR Spectrum of Compound **S6** (400 MHz, $CDCl_3$, 25 °C)

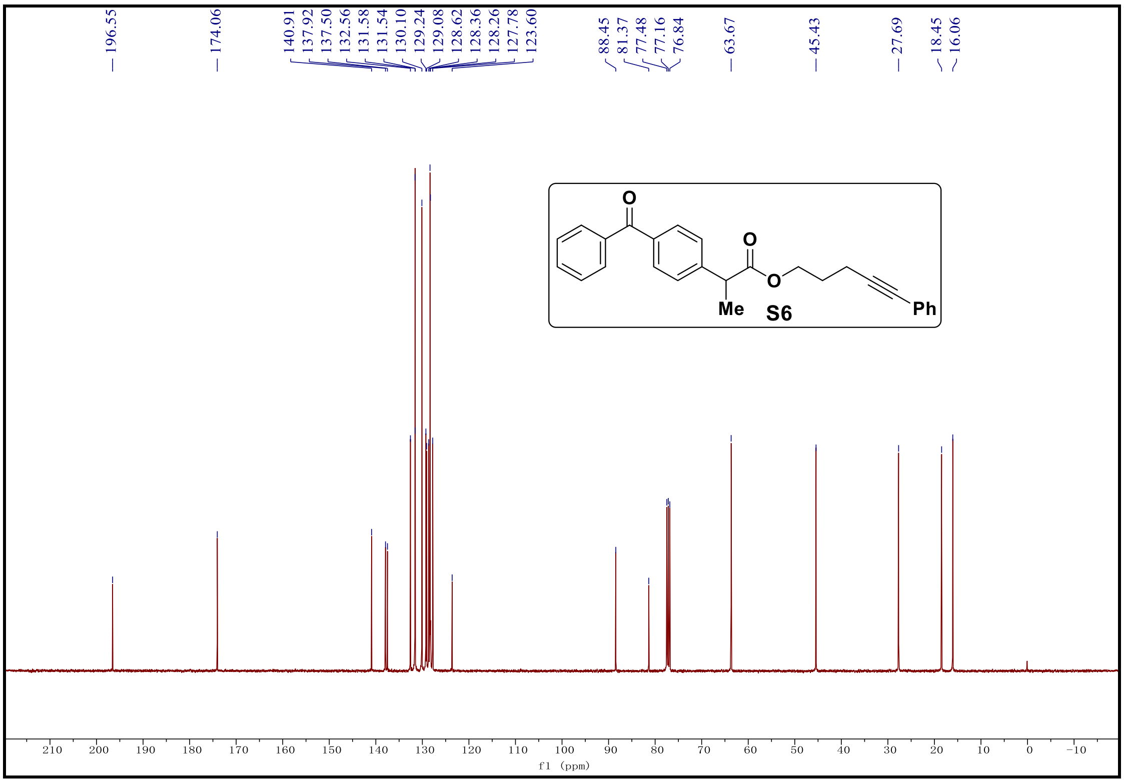

Supplementary Figure 138. ^{13}C NMR Spectrum of Compound **S6** (101 MHz, $CDCl_3$, 25 °C)

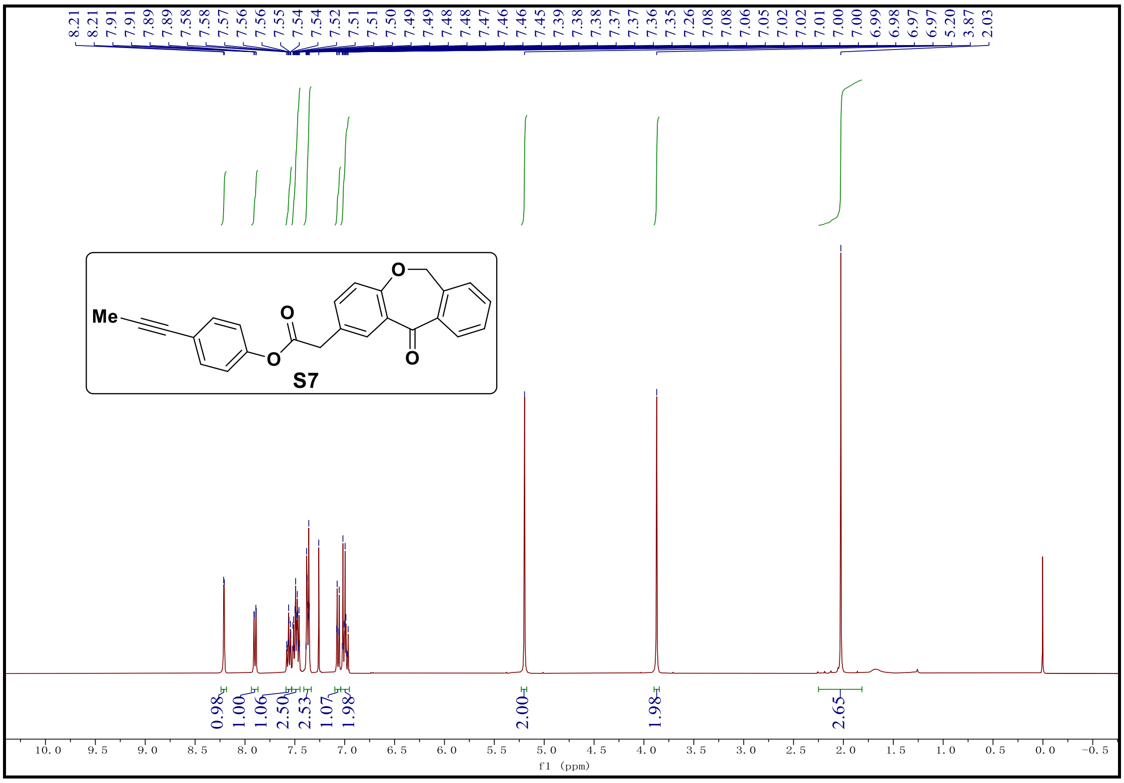

Supplementary Figure 139. ^{1}H NMR Spectrum of Compound **S7** (400 MHz, $CDCl_3$, 25 °C)

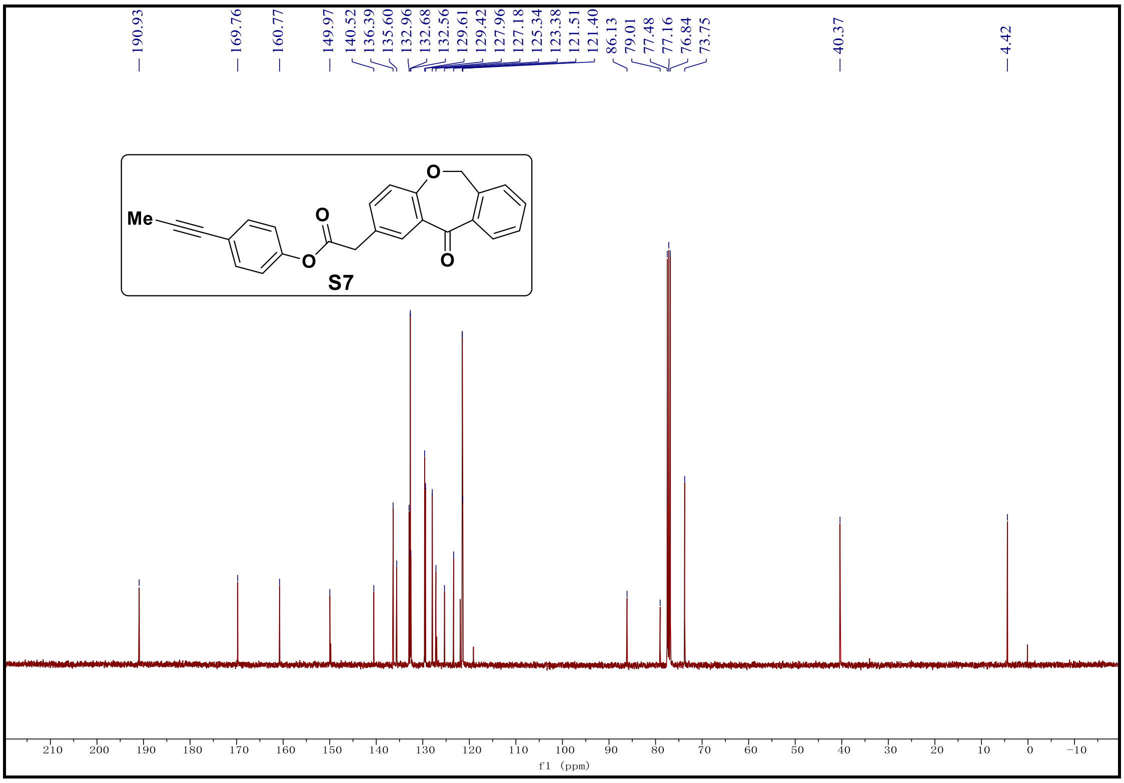

Supplementary Figure 140. ^{13}C NMR Spectrum of Compound **S7** (101 MHz, $CDCl_3$, 25 ˚C)

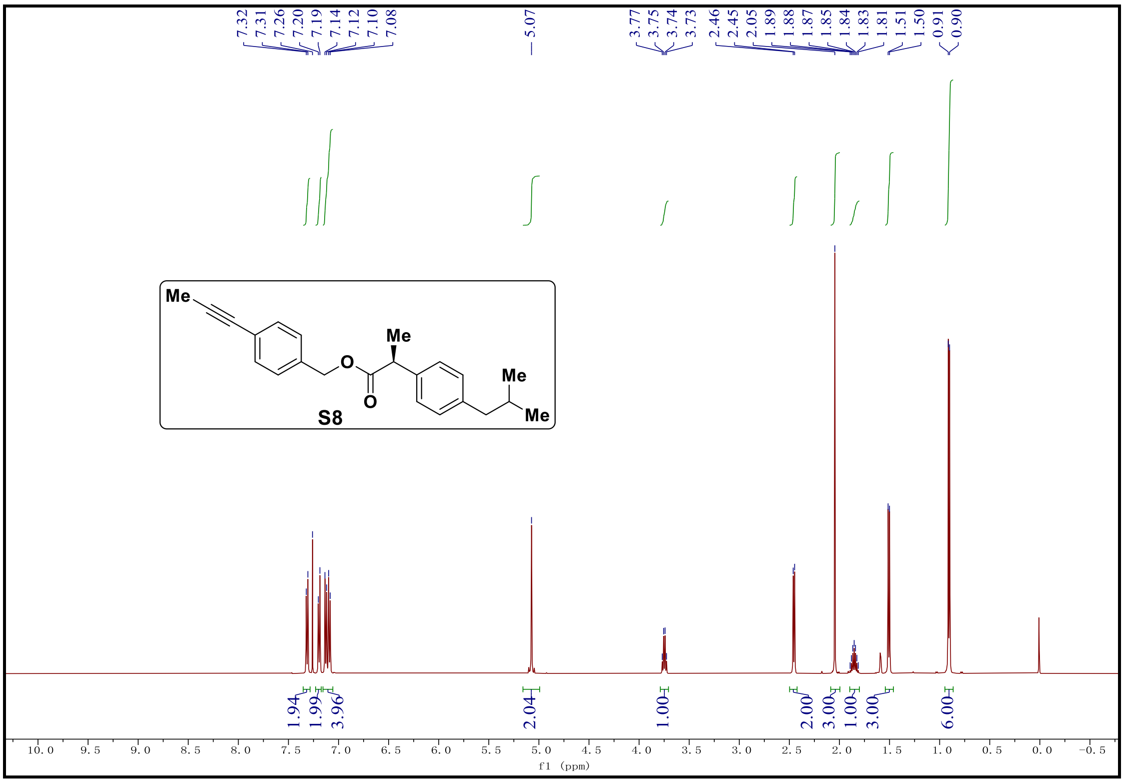

Supplementary Figure 141. ^{1}H NMR Spectrum of Compound **S8** (500 MHz, $CDCl_3$, 25 ˚C)

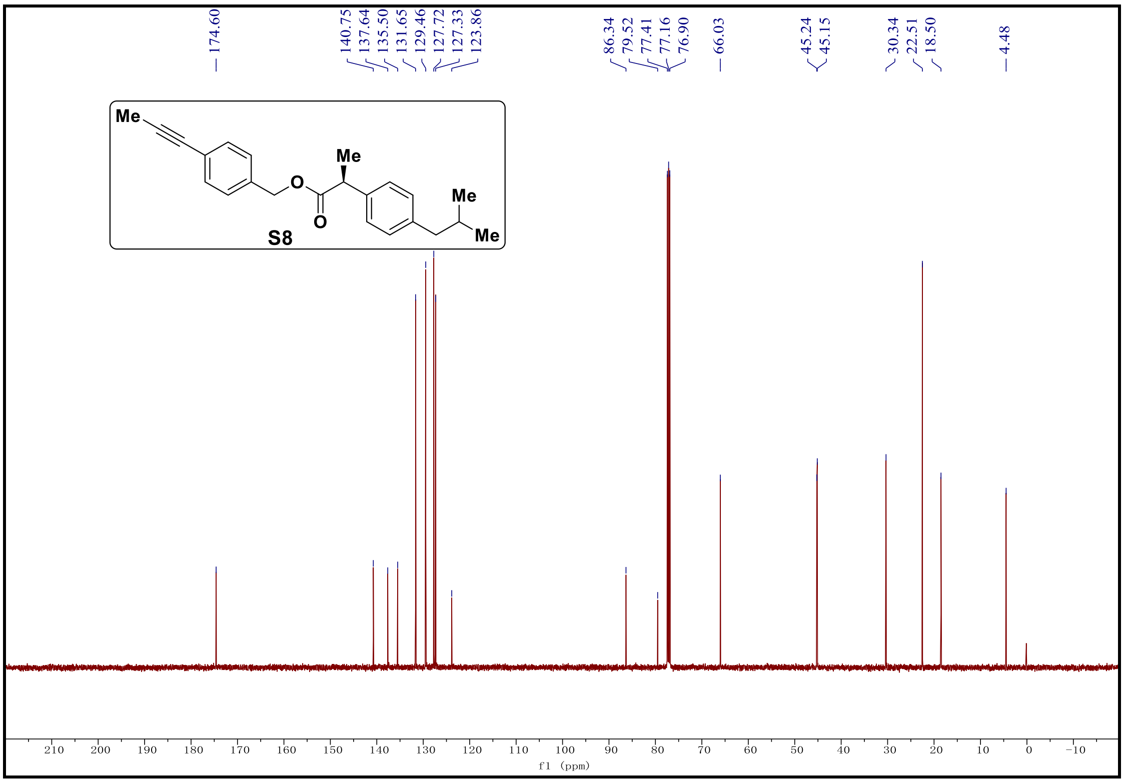

Supplementary Figure 142. ^{13}C NMR Spectrum of Compound **S8** (126 MHz, $CDCl_3$, 25 ˚C)

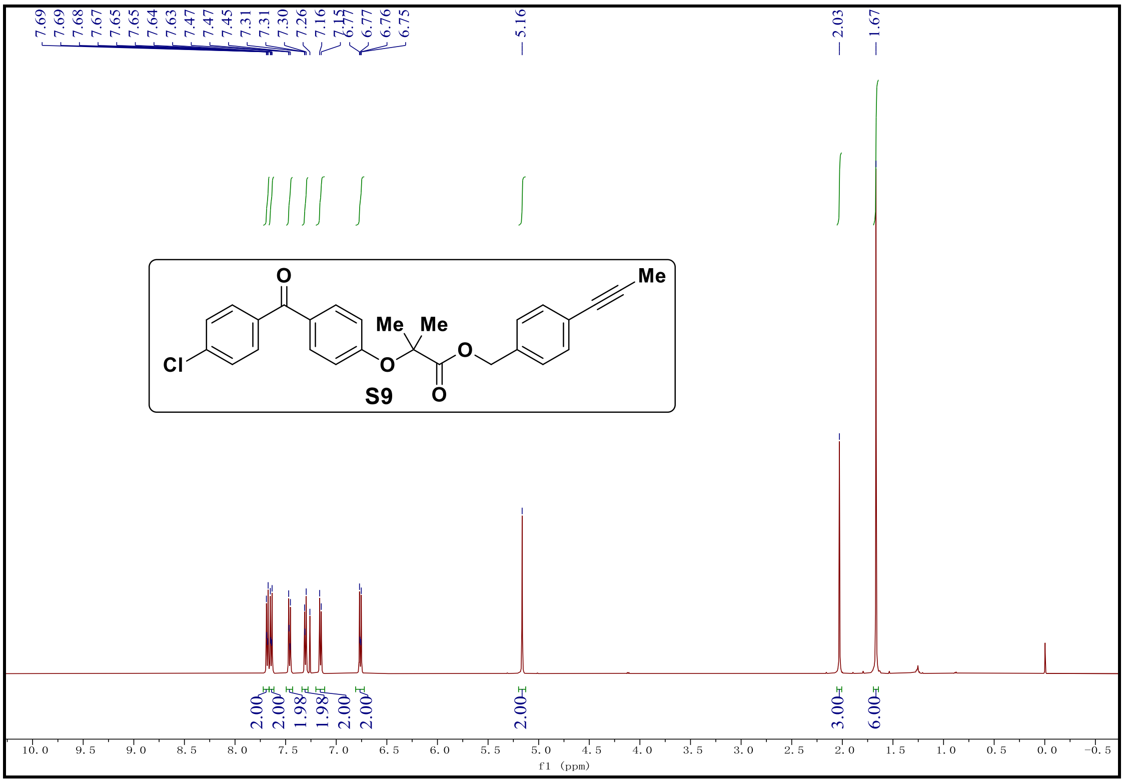

Supplementary Figure 143. ^{1}H NMR Spectrum of Compound **S9** (500 MHz, $CDCl_3$, 25 ˚C)

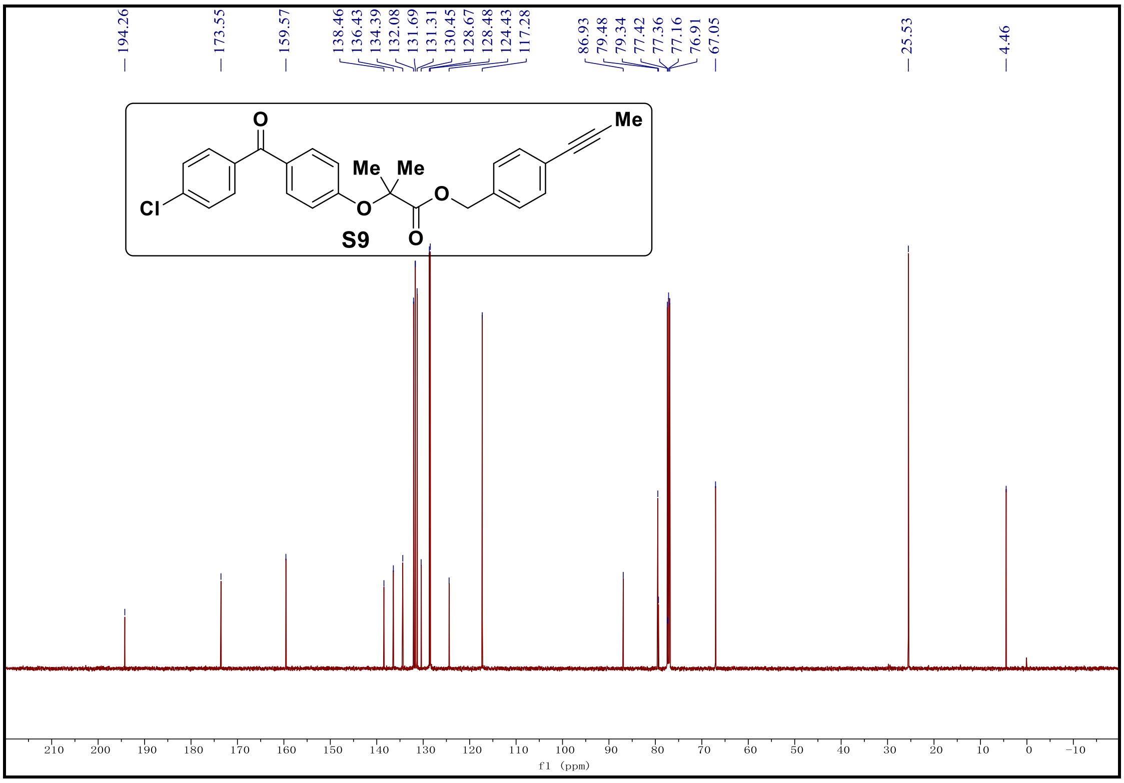

Supplementary Figure 144. ^{13}C NMR Spectrum of Compound **S9** (126 MHz, $CDCl_3$, 25 °C)

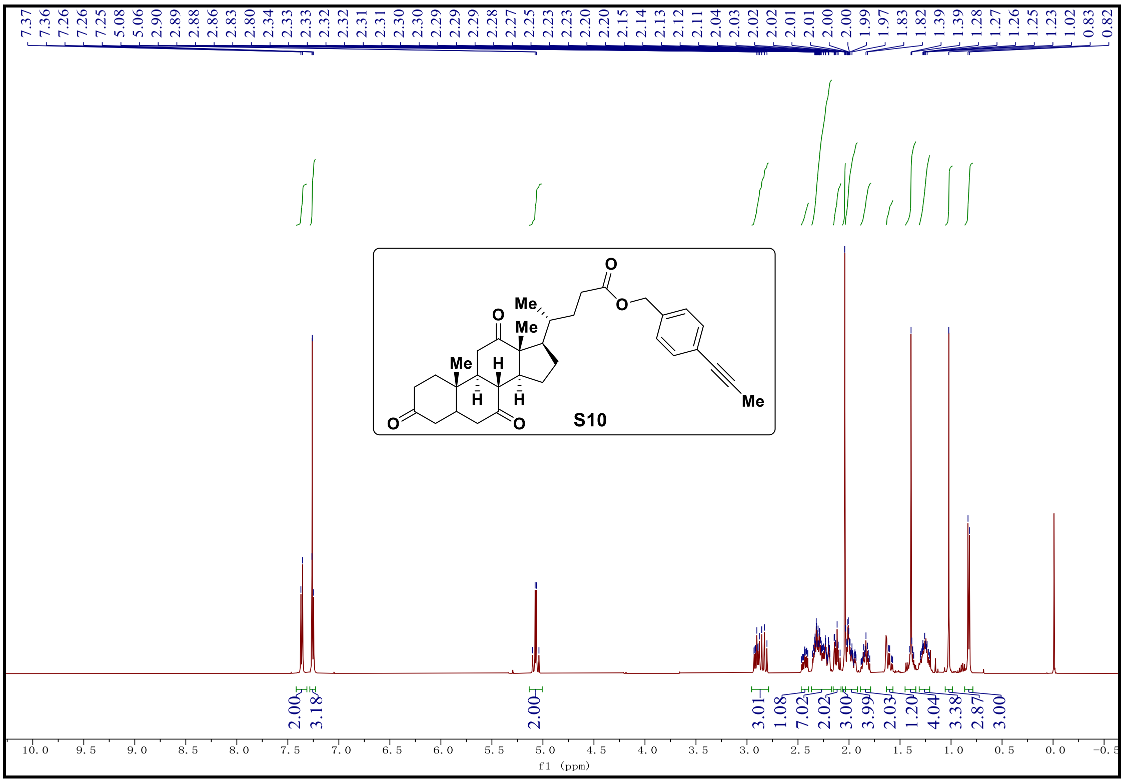

Supplementary Figure 145. ^{1}H NMR Spectrum of Compound **S10** (500 MHz, $CDCl_3$, 25 °C)

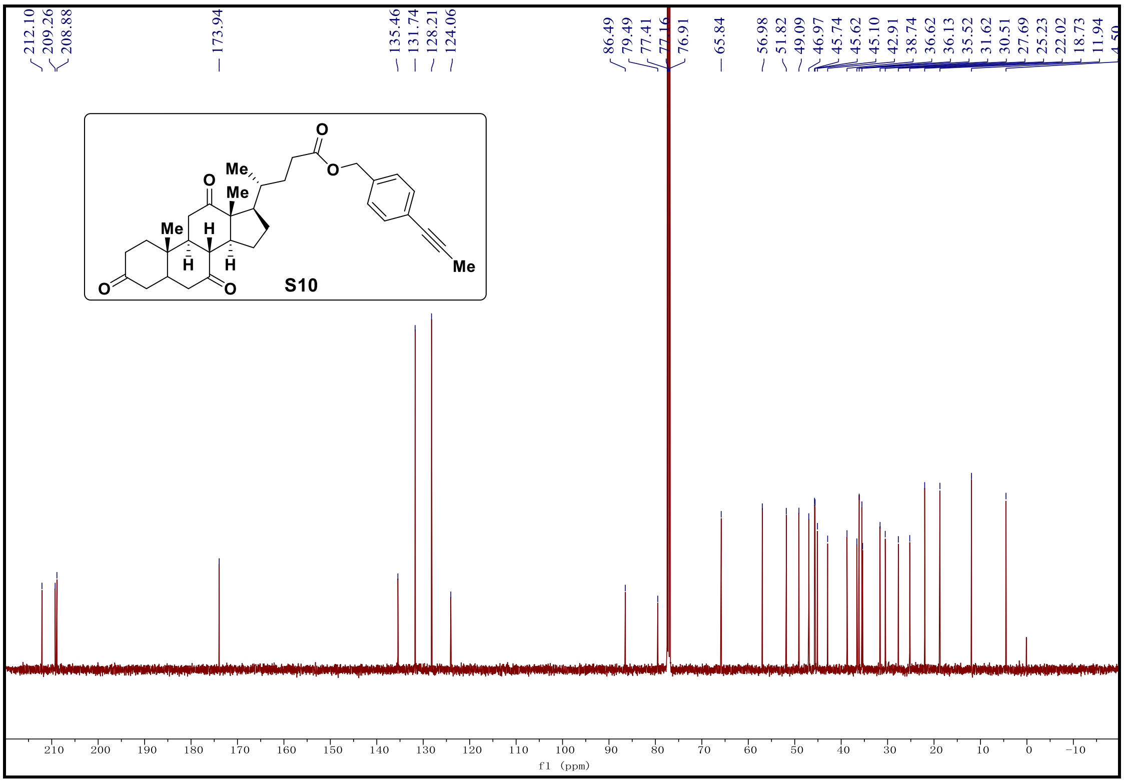

Supplementary Figure 146. ^{13}C NMR Spectrum of Compound **S10** (126 MHz, $CDCl_3$, 25 ˚C)

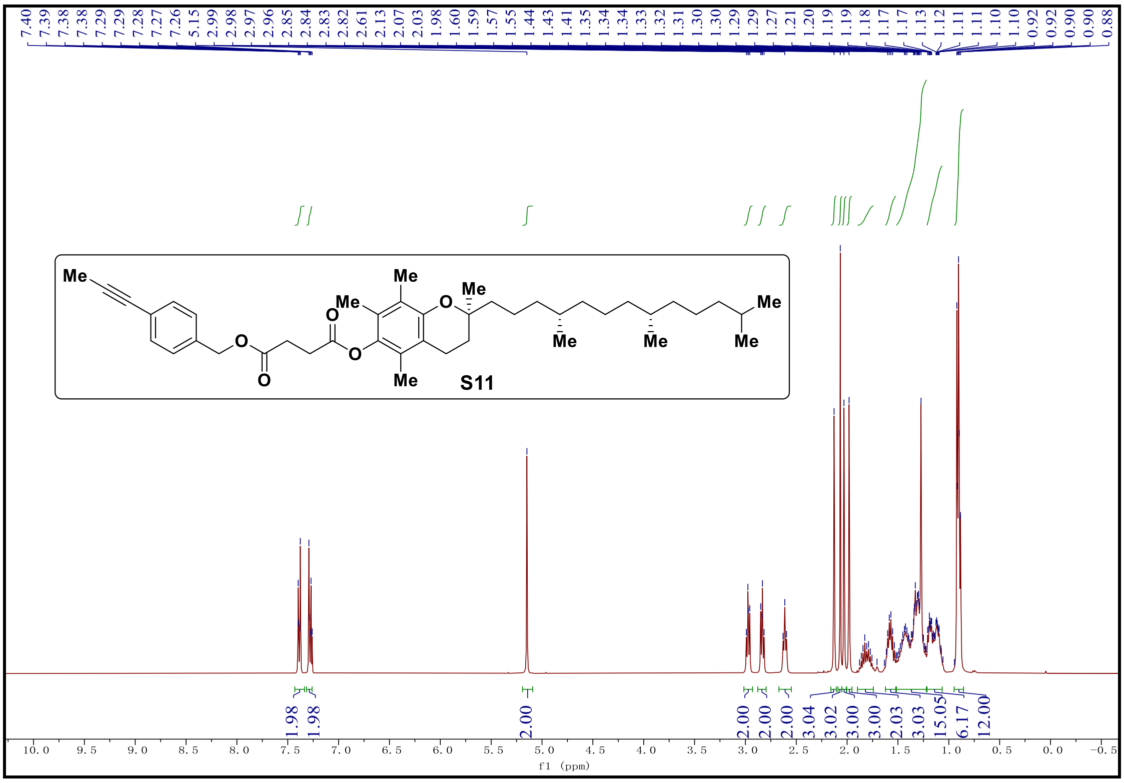

Supplementary Figure 147. ^{1}H NMR Spectrum of Compound **S11** (400 MHz, $CDCl_3$, 25 ˚C)

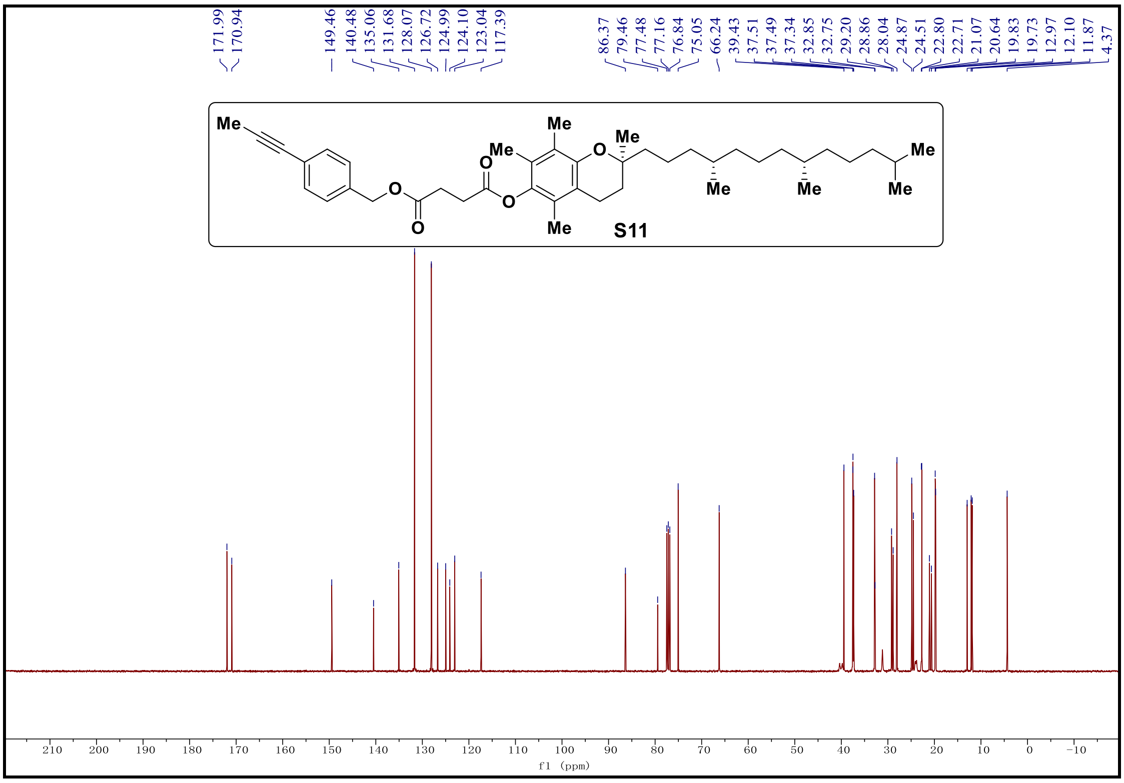

Supplementary Figure 148. ^{13}C NMR Spectrum of Compound **S11** (101 MHz, $CDCl_3$, 25 ˚C)

HPLC Data

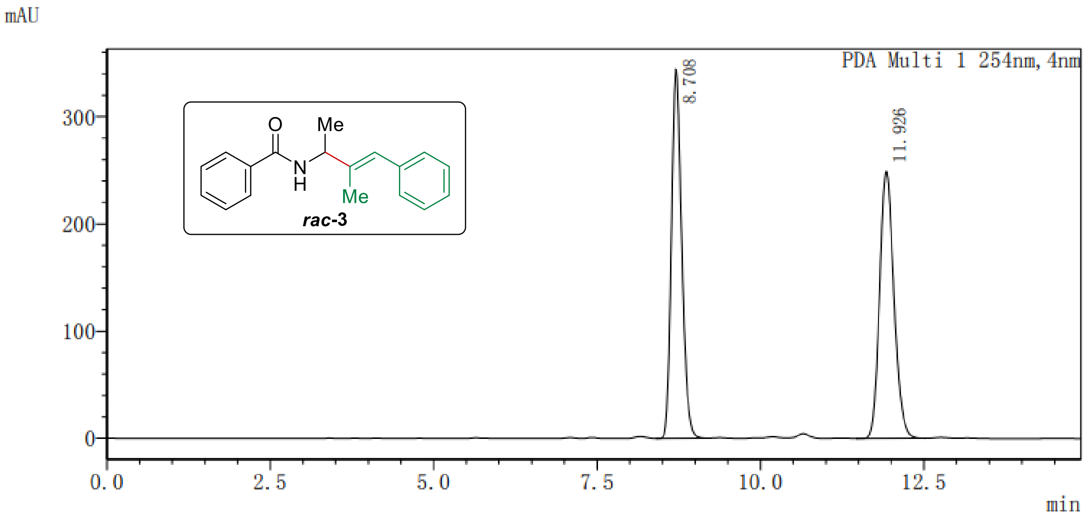

Peak#	Ret. Time	Area	Area%
1	8.708	3795610	49.974
2	11.926	3799620	50.026
Total		7595229	100.000

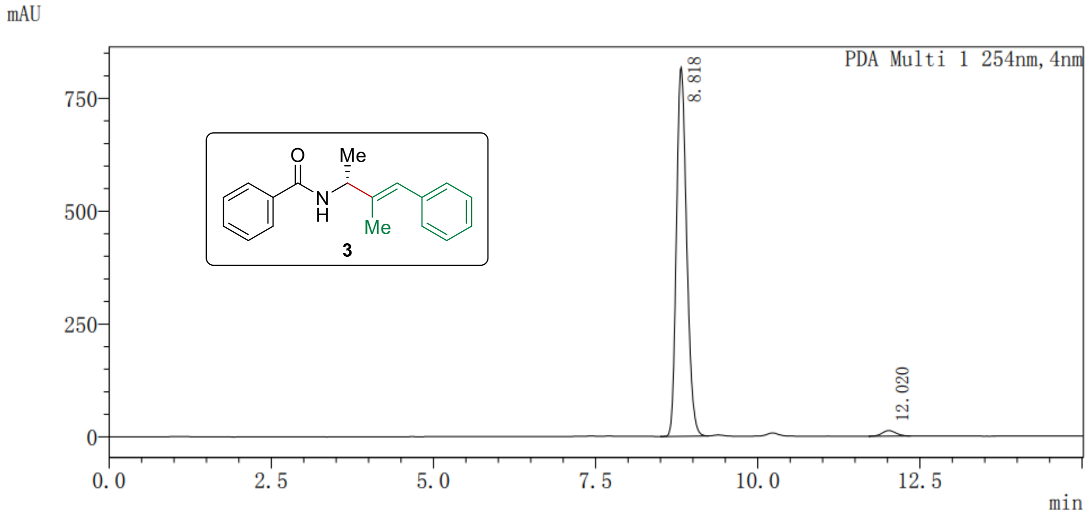

Peak#	Ret. Time	Area	Area%
1	8.818	8961690	98.031
2	12.020	179961	1.969
Total		9141650	100.000

Supplementary Figure 149. Chiral HPLC analysis of Compound **3**

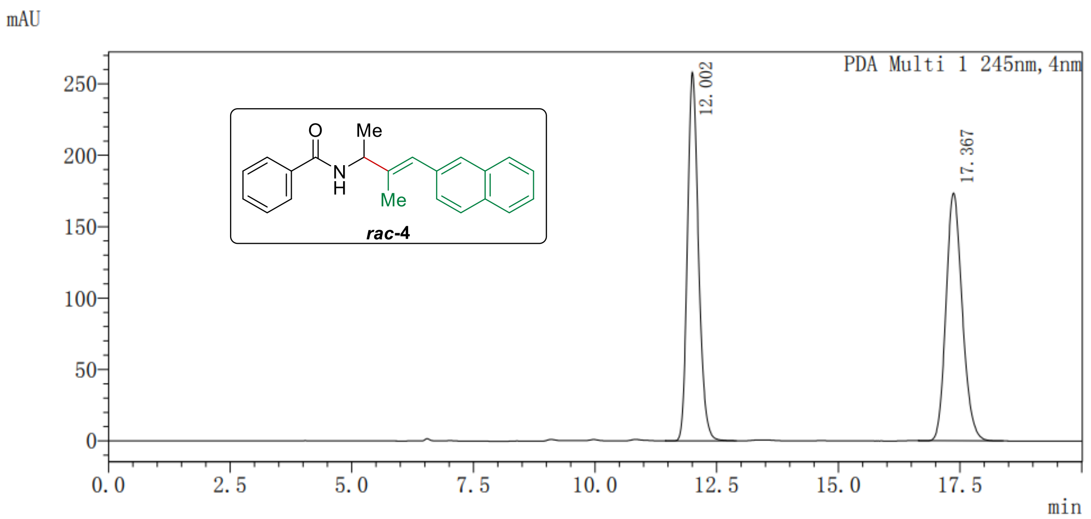

Peak#	Ret. Time	Area	Area%
1	12.002	4063198	50.210
2	17.367	4029286	49.790
Total		8092484	100

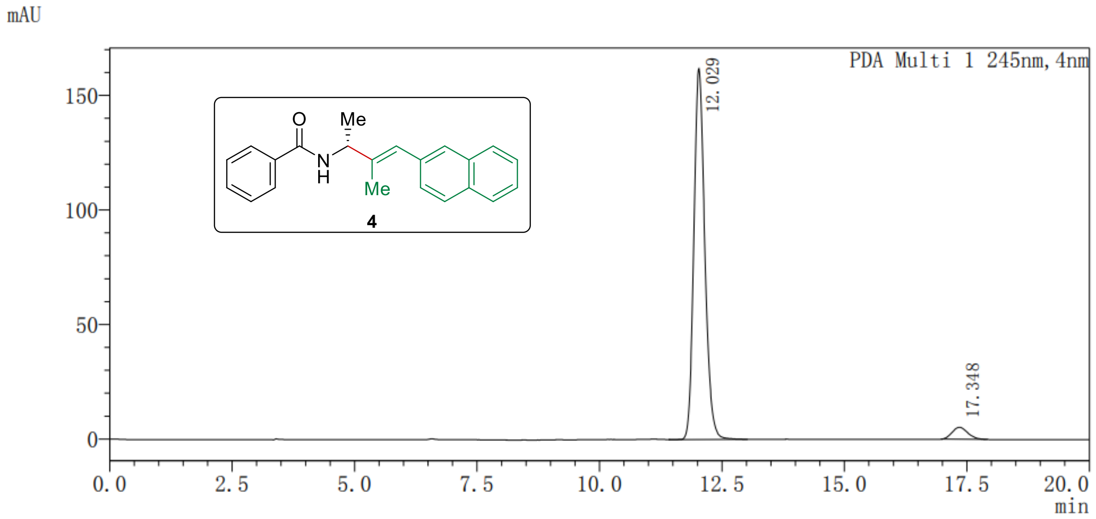

Peak#	Ret. Time	Area	Area%
1	12.029	2517835	95.595
2	17.348	116008	4.405
Total		2633842	100

Supplementary Figure 150. Chiral HPLC analysis of Compound **4**

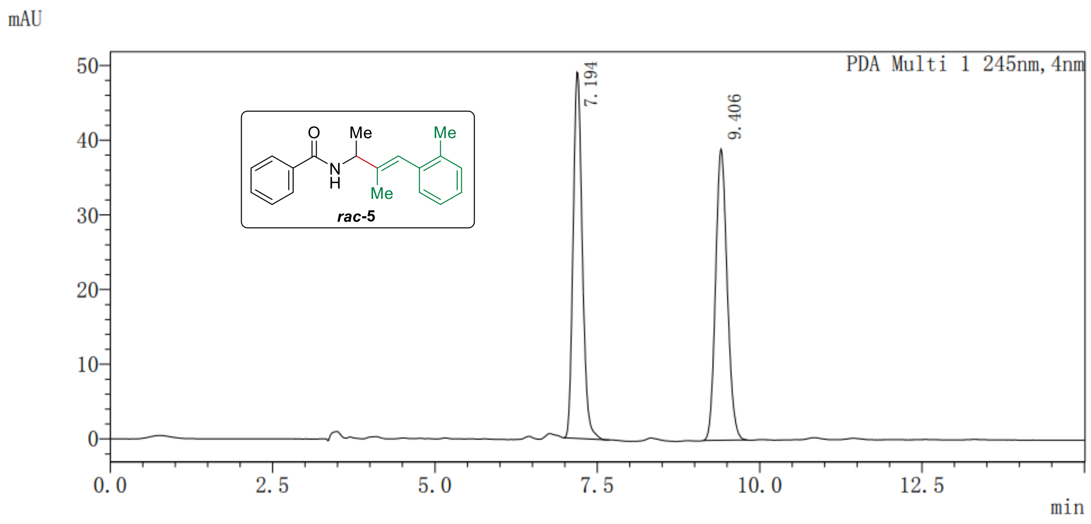

Peak#	Ret. Time	Area	Area%
1	7.194	481060	49.814
2	9.406	484646	50.186
Total		965706	100

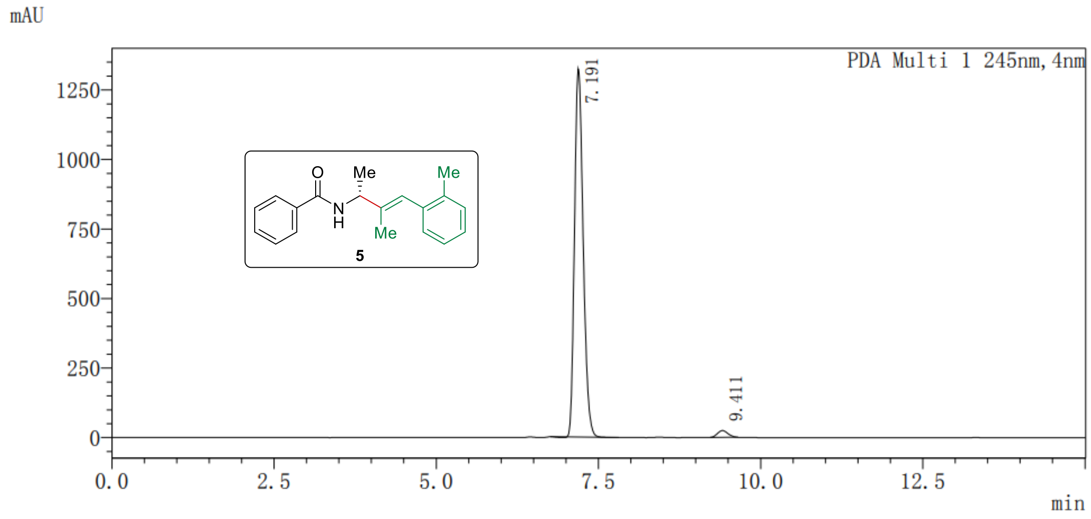

Peak#	Ret. Time	Area	Area%
1	7.191	12313483	97.865
2	9.411	268614	2.135
Total		12582097	100

Supplementary Figure 151. Chiral HPLC analysis of Compound **5**

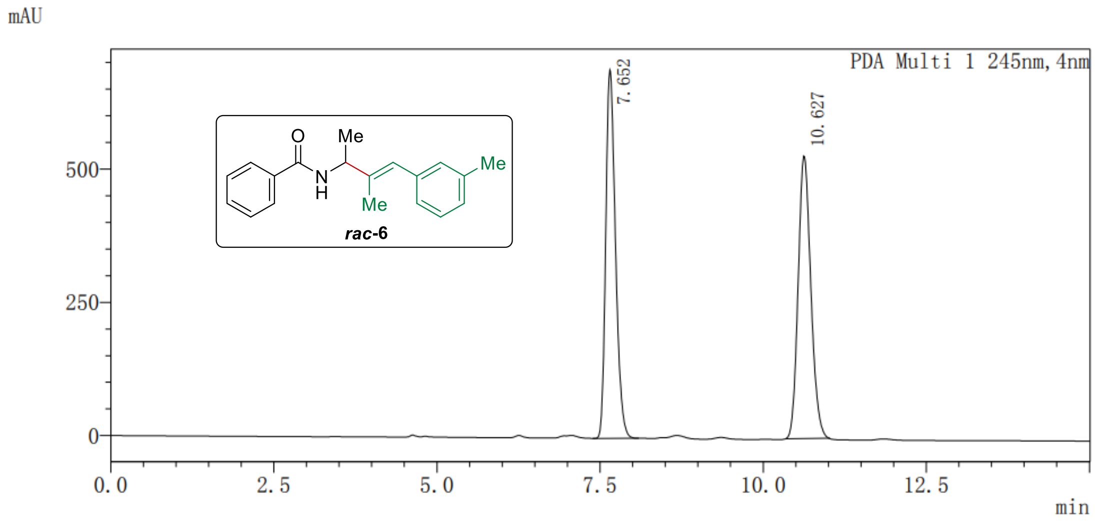

Peak#	Ret. Time	Area	Area%
1	7.652	7157775	49.967
2	10.627	7167101	50.033
Total		14324877	100

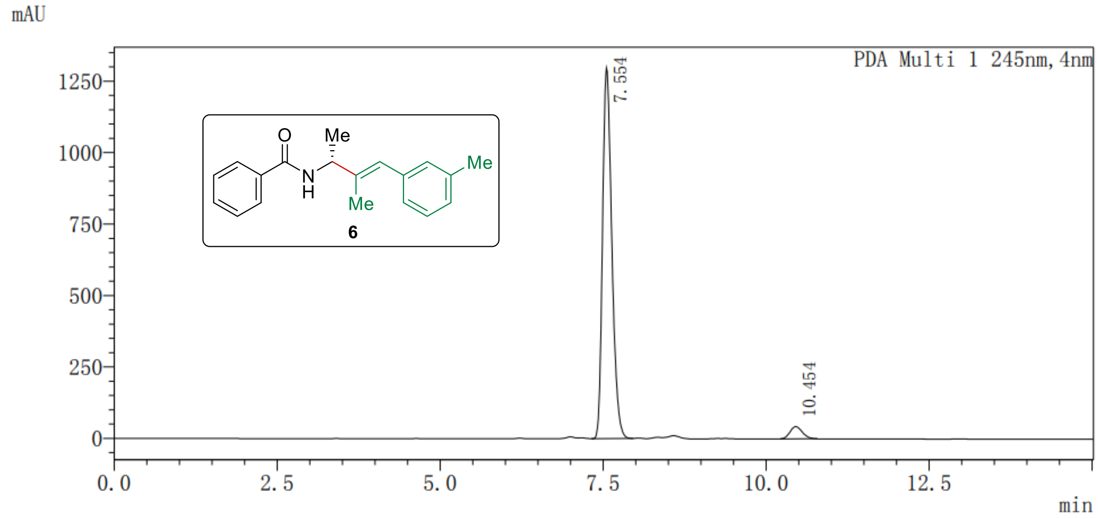

Peak#	Ret. Time	Area	Area%
1	7.554	12845209	95.783
2	10.454	565592	4.217
Total		13410801	100

Supplementary Figure 152. Chiral HPLC analysis of Compound **6**

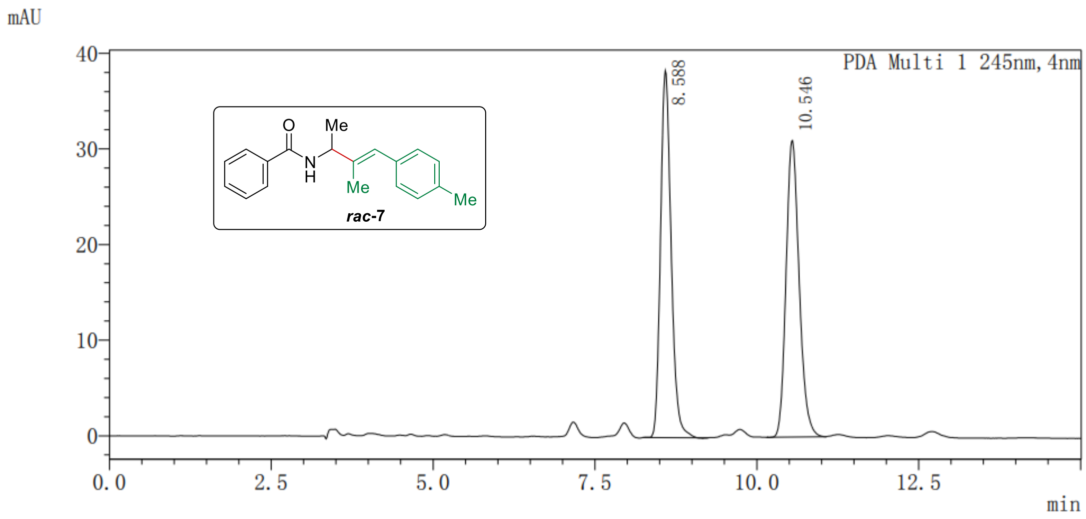

Peak#	Ret. Time	Area	Area%
1	8.588	437877	50.074
2	10.546	436578	49.926
Total		874456	100

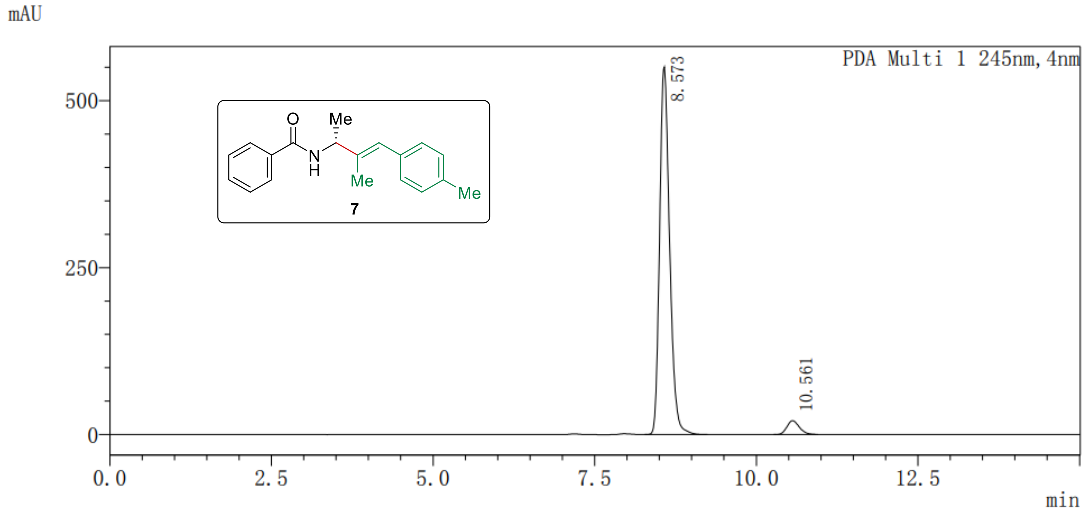

Peak#	Ret. Time	Area	Area%
1	8.573	5918758	95.534
2	10.561	276666	4.466
Total		6195424	100

Supplementary Figure 153. Chiral HPLC analysis of Compound **7**

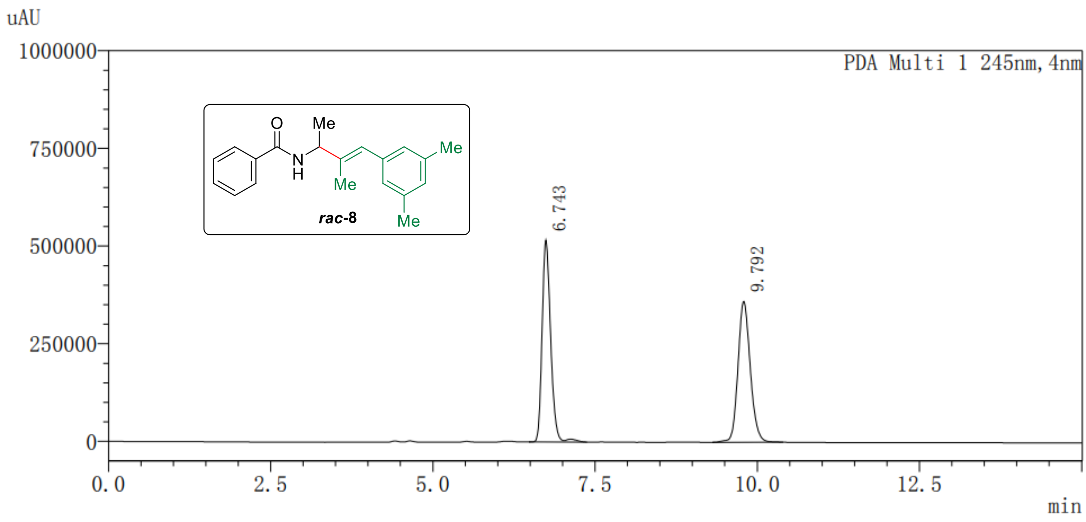

Peak#	Ret. Time	Area	Area%
1	6.743	4763048	50.048
2	9.792	4753911	49.952
Total		9516959	100

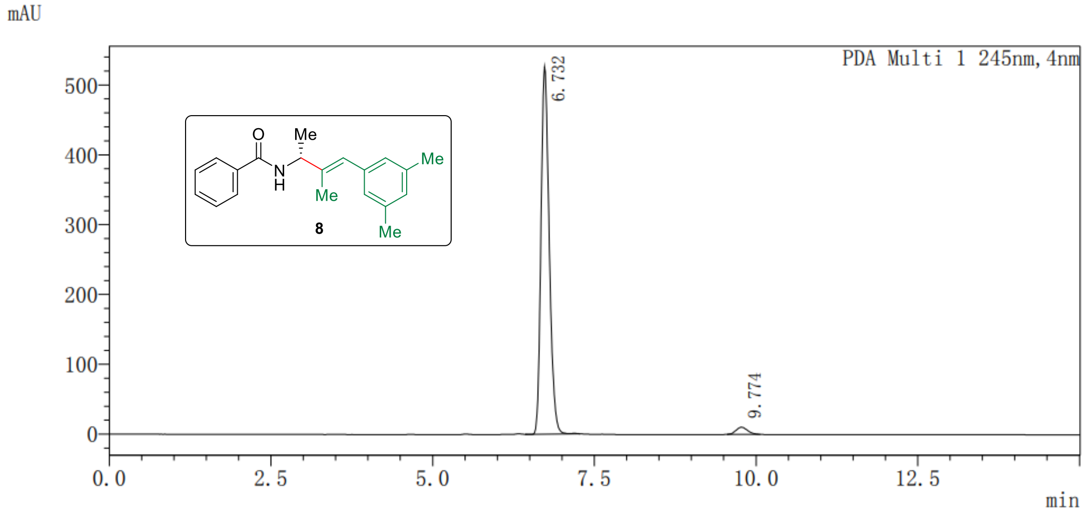

Peak#	Ret. Time	Area	Area%
1	6.732	4646427	97.352
2	9.774	126364	2.648
Total		4772791	100

Supplementary Figure 154. Chiral HPLC analysis of Compound **8**

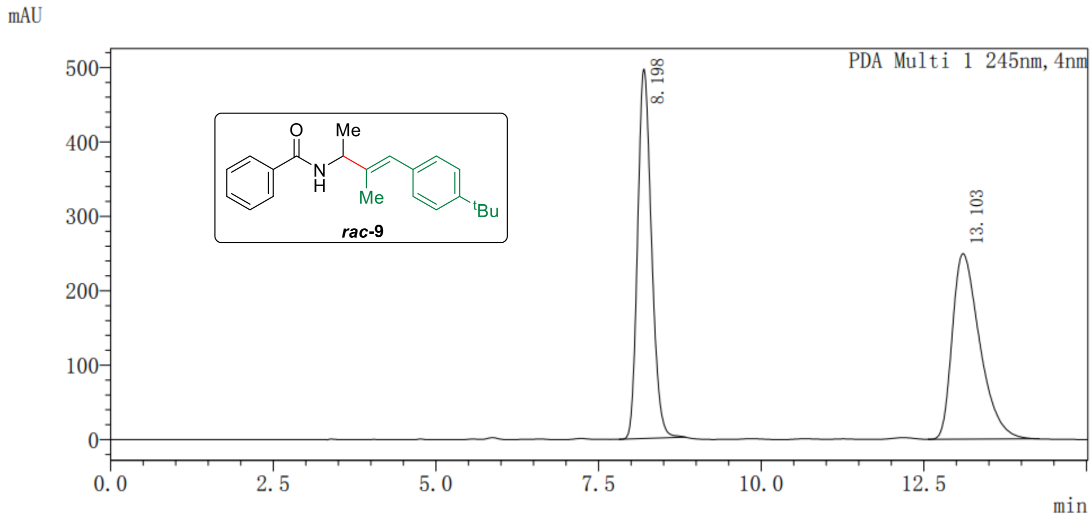

Peak#	Ret. Time	Area	Area%
1	8.198	7363858	50.109
2	13.103	7331918	49.891
Total		14695776	100.000

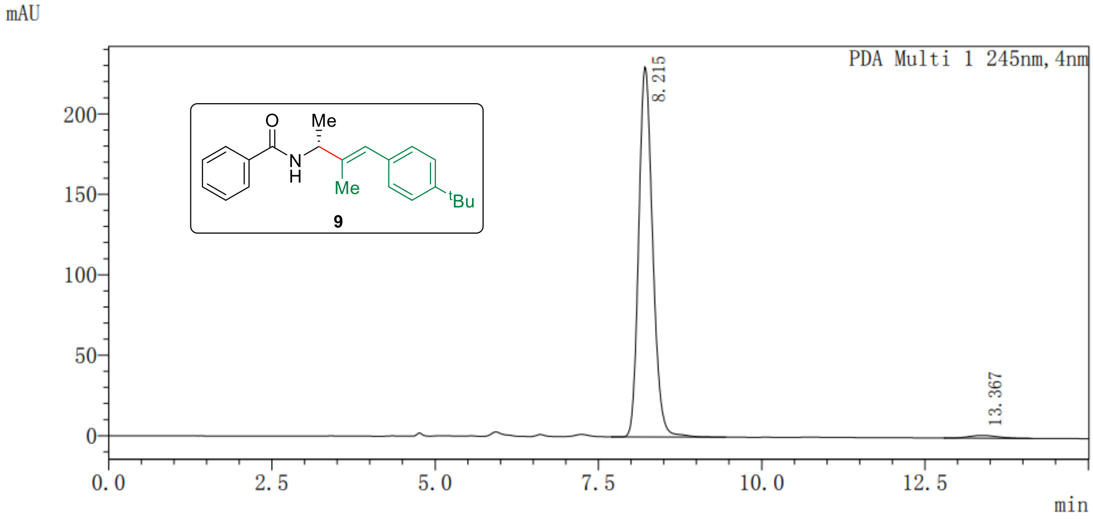

Peak#	Ret. Time	Area	Area%
1	8.215	3410126	98.446
2	13.367	53829	1.554
Total		3463956	100.000

Supplementary Figure 155. Chiral HPLC analysis of Compound **9**

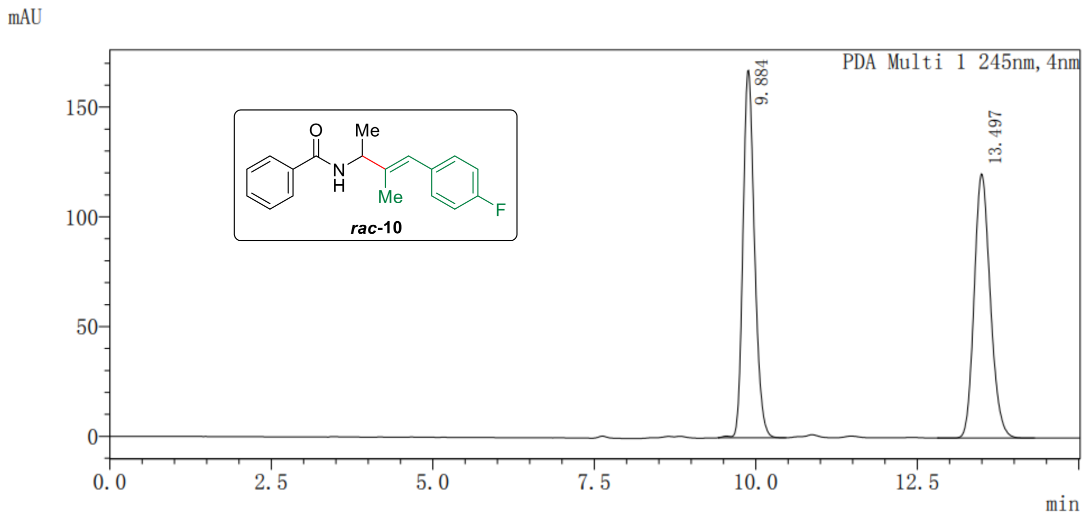

Peak#	Ret. Time	Area	Area%
1	9.884	2081941	49.942
2	13.497	2086793	50.058
Total		4168734	100

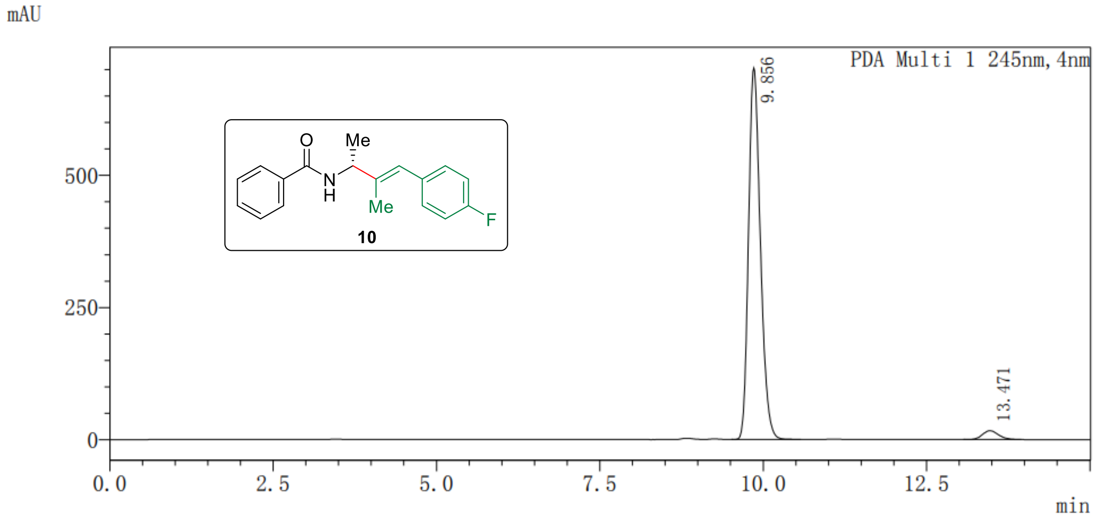

Peak#	Ret. Time	Area	Area%
1	9.856	8864251	96.845
2	13.471	288732	3.155
Total		9152983	100

Supplementary Figure 156. Chiral HPLC analysis of Compound **10**

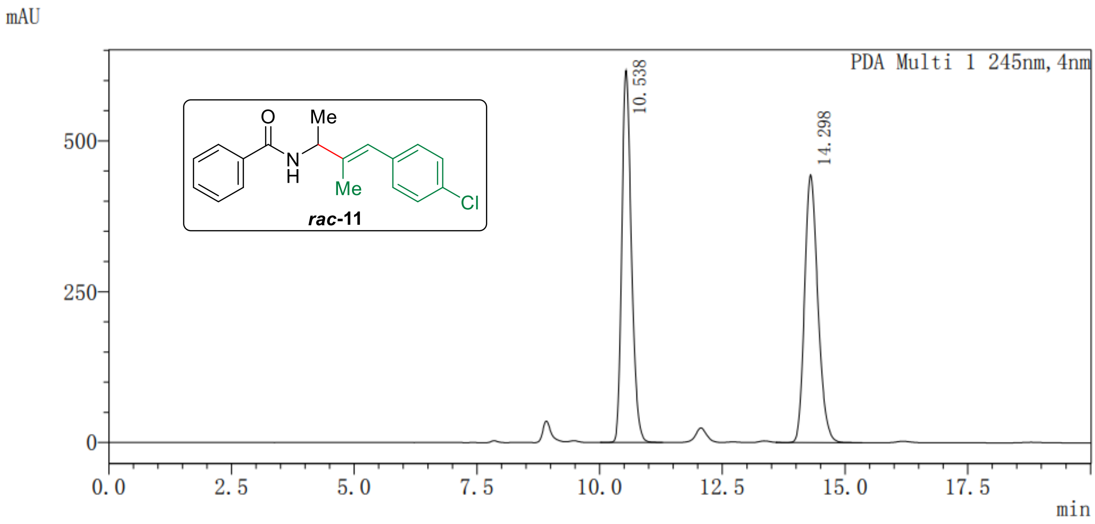

Peak#	Ret. Time	Area	Area%
1	10.538	8370342	50.029
2	14.298	8360515	49.971
Total		16730857	100

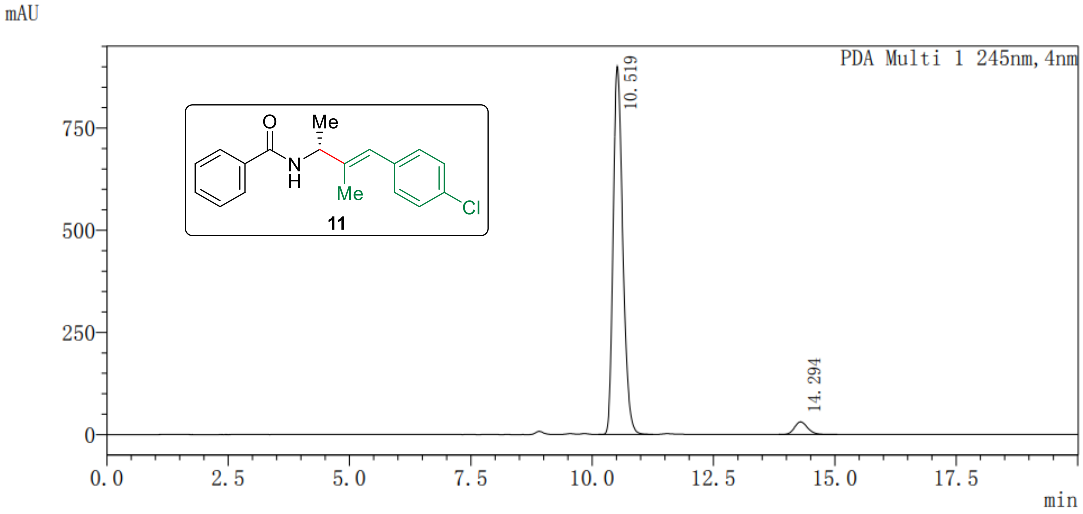

Peak#	Ret. Time	Area	Area%
1	10.519	12451471	95.542
2	14.294	580984	4.458
Total		13032455	100

Supplementary Figure 157. Chiral HPLC analysis of Compound **11**

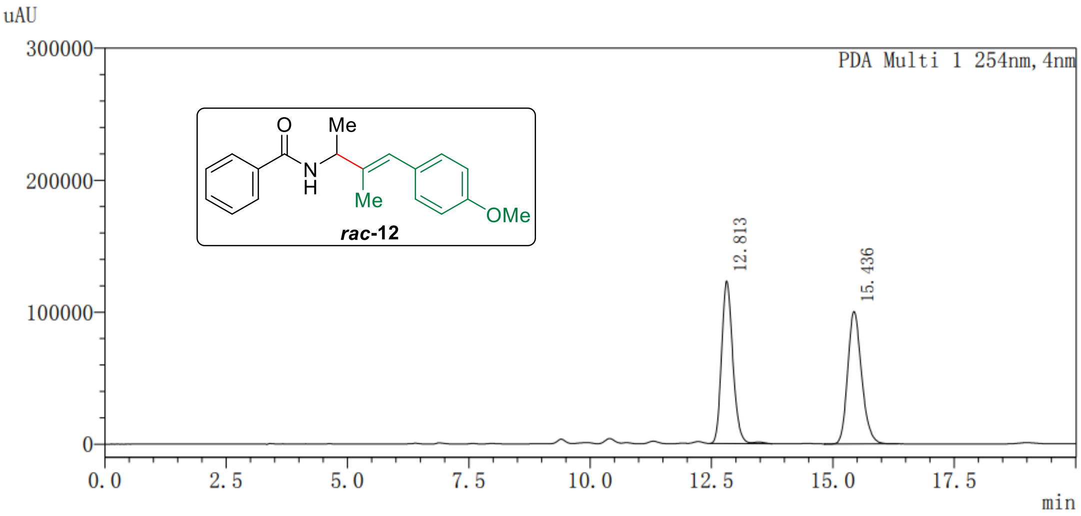

Peak#	Ret. Time	Area	Area%
1	12.813	1991081	49.805
2	15.436	2006645	50.195
Total		3997725	100

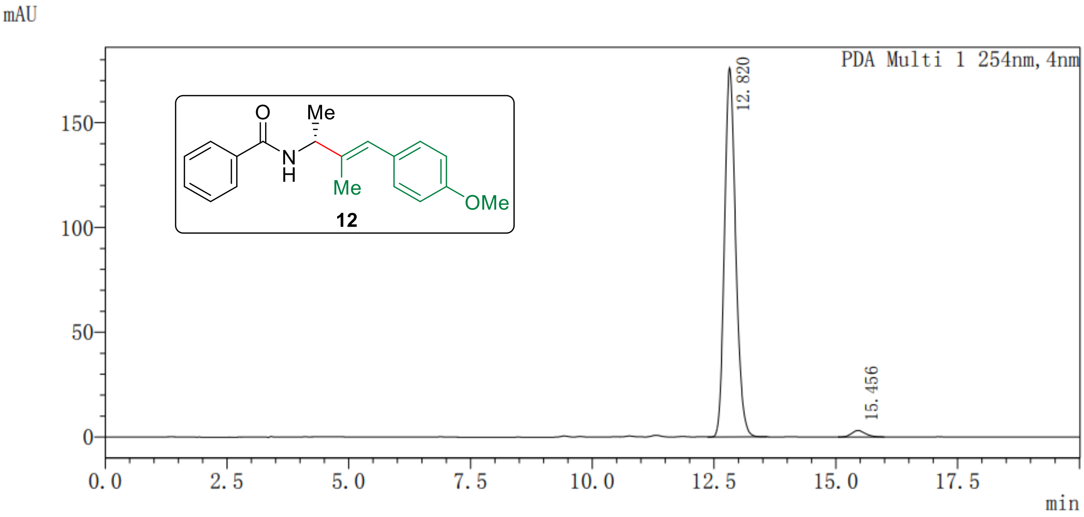

Peak#	Ret. Time	Area	Area%
1	12.820	2809415	97.901
2	15.456	60232	2.099
Total		2869647	100

Supplementary Figure 158. Chiral HPLC analysis of Compound **12**

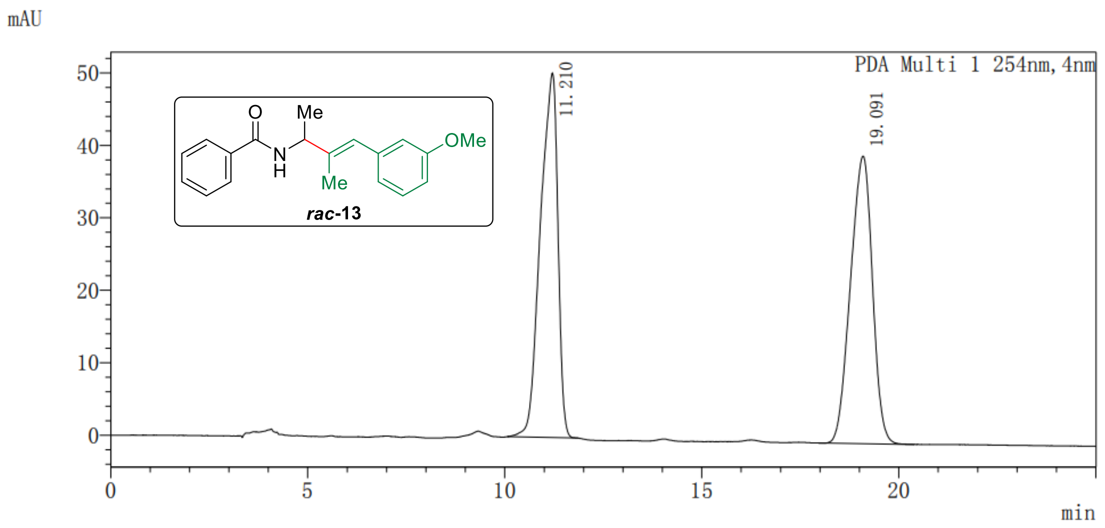

Peak#	Ret. Time	Area	Area%
1	11.210	1545182	50.168
2	19.091	1534823	49.832
Total		3080004	100

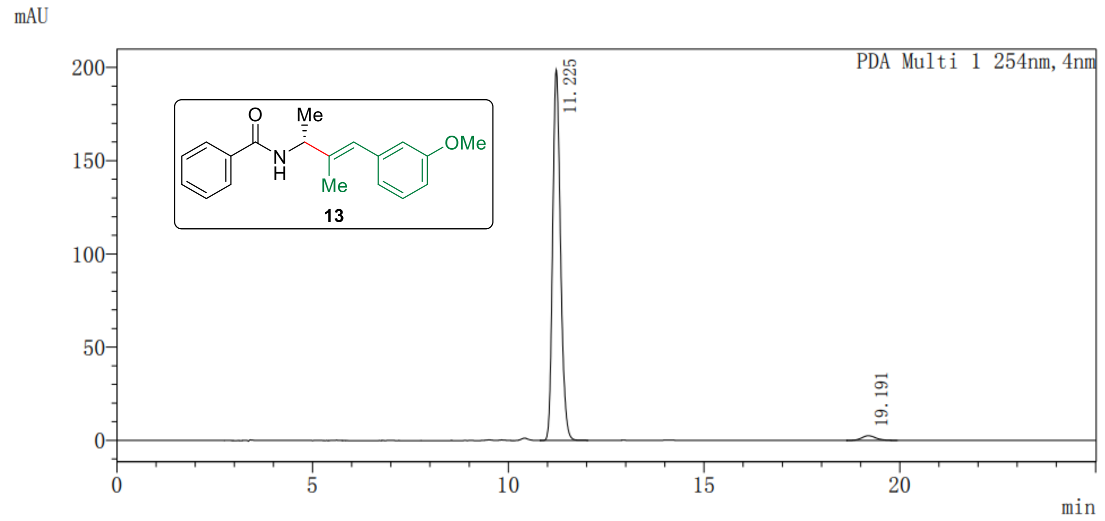

Peak#	Ret. Time	Area	Area%
1	11.225	2765978	97.832
2	19.191	61289	2.168
Total		2827267	100

Supplementary Figure 159. Chiral HPLC analysis of Compound **13**

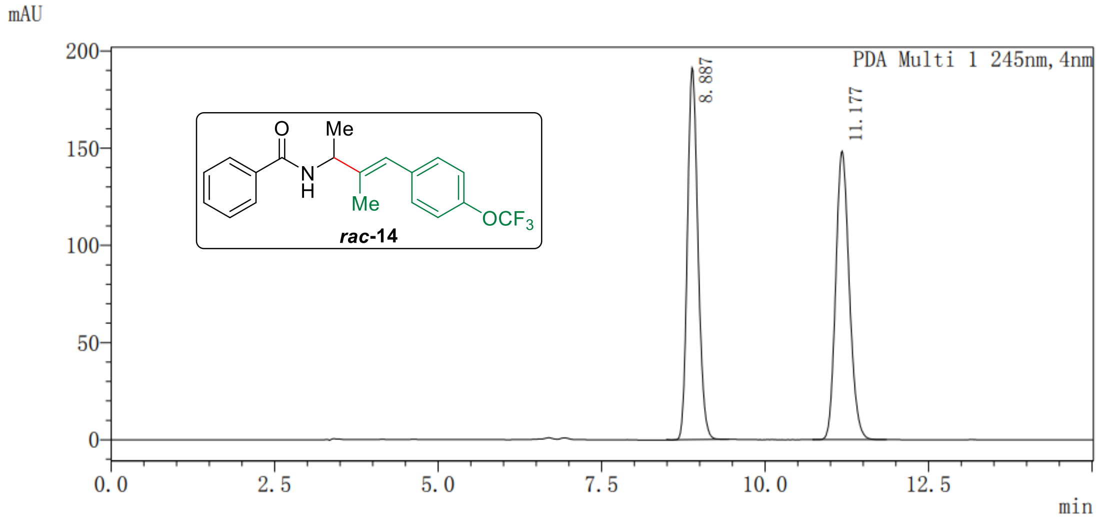

Peak#	Ret. Time	Area	Area%
1	8.887	2136619	49.987
2	11.177	2137735	50.013
Total		4274354	100

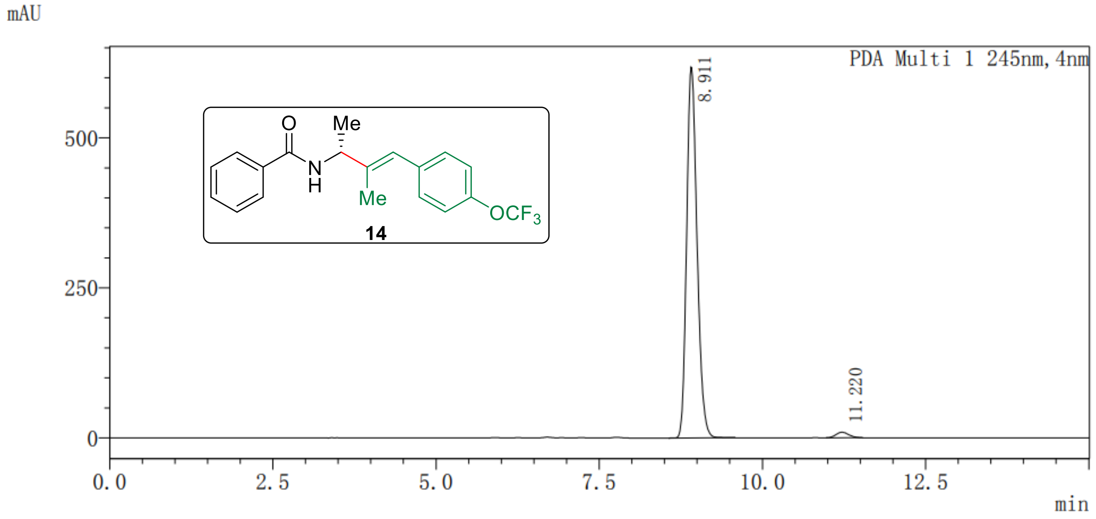

Peak#	Ret. Time	Area	Area%
1	8.911	6840070	98.194
2	11.220	125809	1.806
Total		6965879	100

Supplementary Figure 160. Chiral HPLC analysis of Compound **14**

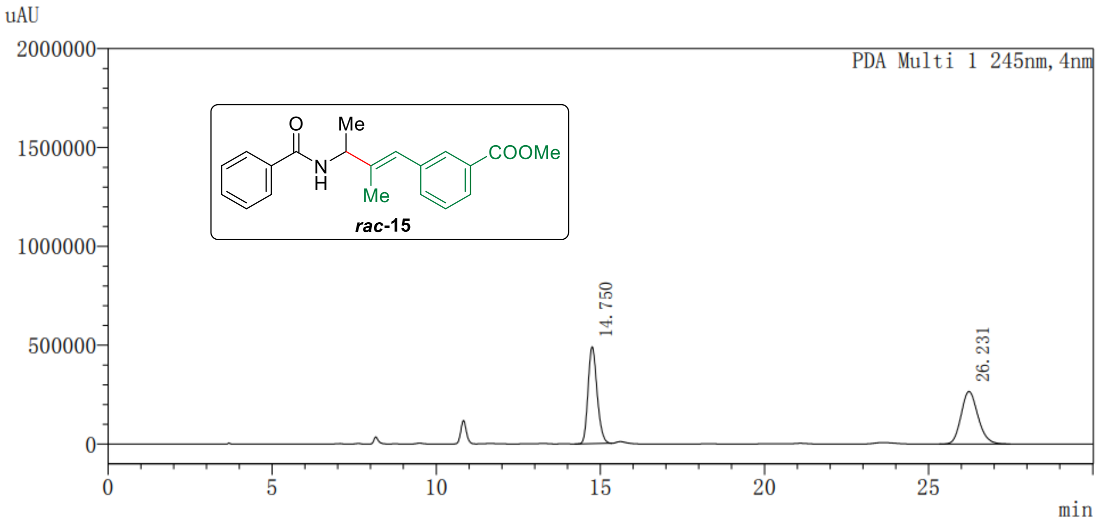

Peak#	Ret. Time	Area	Area%
1	14.750	9408509	49.771
2	26.231	9494947	50.229
Total		18903456	100

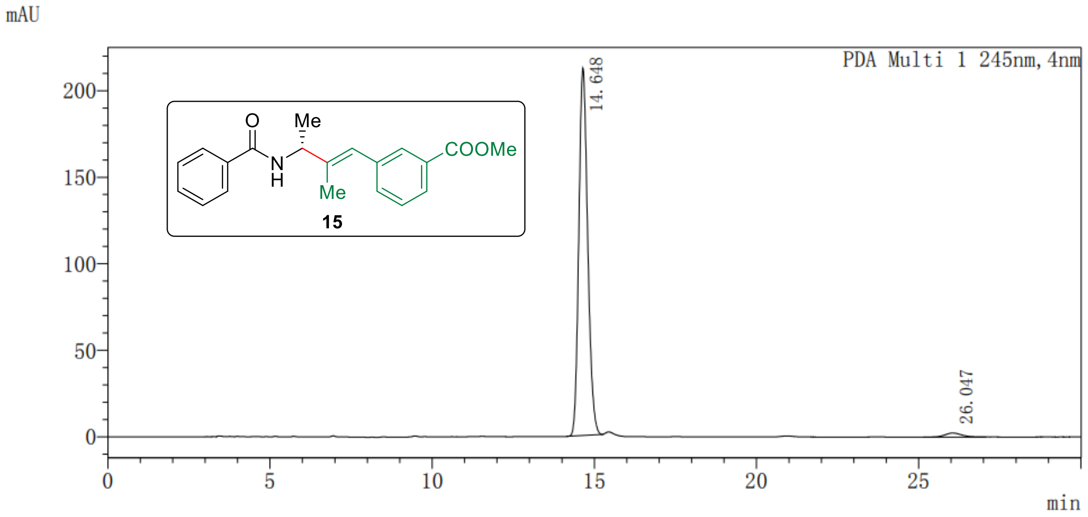

Peak#	Ret. Time	Area	Area%
1	14.648	4048146	98.005
2	26.047	82414	1.995
Total		4130560	100

Supplementary Figure 161. Chiral HPLC analysis of Compound **15**

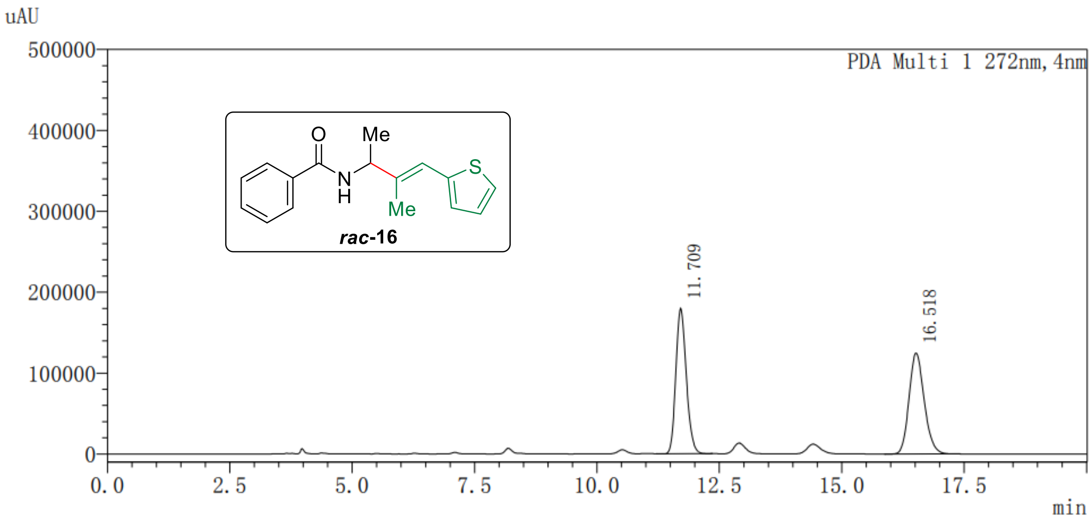

Peak#	Ret. Time	Area	Area%
1	11.709	2673681	49.821
2	16.518	2692909	50.179
Total		5366589	100

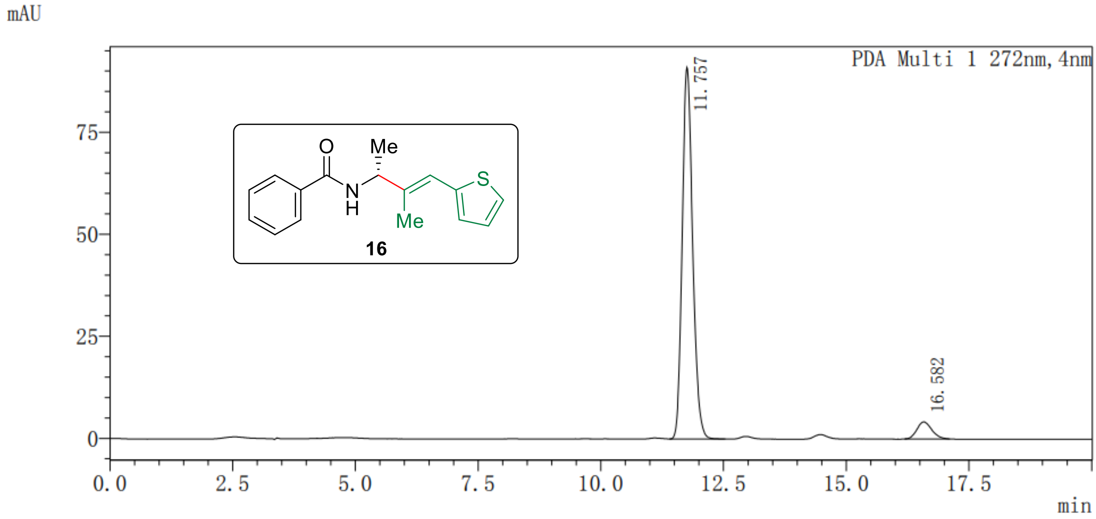

Peak#	Ret. Time	Area	Area%
1	11.757	1346294	93.873
2	16.582	87873	6.127
Total		1434167	100

Supplementary Figure 162. Chiral HPLC analysis of Compound **16**

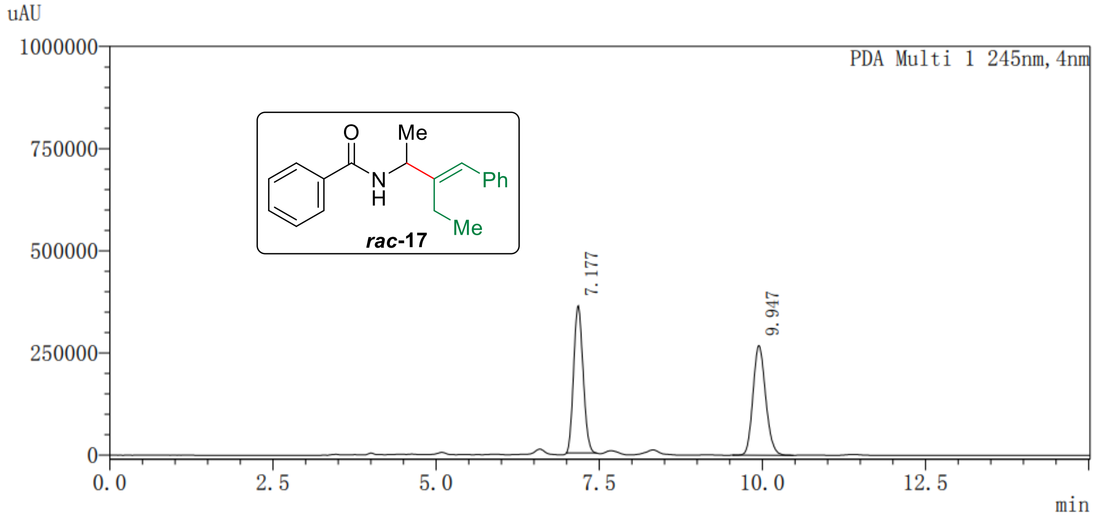

Peak#	Ret. Time	Area	Area%
1	7.177	3637432	50.104
2	9.947	3622404	49.896
Total		7259836	100

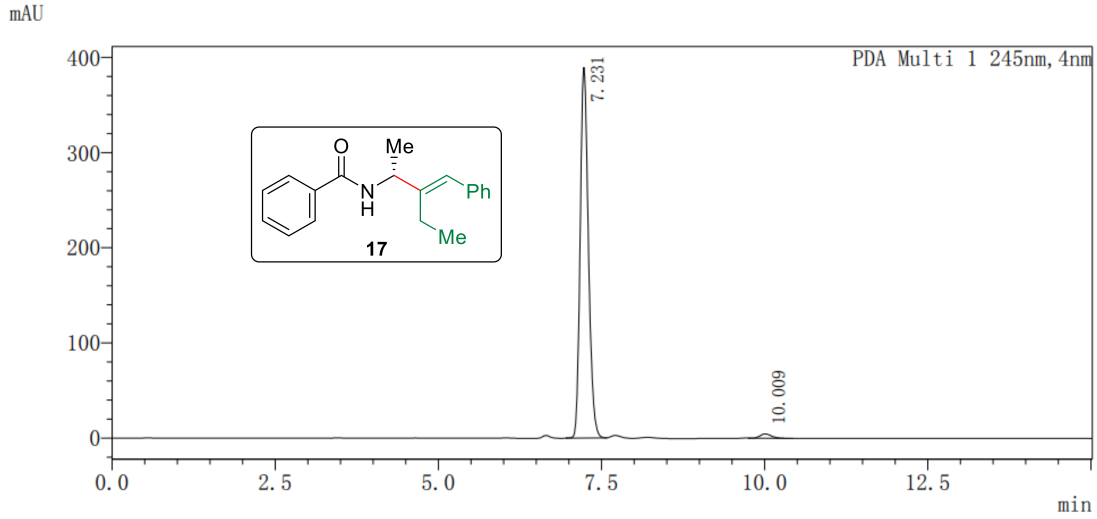

Peak#	Ret. Time	Area	Area%
1	7.231	3412879	98.481
2	10.009	52631	1.519
Total		3465510	100

Supplementary Figure 163. Chiral HPLC analysis of Compound **17**

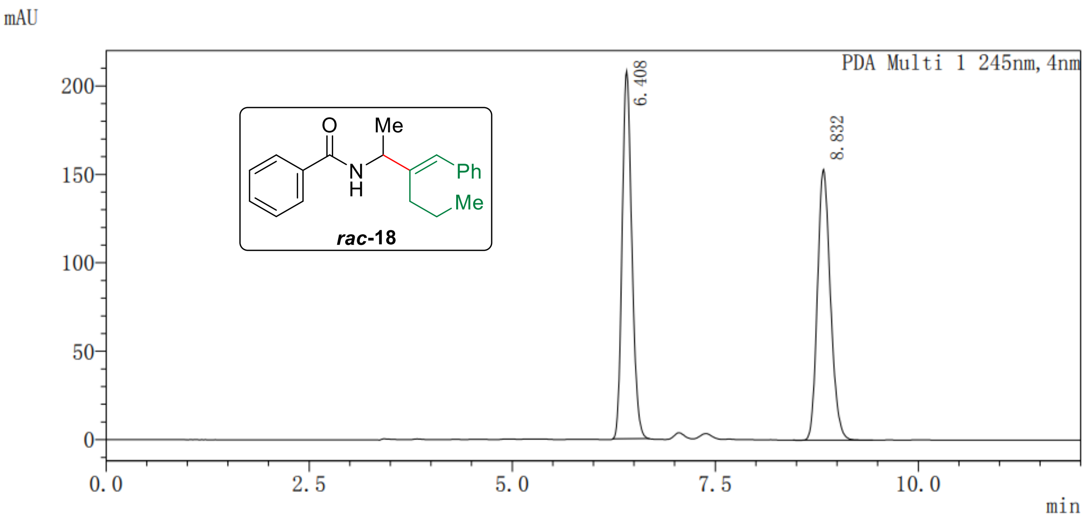

Peak#	Ret. Time	Area	Area%
1	6.408	1726273	50.284
2	8.832	1706787	49.716
Total		3433060	100

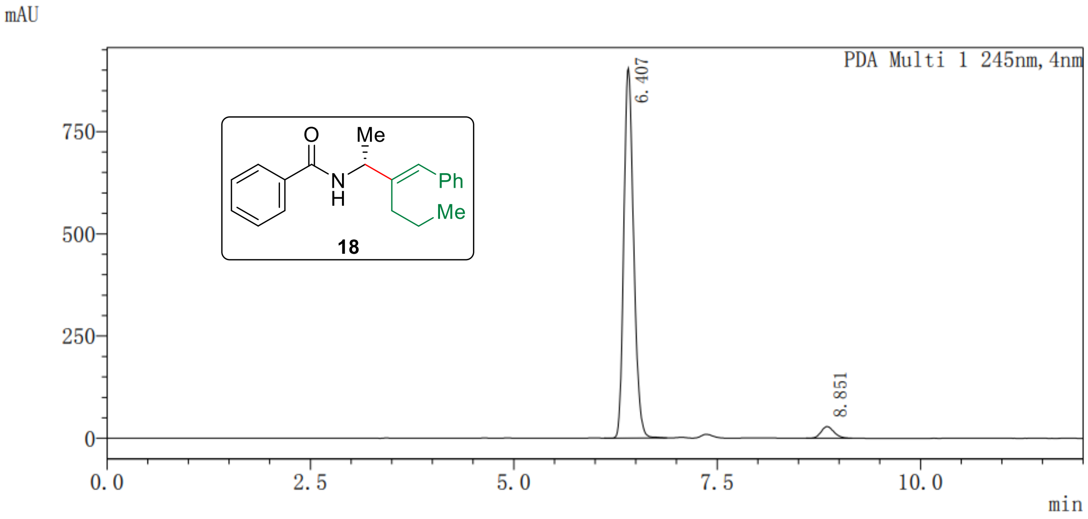

Peak#	Ret. Time	Area	Area%
1	6.407	7618177	96.027
2	8.851	315191	3.973
Total		7933367	100

Supplementary Figure 164. Chiral HPLC analysis of Compound **18**

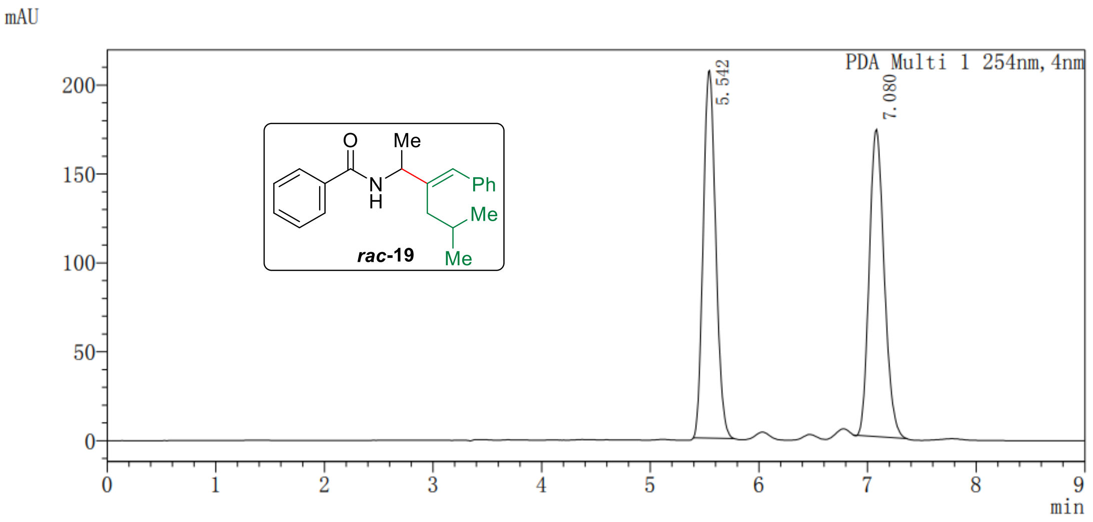

Peak#	Ret. Time	Area	Area%
1	5.542	1654429	49.984
2	7.080	1655517	50.016
Total		3309946	100

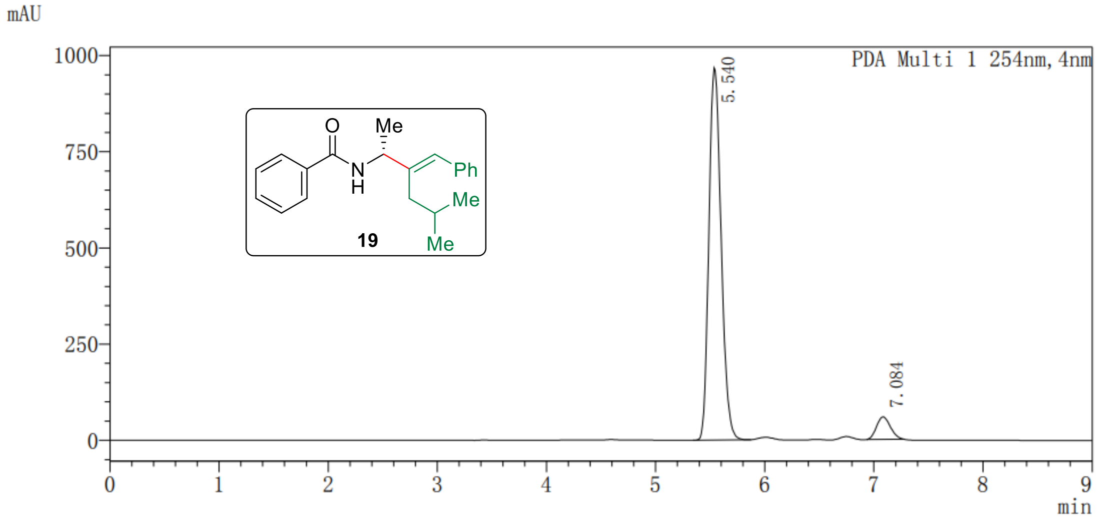

Peak#	Ret. Time	Area	Area%
1	5.540	7533294	93.570
2	7.084	517687	6.430
Total		8050981	100

Supplementary Figure 165. Chiral HPLC analysis of Compound **19**

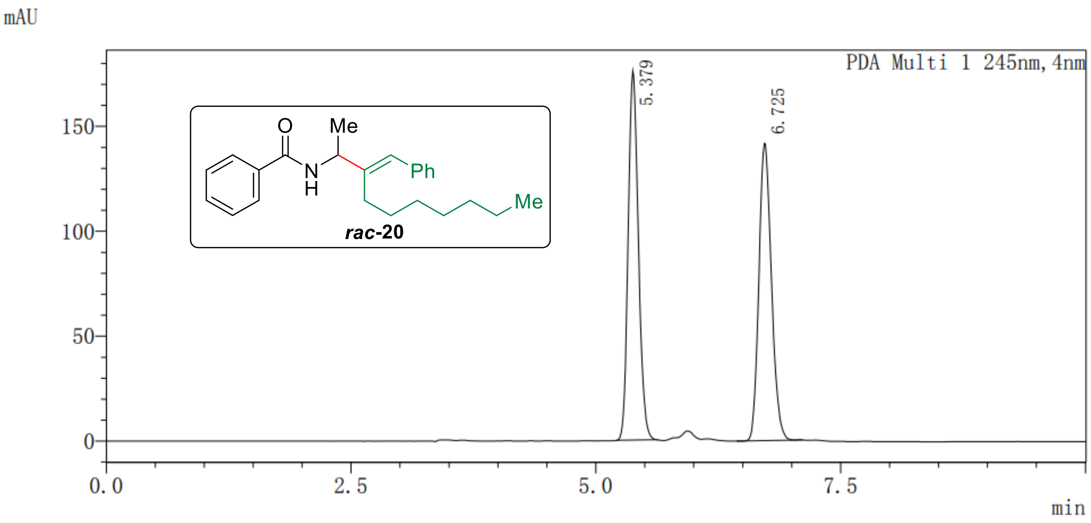

Peak#	Ret. Time	Area	Area%
1	5.379	1275575	50.279
2	6.725	1261405	49.721
Total		2536980	100

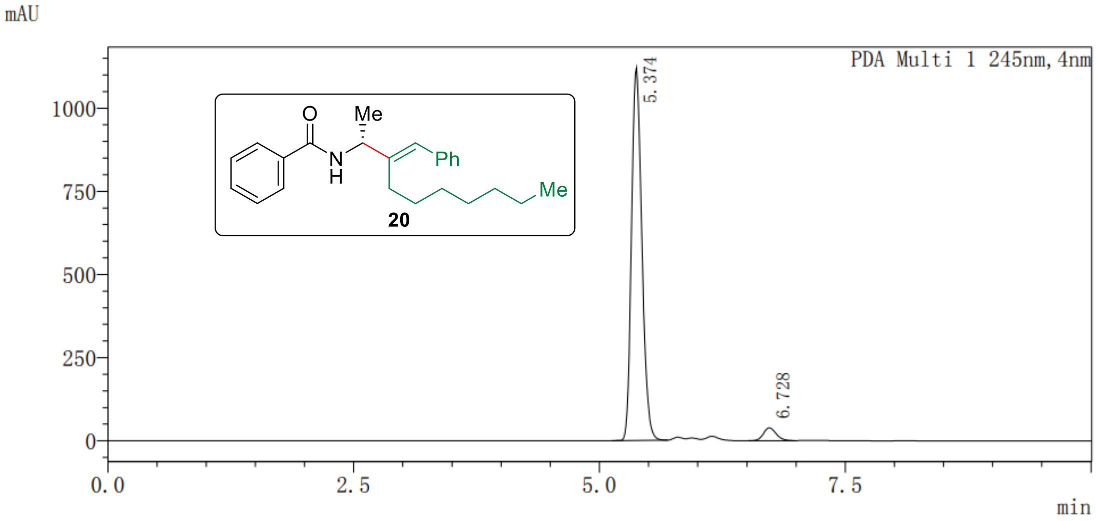

Peak#	Ret. Time	Area	Area%
1	5.374	8198289	95.957
2	6.728	345406	4.043
Total		8543695	100

Supplementary Figure 166. Chiral HPLC analysis of Compound **20**

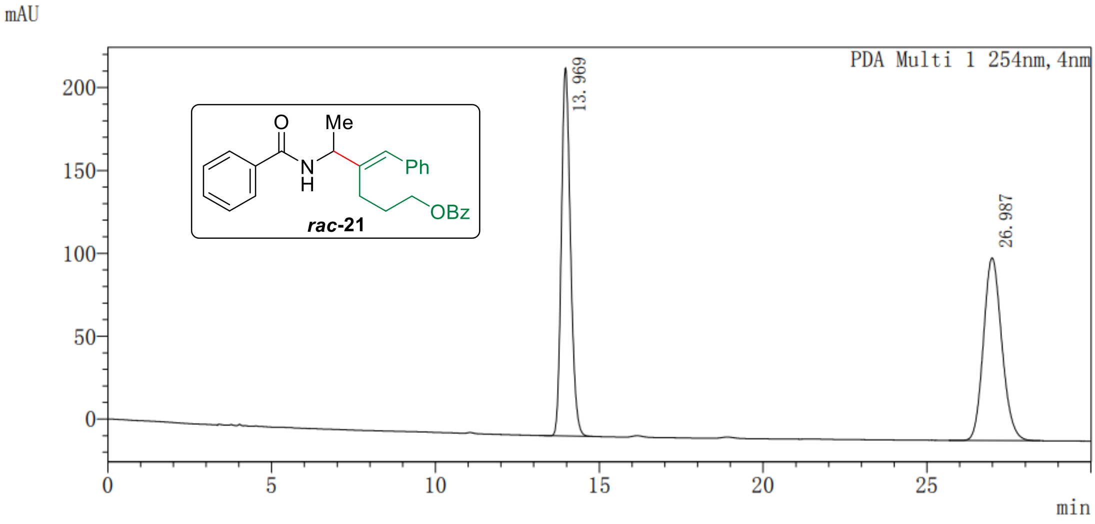

Peak#	Ret. Time	Area	Area%
1	13.969	4304942	50.008
2	26.987	4303516	49.992
Total		8608458	100

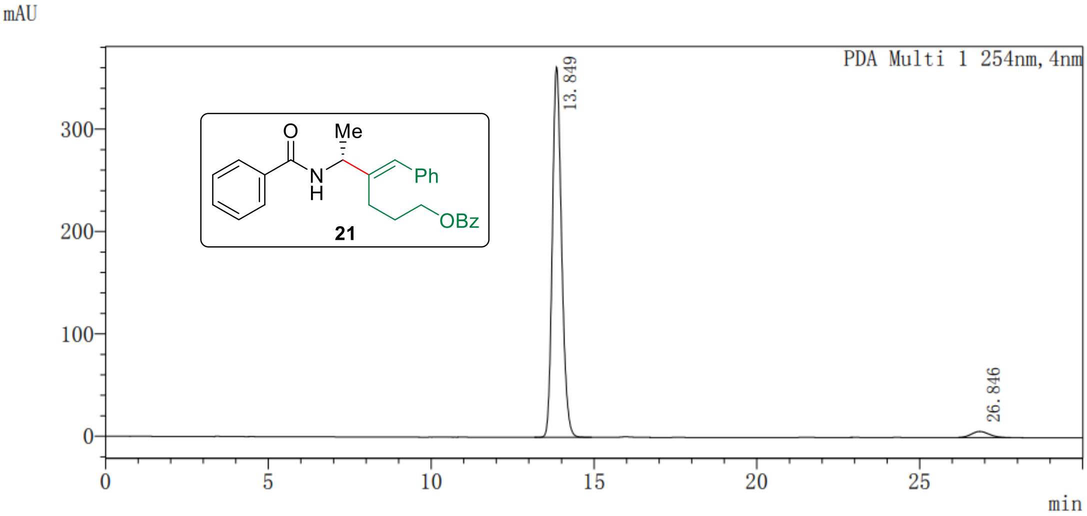

Peak#	Ret. Time	Area	Area%
1	13.849	7122956	96.965
2	26.846	222964	3.035
Total		7345920	100

Supplementary Figure 167. Chiral HPLC analysis of Compound **21**

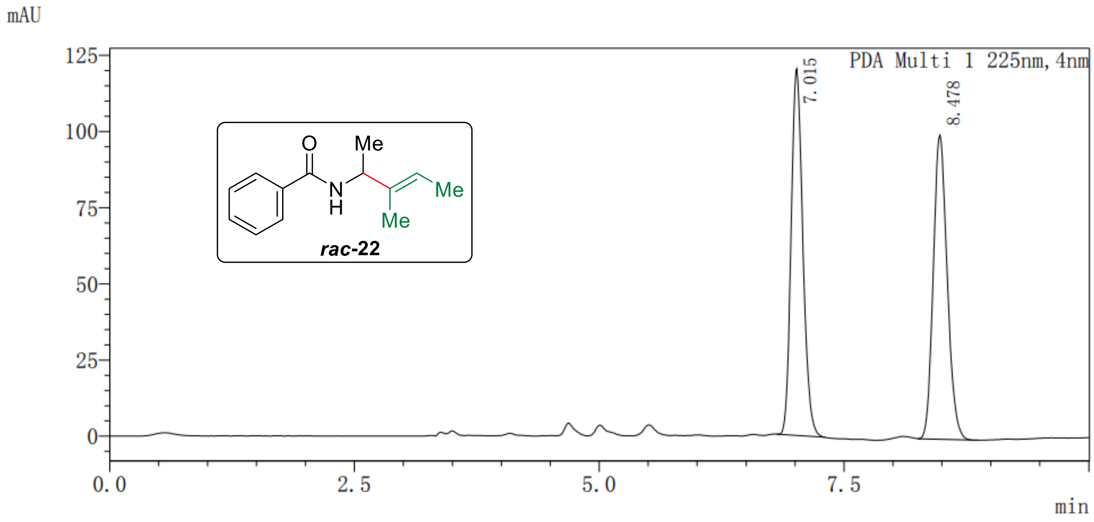

Peak#	Ret. Time	Area	Area%
1	7.015	998160	50.244
2	8.478	988460	49.756
Total		1986620	100

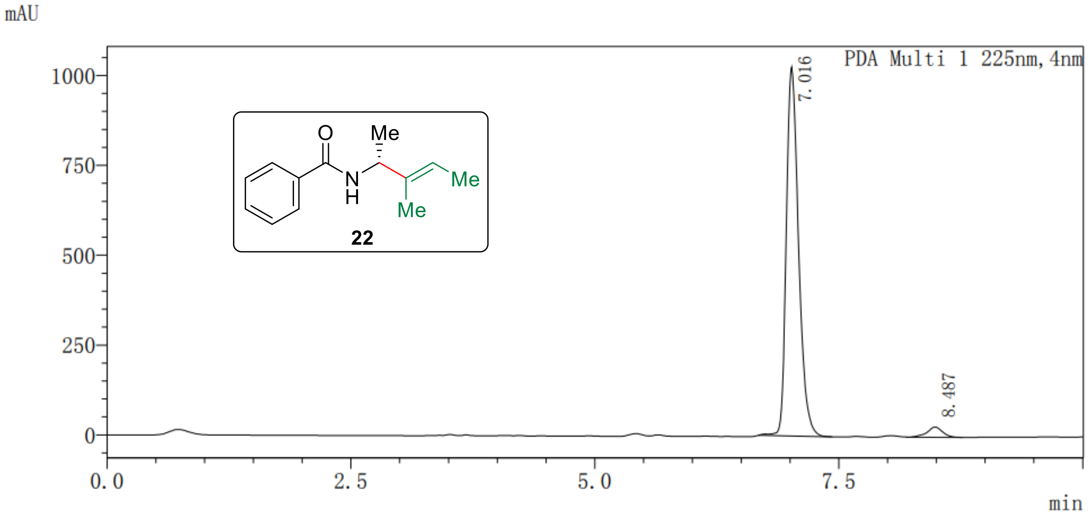

Peak#	Ret. Time	Area	Area%
1	7.016	8958234	96.680
2	8.487	307643	3.320
Total		9265877	100

Supplementary Figure 168. Chiral HPLC analysis of Compound **22**

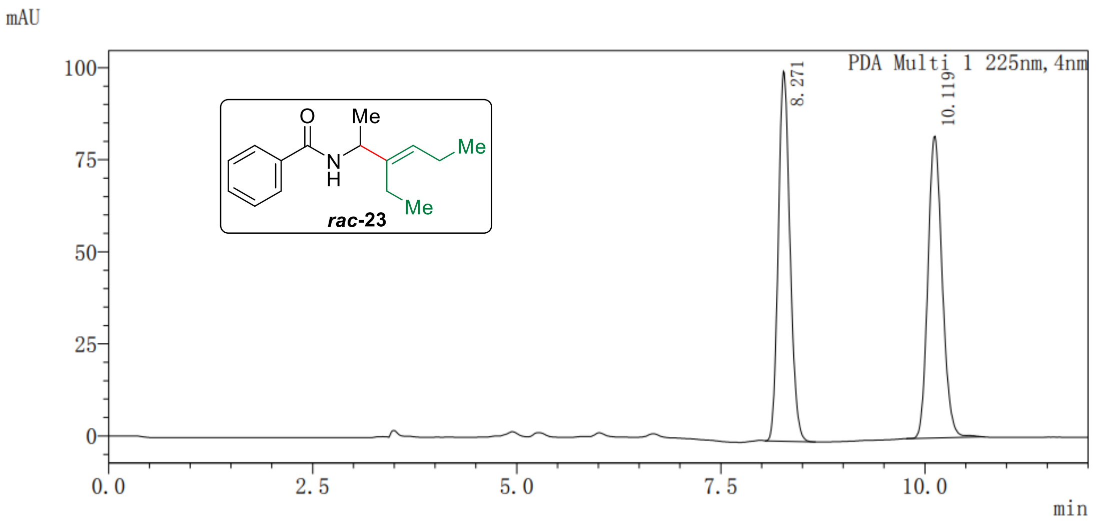

Peak#	Ret. Time	Area	Area%
1	8.271	998070	50.069
2	10.119	995322	49.931
Total		1993392	100

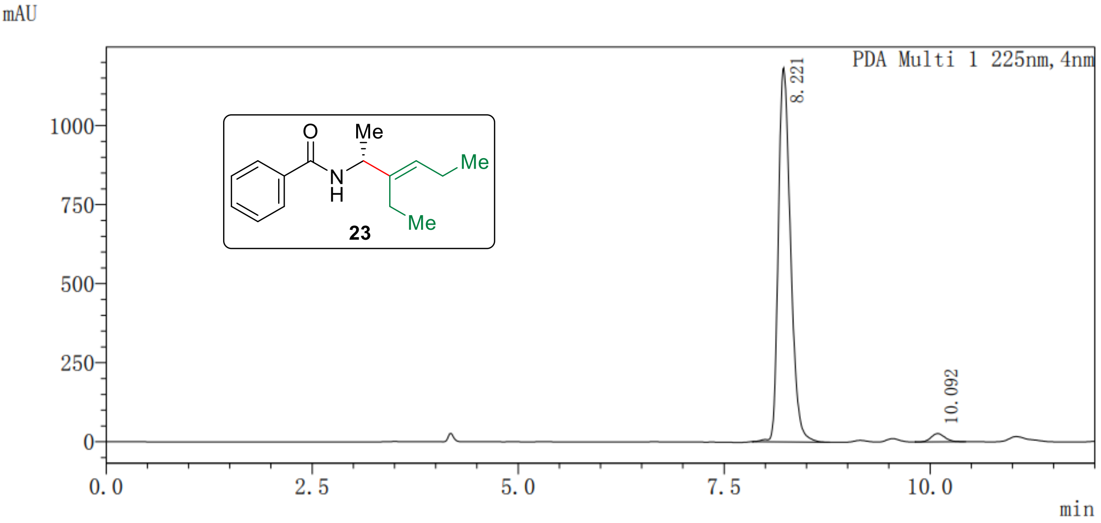

Peak#	Ret. Time	Area	Area%
1	8.221	12169024	97.569
2	10.092	303258	2.431
Total		12472282	100

Supplementary Figure 169. Chiral HPLC analysis of Compound **23**

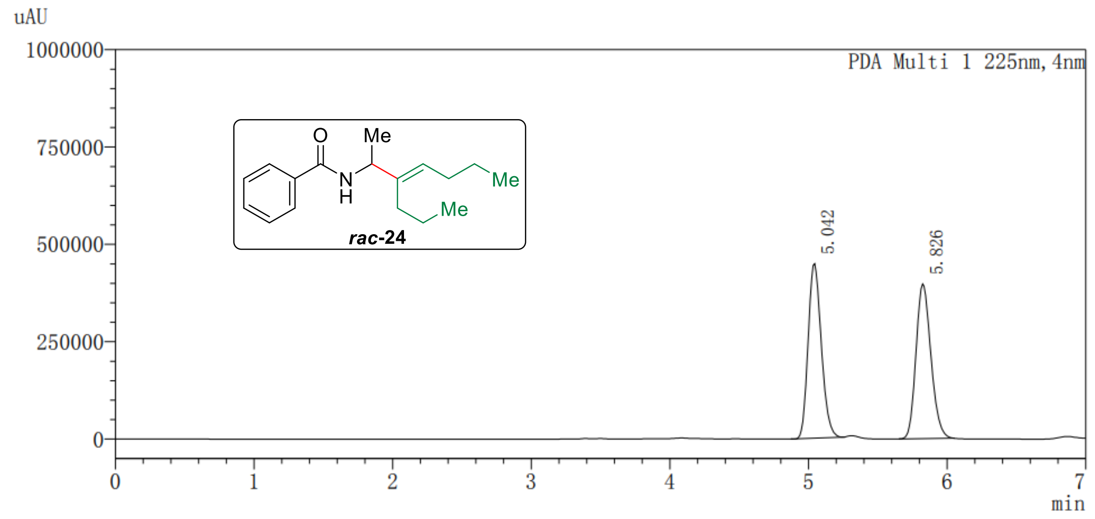

Peak#	Ret. Time	Area	Area%
1	5.042	2958725	49.956
2	5.826	2963954	50.044
Total		5922679	100

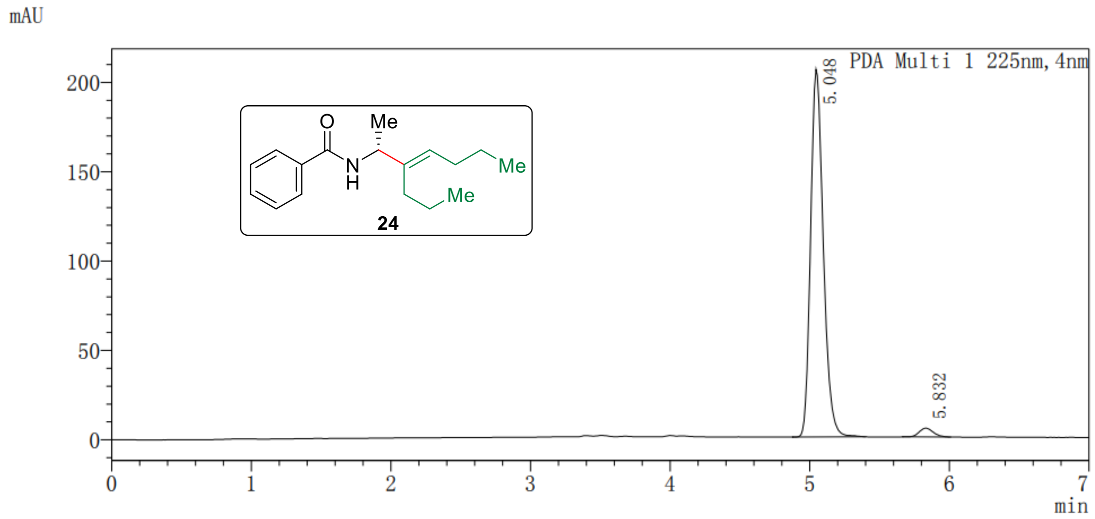

Peak#	Ret. Time	Area	Area%
1	5.048	1297737	97.420
2	5.832	34364	2.580
Total		1332101	100

Supplementary Figure 170. Chiral HPLC analysis of Compound **24**

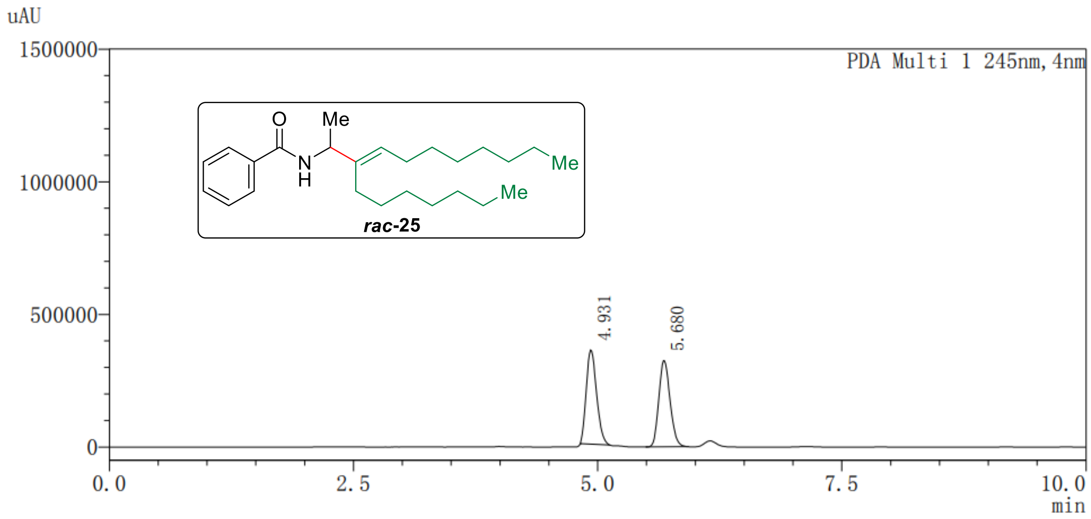

Peak#	Ret. Time	Area	Area%
1	4.931	2617759	50.114
2	5.680	2605867	49.886
Total		5223627	100

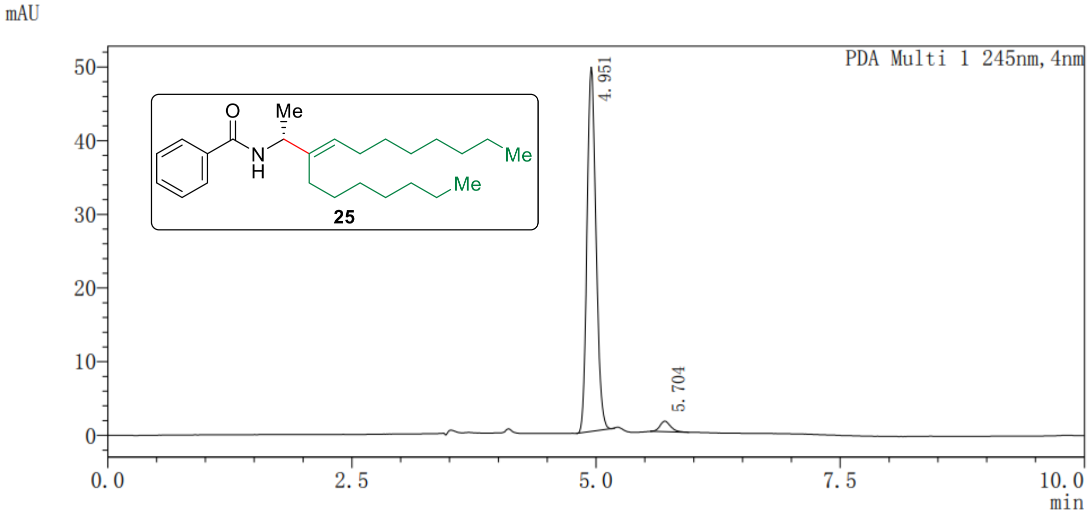

Peak#	Ret. Time	Area	Area%
1	4.951	316596	96.700
2	5.704	10805	3.300
Total		327401	100

Supplementary Figure 171. Chiral HPLC analysis of Compound **25**

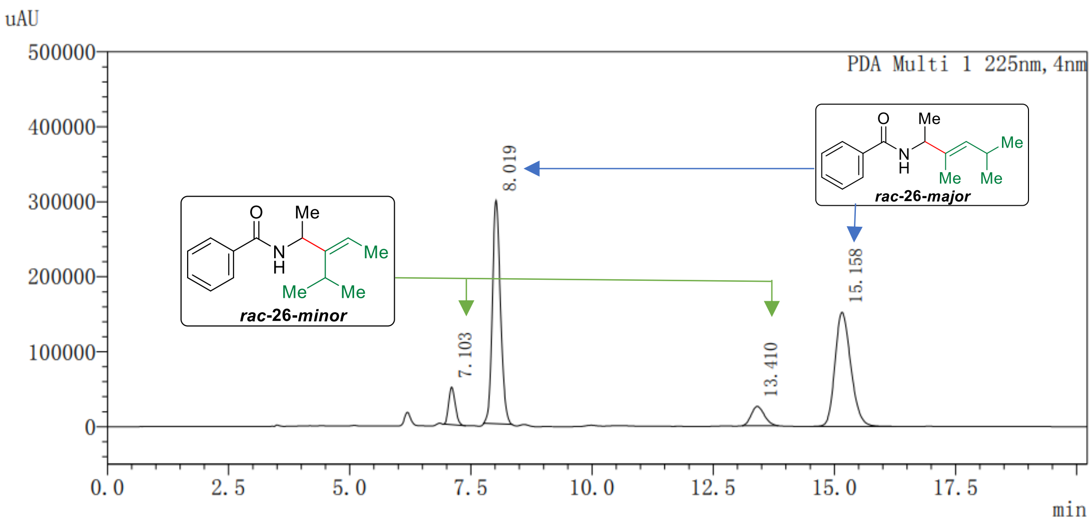

Peak#	Ret. Time	Area	Area%
1	7.103	473245	6.009
2	8.019	3462816	43.970
3	13.410	478867	6.081
4	15.158	3460461	43.940
Total		7875388	100

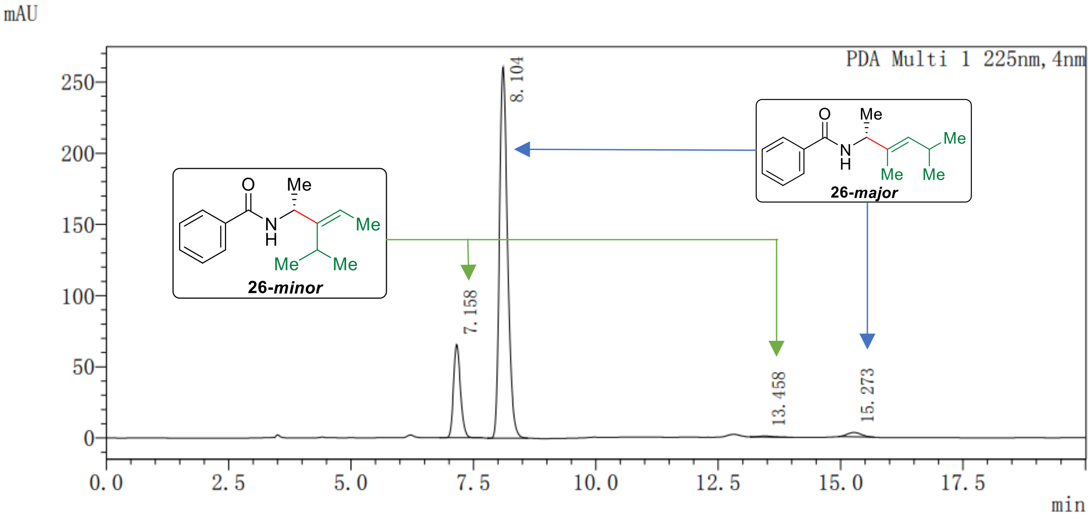

Peak#	Ret. Time	Area	Area%
1	7.158	643376	17.326
2	8.104	2993840	80.624
3	13.458	13966	0.376
4	15.273	62156	1.674
Total		3713338	100

Supplementary Figure 172. Chiral HPLC analysis of Compound **26**

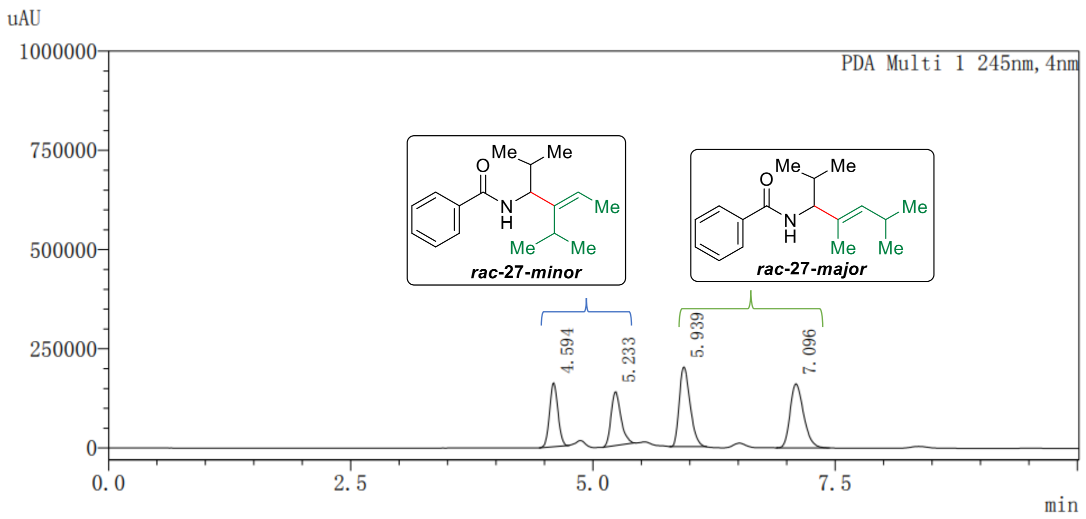

Peak#	Ret. Time	Area	Area%
1	4.594	967975	19.069
2	5.233	966888	19.048
3	5.939	1571348	30.956
4	7.096	1569838	30.926
Total		5076048	100

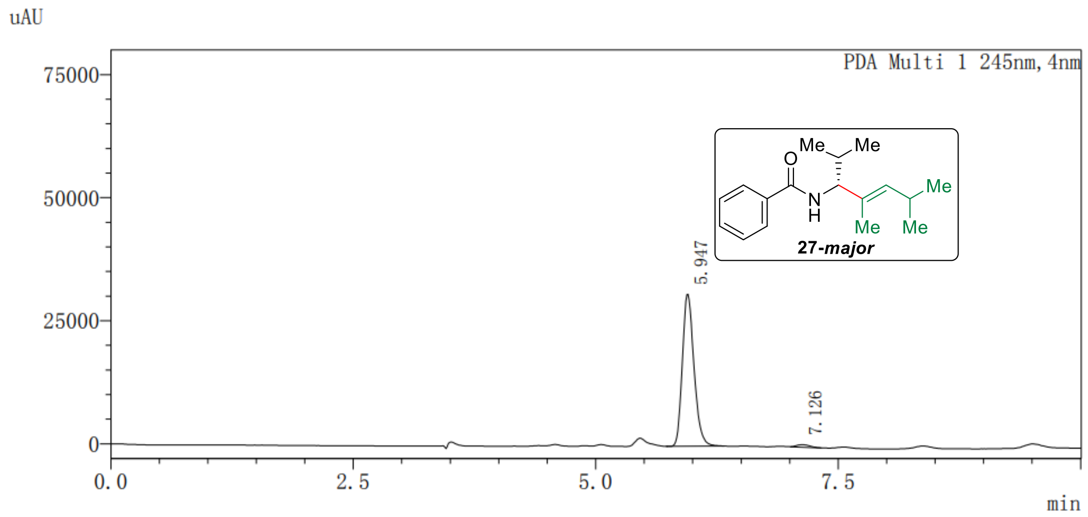

Peak#	Ret. Time	Area	Area%
1	5.947	258495	98.307
2	7.126	4452	1.693
Total		262947	100

Supplementary Figure 173. Chiral HPLC analysis of Compound **27**

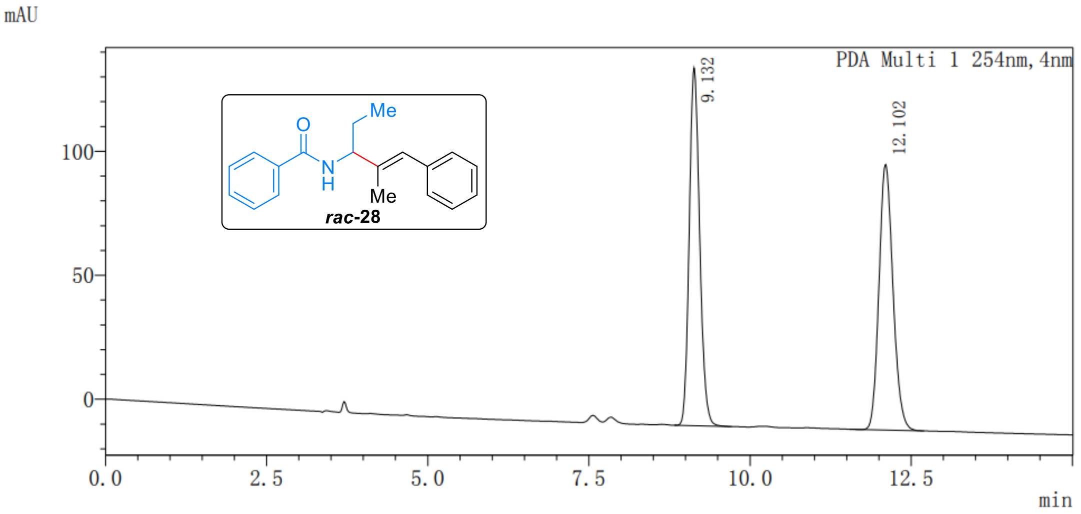

Peak#	Ret. Time	Area	Area%
1	9.132	1622001	50.074
2	12.102	1617191	49.926
Total		3239192	100

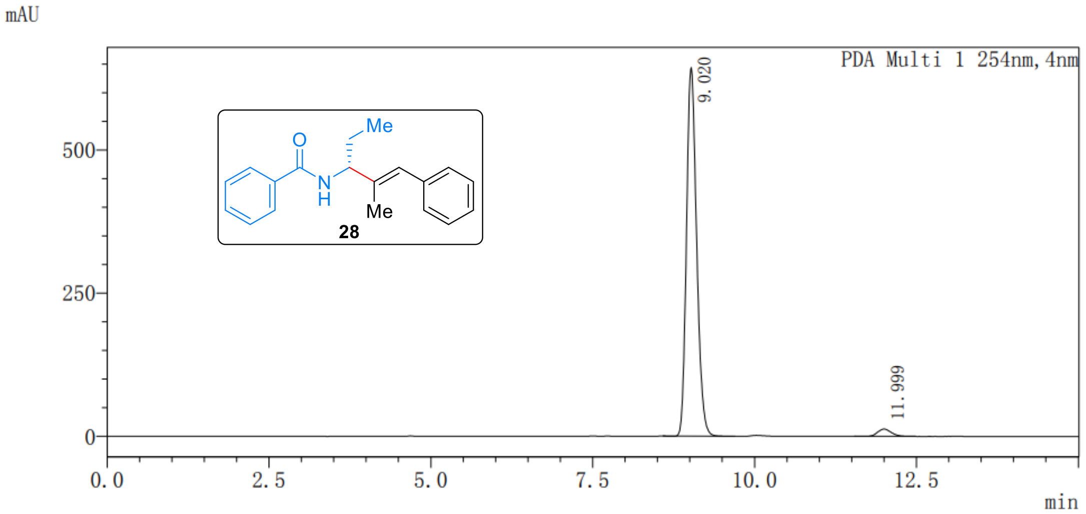

Peak#	Ret. Time	Area	Area%
1	9.020	7002754	97.340
2	11.999	191353	2.660
Total		7194107	100

Supplementary Figure 174. Chiral HPLC analysis of Compound **28**

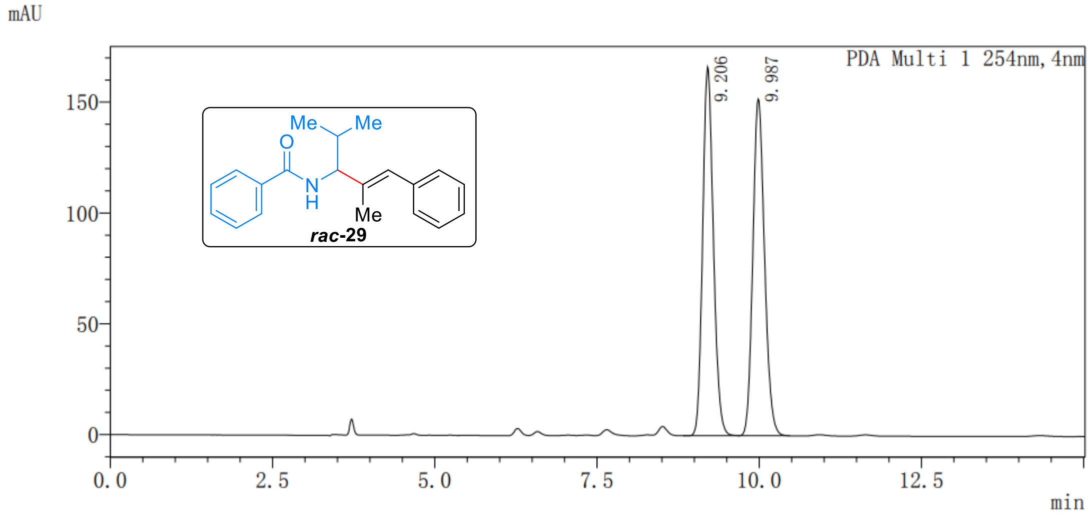

Peak#	Ret. Time	Area	Area%
1	9.206	1906948	50.039
2	9.987	1903964	49.961
Total		3810912	100

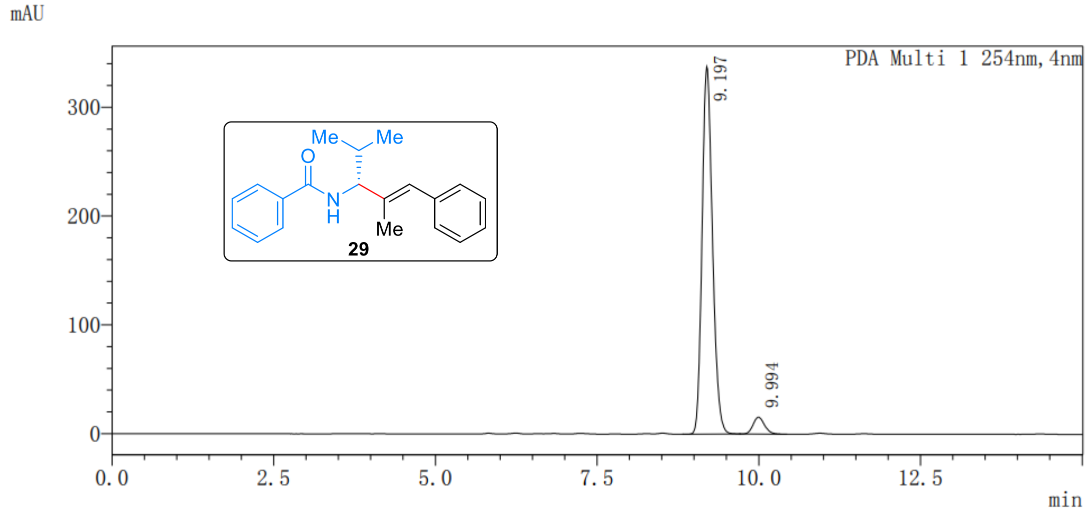

Peak#	Ret. Time	Area	Area%
1	9.197	3787615	95.180
2	9.994	191798	4.820
Total		3979413	100

Supplementary Figure 175. Chiral HPLC analysis of Compound **29**

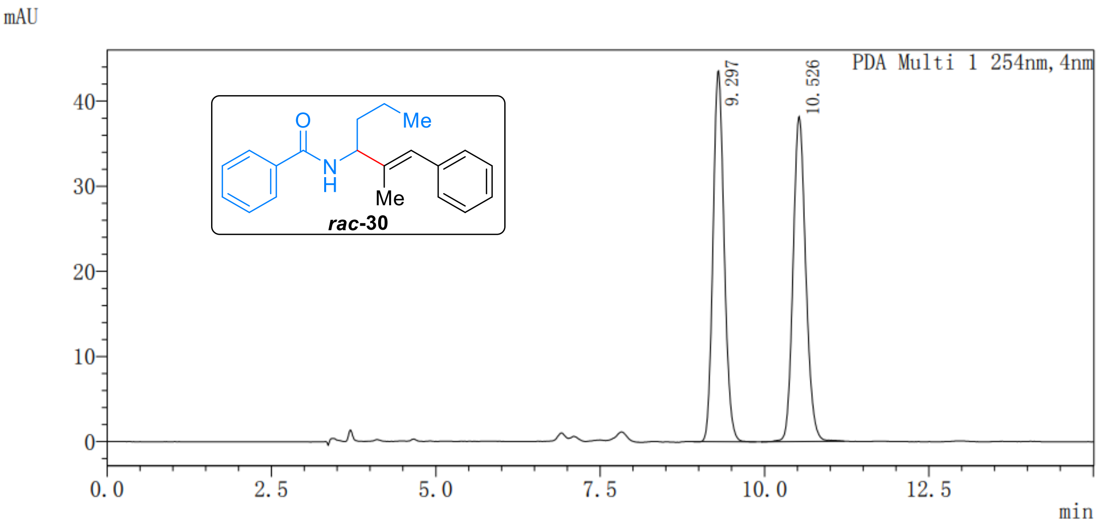

Peak#	Ret. Time	Area	Area%
1	9.297	511793	49.956
2	10.526	512687	50.044
Total		1024480	100

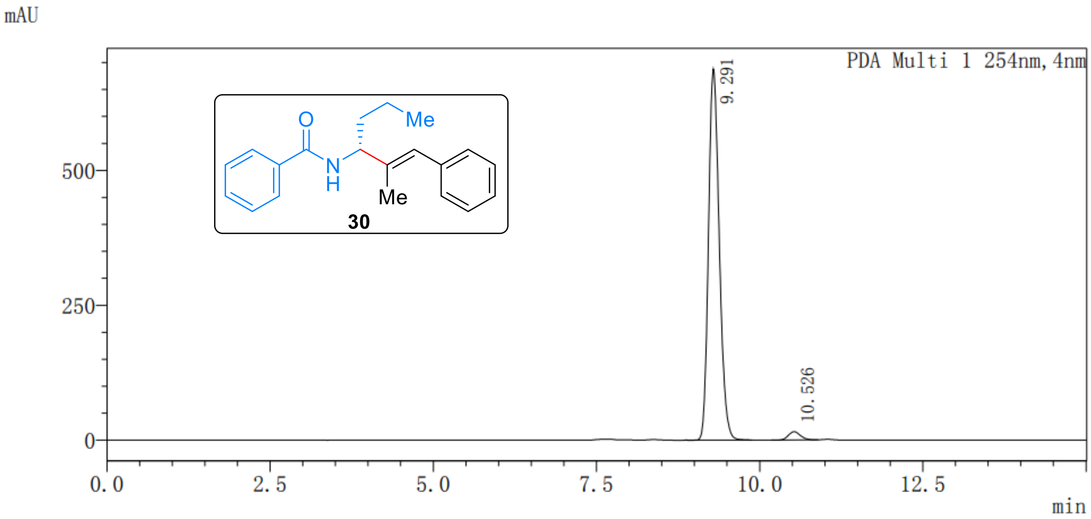

Peak#	Ret. Time	Area	Area%
1	9.291	7895537	97.559
2	10.526	197571	2.441
Total		8093107	100

Supplementary Figure 176. Chiral HPLC analysis of Compound **30**

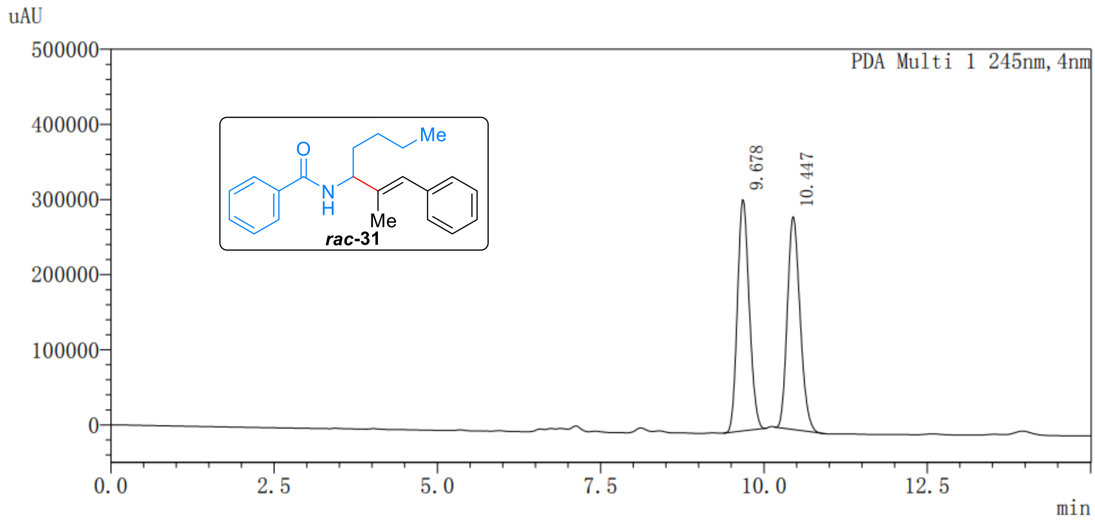

Peak#	Ret. Time	Area	Area%
1	9.678	3866362	50.144
2	10.447	3844124	49.856
Total		7710486	100

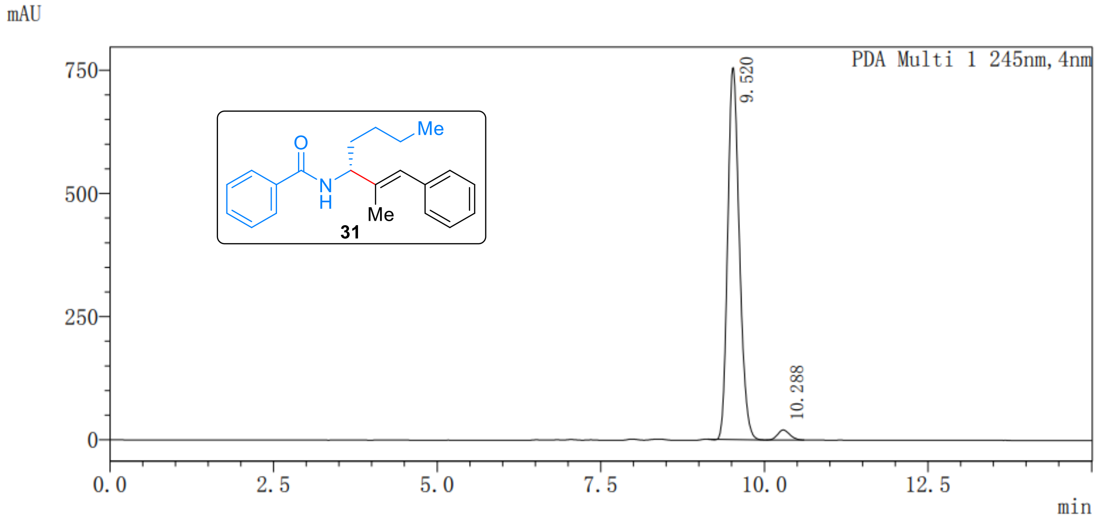

Peak#	Ret. Time	Area	Area%
1	9.520	9332477	97.271
2	10.288	261807	2.729
Total		9594284	100

Supplementary Figure 177. Chiral HPLC analysis of Compound **31**

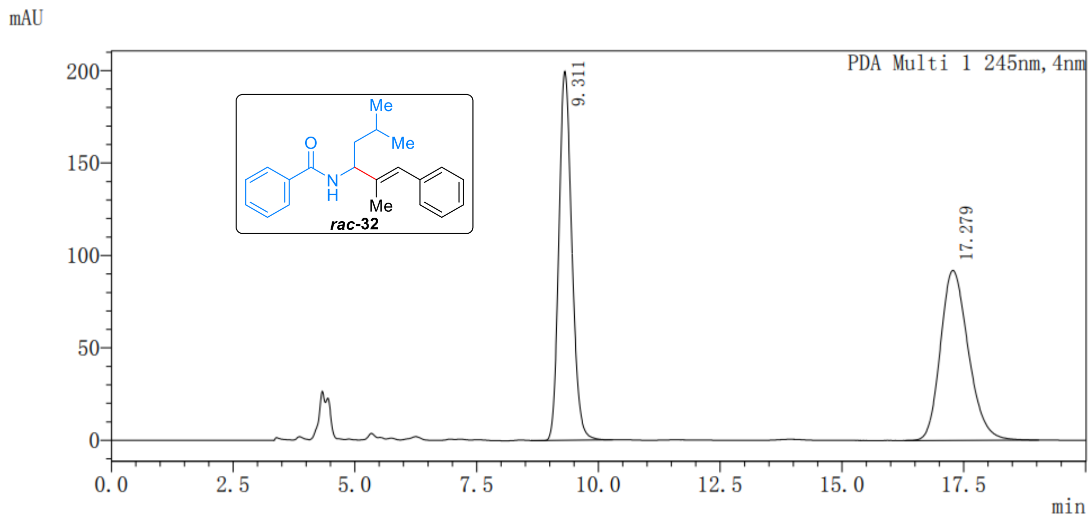

Peak#	Ret. Time	Area	Area%
1	9.311	3688314	50.186
2	17.279	3660972	49.814
Total		7349286	100

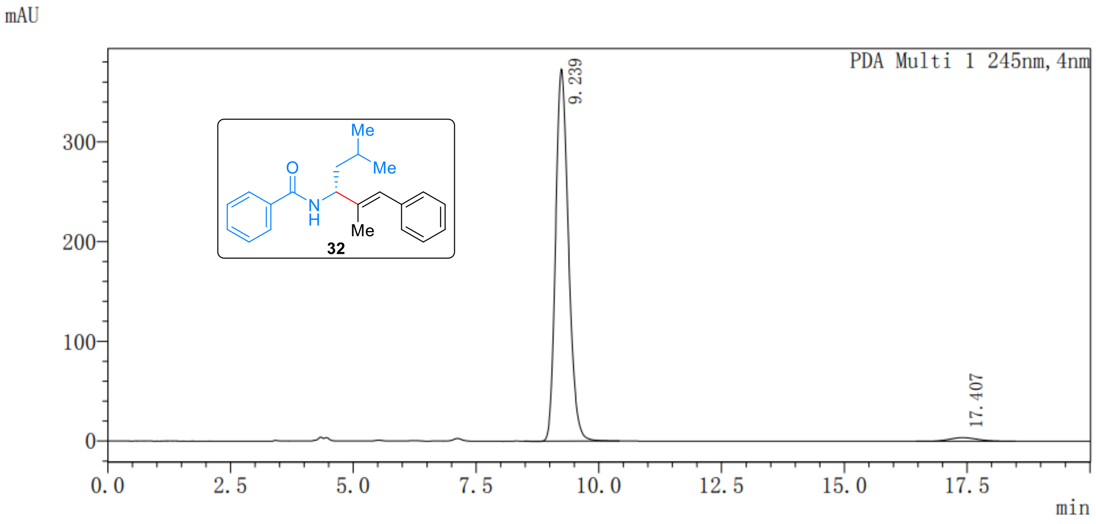

Peak#	Ret. Time	Area	Area%
1	9.239	6795848	97.846
2	17.407	149575	2.154
Total		6945422	100

Supplementary Figure 178. Chiral HPLC analysis of Compound **32**

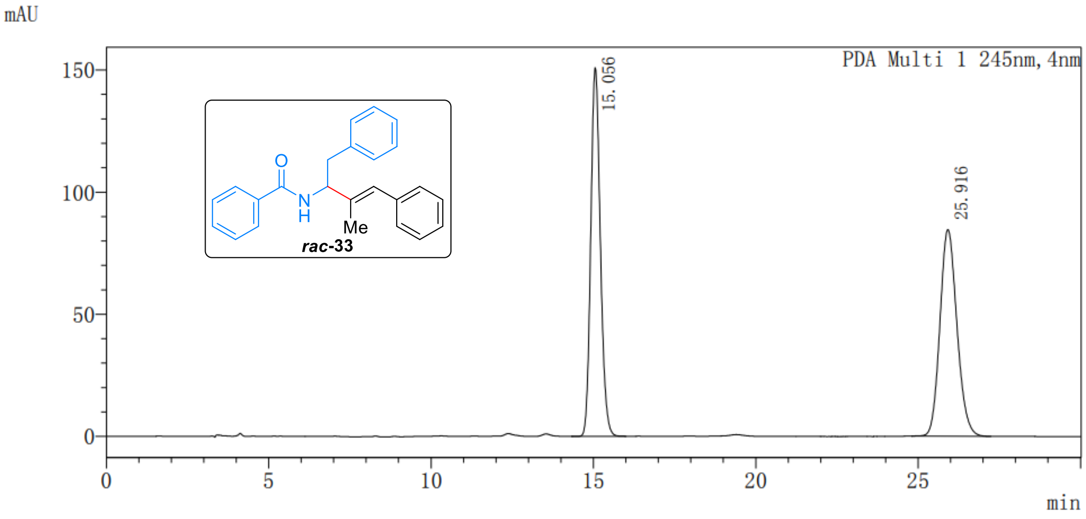

Peak#	Ret. Time	Area	Area%
1	15.056	3088761	50.050
2	25.916	3082583	49.950
Total		6171344	100

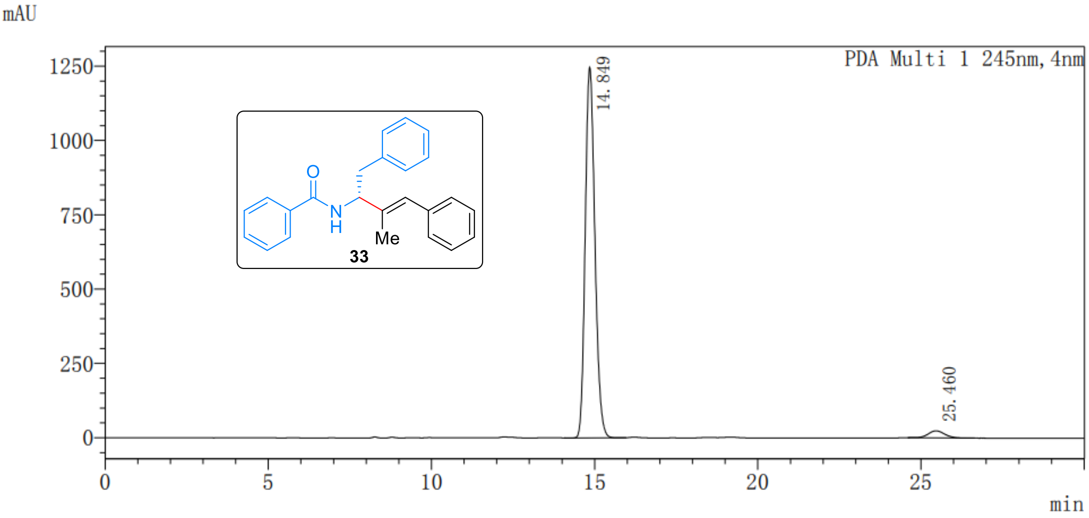

Peak#	Ret. Time	Area	Area%
1	14.849	25665675	96.869
2	25.460	829622	3.131
Total		26495297	100

Supplementary Figure 179. Chiral HPLC analysis of Compound **33**

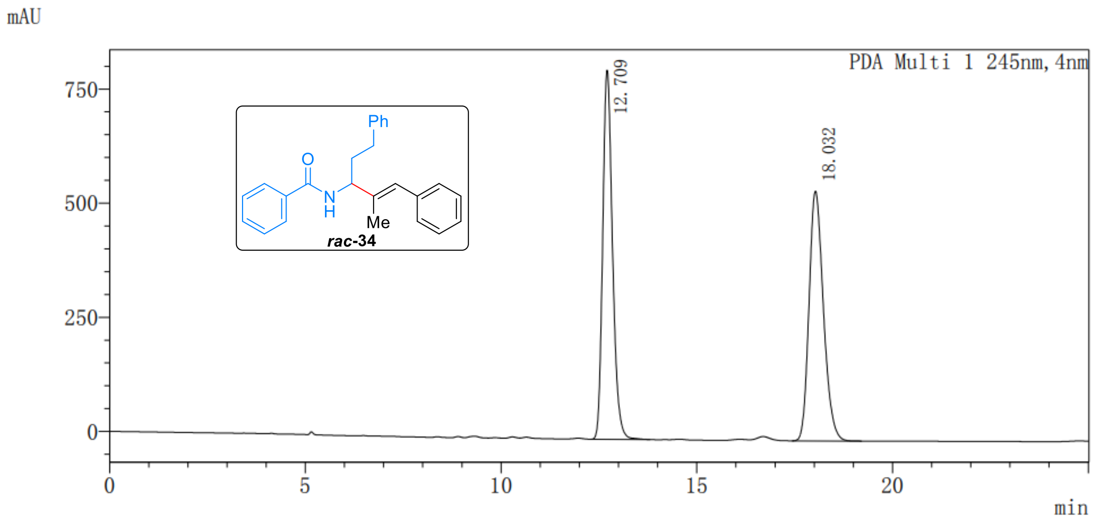

Peak#	Ret. Time	Area	Area%
1	12.709	14062329	50.161
2	18.032	13971961	49.839
Total		28034289	100

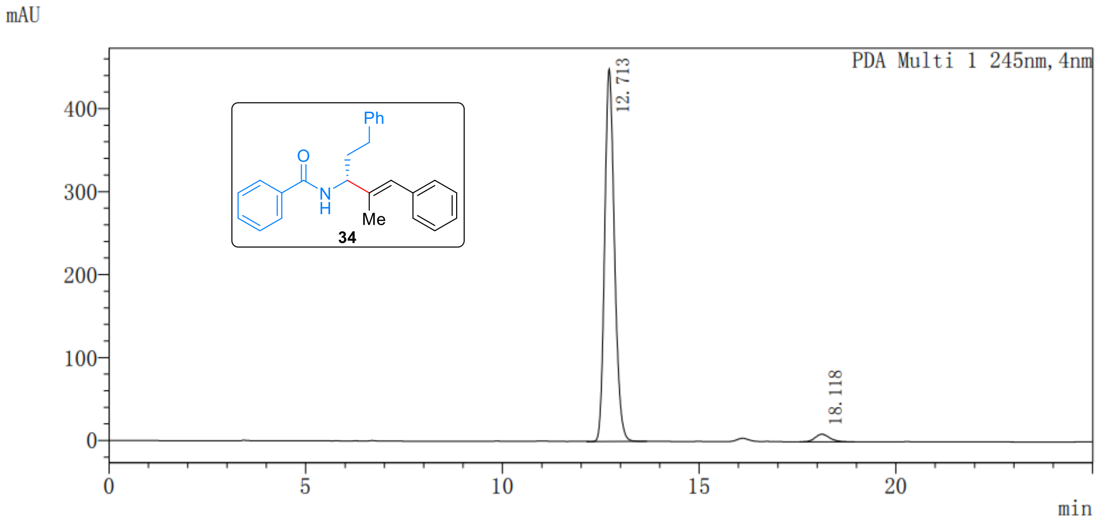

Peak#	Ret. Time	Area	Area%
1	12.713	7750791	97.086
2	18.118	232597	2.914
Total		7983388	100

Supplementary Figure 180. Chiral HPLC analysis of Compound **34**

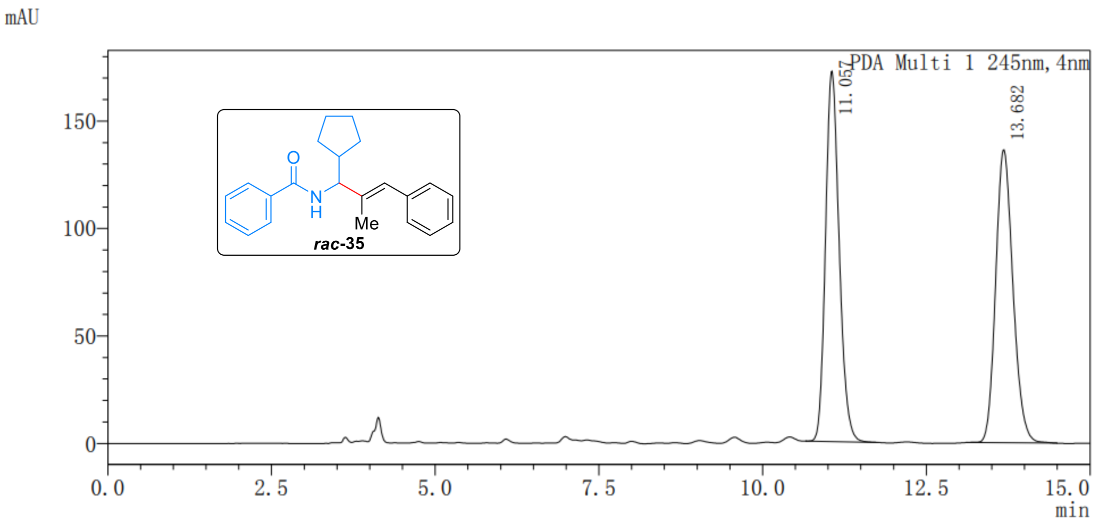

Peak#	Ret. Time	Area	Area%
1	11.057	2507163	49.969
2	13.682	2510235	50.031
Total		5017398	100

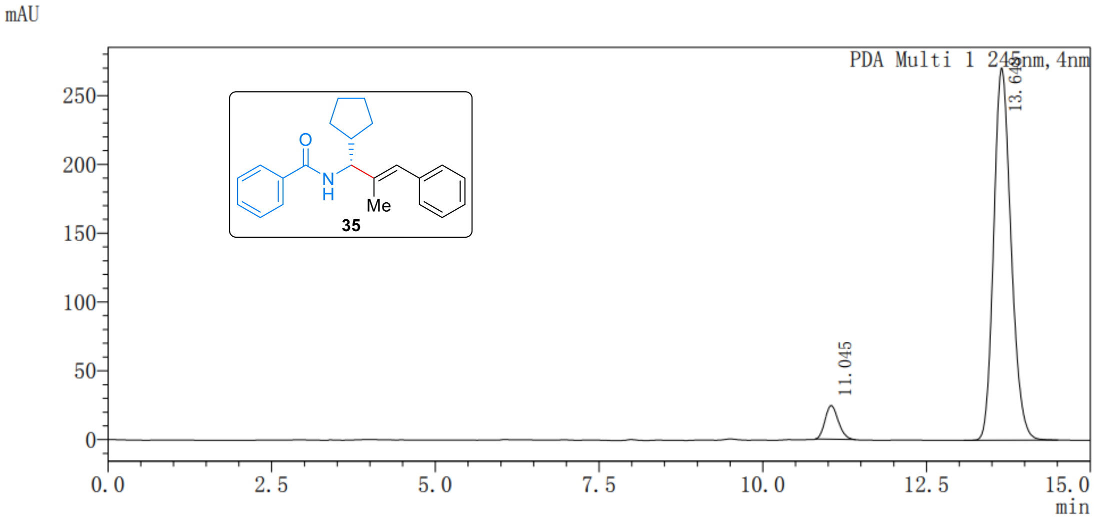

Peak#	Ret. Time	Area	Area%
1	11.045	346645	6.493
2	13.648	4992063	93.507
Total		5338708	100

Supplementary Figure 181. Chiral HPLC analysis of Compound **35**

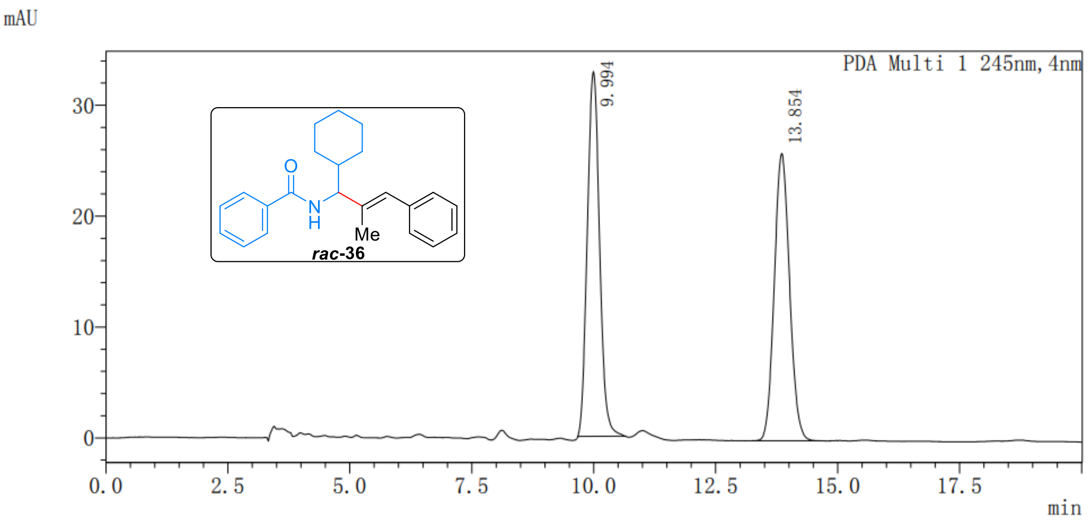

Peak#	Ret. Time	Area	Area%
1	9.994	572871	50.355
2	13.854	564786	49.645
Total		1137657	100

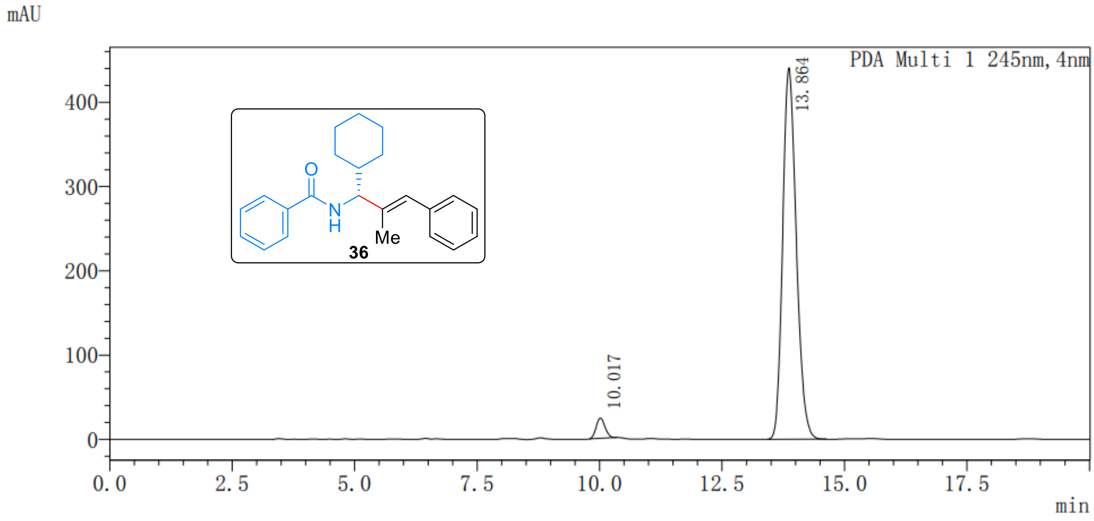

Peak#	Ret. Time	Area	Area%
1	10.017	300677	3.415
2	13.864	8504393	96.585
Total		8805071	100

Supplementary Figure 182. Chiral HPLC analysis of Compound **36**

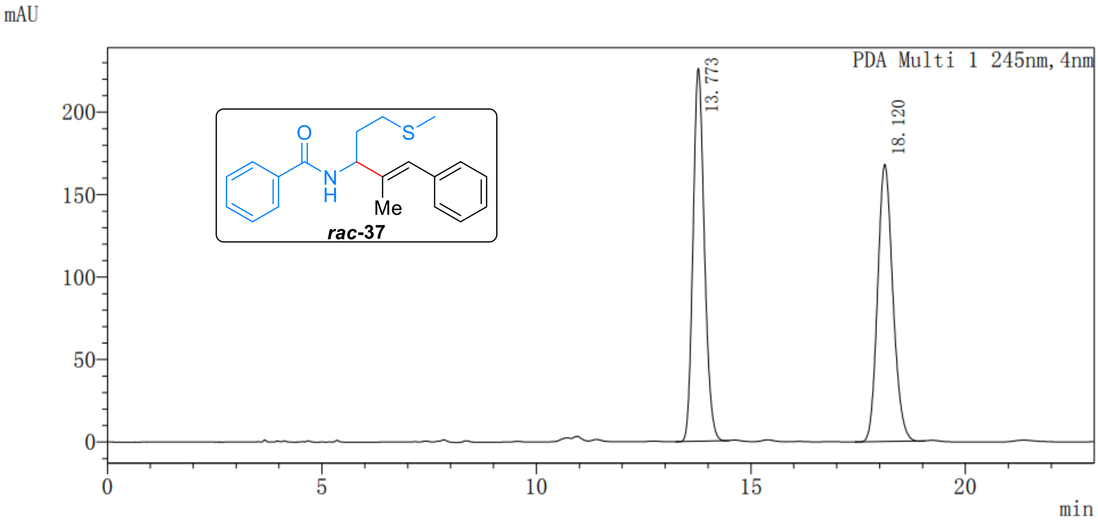

Peak#	Ret. Time	Area	Area%
1	13.773	4160213	50.009
2	18.120	4158638	49.991
Total		8318851	100

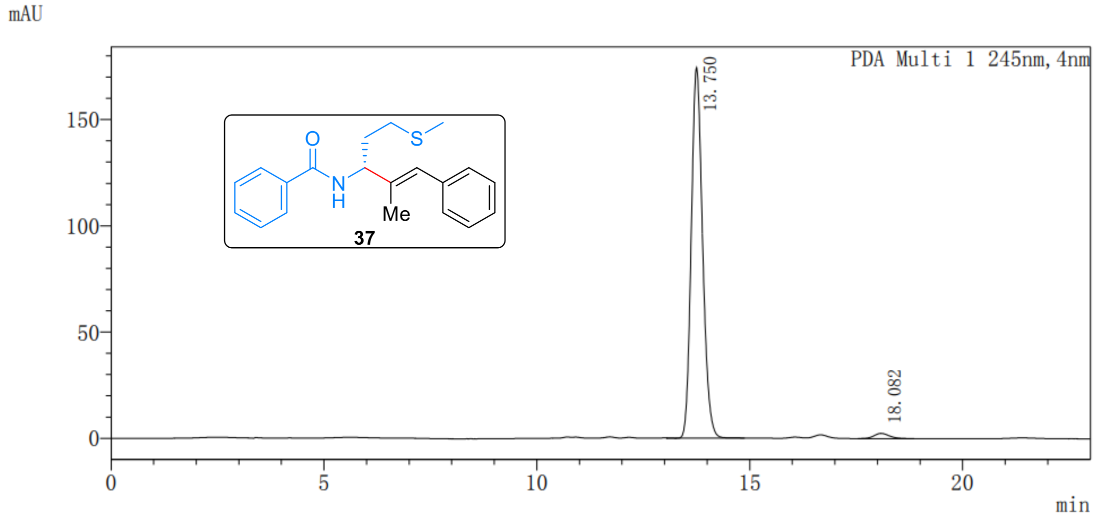

Peak#	Ret. Time	Area	Area%
1	13.750	3212271	98.138
2	18.082	60943	1.862
Total		3273214	100

Supplementary Figure 183. Chiral HPLC analysis of Compound **37**

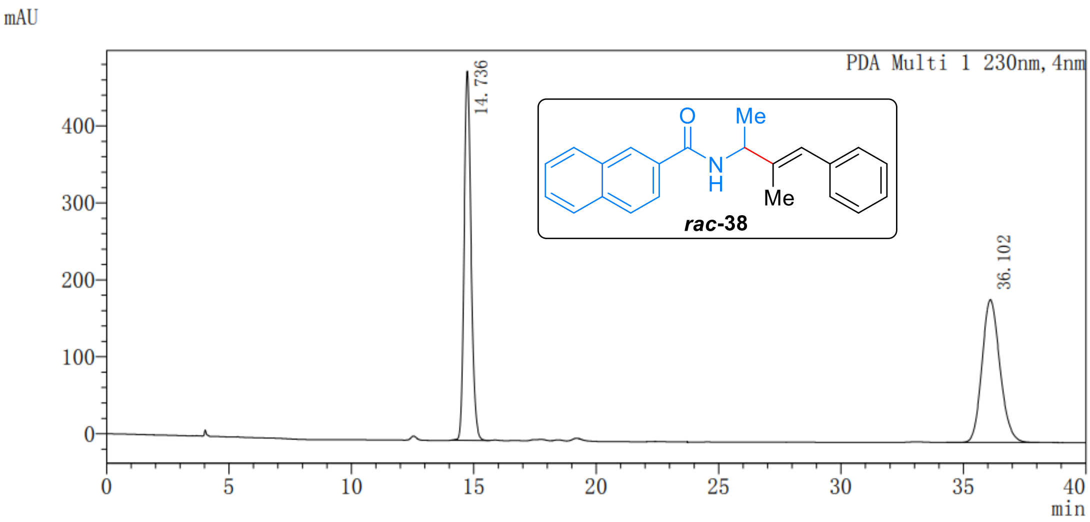

Peak#	Ret. Time	Area	Area%
1	14.736	9448151	49.924
2	36.102	9477030	50.076
Total		18925181	100

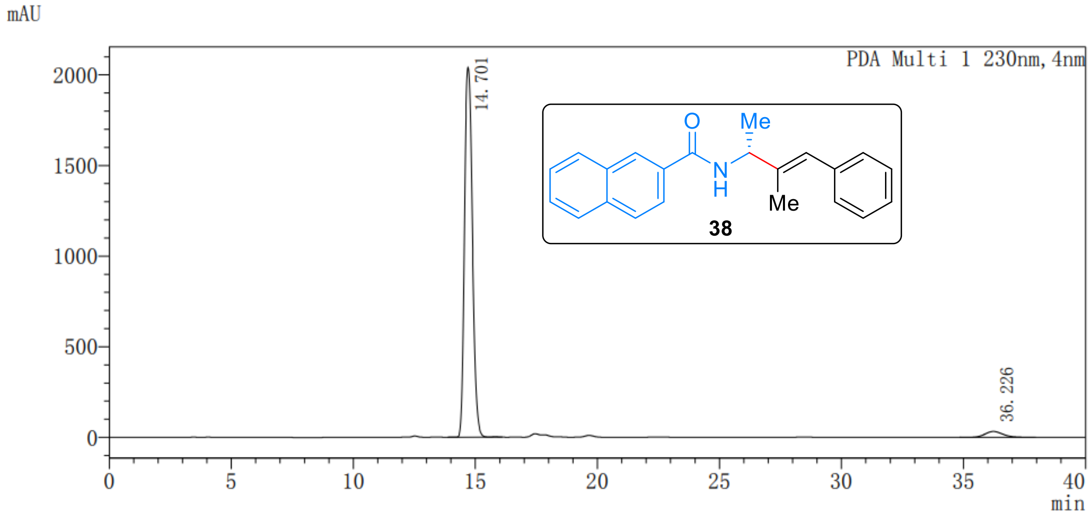

Peak#	Ret. Time	Area	Area%
1	14.701	45951178	96.481
2	36.226	1675804	3.519
Total		47626983	100

Supplementary Figure 184. Chiral HPLC analysis of Compound **38**

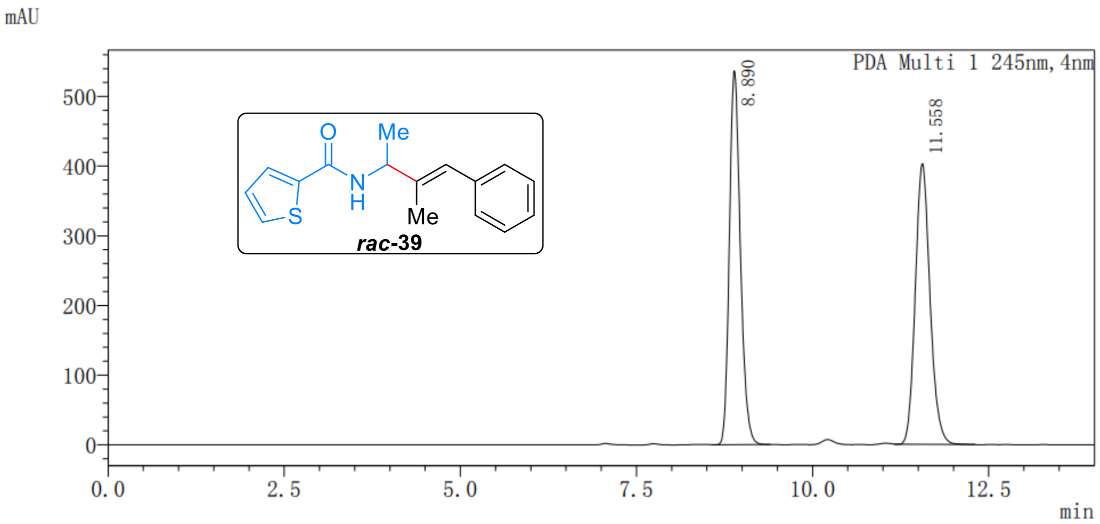

Peak#	Ret. Time	Area	Area%
1	8.890	5836785	49.917
2	11.558	5856223	50.083
Total		11693008	100

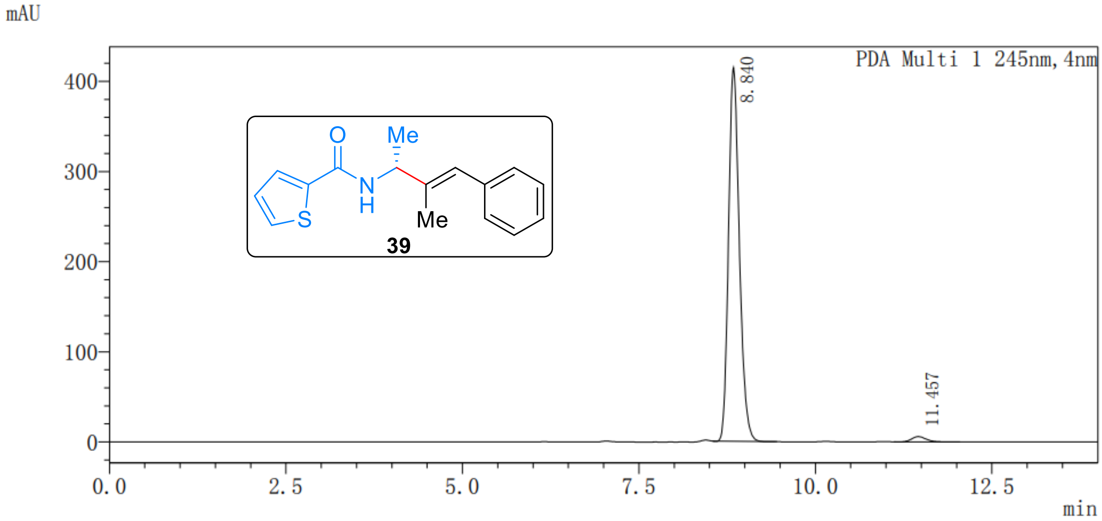

Peak#	Ret. Time	Area	Area%
1	8.840	4446841	98.155
2	11.457	83594	1.845
Total		4530435	100

Supplementary Figure 185. Chiral HPLC analysis of Compound **39**

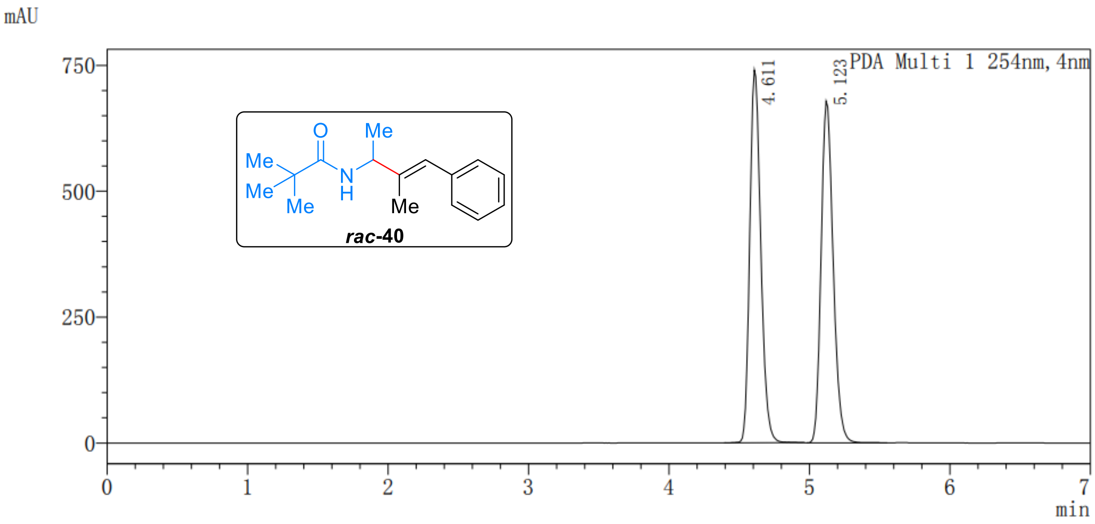

Peak#	Ret. Time	Area	Area%
1	4.611	4028980	49.904
2	5.123	4044410	50.096
Total		8073390	100

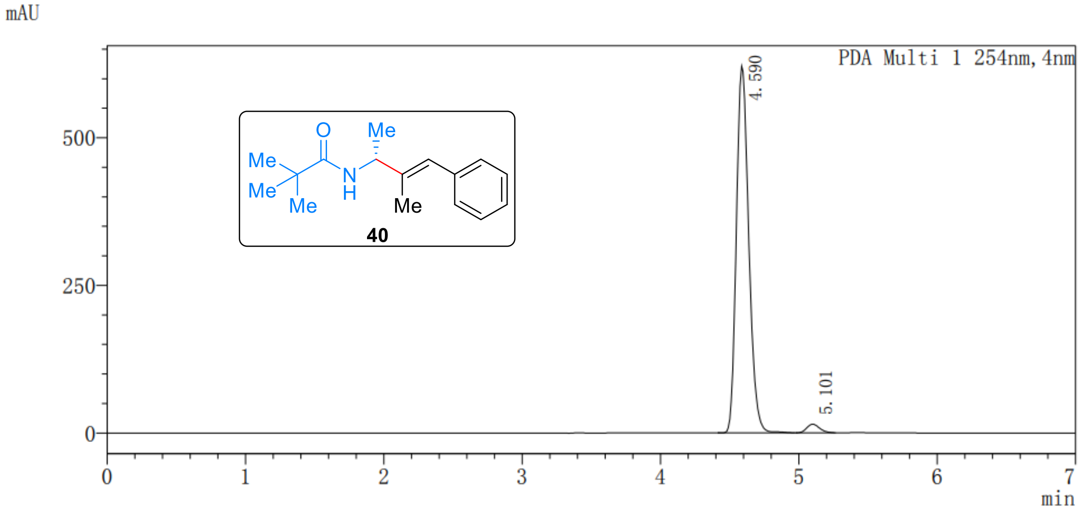

Peak#	Ret. Time	Area	Area%
1	4.590	3856435	97.629
2	5.101	93656	2.371
Total		3950091	100

Supplementary Figure 186. Chiral HPLC analysis of Compound **40**

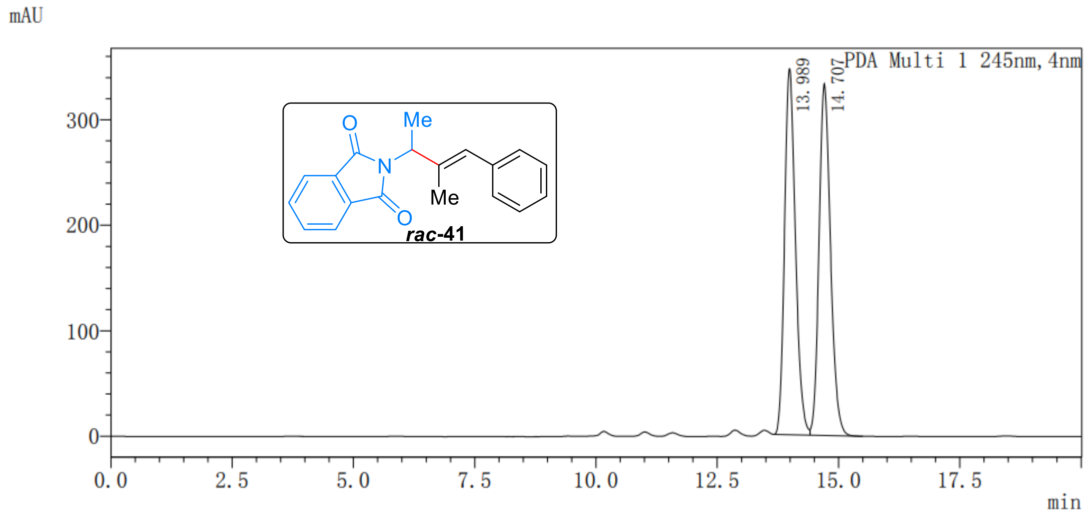

Peak#	Ret. Time	Area	Area%
1	13.989	5422244	49.721
2	14.707	5483088	50.279
Total		10905332	100

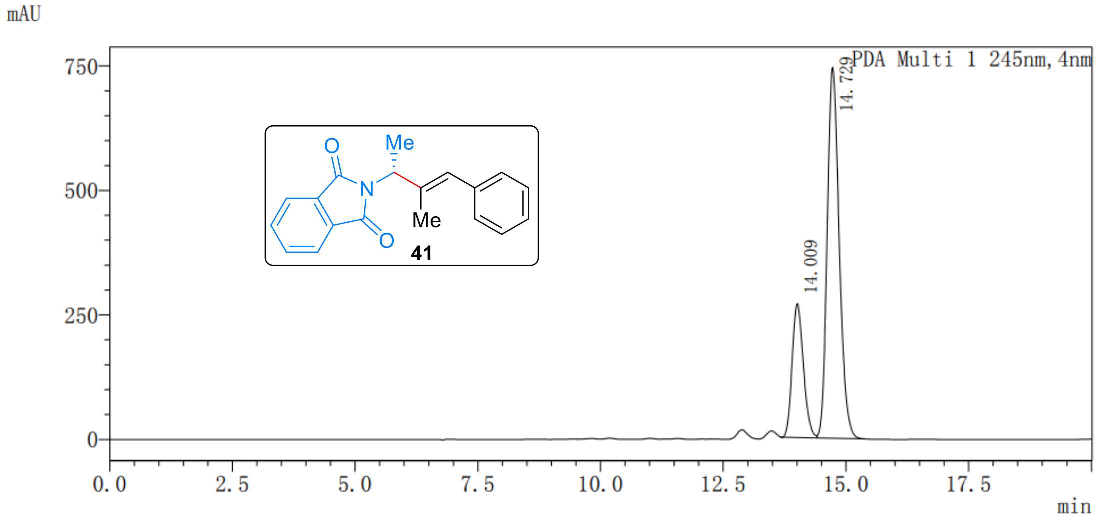

Peak#	Ret. Time	Area	Area%
1	14.009	4290516	25.327
2	14.729	12650216	74.673
Total		16940733	100

Supplementary Figure 187. Chiral HPLC analysis of Compound **41**

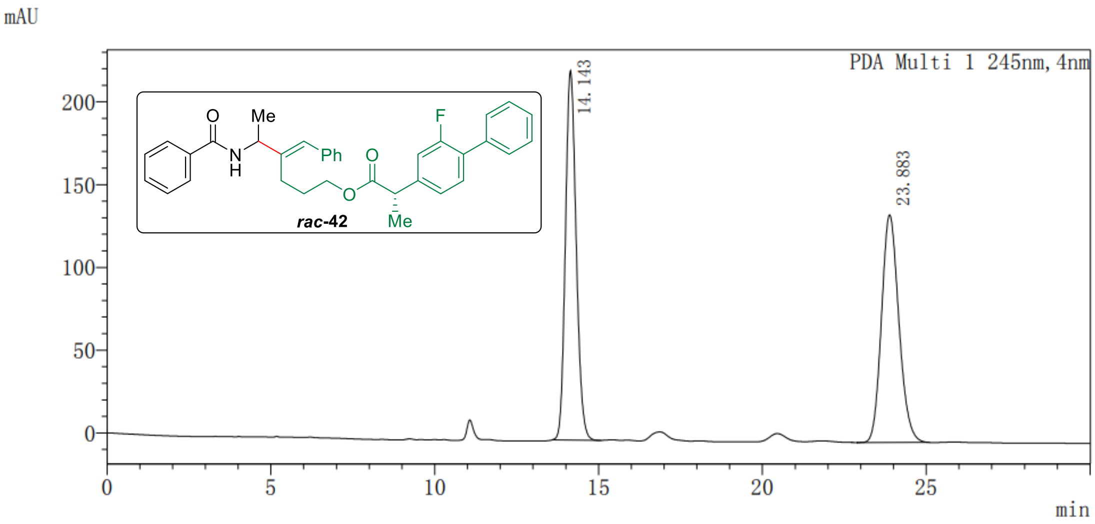

Peak#	Ret. Time	Area	Area%
1	14.143	5087773	50.074
2	23.883	5072739	49.926
Total		10160512	100

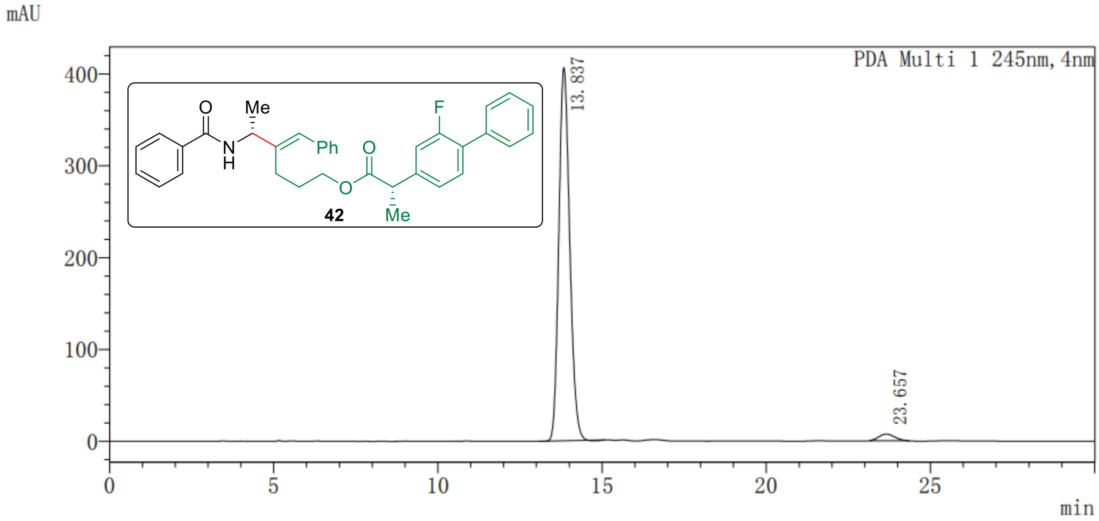

Peak#	Ret. Time	Area	Area%
1	13.837	9333364	97.489
2	23.657	240368	2.511
Total		9573731	100

Supplementary Figure 188. Chiral HPLC analysis of Compound **42**

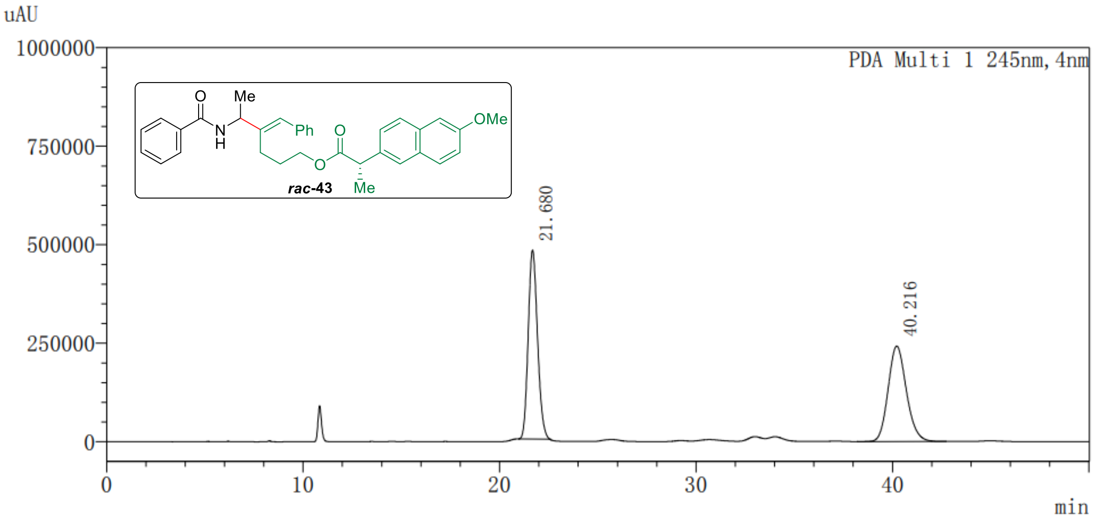

Peak#	Ret. Time	Area	Area%
1	21.680	15815845	50.247
2	40.216	15660332	49.753
Total		31476177	100

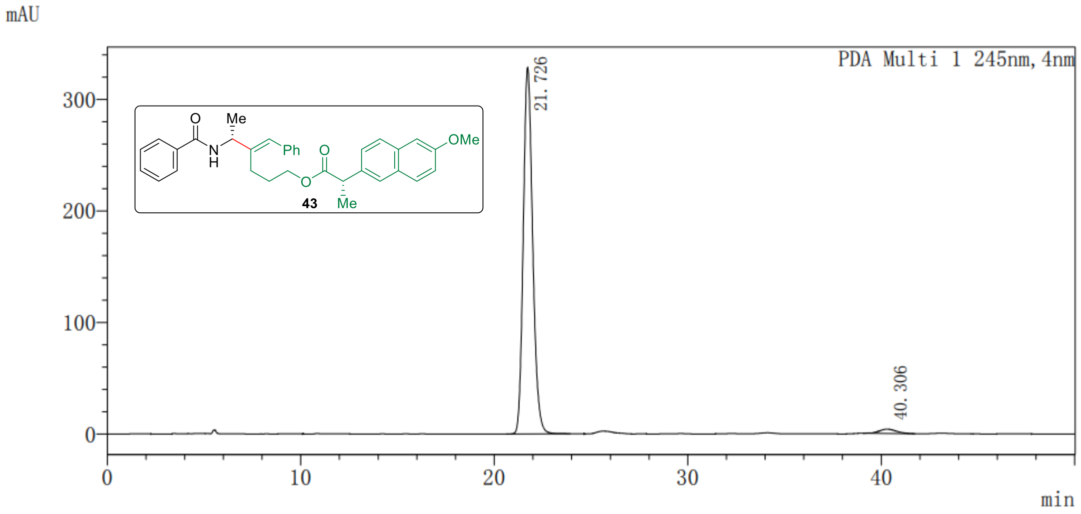

Peak#	Ret. Time	Area	Area%
1	21.726	11104483	97.964
2	40.306	230757	2.036
Total		11335240	100

Supplementary Figure 189. Chiral HPLC analysis of Compound **43**

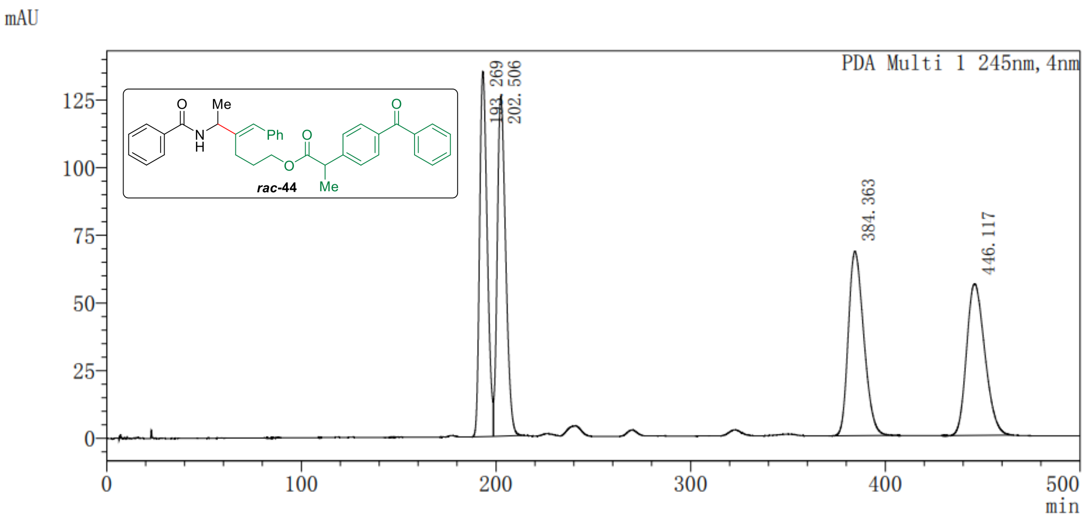

Peak#	Ret. Time	Area	Area%
1	193.269	36656515	24.640
2	202.506	37790766	25.402
3	384.363	37587066	25.265
4	446.117	36734404	24.692
Total		148768752	100

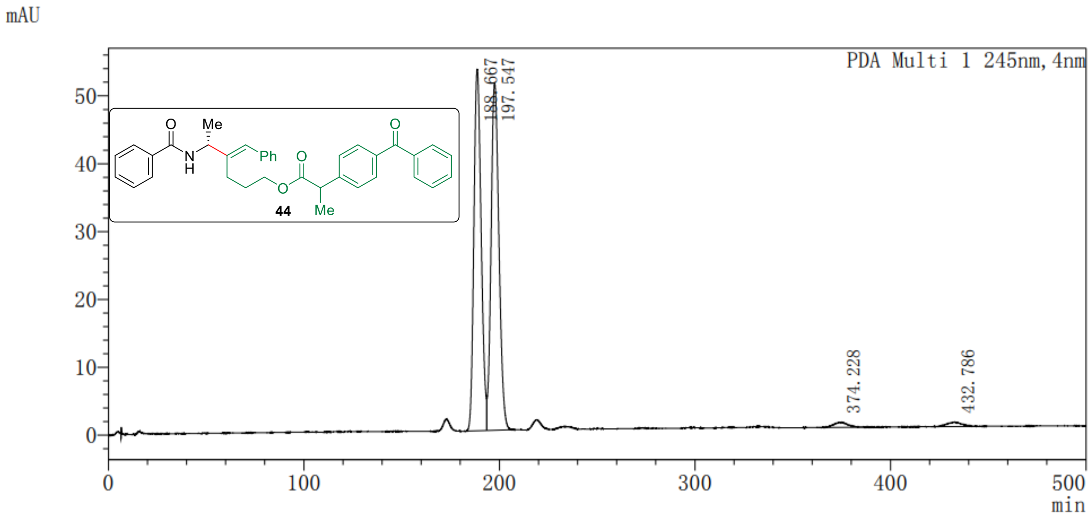

Peak#	Ret. Time	Area	Area%
1	188.667	13805276	47.889
2	197.547	14287662	49.562
3	374.228	370706	1.286
4	432.786	364079	1.263
Total		28827722	100

Supplementary Figure 190. Chiral HPLC analysis of Compound **44**

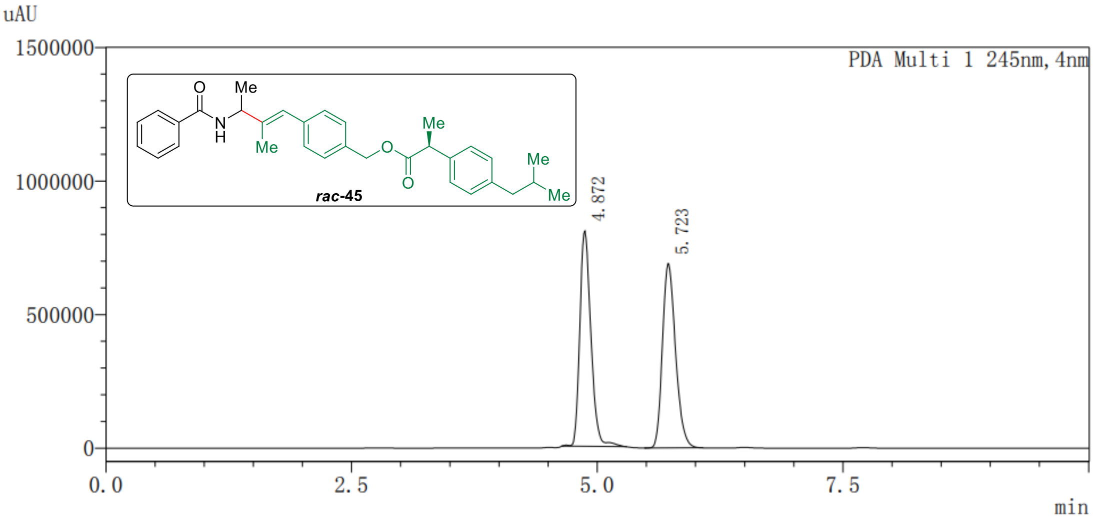

Peak#	Ret. Time	Area	Area%
1	4.872	6356577	49.821
2	5.723	6402164	50.179
Total		12758741	100

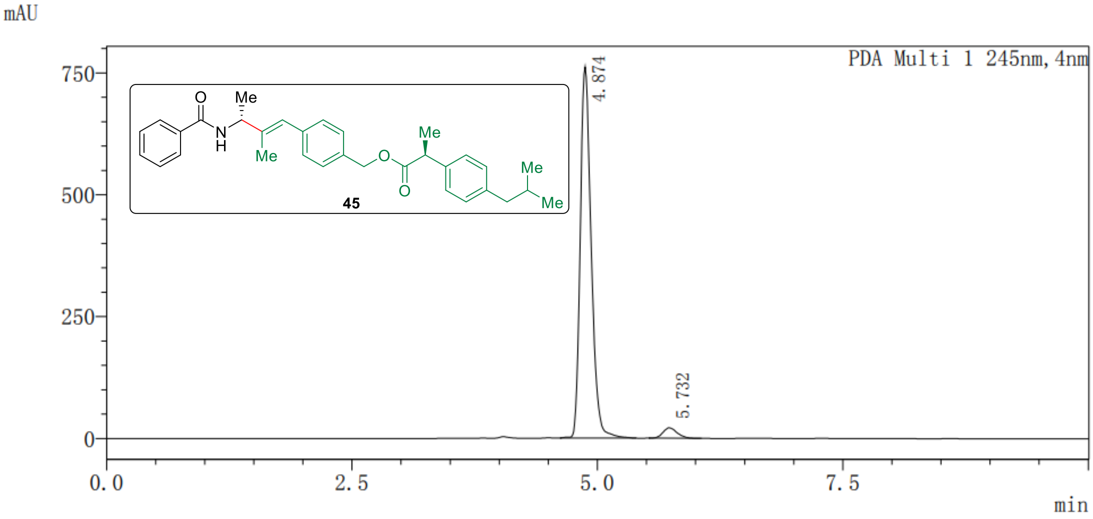

Peak#	Ret. Time	Area	Area%
1	4.874	5898320	96.492
2	5.732	214466	3.508
Total		6112785	100

Supplementary Figure 191. Chiral HPLC analysis of Compound **45**

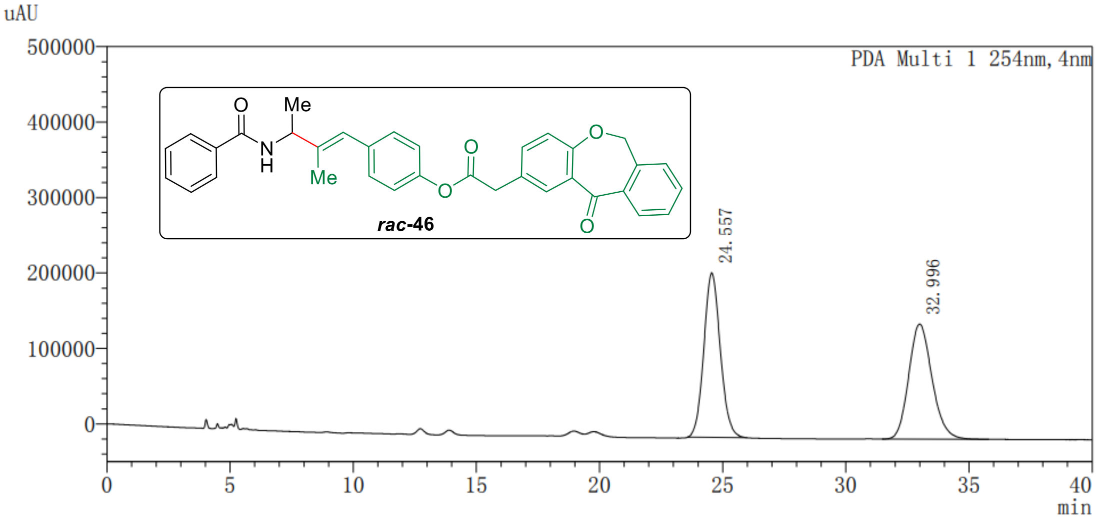

Peak#	Ret. Time	Area	Area%
1	24.557	10150676	50.339
2	32.996	10013944	49.661
Total		20164620	100

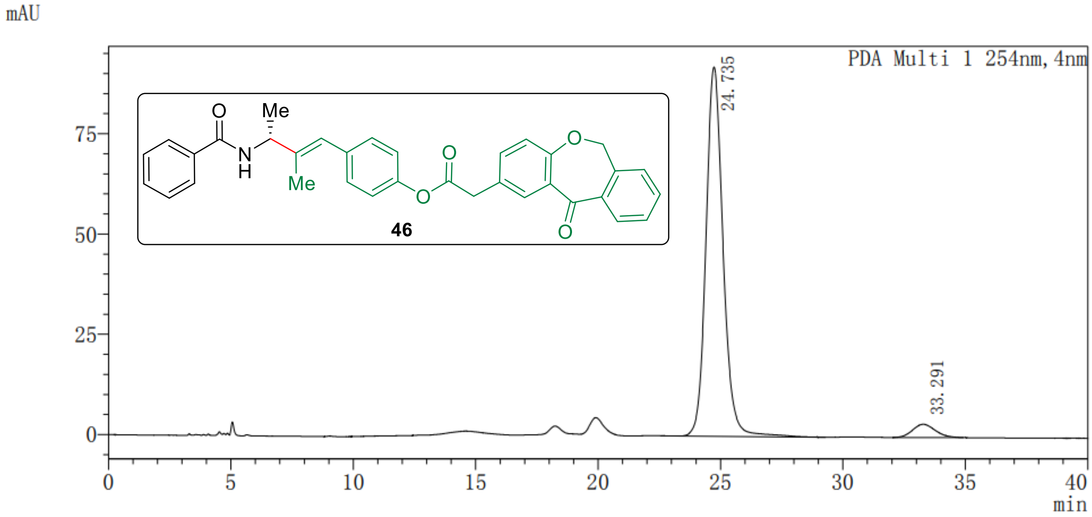

Peak#	Ret. Time	Area	Area%
1	24.735	4468529	95.462
2	33.291	212434	4.538
Total		4680963	100

Supplementary Figure 192. Chiral HPLC analysis of Compound **46**

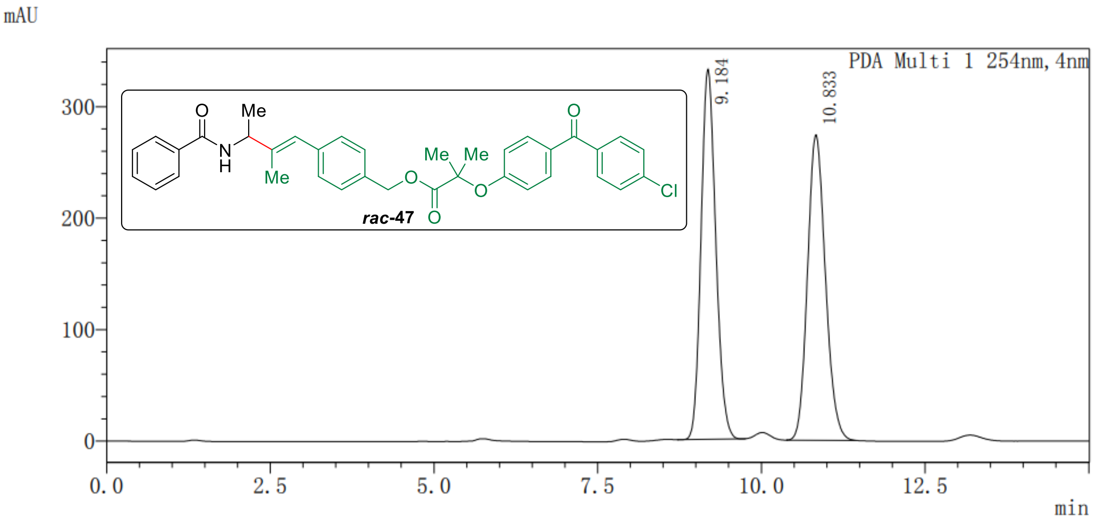

Peak#	Ret. Time	Area	Area%
1	9.184	5194472	49.875
2	10.833	5220554	50.125
Total		10415026	100

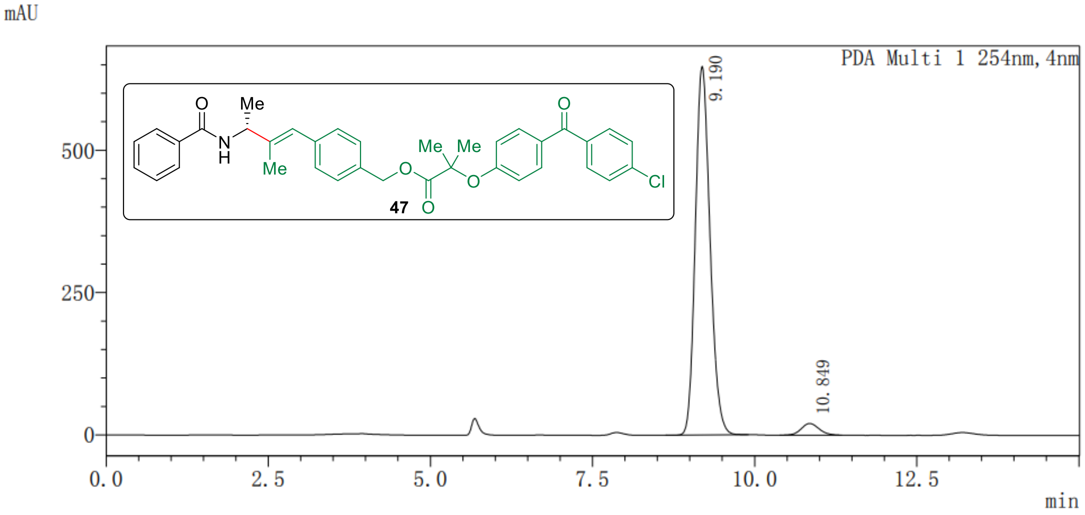

Peak#	Ret. Time	Area	Area%
1	9.190	10206457	96.297
2	10.849	392501	3.703
Total		10598958	100

Supplementary Figure 193. Chiral HPLC analysis of Compound **47**

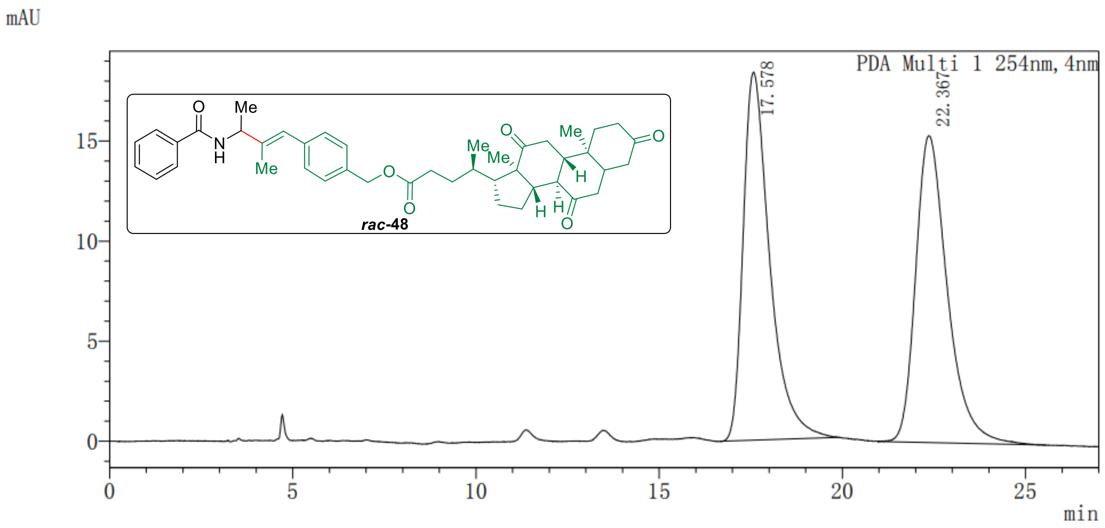

Peak#	Ret. Time	Area	Area%
1	17.578	925156	50.085
2	22.367	922007	49.915
Total		1847163	100

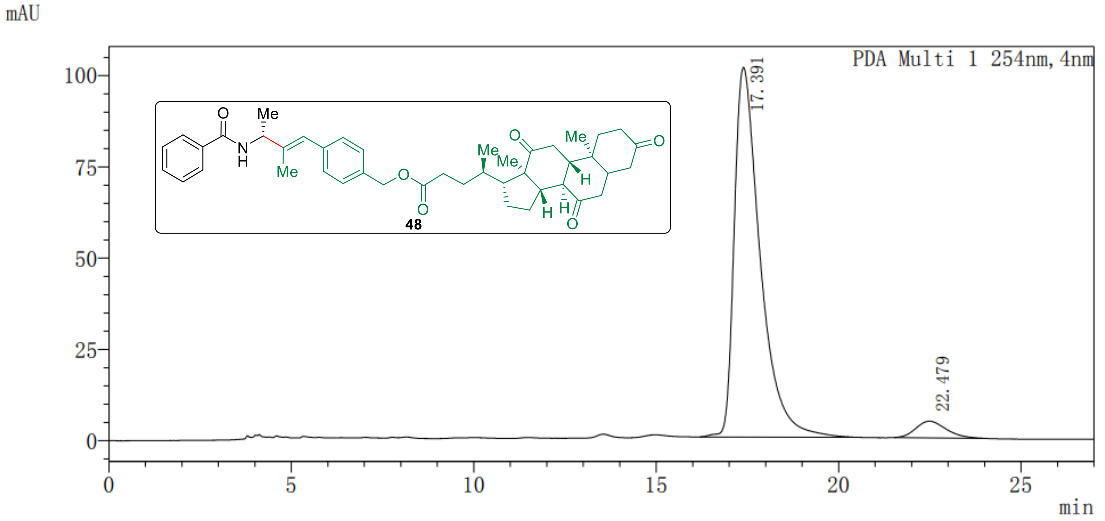

Peak#	Ret. Time	Area	Area%
1	17.391	5006403	95.004
2	22.479	263281	4.996
Total		5269684	100

Supplementary Figure 194. Chiral HPLC analysis of Compound **48**

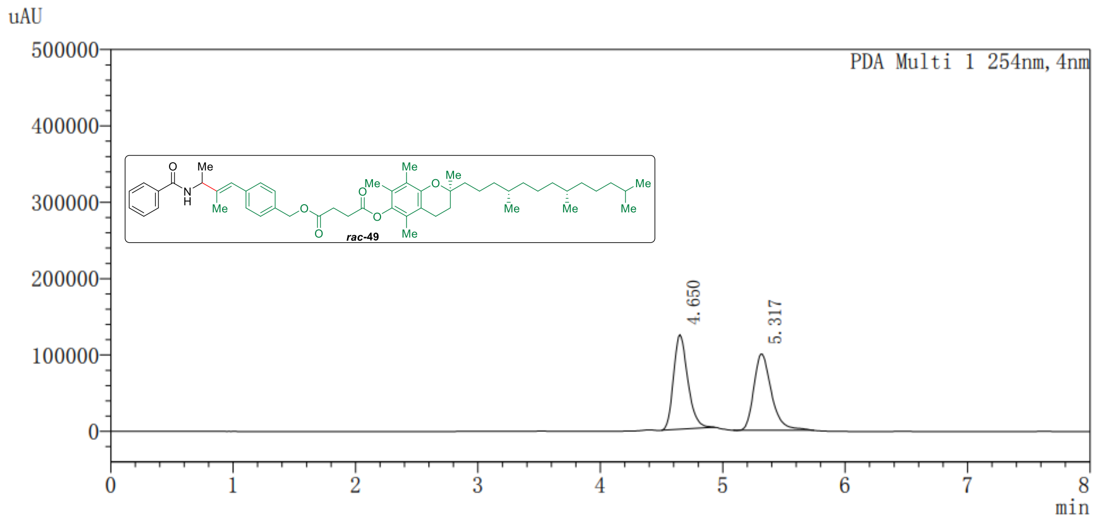

Peak#	Ret. Time	Area	Area%
1	4.650	1003401	50.002
2	5.317	1003322	49.998
Total		2006723	100

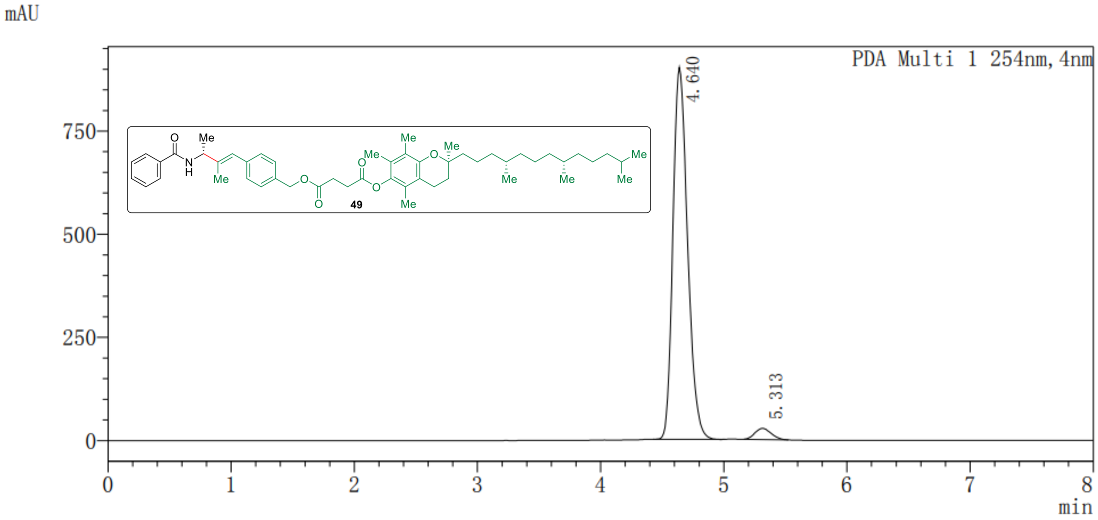

Peak#	Ret. Time	Area	Area%
1	4.640	7449056	96.804
2	5.313	245929	3.196
Total		7694985	100

Supplementary Figure 195. Chiral HPLC analysis of Compound **49**

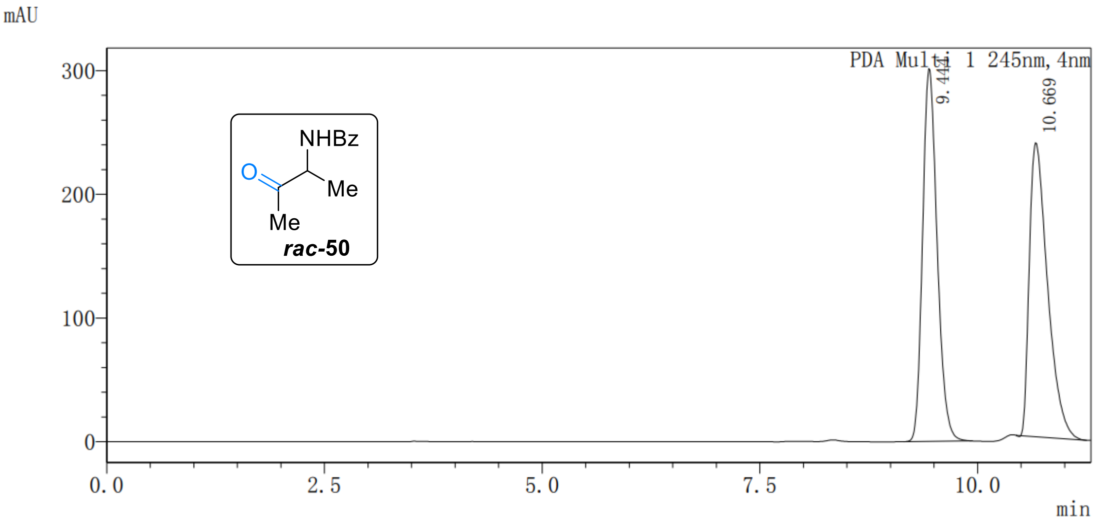

Peak#	Ret. Time	Area	Area%
1	9.444	3495273	50.222
2	10.669	3464367	49.778
Total		6959640	100

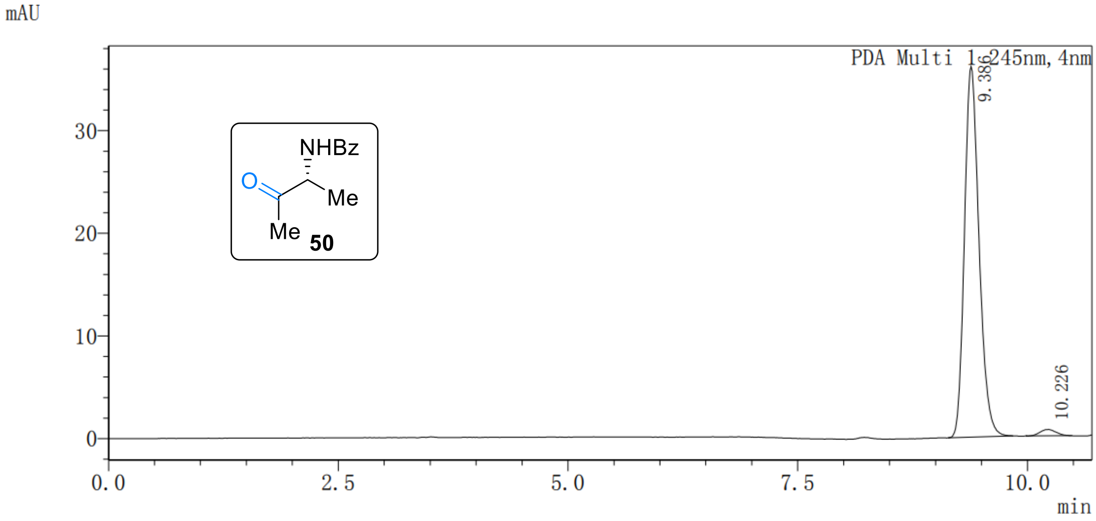

Peak#	Ret. Time	Area	Area%
1	9.386	397903	98.101
2	10.226	7703	1.899
Total		405606	100

Supplementary Figure 196. Chiral HPLC analysis of Compound **50**

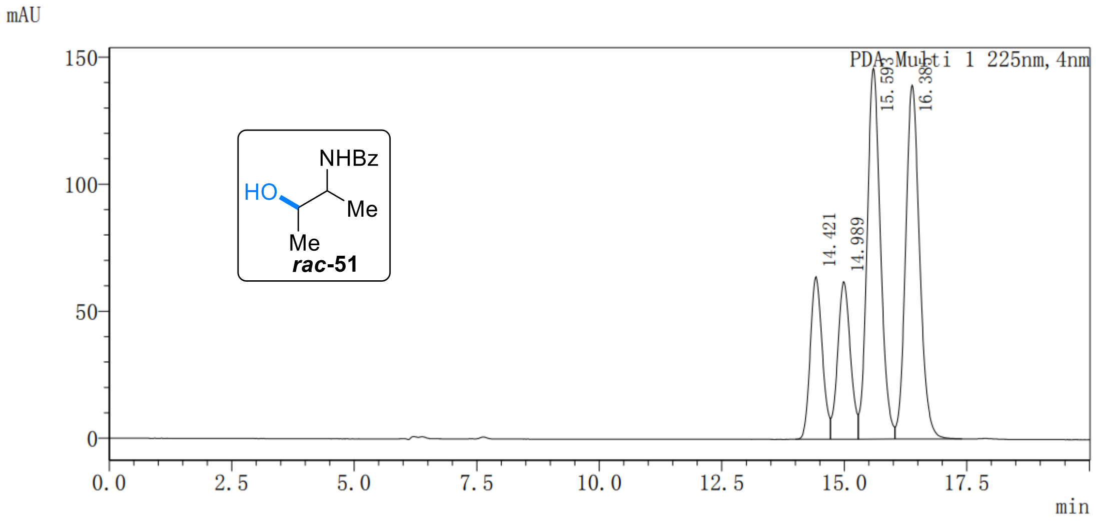

Peak#	Ret. Time	Area	Area%
1	14.421	1096003	14.137
2	14.989	1122120	14.474
3	15.593	2767895	35.701
4	16.385	2766905	35.689
Total		7752922	100.000

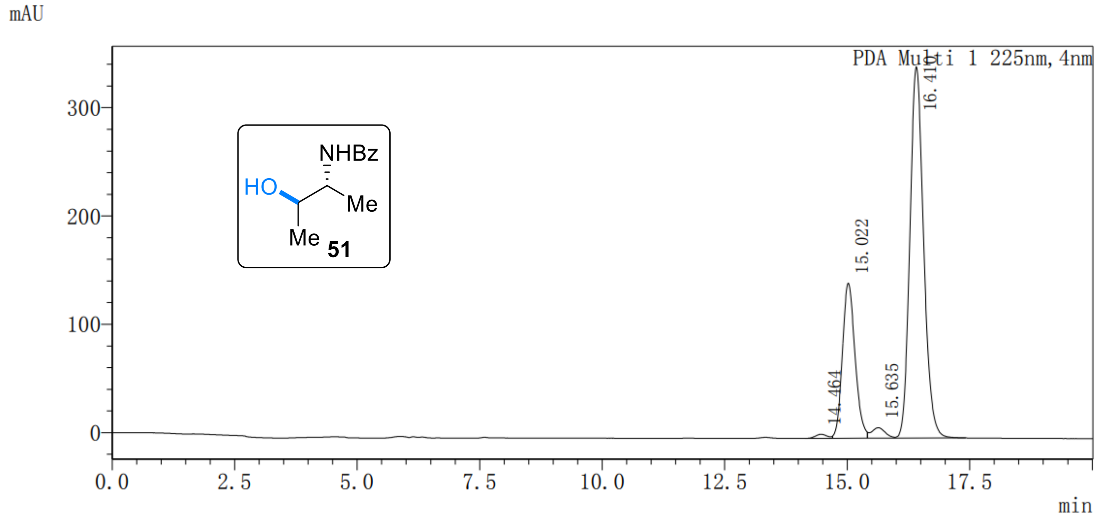

Peak#	Ret. Time	Area	Area%
1	14.464	64103	0.674
2	15.022	2566361	26.995
3	15.635	188868	1.987
4	16.410	6687494	70.344
Total		9506826	100.000

Supplementary Figure 197. Chiral HPLC analysis of Compound **51**

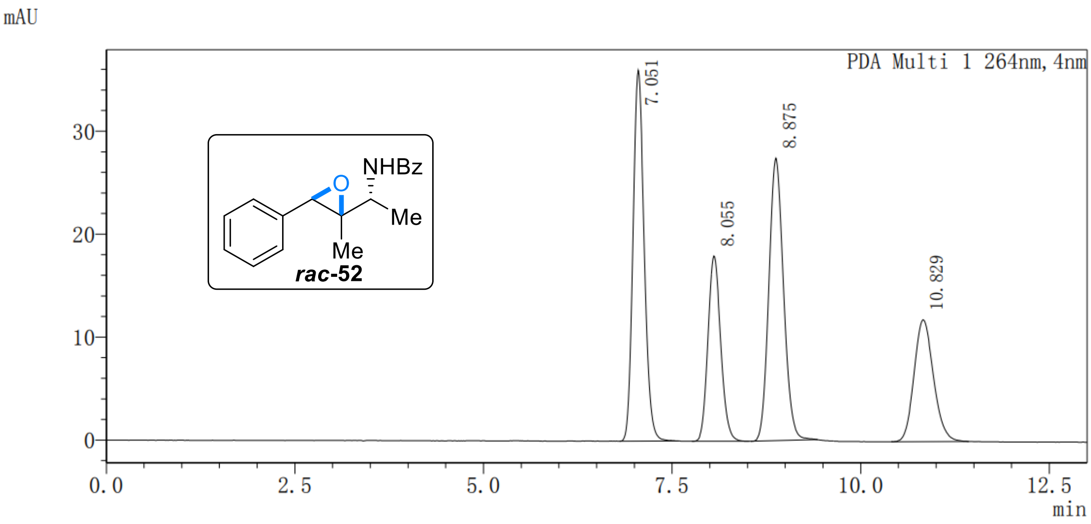

Peak#	Ret. Time	Area	Area%
1	7.051	371102	31.772
2	8.055	212688	18.209
3	8.875	372368	31.881
4	10.829	211853	18.138
Total		1168010	100.000

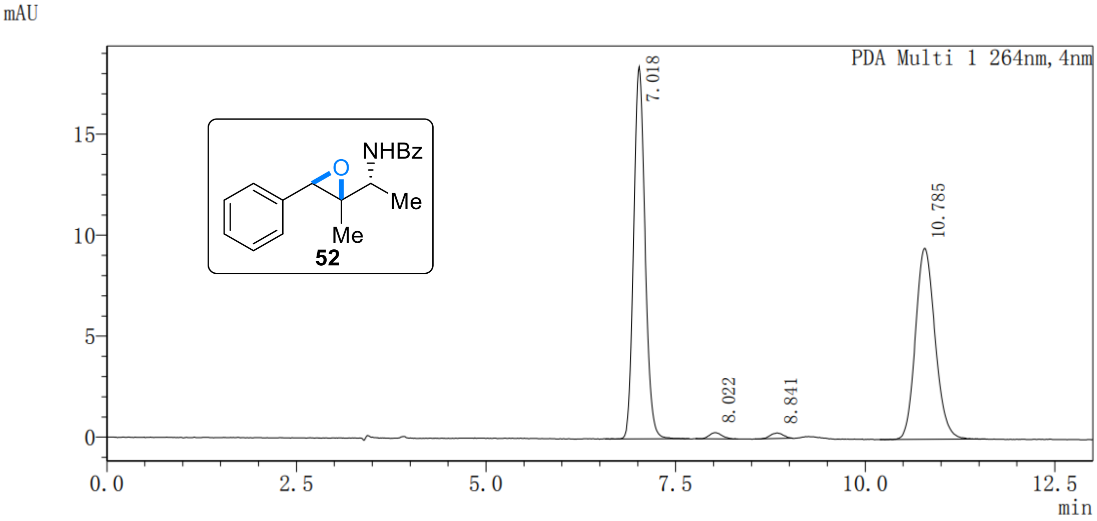

Peak#	Ret. Time	Area	Area%
1	7.018	191276	52.015
2	8.022	3433	0.934
3	8.841	3336	0.907
4	10.785	169689	46.144
Total		367734	100.000

Supplementary Figure 198. Chiral HPLC analysis of Compound **52**

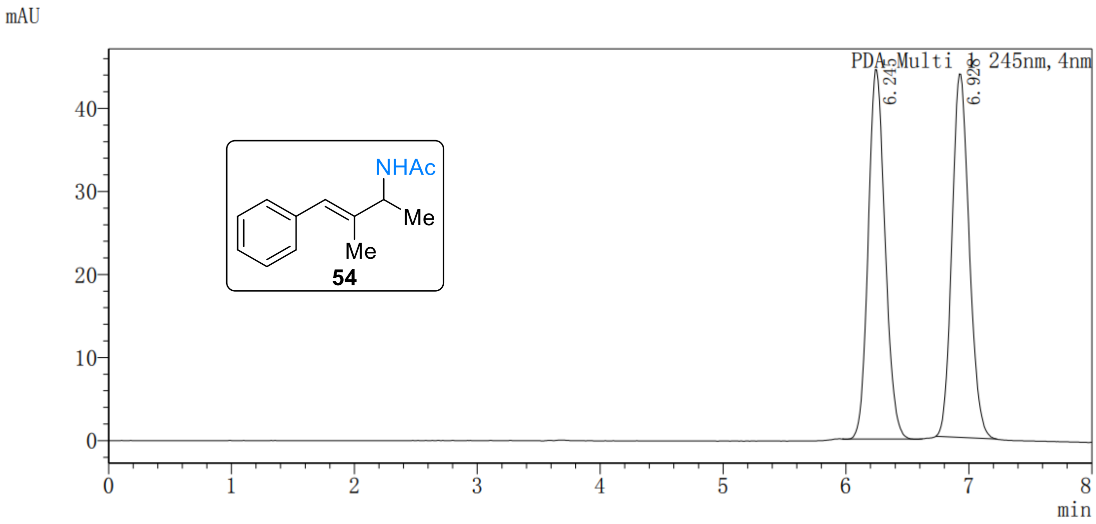

Peak#	Ret. Time	Area	Area%
1	6.245	424553	49.871
2	6.928	426757	50.129
Total		851310	100.000

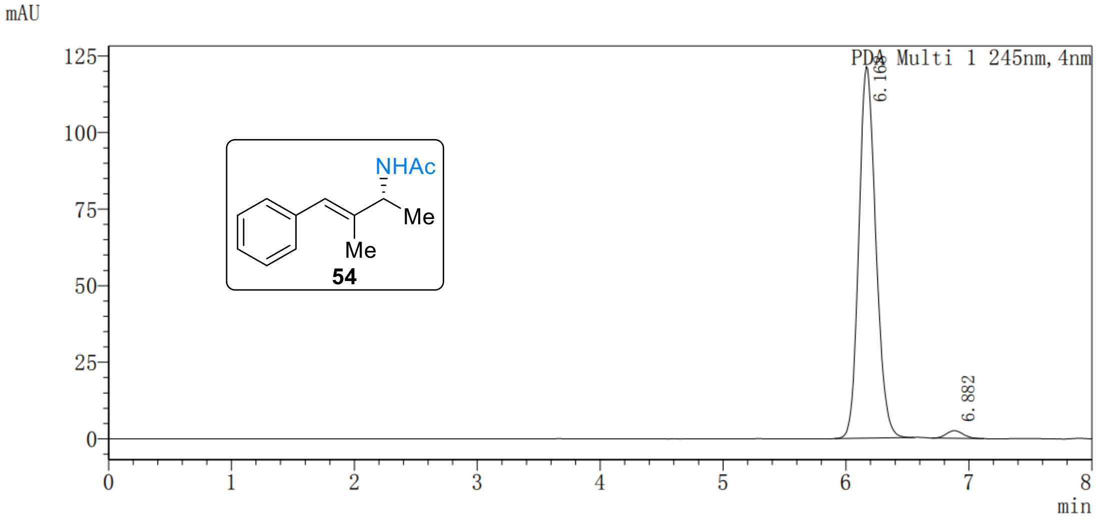

Peak#	Ret. Time	Area	Area%
1	6.168	1166261	97.994
2	6.882	23874	2.006
Total		1190136	100.000

Supplementary Figure 199. Chiral HPLC analysis of Compound **54**

Supplementary References

1. Shen, M.-L., Shen, Y. & Wang, P.-S. Merging Visible-Light Photoredox and Chiral Phosphate Catalysis for Asymmetric Friedel–Crafts Reaction with in Situ Generation of N-Acyl Imines. *Org. Lett.* **21**, 2993-2997 (2019).

2. Chen, J. & Zhu, S.-L. Nickel-Catalyzed Multicomponent Coupling: Synthesis of α-Chiral Ketones by Reductive Hydrocarbonylation of Alkenes. *J. Am. Chem. Soc.* **143**, 14089-14096 (2021).

3. Lu, H.-J., Li, C.-Q., Jiang, H.-L., Lizardi, C. L. & Zhang, X. P. Chemoselective Amination of Propargylic C(sp3)-H Bonds by Cobalt(II)-Based Metalloradical Catalysis. *Angew. Chem. Int. Ed.* **53**, 7028-7032 (2014).

4. Malpani, Y. R., Biswas, B. K., Han, H. S., Jung, Y.-S. & Han, S. B. Multicomponent Oxidative Trifluoromethylation of Alkynes with Photoredox Catalysis: Synthesis of α-Trifluoromethyl Ketones. *Org. Lett.* **20**, 1693-1697 (2018).

5. Tomita, R., Koike, T. & Akita, M. Photoredox-Catalyzed Stereoselective Conversion of Alkynes into Tetrasubstituted Trifluoromethylated Alkenes. *Angew. Chem. Int. Ed.* **54**, 12923-12927 (2015).
